# The ground beetles (Coleoptera: Carabidae) of the Strandzha Mountain and adjacent coastal territories (Bulgaria and Turkey)

**DOI:** 10.3897/BDJ.4.e8135

**Published:** 2016-04-01

**Authors:** Rumyana Kostova, Borislav Guéorguiev

**Affiliations:** ‡ Sofia University, Faculty of Biology, Sofia, Bulgaria; § National Museum of Natural History Sofia, Sofia, Bulgaria

**Keywords:** Carabidae, Strandzha, Yildiz, Bulgaria, European part of Turkey

## Abstract

**Background:**

The knowledge of the ground-beetle fauna of Strandzha is currently incomplete, and is largely based on data from the Bulgarian part of the region and on records resulting from casual collecting. This study represents a critical revision of the available literature, museum collections and a three years field study of the carabid beetles of the Bulgarian and Turkish parts of Strandzha Mountain and the adjacent Black Sea Coast territories.

**New information:**

A total of 328 species and subspecies of Carabidae, belonging to 327 species from the region of Strandzha Mountain and adjacent seacoast area, have been listed. Of these, 77 taxa represent new records for the Bulgarian part of the region, and 110 taxa new records for Turkish part of the studied region.

Two taxa, one subgenus (*Haptotapinus* Reitter, 1886) and one species (*Pterostichus crassiusculus*), are new to the fauna of Bulgaria. Based on a misidentification, the species *Apotomus testaceus* is excluded from the list of the Bulgarian fauna. Seven species (*Carabus violaceus azurescens*, *Apotomus rufus*, *Platynus proximus*, *Molops alpestris kalofericus*, *M. dilatatus angulicollis*, *Pterostichus merklii*, and *Calathus metallicus*) are treated as doubtful for the regional fauna, and one (*Apotomus rufus*) also for the Bulgarian fauna.

Altogether, 43 taxa collected in the Turkish part of the region are new for European Turkey. New taxa for Turkey are the genera *Myas* and *Oxypselaphus*, the subgenus *Feronidius*, and nine species and subspecies (*Carabus granulatus granulatus*, *Dyschirius tristis*, *Bembidion normannum apfelbecki*, *B. subcostatum vau*, *Acupalpus exiguus*, *Myas chalybaeus*, *Oxypselaphus obscurus*, *Pterostichus leonisi*, *Pt. melas*). In addition, there are a further seven species that are here confirmed for Turkey.

## Introduction

Strandzha (or Yildiz, in Turkish) is a low altitude mountain located at the southeast corner of the Balkan Peninsula. In fact, it is the last extensive European massif close to the Bosphorus, the point dividing Europe and Asia. Strandzha extends to both sides of the frontier between Bulgaria and Turkey, and the largest part of the mountain is in the latter country. It is bordered on the east by the Black Sea coast, and on the west by the Derventski Vazvisheniya Hills. The mountain has a number of physical characteristics that contribute to its high level of species diversity, such as relatively low altitude combined with varied mountainous relief, a wide variety of habitats, relatively well preserved natural ecosystems, and a mild climate for the proximity of the Black Sea and the Mediterranean. The vegetation has a markedly relict character with specific Euxinian floral elements (Georgiev 1993).

The fact that Strandzha’s invertebrate fauna is poorly investigated is largely due to the borderline location of the mountain. A vast area of the mountain was mostly inaccessible until recently. However, this helped to conserve some of the most interesting and species-rich ecosystems throughout Bulgaria and Turkey.

Knowledge of the ground-beetle fauna of Strandzha is considered insufficient and largely based on data from the Bulgarian part of the region and the available faunal records from casual collecting. The first records were published in the work of Czech entomologist Rambousek (1912). His paper includes 10 species from the region with the locality indicated as “Strandzha Mt.” [in Bulgarian], collected during the spring of the year 1909. In the twenties and thirties of the 20th century, data on eight representatives of the tribes Cicindelini and Carabini were added by Breuning (1928), Breuning (1932), Buresch and Kantardjieva (1928), Kantardjieva (1928) and Kantardjieva (1934). Later Mařan (1933), Mařan (1944), Mařan (1949) and Drensky et al. (1951) published supplementary information for six species. Fragmentary data for carabids from Strandzha appeared also in the works of Hieke (1970) – 1 species, Pawlowski (1973) – 5 species, Mlynář (1977) – 2 species, Müller-Motzfeld (1986) – 2 species, Wrase (1987) – 1 species, Casale (1988) – 1 species, Hůrka (1988) – 3 species, Vassilev and Necheva (1989) – 1 species and Wrase (1989) – 1 species. The most important contribution with respect to the carabids of the Bulgarian part of Strandzha and the adjacent Black Sea Coast, was the catalogue of Hieke and Wrase (1988) and its supplement (Wrase 1991). These two works included 169 and 9 species, respectively, or altogether 178 species. Guéorguiev (1992) published records for 15 species, based on material in Czech and Bulgarian collections, identified by the late Prof. O. L. Kryzhanovskij and a few Czech specialists. The second most significant contribution to the Bulgarian part of the region appeared to be the catalogue of Guéorguiev and Guéorguiev (1995), in which 61 species in total were mentioned. From the beginning of the XXI century until the present, only scanty information has been added: Guéorguiev and Muilwijk (2000) – 2 species, Guéorguiev and Muilwijk (2001) – 2 species, Guéorguiev (2001) – 1 species, Guéorguiev (2011) – 2 species and Guéorguiev and Kostova (2011) – 1 species. However, among the eight taxa most recently recorded are the only local endemics known from the study area: Cymindis (Paracymindis) vassili Guéorguiev, 2001 and Duvalius (Biharotrechus) bekchievi Guéorguiev, 2011.

Up to now, records from the Turkish section of Strandzha Mt. were largerly lacking. Data from there were published only by Battoni and Vereschagina (1984) - 2 species of *Calathus* s.str. Bonelli, 1810, as well as by Guéorguiev (2011) and Lapeva-Gjonova et al. (2011) - for a species of *Abax* Bonelli, 1810 and a species of *Paussus* Linnaeus, 1775, respectivelly.

The aim of this study is to expand the scarce information available on the carabid beetle fauna of the Strandzha Mountain and adjacent Black Sea Coast.

## Materials and methods

The present study combines the results from the revision of the known literature on the carabids of Strandzha and the adjacent Black Sea Coast, a revision of specimens from the region in collections of the National Museum of Natural History, Sofia – BAS (NMNHS) and Národni muzeum Prague (NMP), and a comprehensive study of the ground beetle assemblages conducted during the period of 2009-2011. This study was possible as a scientific project funded by the Bulgarian Ministry of Education and Science.

The study area comprises the Strandzha Mountain (Bulgarian and Turkish parts) and the adjacent Black Sea coast, due to the lack of clear border, either geographic or biotic, between the mountain and the coastline. The study area and main collection localities where the material was collected in 2009-2011.

The collecting methods used for the field study were as follows: manual collection under tree bark and stones and substrate sifting (soil, leaf litter, etc.) by sifter with aperture dimensions of 6 and 10 mm. The material was checked directly in the field or placed in Winkler-Mokzarski extractors. Pitfall traps comprising 500 ml containers filled with propylene glycol as a preservative, were set in transects in selected sites. Light traps comprised a tower with two types of light sources: 160W MBTF and F8T5 – 365 nm. Vacuum sampling from different substrates with a modified leaf blower Partner GBV 325. The material from pitfall traps was collected monthly from May to October 2009. The other methods of collection aim to cover a maximum number of habitats in the region and are non-systematic. Manual sampling, sifting and vacuum sampling were performed in all studied localities during 2009-2011. During the same period, light trapping was used at different localities for 1 - 3 days per month, depending on the weather conditions. The collected material was preserved in tubes filled with 70% ethanol. Determination of the species was performed using a Zeiss - Stemi 2000 stereomicroscope. Carabidae voucher specimens collected during this study are deposited at the Department of Zoology and Anthropology, Faculty of Biology, Sofia University.

## Checklists

### List of ground beetles species from Strandzha Mountain and the adjacent Black Sea Coast

#### Leistus (Pogonophorus) parvicollis

Chaudoir, 1869

##### Materials

- **Type status:** Other material. **Location:** countryCode: BG; locality: Malko Tarnovo; **Record Level:** bibliographicCitation: Guéorguiev (1992: 62)

#### Leistus (Pogonophorus) rufomarginatus

(Duftschmid, 1812)

##### Materials

- **Type status:** Other material. **Occurrence:** recordedBy: R. Kostova; individualCount: 1; **Location:** countryCode: BG; locality: Belevren Vill. surroundings; verbatimElevation: 485; verbatimCoordinates: N42°06'34.3", E27°09'58.1"; geodeticDatum: WGS84; **Event:** eventDate: 05/05/2009
- **Type status:** Other material. **Occurrence:** recordedBy: R. Kostova; individualCount: 1; **Location:** countryCode: BG; locality: Varovnik Vill., near the bridge at Fakiiska River; verbatimElevation: 125; verbatimCoordinates: N42°13'33.7", E27°11'33.7"; geodeticDatum: WGS84; **Event:** eventDate: 09/06/2009
- **Type status:** Other material. **Occurrence:** recordedBy: R. Kostova; individualCount: 2; **Location:** countryCode: BG; locality: Malko Tarnovo, the Tracian tumbs; verbatimElevation: 383; verbatimCoordinates: N41°58'54.6", E27°29'27.8"; geodeticDatum: WGS84; **Event:** eventDate: 27/05/2010
- **Type status:** Other material. **Occurrence:** recordedBy: R. Kostova; individualCount: 23; **Location:** countryCode: BG; locality: Slivarovo Vill., "Shafariitsa" Place; verbatimElevation: 224; verbatimCoordinates: N41°57'37.8", E27°39'34.8"; geodeticDatum: WGS84; **Event:** eventDate: 15.04-02.07.2009; habitat: black alder-oak forest
- **Type status:** Other material. **Occurrence:** recordedBy: R. Kostova; individualCount: 1; **Location:** countryCode: BG; locality: Kondolovo Vill., "Byalata prast" Place; verbatimElevation: 283; verbatimCoordinates: N42°05'49.7", E27°39'56.3"; geodeticDatum: WGS84; **Event:** eventDate: 15.04-08.05.2009; habitat: beech forest with Vaccinium arctostaphylos
- **Type status:** Other material. **Location:** countryCode: BG; locality: Malko Tarnovo; **Record Level:** bibliographicCitation: Guéorguiev (1992: 62)

#### Leistus (Pogonophorus) spinibarbis rufipes

Chaudoir, 1843

##### Materials

- **Type status:** Other material. **Occurrence:** recordedBy: R. Kostova; individualCount: 3; **Location:** countryCode: TR; locality: Demirköy surroundings; verbatimElevation: 655; verbatimCoordinates: N41°47'43.1", E27°44'13.3"; geodeticDatum: WGS84; **Event:** eventDate: 25/05/2010

#### Nebria (Nebria) brevicollis brevicollis

(Fabricius, 1792)

##### Materials

- **Type status:** Other material. **Occurrence:** recordedBy: R. Kostova; individualCount: 1; **Location:** countryCode: BG; locality: Near "Kachul" Place; verbatimElevation: 91; verbatimCoordinates: N42°01'57.1", E27°37'12.3"; geodeticDatum: WGS84; **Event:** eventDate: 17/04/2009
- **Type status:** Other material. **Occurrence:** recordedBy: R. Bekchiev; individualCount: 1; **Location:** countryCode: BG; locality: Sinemorets Vill., PA "Estuary of Veleka river"; verbatimElevation: 6; verbatimCoordinates: N42°03'39.6", E27°57'55.2"; geodeticDatum: WGS84; **Event:** eventDate: 18/04/2009
- **Type status:** Other material. **Occurrence:** recordedBy: R. Kostova; individualCount: 1; **Location:** countryCode: BG; locality: Varvara Vill. surroundings; verbatimElevation: 46; verbatimCoordinates: N42°06'49.1", E27°53'18.9"; geodeticDatum: WGS84; **Event:** eventDate: 10/05/2009
- **Type status:** Other material. **Occurrence:** recordedBy: A. Gijonova; individualCount: 1; **Location:** countryCode: TR; locality: Demirköy, Dökümhanesi; verbatimElevation: 179; verbatimCoordinates: N41°49'09.6", E27°48'43.2"; geodeticDatum: WGS84; **Event:** eventDate: 30/09/2009
- **Type status:** Other material. **Occurrence:** recordedBy: A. Gijonova; individualCount: 2; **Location:** countryCode: TR; locality: Igneada surroundings; verbatimElevation: 3; verbatimCoordinates: N41°51'27.3", E27°57'28.7"; geodeticDatum: WGS84; **Event:** eventDate: 02/10/2009; habitat: marsh
- **Type status:** Other material. **Occurrence:** recordedBy: R. Kostova; individualCount: 1; **Location:** countryCode: TR; locality: Demirköy surroundings; verbatimElevation: 655; verbatimCoordinates: N41°47'43.1", E27°44'13.3"; geodeticDatum: WGS84; **Event:** eventDate: 25/05/2010
- **Type status:** Other material. **Occurrence:** recordedBy: R. Kostova; individualCount: 1; **Location:** countryCode: TR; locality: Igneada, Hamam Gölü; verbatimElevation: 11; verbatimCoordinates: N41°49'31.6", E27°57'33.8"; geodeticDatum: WGS84; **Event:** eventDate: 25/05/2010
- **Type status:** Other material. **Occurrence:** recordedBy: R. Kostova; individualCount: 1; **Location:** countryCode: BG; locality: Malko Tarnovo surroundings; verbatimElevation: 375; verbatimCoordinates: N41°59'01.7", E27°29'49.4"; geodeticDatum: WGS84; **Event:** eventDate: 27/05/2010
- **Type status:** Other material. **Occurrence:** recordedBy: R. Kostova; individualCount: 2; **Location:** countryCode: TR; locality: Kiyiköy surroundings; verbatimElevation: 38; verbatimCoordinates: N41°39'25.7", E28°05'11.2"; geodeticDatum: WGS84; **Event:** eventDate: 22/05/2011
- **Type status:** Other material. **Occurrence:** recordedBy: R. Kostova; individualCount: 2; **Location:** countryCode: TR; locality: Sergen Vill. surroundings; verbatimElevation: 818; verbatimCoordinates: N41°44'37.0", E27°42'24.8"; geodeticDatum: WGS84; **Event:** eventDate: 25/05/2011
- **Type status:** Other material. **Occurrence:** recordedBy: R. Kostova; individualCount: 1; **Location:** countryCode: BG; locality: Bliznak Vill., PA"Bataka"; verbatimElevation: 324; verbatimCoordinates: N42°11'37.3", E27°19'35.8"; geodeticDatum: WGS84; **Event:** eventDate: 09.05-08.06.2009; habitat: oak forest
- **Type status:** Other material. **Occurrence:** recordedBy: R. Kostova; individualCount: 37; **Location:** countryCode: BG; locality: Slivarovo Vill., "Shafariitsa" Place; verbatimElevation: 224; verbatimCoordinates: N41°57'37.8", E27°39'34.8"; geodeticDatum: WGS84; **Event:** eventDate: 09.05-02.08.2009; habitat: black alder-oak forest
- **Type status:** Other material. **Occurrence:** recordedBy: R. Kostova; individualCount: 35; **Location:** countryCode: BG; locality: Kosti Vill., "St. Ilia" Place; verbatimElevation: 35; verbatimCoordinates: N42°03'23.2", E27°45'51.6"; geodeticDatum: WGS84; **Event:** eventDate: 15.04-02.07.2009; habitat: meadow with single trees
- **Type status:** Other material. **Occurrence:** recordedBy: R. Kostova; individualCount: 8; **Location:** countryCode: BG; locality: Sinemorets Vill., PA "Estuary of Veleka river"; verbatimElevation: 6; verbatimCoordinates: N42°03'39.6", E27°57'55.2"; geodeticDatum: WGS84; **Event:** eventDate: 09.05-02.08.2009; habitat: swamp forest
- **Type status:** Other material. **Occurrence:** recordedBy: R. Kostova; individualCount: 1; **Location:** countryCode: BG; locality: Mladezhko Vill., the springs of Mladezhka River; verbatimElevation: 231; verbatimCoordinates: N42°09'04.5", E27°21'26.1"; geodeticDatum: WGS84; **Event:** eventDate: 09.05-08.06.2009; habitat: black alder and hornbeam forest
- **Type status:** Other material. **Occurrence:** recordedBy: R. Kostova; individualCount: 1; **Location:** countryCode: TR; locality: Igneada surroundings; verbatimElevation: 15; verbatimCoordinates: N41°51'51.0", E27°56'54.1"; geodeticDatum: WGS84; **Event:** eventDate: 06.07-29.09.2009; habitat: swamp forest
- **Type status:** Other material. **Occurrence:** recordedBy: J. Mařan & K. Taborsky; individualCount: 10; **Location:** countryCode: BG; locality: Maslen nos Cape; **Event:** eventDate: VI.1933; **Record Level:** institutionCode: NMP
- **Type status:** Other material. **Location:** countryCode: BG; locality: Strandzha; **Record Level:** bibliographicCitation: Rambousek (1912: 67)
- **Type status:** Other material. **Occurrence:** recordedBy: P. Drenski; individualCount: 2; **Location:** countryCode: BG; locality: Rezovo Vill.; **Event:** eventDate: 15/05/1931; **Record Level:** institutionCode: NMNHS
- **Type status:** Other material. **Occurrence:** recordedBy: P. Beron; individualCount: 1; **Location:** countryCode: BG; locality: Kiten; **Event:** eventDate: 16-22.12.1984; **Record Level:** institutionCode: NMNHS
- **Type status:** Other material. **Occurrence:** recordedBy: B. Guéorguiev; individualCount: 5; **Location:** countryCode: BG; locality: West of Varovnik Vill.; **Event:** eventDate: 24-25.05.1995; habitat: oak forest, in night; **Record Level:** institutionCode: NMNHS
- **Type status:** Other material. **Occurrence:** recordedBy: B. Guéorguiev; individualCount: 1; **Location:** countryCode: BG; locality: Aydere River; verbatimElevation: 350-450; **Event:** eventDate: 26/05/1995; **Record Level:** institutionCode: NMNHS
- **Type status:** Other material. **Occurrence:** recordedBy: B. Guéorguiev; individualCount: 1; **Location:** countryCode: BG; locality: mouth of Veleka River near Sinemorets Vill.; **Event:** eventDate: 28/05/1995; **Record Level:** institutionCode: NMNHS

#### Notiophilus bigutatus

(Fabricius, 1779)

##### Materials

- **Type status:** Other material. **Occurrence:** recordedBy: R. Kostova; individualCount: 1; **Location:** countryCode: BG; locality: Kondolovo Vill., "Byalata prast" Place; verbatimElevation: 271; verbatimCoordinates: N42°05'47.7", E27°39'55.0"; geodeticDatum: WGS84; **Event:** eventDate: 18/04/2009; habitat: oak-beech mixed forest
- **Type status:** Other material. **Occurrence:** recordedBy: R. Bekchiev; individualCount: 1; **Location:** countryCode: BG; locality: Brodilovo Vill., under Papiya peak; verbatimElevation: 336; verbatimCoordinates: N42°06'23.7", E27°50'36.1"; geodeticDatum: WGS84; **Event:** eventDate: 03/07/2009; habitat: oak forest
- **Type status:** Other material. **Occurrence:** recordedBy: B. Guéorguiev; individualCount: 1; **Location:** countryCode: BG; locality: Tisovitsa Reserve near Bulgari Vill.; **Event:** eventDate: 27/05/1995; **Record Level:** institutionCode: NMNHS
- **Type status:** Other material. **Occurrence:** recordedBy: R. Kostova; individualCount: 5; **Location:** countryCode: TR; locality: Demirköy, to Mahya Dağı peak; verbatimElevation: 488; verbatimCoordinates: N41°52'41.1", E27°54'27.0"; geodeticDatum: WGS84; **Event:** eventDate: 05/07/2009
- **Type status:** Other material. **Occurrence:** recordedBy: R. Kostova; individualCount: 1; **Location:** countryCode: TR; locality: Demirköy surroundings; verbatimElevation: 655; verbatimCoordinates: N41°47'43.1", E27°44'13.3"; geodeticDatum: WGS84; **Event:** eventDate: 25/05/2010
- **Type status:** Other material. **Occurrence:** recordedBy: R. Kostova; individualCount: 1; **Location:** countryCode: TR; locality: Sergen Vill. surroundings; verbatimElevation: 818; verbatimCoordinates: N41°44'37.0", E27°42'24.8"; geodeticDatum: WGS84; **Event:** eventDate: 26/05/2011

#### Notiophilus danieli

Reitter, 1897

##### Materials

- **Type status:** Other material. **Location:** countryCode: BG; locality: Ropotamo; **Record Level:** bibliographicCitation: Hieke & Wrase (1988: 25)
- **Type status:** Other material. **Location:** countryCode: BG; locality: Ropotamo; **Record Level:** bibliographicCitation: Guéorguiev & Guéorguiev (1995: 60)

#### Notiophilus palustris

(Duftschmid, 1812)

##### Materials

- **Type status:** Other material. **Occurrence:** recordedBy: R. Kostova; individualCount: 1; **Location:** countryCode: BG; locality: Slivarovo Vill., "Shafariitsa" Place; verbatimElevation: 224; verbatimCoordinates: N41°57'37.8", E27°39'34.8"; geodeticDatum: WGS84; **Event:** eventDate: 05-07-09
- **Type status:** Other material. **Occurrence:** recordedBy: R. Kostova; individualCount: 2; **Location:** countryCode: BG; locality: Slivarovo Vill., "Shafariitsa" Place; verbatimElevation: 224; verbatimCoordinates: N41°57'37.8", E27°39'34.8"; geodeticDatum: WGS84; **Event:** eventDate: 09.05-08.06.2009; habitat: black alder-oak forest
- **Type status:** Other material. **Location:** countryCode: BG; locality: Ropotamo; **Record Level:** bibliographicCitation: Hieke & Wrase (1988: 24)

#### Notiophilus rufipes

Curtis, 1829

##### Materials

- **Type status:** Other material. **Occurrence:** recordedBy: R. Kostova; individualCount: 1; **Location:** countryCode: BG; locality: Mladezhko Vill., along Mladezhka River; verbatimElevation: 248; verbatimCoordinates: N42°09'00.9", E27°21'44.2"; geodeticDatum: WGS84; **Event:** eventDate: 16/04/2009
- **Type status:** Other material. **Occurrence:** recordedBy: R. Kostova; individualCount: 1; **Location:** countryCode: BG; locality: Kondolovo Vill., "Marsevski dol" Place; verbatimElevation: 284; verbatimCoordinates: N42°06'02.7", E27°40'53.5"; geodeticDatum: WGS84; **Event:** eventDate: 18/04/2009
- **Type status:** Other material. **Occurrence:** recordedBy: R. Kostova; individualCount: 3; **Location:** countryCode: BG; locality: Stoilovo Vill., "Sredoka" Reserve, along Mechi dol River; verbatimElevation: 206; verbatimCoordinates: N42°01'51.1", E27°30'50.1"; geodeticDatum: WGS84; **Event:** eventDate: 19/04/2009
- **Type status:** Other material. **Occurrence:** recordedBy: R. Kostova; individualCount: 2; **Location:** countryCode: BG; locality: Slivarovo Vill., "Shafariitsa" Place; verbatimElevation: 224; verbatimCoordinates: N41°57'37.8", E27°39'34.8"; geodeticDatum: WGS84; **Event:** eventDate: 07/05/2009
- **Type status:** Other material. **Occurrence:** recordedBy: R. Kostova; individualCount: 1; **Location:** countryCode: BG; locality: Varvara Vill. surroundings; verbatimElevation: 46; verbatimCoordinates: N42°06'49.1", E27°53'18.9"; geodeticDatum: WGS84; **Event:** eventDate: 10/05/2009
- **Type status:** Other material. **Occurrence:** recordedBy: R. Kostova; individualCount: 1; **Location:** countryCode: BG; locality: Brodilovo Vill, along Eleshnitsa River; verbatimElevation: 17; verbatimCoordinates: N42°05'35.4", E27°49'53.0"; geodeticDatum: WGS84; **Event:** eventDate: 05/07/2009
- **Type status:** Other material. **Occurrence:** recordedBy: R. Kostova; individualCount: 1; **Location:** countryCode: TR; locality: Demirköy, along Degirmen River; verbatimElevation: 289; verbatimCoordinates: N41°49'17.2", E27°45'07.7"; geodeticDatum: WGS84; **Event:** eventDate: 06/07/2009
- **Type status:** Other material. **Occurrence:** recordedBy: A. Gijonova; individualCount: 2; **Location:** countryCode: TR; locality: Igneada surroundings; verbatimElevation: 20; verbatimCoordinates: N41°48'35.4", E27°57'51.6"; geodeticDatum: WGS84; **Event:** eventDate: 02/10/2009; habitat: marsh
- **Type status:** Other material. **Occurrence:** recordedBy: R. Kostova; individualCount: 1; **Location:** countryCode: TR; locality: Along Demirköy - Dupnisa Cave road; verbatimElevation: 337; verbatimCoordinates: N41°52'20.0", E27°37'06.4"; geodeticDatum: WGS84; **Event:** eventDate: 23/05/2010
- **Type status:** Other material. **Occurrence:** recordedBy: R. Kostova; individualCount: 1; **Location:** countryCode: TR; locality: Igneada, Hamam Gölü; verbatimElevation: 11; verbatimCoordinates: N41°49'31.6", E27°57'33.8"; geodeticDatum: WGS84; **Event:** eventDate: 25/05/2010
- **Type status:** Other material. **Occurrence:** recordedBy: R. Kostova; individualCount: 1; **Location:** countryCode: BG; locality: Stoilovo Vill., "Sredoka" Reserve, along Mechi dol River; verbatimElevation: 206; verbatimCoordinates: N42°01'51.1", E27°30'50.1"; geodeticDatum: WGS84; **Event:** eventDate: 27/05/2010
- **Type status:** Other material. **Occurrence:** recordedBy: R. Kostova; individualCount: 1; **Location:** countryCode: TR; locality: Kiyiköy surroundings; verbatimElevation: 38; verbatimCoordinates: N41°39'25.7", E28°05'11.2"; geodeticDatum: WGS84; **Event:** eventDate: 22/05/2011
- **Type status:** Other material. **Occurrence:** recordedBy: R. Kostova; individualCount: 2; **Location:** countryCode: TR; locality: Yalikŏy, river estuary; verbatimElevation: 9; verbatimCoordinates: N41°29'27.7", E28°16'40.0"; geodeticDatum: WGS84; **Event:** eventDate: 23/05/2011
- **Type status:** Other material. **Occurrence:** recordedBy: R. Kostova; individualCount: 1; **Location:** countryCode: TR; locality: Sivriler Vill. suroundings; verbatimElevation: 379; verbatimCoordinates: N41°47'30.2", E27°50'00.9"; geodeticDatum: WGS84; **Event:** eventDate: 24/05/2011
- **Type status:** Other material. **Occurrence:** recordedBy: R. Kostova; individualCount: 18; **Location:** countryCode: BG; locality: Bliznak Vill., PA"Bataka"; verbatimElevation: 324; verbatimCoordinates: N42°11'37.3", E27°19'35.8"; geodeticDatum: WGS84; **Event:** eventDate: 15.04-02.07.2009; habitat: oak forest
- **Type status:** Other material. **Occurrence:** recordedBy: R. Kostova; individualCount: 41; **Location:** countryCode: BG; locality: Slivarovo Vill., "Shafariitsa" Place; verbatimElevation: 224; verbatimCoordinates: N41°57'37.8", E27°39'34.8"; geodeticDatum: WGS84; **Event:** eventDate: 15.04-08.06.2009; habitat: black alder-oak forest
- **Type status:** Other material. **Occurrence:** recordedBy: R. Kostova; individualCount: 1; **Location:** countryCode: BG; locality: Kondolovo Vill., "Byalata prast" Place; verbatimElevation: 271; verbatimCoordinates: N42°05'47.7", E27°39'55.0"; geodeticDatum: WGS84; **Event:** eventDate: 15.04-08.05.2009; habitat: oak-beech mixed forest
- **Type status:** Other material. **Occurrence:** recordedBy: R. Kostova; individualCount: 1; **Location:** countryCode: BG; locality: Kondolovo Vill., "Byalata prast" Place; verbatimElevation: 283; verbatimCoordinates: N42°05'49.7", E27°39'56.3"; geodeticDatum: WGS84; **Event:** eventDate: 09.05-08.06.2009; habitat: beech forest with Vaccinium arctostaphylos
- **Type status:** Other material. **Occurrence:** recordedBy: R. Kostova; individualCount: 23; **Location:** countryCode: BG; locality: Bulgari Vill., PA "Marina reka"; verbatimElevation: 183; verbatimCoordinates: N42°06'41.7", E27°45'53.0"; geodeticDatum: WGS84; **Event:** eventDate: 15.04-02.07.2009; habitat: oriental beech-oak forest with Rododendron ponticum
- **Type status:** Other material. **Occurrence:** recordedBy: B. Guéorguiev; individualCount: 1; **Location:** countryCode: BG; locality: Veleka River near Mladezko Village; **Event:** eventDate: 25/05/1995; **Record Level:** institutionCode: NMNHS
- **Type status:** Other material. **Occurrence:** recordedBy: B. Guéorguiev; individualCount: 1; **Location:** countryCode: BG; locality: Rezovska River at Uzunbudzhak Reserve; **Event:** eventDate: 26/05/1995; **Record Level:** institutionCode: NMNHS
- **Type status:** Other material. **Occurrence:** recordedBy: B. Guéorguiev; individualCount: 2; **Location:** countryCode: BG; locality: Tisovitsa Reserve near Bulgari Vill.; **Event:** eventDate: 27/05/1995; **Record Level:** institutionCode: NMNHS
- **Type status:** Other material. **Occurrence:** recordedBy: B. Guéorguiev; individualCount: 2; **Location:** countryCode: BG; locality: Sinemorets Village, south bay; **Event:** eventDate: 27/05/1995; **Record Level:** institutionCode: NMNHS

#### Notiophilus substriatus

G.R. Waterhouse, 1833

##### Materials

- **Type status:** Other material. **Occurrence:** recordedBy: R. Kostova; individualCount: 1; **Location:** countryCode: BG; locality: Malko Tarnovo, the Tracian tumbs; verbatimElevation: 383; verbatimCoordinates: N41°58'54.6", E27°29'27.8"; geodeticDatum: WGS84; **Event:** eventDate: 27/05/2010
- **Type status:** Other material. **Occurrence:** recordedBy: R. Bekchiev; individualCount: 4; **Location:** countryCode: TR; locality: Demirköy, Mahya Dağı Peak; verbatimElevation: 820; verbatimCoordinates: N41°46'15.6", E27°38'18.0"; geodeticDatum: WGS84; **Event:** eventDate: 30/09/2009
- **Type status:** Other material. **Occurrence:** recordedBy: C. Purkyně; individualCount: 1; **Location:** countryCode: BG; locality: Maslen nos Cape; **Record Level:** institutionCode: NMP
- **Type status:** Other material. **Occurrence:** recordedBy: B. Guéorguiev; individualCount: 1; **Location:** countryCode: BG; locality: Ahtopol; **Event:** eventDate: 28/05/1995; **Record Level:** institutionCode: NMNHS
- **Type status:** Other material. **Location:** countryCode: BG; locality: Malko Tarnovo; **Record Level:** bibliographicCitation: Guéorguiev & Guéorguiev (1995: 60)

#### Calomera littoralis nemoralis

(Olivier, 1790)

##### Materials

- **Type status:** Other material. **Location:** countryCode: BG; locality: Strandzha; **Record Level:** bibliographicCitation: Kantardjieva (1928: 111, as C. lunulata nemoralis)
- **Type status:** Other material. **Location:** countryCode: BG; locality: Kiten; **Record Level:** bibliographicCitation: Hieke & Wrase (1988: 11, as Cicindela nemoralis)
- **Type status:** Other material. **Location:** countryCode: BG; locality: Primorsko; **Record Level:** bibliographicCitation: Hieke & Wrase (1988: 11, as Cicindela nemoralis)

#### Cicindela (Cicindela) campestris pontica

Fischer-Waldheim, 1828

##### Materials

- **Type status:** Other material. **Occurrence:** recordedBy: R. Kostova; individualCount: 1; **Location:** countryCode: BG; locality: Kondolovo Vill., "Byalata prast" Place; verbatimElevation: 271; verbatimCoordinates: N42°05'47.7", E27°39'55.0"; geodeticDatum: WGS84; **Event:** eventDate: 18/04/2009; habitat: oak-beech mixed forest
- **Type status:** Other material. **Occurrence:** recordedBy: R. Kostova; individualCount: 1; **Location:** countryCode: BG; locality: Slivarovo Vill., "Shafariitsa" Place; verbatimElevation: 224; verbatimCoordinates: N41°57'37.8", E27°39'34.8"; geodeticDatum: WGS84; **Event:** eventDate: 07/05/2009
- **Type status:** Other material. **Occurrence:** recordedBy: R. Kostova; individualCount: 1; **Location:** countryCode: TR; locality: Sergen Vill. surroundings; verbatimElevation: 818; verbatimCoordinates: N41°44'37.0", E27°42'24.8"; geodeticDatum: WGS84; **Event:** eventDate: 25/05/2011
- **Type status:** Other material. **Location:** countryCode: BG; locality: Kosti Vill.; **Record Level:** bibliographicCitation: Kantardjieva (1928: 106)
- **Type status:** Other material. **Occurrence:** recordedBy: S. Petrov; individualCount: 1; **Location:** countryCode: BG; locality: Vitanovo Reserve; **Event:** eventDate: 3-15.05.2000; **Record Level:** institutionCode: NMNHS

#### Cicindela (Cicindela) monticola rumelica

Apfelbeck, 1904

##### Materials

- **Type status:** Other material. **Occurrence:** recordedBy: E. Tasheva; individualCount: 1; **Location:** countryCode: BG; locality: Primorsko, Ropotamo Reserve; verbatimElevation: 23; verbatimCoordinates: N42°19'25.0", E27°44'01.0"; geodeticDatum: WGS84; **Event:** eventDate: 29/04/2011
- **Type status:** Other material. **Location:** countryCode: BG; locality: Ahtopol; **Record Level:** bibliographicCitation: Hieke & Wrase (1988: 10, as C. hybrida rumelica)
- **Type status:** Other material. **Location:** countryCode: BG; locality: Kiten; **Record Level:** bibliographicCitation: Hieke & Wrase (1988: 10, as C. hybrida rumelica)
- **Type status:** Other material. **Location:** countryCode: BG; locality: Tsarevo (= Micurin); **Record Level:** bibliographicCitation: Hieke & Wrase (1988: 10, as C. hybrida rumelica)
- **Type status:** Other material. **Location:** countryCode: BG; locality: Primorsko; **Record Level:** bibliographicCitation: Hieke & Wrase (1988: 10, as C. hybrida rumelica)
- **Type status:** Other material. **Occurrence:** recordedBy: T. Ljubomirov; individualCount: 2; **Location:** countryCode: BG; locality: North of Lozenets Vill.; verbatimElevation: 0; verbatimCoordinates: N42°13'00.0", E27°47'00.0"; **Event:** eventDate: 25/07/2006; **Record Level:** institutionCode: NMNHS

#### Calosoma (Calosoma) inquisitor inquisitor

(Linnaeus, 1758)

##### Materials

- **Type status:** Other material. **Occurrence:** recordedBy: R. Kostova; individualCount: 1; **Location:** countryCode: BG; locality: Kondolovo Vill., "Byalata prast" Place; verbatimElevation: 271; verbatimCoordinates: N42°05'47.7", E27°39'55.0"; geodeticDatum: WGS84; **Event:** eventDate: 18/04/2009; habitat: oak-beech mixed forest
- **Type status:** Other material. **Occurrence:** recordedBy: R. Kostova; individualCount: 2; **Location:** countryCode: BG; locality: Bulgari Vill., PA "Marina reka"; verbatimElevation: 172; verbatimCoordinates: N42°06'41.7", E27°45'53.0"; geodeticDatum: WGS84; **Event:** eventDate: 08/05/2009
- **Type status:** Other material. **Occurrence:** recordedBy: R. Kostova; individualCount: 2; **Location:** countryCode: BG; locality: Varvara Vill. surroundings; verbatimElevation: 46; verbatimCoordinates: N42°06'49.1", E27°53'18.9"; geodeticDatum: WGS84; **Event:** eventDate: 10/05/2009
- **Type status:** Other material. **Occurrence:** recordedBy: R. Kostova; individualCount: 2; **Location:** countryCode: TR; locality: Demirköy surroundings; verbatimElevation: 655; verbatimCoordinates: N41°47'43.1", E27°44'13.3"; geodeticDatum: WGS84; **Event:** eventDate: 25/05/2010
- **Type status:** Other material. **Occurrence:** recordedBy: R. Kostova; individualCount: 1; **Location:** countryCode: TR; locality: Vize surroundings; verbatimElevation: 366; verbatimCoordinates: N41°35'06.1", E27°47'41.5"; geodeticDatum: WGS84; **Event:** eventDate: 25/05/2011
- **Type status:** Other material. **Occurrence:** recordedBy: R. Kostova; individualCount: 1; **Location:** countryCode: BG; locality: Sinemorets Vill., PA"Silistar"; verbatimElevation: 23; verbatimCoordinates: N42°01'30.7", E28°00'25.4"; geodeticDatum: WGS84; **Event:** eventDate: 27/05/2011; habitat: oak forest
- **Type status:** Other material. **Occurrence:** recordedBy: R. Kostova; individualCount: 39; **Location:** countryCode: BG; locality: Brodilovo Vill., under Papiya peak; verbatimElevation: 336; verbatimCoordinates: N42°06'23.7", E27°50'36.1"; geodeticDatum: WGS84; **Event:** eventDate: 15.04-08.06.2009; habitat: oak forest
- **Type status:** Other material. **Occurrence:** recordedBy: R. Kostova; individualCount: 4; **Location:** countryCode: BG; locality: Kondolovo Vill., "Byalata prast" Place; verbatimElevation: 271; verbatimCoordinates: N42°05'47.7", E27°39'55.0"; geodeticDatum: WGS84; **Event:** eventDate: 09.05-08.06.2009; habitat: oak-beech mixed forest
- **Type status:** Other material. **Location:** countryCode: BG; locality: Brodilovo Vill.; **Record Level:** bibliographicCitation: Buresch & Kantardjieva (1928: 63)
- **Type status:** Other material. **Location:** countryCode: BG; locality: Kiten; **Record Level:** bibliographicCitation: Hieke & Wrase (1988: 12)
- **Type status:** Other material. **Occurrence:** recordedBy: P. Drenski; individualCount: 1; **Location:** countryCode: BG; locality: Strandzha; **Event:** eventDate: 03/08/1935
- **Type status:** Other material. **Occurrence:** recordedBy: S. Zagorchinov; individualCount: 2; **Location:** countryCode: BG; locality: Strandzha; **Event:** eventDate: 18/06/1973; **Record Level:** institutionCode: NMP
- **Type status:** Other material. **Occurrence:** recordedBy: S. Zagorchinov; individualCount: 1; **Location:** countryCode: BG; locality: Strandzha; **Event:** eventDate: 18/05/1978
- **Type status:** Other material. **Occurrence:** recordedBy: M. Josifov; individualCount: 1; **Location:** countryCode: BG; locality: Perla Place, north of Primorsko; **Event:** eventDate: 27/05/1973; **Record Level:** institutionCode: NMNHS
- **Type status:** Other material. **Occurrence:** recordedBy: B. Petrov; individualCount: 1; **Location:** countryCode: BG; locality: Maslen nos Cape; **Event:** eventDate: 07/04/1991; habitat: oak forest; **Record Level:** institutionCode: NMNHS

#### Calosoma (Calosoma) sycophanta

(Linnaeus, 1758)

##### Materials

- **Type status:** Other material. **Occurrence:** recordedBy: R. Kostova; individualCount: 1; **Location:** countryCode: BG; locality: Bliznak Vill., PA"Bataka"; verbatimElevation: 324; verbatimCoordinates: N42°11'37.3", E27°19'35.8"; geodeticDatum: WGS84; **Event:** eventDate: 09.05-08.06.2009; habitat: oak forest
- **Type status:** Other material. **Occurrence:** recordedBy: R. Kostova; individualCount: 1; **Location:** countryCode: BG; locality: Slivarovo Vll. surroundings; verbatimElevation: 340; verbatimCoordinates: N41°58'21.8", E27°39'11.9"; geodeticDatum: WGS84; **Event:** eventDate: 05-28-10
- **Type status:** Other material. **Location:** countryCode: BG; locality: Near Brodilovo Vill.; **Record Level:** bibliographicCitation: Buresch & Kantardjieva (1928: 64)
- **Type status:** Other material. **Location:** countryCode: BG; locality: Near Gramatikovo Vill.; **Record Level:** bibliographicCitation: Buresch & Kantardjieva (1928: 64)
- **Type status:** Other material. **Location:** countryCode: BG; locality: Near Bulgari Vill. (=Vurgari); **Record Level:** bibliographicCitation: Buresch & Kantardjieva (1928: 64)
- **Type status:** Other material. **Location:** countryCode: BG; locality: Ahtopol; **Record Level:** bibliographicCitation: Buresch & Kantardjieva (1928: 64)
- **Type status:** Other material. **Location:** countryCode: BG; locality: Ropotamo; **Record Level:** bibliographicCitation: Hieke & Wrase (1988: 12)
- **Type status:** Other material. **Occurrence:** recordedBy: K. Tuleshkov; individualCount: 2; **Location:** countryCode: BG; locality: Maslen nos Cape; **Event:** eventDate: 07-16-33
- **Type status:** Other material. **Occurrence:** recordedBy: V. D.; individualCount: 1; **Location:** countryCode: BG; locality: Strandzha; **Record Level:** institutionCode: NMP
- **Type status:** Other material. **Occurrence:** recordedBy: K. Iwanow; individualCount: 1; **Location:** countryCode: BG; locality: Maslen nos Cape; **Event:** eventDate: 23-28.05.1923; **Record Level:** institutionCode: NMNHS

#### Calosoma (Campalita) auropunctatum auropunctatum

(Herbst, 1784)

##### Materials

- **Type status:** Other material. **Occurrence:** recordedBy: Z. Hubenov; individualCount: "several"; **Location:** countryCode: BG; locality: Ahtopol; **Event:** eventDate: 18-25.08.1976; habitat: under seacost sand
- **Type status:** Other material. **Occurrence:** recordedBy: T. Shtirkov; individualCount: 1; **Location:** countryCode: BG; locality: "Oasis" Camping site; **Event:** eventDate: 21/07/1986; **Record Level:** institutionCode: NMNHS
- **Type status:** Other material. **Occurrence:** recordedBy: B. Petrov; **Location:** countryCode: BG; locality: Maslen nos Cape; **Event:** habitat: forest; **Record Level:** institutionCode: NMNHS

#### Carabus (Archicarabus) montivagus montivagus

Palliardi, 1825

##### Materials

- **Type status:** Other material. **Location:** countryCode: BG; locality: Yasna Polyana Vill.; **Record Level:** bibliographicCitation: Hieke & Wrase (1988: 18)
- **Type status:** Other material. **Location:** countryCode: BG; locality: Ropotamo; **Record Level:** bibliographicCitation: Hieke & Wrase (1988: 18)
- **Type status:** Other material. **Occurrence:** recordedBy: V. Popov; individualCount: 1; **Location:** countryCode: BG; locality: Kosti Vill.; **Event:** eventDate: 07-02-84
- **Type status:** Other material. **Occurrence:** recordedBy: B. Petrov; individualCount: 5; **Location:** countryCode: BG; locality: Ahtopol; **Event:** eventDate: 04-05-90
- **Type status:** Other material. **Occurrence:** recordedBy: B. Petrov; individualCount: 1; **Location:** countryCode: BG; locality: Mladezhko Vill.; verbatimElevation: 250; **Event:** eventDate: 04-26-90

#### Carabus (Archicarabus) wiedemanni wiedemanni

Ménéntriés, 1836

##### Materials

- **Type status:** Other material. **Occurrence:** recordedBy: R. Kostova; individualCount: 2; **Location:** countryCode: BG; locality: Near "Kachul" Place; verbatimElevation: 91; verbatimCoordinates: N42°01'57.1", E27°37'12.3"; geodeticDatum: WGS84; **Event:** eventDate: 04-17-09
- **Type status:** Other material. **Occurrence:** recordedBy: A. Gijonova; individualCount: 1; **Location:** countryCode: TR; locality: Sislioba Vill. surroundings; verbatimElevation: 52; verbatimCoordinates: N41°57'43.3", E27°54'35.2"; geodeticDatum: WGS84; **Event:** eventDate: 10-03-09
- **Type status:** Other material. **Occurrence:** recordedBy: R. Kostova; individualCount: 2; **Location:** countryCode: BG; locality: Slivarovo Vll. surroundings; verbatimElevation: 331; verbatimCoordinates: N41°58'11.9", E27°39'31.1"; geodeticDatum: WGS84; **Event:** eventDate: 09.06-02.08.2009; habitat: shrubs, mainly Calluna vulgaris
- **Type status:** Other material. **Occurrence:** recordedBy: R. Kostova; individualCount: 1; **Location:** countryCode: BG; locality: Slivarovo Vill., "Shafariitsa" Place; verbatimElevation: 224; verbatimCoordinates: N41°57'37.8", E27°39'34.8"; geodeticDatum: WGS84; **Event:** eventDate: 09.06-02.07.2009
- **Type status:** Other material. **Occurrence:** recordedBy: R. Kostova; individualCount: 5; **Location:** countryCode: BG; locality: Kosti Vill., "St. Ilia" Place; verbatimElevation: 35; verbatimCoordinates: N42°03'23.2", E27°45'51.6"; geodeticDatum: WGS84; **Event:** eventDate: 09.06-02.08.2009; habitat: meadow with single trees
- **Type status:** Other material. **Occurrence:** recordedBy: R. Kostova; individualCount: 1; **Location:** countryCode: BG; locality: Bulgari Vill., PA "Marina reka"; verbatimElevation: 183; verbatimCoordinates: N42°06'41.7", E27°45'53.0"; geodeticDatum: WGS84; **Event:** eventDate: 09.05-08.06.2009; habitat: oriental beech-oak forest with Rododendron ponticum
- **Type status:** Other material. **Occurrence:** recordedBy: R. Kostova; individualCount: 2; **Location:** countryCode: BG; locality: Brodilovo Vill., under Papiya peak; verbatimElevation: 296; verbatimCoordinates: N42°06'21.7", E27°50'32.6"; geodeticDatum: WGS84; **Event:** eventDate: 09.05-02.07.2009; habitat: shrubs, Phyllyrea latifolia
- **Type status:** Other material. **Occurrence:** recordedBy: R. Kostova; individualCount: 4; **Location:** countryCode: BG; locality: Sinemorets Vill., PA"Silistar", "Butamyata" Place; verbatimElevation: 17; verbatimCoordinates: N42°03'10.3", E27°59'13.4"; geodeticDatum: WGS84; **Event:** eventDate: 09.05-02.07.2009; habitat: oak forest
- **Type status:** Other material. **Occurrence:** recordedBy: R. Kostova; individualCount: 3; **Location:** countryCode: BG; locality: Sinemorets Vill., PA"Silistar", "Butamyata" Place; verbatimElevation: 10; verbatimCoordinates: N42°03'11.2", E27°59'19.7"; geodeticDatum: WGS84; **Event:** eventDate: 15.04-08.06.2009; habitat: meadow, single shrubs
- **Type status:** Other material. **Occurrence:** recordedBy: R. Kostova; individualCount: 4; **Location:** countryCode: BG; locality: Stoilovo Vill., "Petrova niva" Place; verbatimElevation: 234; verbatimCoordinates: N42°03'40.3", E27°31'42.3"; geodeticDatum: WGS84; **Event:** eventDate: 09.05-02.08.2009; habitat: meadow, single shrubs
- **Type status:** Other material. **Occurrence:** recordedBy: R. Kostova; individualCount: 2; **Location:** countryCode: BG; locality: Sinemorets Vill., PA"Silistar"; verbatimElevation: 23; verbatimCoordinates: N42°01'30.7", E28°00'25.4"; geodeticDatum: WGS84; **Event:** eventDate: 03.07-02.08.2009; habitat: oak forest
- **Type status:** Other material. **Occurrence:** recordedBy: R. Kostova; individualCount: 2; **Location:** countryCode: TR; locality: Igneada surroundings; verbatimElevation: 15; verbatimCoordinates: N41°51'51.0", E27°56'54.1"; geodeticDatum: WGS84; **Event:** eventDate: 06.07-29.09.2009; habitat: swamp forest
- **Type status:** Other material. **Occurrence:** recordedBy: R. Kostova; individualCount: 6; **Location:** countryCode: TR; locality: Kiyiköy surroundings; verbatimElevation: 38; verbatimCoordinates: N41°39'25.7", E28°05'11.2"; geodeticDatum: WGS84; **Event:** eventDate: 06.07 - 29.09.2009; habitat: oak with Arbutus unedo
- **Type status:** Other material. **Occurrence:** recordedBy: J. Ganev; individualCount: 1; **Location:** countryCode: BG; locality: Gramatikovo Vill.; **Event:** eventDate: 05/08/1983
- **Type status:** Other material. **Occurrence:** recordedBy: K. Taborsky & Prochazka; individualCount: 1; **Location:** countryCode: BG; locality: Primorsko; **Event:** eventDate: VII.1934; **Record Level:** institutionCode: NMP
- **Type status:** Other material. **Occurrence:** recordedBy: K. Taborsky & Prochazka; individualCount: 1; **Location:** countryCode: BG; locality: Primorsko (= Kyupriya); **Event:** eventDate: VII.1934; **Record Level:** institutionCode: NMP
- **Type status:** Other material. **Occurrence:** recordedBy: J. Mařan & K. Taborsky; individualCount: 5; **Location:** countryCode: BG; locality: Maslen nos Cape; **Event:** eventDate: VI.1933; **Record Level:** institutionCode: NMP
- **Type status:** Other material. **Location:** countryCode: BG; locality: Strandzha; **Record Level:** bibliographicCitation: Rambousek (1912: 66, as Carabus wiedemanni)
- **Type status:** Other material. **Location:** countryCode: BG; locality: Strandzha; **Record Level:** bibliographicCitation: Breuning (1932: 679, as C. (Archicarabus) w. m. burgassiensis)
- **Type status:** Other material. **Location:** countryCode: BG; locality: Rezovo Vill.; **Record Level:** bibliographicCitation: Kantardjieva (1934: 220, as Carabus wiedemanni)
- **Type status:** Other material. **Location:** countryCode: BG; locality: Primorsko; **Record Level:** bibliographicCitation: Hieke & Wrase (1988: 19)

#### Carabus (Carabus) granulatus granulatus

Linnaeus, 1758

##### Materials

- **Type status:** Other material. **Occurrence:** recordedBy: R. Kostova; individualCount: 120; **Location:** countryCode: BG; locality: Sinemorets Vill., PA "Estuary of Veleka river"; verbatimElevation: 6; verbatimCoordinates: N42°03'39.6", E27°57'55.2"; geodeticDatum: WGS84; **Event:** eventDate: 15.04-07.09.2009; habitat: swamp forest
- **Type status:** Other material. **Occurrence:** recordedBy: R. Kostova; individualCount: 4; **Location:** countryCode: TR; locality: Igneada surroundings; verbatimElevation: 15; verbatimCoordinates: N41°51'51.0", E27°56'54.1"; geodeticDatum: WGS84; **Event:** eventDate: 06.07-29.09.2009; habitat: swamp forest
- **Type status:** Other material. **Location:** countryCode: BG; locality: Tsarevo (= Micurin); **Record Level:** bibliographicCitation: Guéorguiev & Guéorguiev (1995: 43)
- **Type status:** Other material. **Location:** countryCode: BG; locality: Ahtopol; **Record Level:** bibliographicCitation: Guéorguiev & Guéorguiev (1995: 43)

#### Carabus (Chaetocarabus) intricatus intricatus

Linnaeus, 1761

##### Materials

- **Type status:** Other material. **Occurrence:** recordedBy: R. Kostova; individualCount: 2; **Location:** countryCode: BG; locality: Bliznak Vill., PA"Bataka"; verbatimElevation: 324; verbatimCoordinates: N42°11'37.3", E27°19'35.8"; geodeticDatum: WGS84; **Event:** eventDate: 15.04-08.06.2009; habitat: oak forest
- **Type status:** Other material. **Occurrence:** recordedBy: R. Kostova; individualCount: 4; **Location:** countryCode: BG; locality: Malko Tarnovo, "Propada" Place; verbatimElevation: 401; verbatimCoordinates: N41°58'49.6", E27°29'25.7"; geodeticDatum: WGS84; **Event:** eventDate: 15.04-02.07.2009; habitat: oriental beech forest
- **Type status:** Other material. **Occurrence:** recordedBy: R. Kostova; individualCount: 4; **Location:** countryCode: BG; locality: Mladezhko Vill., The springs of Mladezhka River; verbatimElevation: 231; verbatimCoordinates: N42°09'04.5", E27°21'26.1"; geodeticDatum: WGS84; **Event:** eventDate: 09.05-02.07.2009; habitat: black alder and hornbeam forest
- **Type status:** Other material. **Location:** countryCode: BG; locality: Strandzha; **Record Level:** bibliographicCitation: Guéorguiev & Guéorguiev (1995: 49)
- **Type status:** Other material. **Occurrence:** recordedBy: K. Tuleshkov; individualCount: 1; **Location:** countryCode: BG; locality: Maslen nos Cape; **Record Level:** institutionCode: NMNHS

#### Carabus (Eucarabus) ullrichi rhilensis

Kraatz, 1876

##### Materials

- **Type status:** Other material. **Location:** countryCode: BG; locality: Primorsko; **Record Level:** bibliographicCitation: Hieke & Wrase (1988: 17)

#### Carabus (Heterocarabus) mariettii mariettii

Cristofori & Jan, 1837

##### Materials

- **Type status:** Other material. **Occurrence:** recordedBy: R. Kostova; individualCount: 1; **Location:** countryCode: TR; locality: Sergen Vill. surroundings; verbatimElevation: 818; verbatimCoordinates: N41°44'37.0", E27°42'24.8"; geodeticDatum: WGS84; **Event:** eventDate: 05-25-11
- **Type status:** Other material. **Occurrence:** recordedBy: R. Kostova; individualCount: 3; **Location:** countryCode: BG; locality: Sinemorets Vill., PA"Silistar", "Butamyata" Place; verbatimElevation: 17; verbatimCoordinates: N42°03'10.3", E27°59'13.4"; geodeticDatum: WGS84; **Event:** eventDate: 15.04-08.05.2009; habitat: oak forest
- **Type status:** Other material. **Occurrence:** recordedBy: R. Kostova; individualCount: 23; **Location:** countryCode: BG; locality: Malko Tarnovo, "Propada" Place; verbatimElevation: 401; verbatimCoordinates: N41°58'49.6", E27°29'25.7"; geodeticDatum: WGS84; **Event:** eventDate: 15.04-02.07.2009; habitat: oriental beech forest
- **Type status:** Other material. **Occurrence:** recordedBy: R. Kostova; individualCount: 1; **Location:** countryCode: BG; locality: Slivarovo Vill., "Shafariitsa" Place; verbatimElevation: 224; verbatimCoordinates: N41°57'37.8", E27°39'34.8"; geodeticDatum: WGS84; **Event:** eventDate: 15.04-08.05.2009
- **Type status:** Other material. **Occurrence:** recordedBy: R. Kostova; individualCount: 7; **Location:** countryCode: BG; locality: Kondolovo Vill., "Byalata prast" Place; verbatimElevation: 271; verbatimCoordinates: N42°05'47.7", E27°39'55.0"; geodeticDatum: WGS84; **Event:** eventDate: 09.05-08.06.2009; habitat: oak-beech mixed forest
- **Type status:** Other material. **Occurrence:** recordedBy: R. Kostova; individualCount: 1; **Location:** countryCode: BG; locality: Kondolovo Vill., "Byalata prast" Place; verbatimElevation: 283; verbatimCoordinates: N42°05'49.7", E27°39'56.3"; geodeticDatum: WGS84; **Event:** eventDate: 09.05-08.06.2009; habitat: beech forest with Vaccinium arctostaphylos
- **Type status:** Other material. **Occurrence:** recordedBy: R. Kostova; individualCount: 69; **Location:** countryCode: BG; locality: Bulgari Vill., PA "Marina reka"; verbatimElevation: 183; verbatimCoordinates: N42°06'41.7", E27°45'53.0"; geodeticDatum: WGS84; **Event:** eventDate: 15.04-02.07.2009; habitat: oriental beech-oak forest with Rododendron ponticum
- **Type status:** Other material. **Occurrence:** recordedBy: R. Kostova; individualCount: 1; **Location:** countryCode: BG; locality: Brodilovo Vill., under Papiya peak; verbatimElevation: 336; verbatimCoordinates: N42°06'23.7", E27°50'36.1"; geodeticDatum: WGS84; **Event:** eventDate: 15.04-08.05.2009; habitat: oak forest
- **Type status:** Other material. **Occurrence:** recordedBy: R. Kostova; individualCount: 1; **Location:** countryCode: BG; locality: Brodilovo Vill., under Papiya peak; verbatimElevation: 336; verbatimCoordinates: N42°06'23.7", E27°50'36.1"; geodeticDatum: WGS84; **Event:** eventDate: 09.06-02.07.2009; habitat: oak forest
- **Type status:** Other material. **Occurrence:** recordedBy: R. Kostova; individualCount: 2; **Location:** countryCode: BG; locality: Mladezhko Vill., The springs of Mladezhka River; verbatimElevation: 231; verbatimCoordinates: N42°09'04.5", E27°21'26.1"; geodeticDatum: WGS84; **Event:** eventDate: 09.06-02.07.2009; habitat: black alder and hornbeam forest
- **Type status:** Other material. **Location:** countryCode: BG; locality: Primorsko; **Record Level:** bibliographicCitation: Hieke & Wrase (1988: 16)
- **Type status:** Other material. **Location:** countryCode: BG; locality: Ropotamo; **Record Level:** bibliographicCitation: Hieke & Wrase (1988: 16)
- **Type status:** Other material. **Location:** countryCode: BG; locality: Kosti Vill., 1 km of the Bulgaria - Turkey border; **Record Level:** bibliographicCitation: Guéorguiev & Guéorguiev (1995: 49)
- **Type status:** Other material. **Occurrence:** recordedBy: B. Guéorguiev; individualCount: 1; **Location:** countryCode: BG; locality: Aydere River; verbatimElevation: 350-450; **Event:** eventDate: 26/05/1995; **Record Level:** institutionCode: NMNHS

#### Carabus (Megodontus) violaceus azurescens

Dejean, 1826

##### Materials

- **Type status:** Other material. **Location:** countryCode: BG; locality: Strandzha; **Record Level:** bibliographicCitation: Guéorguiev & Guéorguiev (1995: 50)
- **Type status:** Other material. **Location:** countryCode: BG; locality: Maslen nos Cape; **Record Level:** bibliographicCitation: Guéorguiev & Guéorguiev (1995: 50)
- **Type status:** Other material. **Location:** countryCode: BG; locality: Maslen nos Cape; **Record Level:** bibliographicCitation: Guéorguiev & Muilwijk (2001: 113)

##### Notes

This record is probably based on mislabelled specimens (see also Guéorguiev & Muilwijk, 2000: 113)

#### Carabus (Pachystus) graecus morio

Mannerheim, 1830

##### Materials

- **Type status:** Other material. **Occurrence:** recordedBy: P. Mitov; individualCount: 1; **Location:** countryCode: TR; locality: Pinarhisar surroundings; verbatimElevation: 204; verbatimCoordinates: N41°37'43.1", E27°30'52.6"; geodeticDatum: WGS84; **Event:** eventDate: 22/05/2010

#### Carabus (Procerus) scabrosus scabrosus

Olivier, 1790

##### Materials

- **Type status:** Other material. **Occurrence:** recordedBy: R. Kostova; individualCount: 1; **Location:** countryCode: BG; locality: Malko Tarnovo, "Indipasqua" Place; verbatimElevation: 211; verbatimCoordinates: N42°00'16.9", E27°39'09.2"; geodeticDatum: WGS84; **Event:** eventDate: 17/04/2009
- **Type status:** Other material. **Occurrence:** recordedBy: R. Kostova; individualCount: 1; **Location:** countryCode: BG; locality: Stoilovo Vill., "Sredoka" Reserve, along Mechi dol River; verbatimElevation: 206; verbatimCoordinates: N42°01'51.1", E27°30'50.1"; geodeticDatum: WGS84; **Event:** eventDate: 19/04/2009
- **Type status:** Other material. **Occurrence:** recordedBy: R. Kostova; individualCount: 1; **Location:** countryCode: BG; locality: Bulgari Vill., PA "Marina reka"; verbatimElevation: 172; verbatimCoordinates: N42°06'41.7", E27°45'53.0"; geodeticDatum: WGS84; **Event:** eventDate: 11/06/2009
- **Type status:** Other material. **Occurrence:** recordedBy: R. Kostova; individualCount: 6; **Location:** countryCode: BG; locality: Malko Tarnovo, "Propada" Place; verbatimElevation: 401; verbatimCoordinates: N41°58'49.6", E27°29'25.7"; geodeticDatum: WGS84; **Event:** eventDate: 09.05-08.06.2009; habitat: oriental beech forest
- **Type status:** Other material. **Occurrence:** recordedBy: R. Kostova; individualCount: 1; **Location:** countryCode: TR; locality: Igneada surroundings; verbatimElevation: 15; verbatimCoordinates: N41°51'51.0", E27°56'54.1"; geodeticDatum: WGS84; **Event:** eventDate: 06.07-29.09.2009; habitat: swamp forest
- **Type status:** Other material. **Location:** countryCode: BG; locality: Malko Tarnovo; **Record Level:** bibliographicCitation: Buresch & Kantardjieva (1928: 66)
- **Type status:** Other material. **Location:** countryCode: BG; locality: Kosti Vill.; **Record Level:** bibliographicCitation: Buresch & Kantardjieva (1928: 66)
- **Type status:** Other material. **Occurrence:** recordedBy: M. Josifov; individualCount: 1; **Location:** countryCode: BG; locality: Primorsko, Ropotamo Reserve; **Event:** eventDate: 03/06/1959
- **Type status:** Other material. **Occurrence:** recordedBy: V. Popov; individualCount: 1; **Location:** countryCode: BG; locality: Sinemorets Vill., near Veleka River; **Event:** eventDate: 02/07/1984; habitat: hornbeam forest
- **Type status:** Other material. **Occurrence:** recordedBy: B. Petrov; individualCount: 1; **Location:** countryCode: BG; locality: "Kachula" Place; **Event:** eventDate: 27/04/1990
- **Type status:** Other material. **Occurrence:** recordedBy: B. Petrov; individualCount: 1; **Location:** countryCode: BG; locality: Mladezhko Vill.; **Event:** eventDate: 28/04/1990
- **Type status:** Other material. **Occurrence:** recordedBy: B. Petrov; individualCount: 2; **Location:** countryCode: BG; locality: Mladezhko Vill.; **Event:** eventDate: 01/05/1991
- **Type status:** Other material. **Occurrence:** recordedBy: B. Petrov; individualCount: 1; **Location:** countryCode: BG; locality: Trakijski stan Place; **Event:** eventDate: 04/05/1991
- **Type status:** Other material. **Occurrence:** recordedBy: B. Petrov; individualCount: 1; **Location:** countryCode: BG; locality: Kosti Vill.; **Event:** eventDate: 28/04/1990
- **Type status:** Other material. **Occurrence:** recordedBy: S. Beshkov; individualCount: 1; **Location:** countryCode: BG; locality: 3 km East of Kondolovo Vill.

#### Carabus (Procrustes) coriaceus kindermanni

Waltl, 1838

##### Materials

- **Type status:** Other material. **Occurrence:** recordedBy: R. Kostova; individualCount: 1; **Location:** countryCode: BG; locality: Mladezhko Vill., along Mladezhka River; verbatimElevation: 248; verbatimCoordinates: N42°09'00.9", E27°21'44.2"; geodeticDatum: WGS84; **Event:** eventDate: 16/04/2009
- **Type status:** Other material. **Occurrence:** recordedBy: R. Kostova; individualCount: 1; **Location:** countryCode: BG; locality: Bulgari Vill., PA "Marina reka"; verbatimElevation: 183; verbatimCoordinates: N42°06'41.7", E27°45'53.0"; geodeticDatum: WGS84; **Event:** eventDate: 07/05/2009
- **Type status:** Other material. **Occurrence:** recordedBy: R. Kostova; individualCount: 1; **Location:** countryCode: TR; locality: Sarpdere Vill., Dupnisa Cave surroundings; verbatimElevation: 356; verbatimCoordinates: N41°50'26.0", E27°33'22.4"; geodeticDatum: WGS84; **Event:** eventDate: 23/05/2010
- **Type status:** Other material. **Occurrence:** recordedBy: R. Kostova; individualCount: 1; **Location:** countryCode: BG; locality: Malko Tarnovo, the Tracian tumbs; verbatimElevation: 383; verbatimCoordinates: N41°58'54.6", E27°29'27.8"; geodeticDatum: WGS84; **Event:** eventDate: 27/05/2010
- **Type status:** Other material. **Occurrence:** recordedBy: R. Kostova; individualCount: 1; **Location:** countryCode: TR; locality: Sivriler Vill. suroundings; verbatimElevation: 379; verbatimCoordinates: N41°47'30.2", E27°50'00.9"; geodeticDatum: WGS84; **Event:** eventDate: 24/05/2011
- **Type status:** Other material. **Occurrence:** recordedBy: R. Kostova; individualCount: 1; **Location:** countryCode: TR; locality: Sarpdere Vill., Dupnisa Cave surroundings; verbatimElevation: 440; verbatimCoordinates: N41°49'56.3", E27°33'24.0"; geodeticDatum: WGS84; **Event:** eventDate: 26/05/2011; habitat: meadow
- **Type status:** Other material. **Occurrence:** recordedBy: R. Kostova; individualCount: 9; **Location:** countryCode: BG; locality: Bliznak Vill., PA"Bataka"; verbatimElevation: 324; verbatimCoordinates: N42°11'37.3", E27°19'35.8"; geodeticDatum: WGS84; **Event:** eventDate: 09.05-07.09.2009; habitat: oak forest
- **Type status:** Other material. **Occurrence:** recordedBy: R. Kostova; individualCount: 47; **Location:** countryCode: BG; locality: Malko Tarnovo, "Propada" Place; verbatimElevation: 401; verbatimCoordinates: N41°58'49.6", E27°29'25.7"; geodeticDatum: WGS84; **Event:** eventDate: 15.04-02.08.2009; habitat: oriental beech forest
- **Type status:** Other material. **Occurrence:** recordedBy: R. Kostova; individualCount: 13; **Location:** countryCode: BG; locality: Slivarovo Vll. surroundings; verbatimElevation: 331; verbatimCoordinates: N41°58'11.9", E27°39'31.1"; geodeticDatum: WGS84; **Event:** eventDate: 15.04-02.08.2009; habitat: shrubs, mainly Calluna vulgaris
- **Type status:** Other material. **Occurrence:** recordedBy: R. Kostova; individualCount: 59; **Location:** countryCode: BG; locality: Kondolovo Vill., "Byalata prast" Place; verbatimElevation: 271; verbatimCoordinates: N42°05'47.7", E27°39'55.0"; geodeticDatum: WGS84; **Event:** eventDate: 09.05-07.09.2009; habitat: oak-beech mixed forest
- **Type status:** Other material. **Occurrence:** recordedBy: R. Kostova; individualCount: 17; **Location:** countryCode: BG; locality: Kondolovo Vill., "Byalata prast" Place; verbatimElevation: 283; verbatimCoordinates: N42°05'49.7", E27°39'56.3"; geodeticDatum: WGS84; **Event:** eventDate: 15.04-02.08.2009; habitat: beech forest with Vaccinium arctostaphylos
- **Type status:** Other material. **Occurrence:** recordedBy: R. Kostova; individualCount: 3; **Location:** countryCode: BG; locality: Kosti Vill., "St. Ilia" Place; verbatimElevation: 35; verbatimCoordinates: N42°03'23.2", E27°45'51.6"; geodeticDatum: WGS84; **Event:** eventDate: 09.05-07.09.2009; habitat: meadow with single trees
- **Type status:** Other material. **Occurrence:** recordedBy: R. Kostova; individualCount: 20; **Location:** countryCode: BG; locality: Bulgari Vill., PA "Marina reka"; verbatimElevation: 183; verbatimCoordinates: N42°06'41.7", E27°45'53.0"; geodeticDatum: WGS84; **Event:** eventDate: 09.05-02.08.2009; habitat: oriental beech-oak forest with Rododendron ponticum
- **Type status:** Other material. **Occurrence:** recordedBy: R. Kostova; individualCount: 19; **Location:** countryCode: BG; locality: Brodilovo Vill., under Papiya peak; verbatimElevation: 336; verbatimCoordinates: N42°06'23.7", E27°50'36.1"; geodeticDatum: WGS84; **Event:** eventDate: 09.05-02.07.2009; habitat: oak forest
- **Type status:** Other material. **Occurrence:** recordedBy: R. Kostova; individualCount: 3; **Location:** countryCode: BG; locality: Brodilovo Vill., under Papiya peak; verbatimElevation: 296; verbatimCoordinates: N42°06'21.7", E27°50'32.6"; geodeticDatum: WGS84; **Event:** eventDate: 09.05-02.07.2009; habitat: shrubs, Phyllyrea latifolia
- **Type status:** Other material. **Occurrence:** recordedBy: R. Kostova; individualCount: 4; **Location:** countryCode: BG; locality: Sinemorets Vill., PA "Estuary of Veleka river"; verbatimElevation: 6; verbatimCoordinates: N42°03'39.6", E27°57'55.2"; geodeticDatum: WGS84; **Event:** eventDate: 09.05-02.07.2009
- **Type status:** Other material. **Occurrence:** recordedBy: R. Kostova; individualCount: 32; **Location:** countryCode: BG; locality: Sinemorets Vill., PA"Silistar", "Butamyata" Place; verbatimElevation: 17; verbatimCoordinates: N42°03'10.3", E27°59'13.4"; geodeticDatum: WGS84; **Event:** eventDate: 09.05-02.07.2009; habitat: oak forest
- **Type status:** Other material. **Occurrence:** recordedBy: R. Kostova; individualCount: 17; **Location:** countryCode: BG; locality: Sinemorets Vill., PA"Silistar", "Butamyata" Place; verbatimElevation: 10; verbatimCoordinates: N42°03'11.2", E27°59'19.7"; geodeticDatum: WGS84; **Event:** eventDate: 09.05-02.07.2009; habitat: meadow, single shrubs
- **Type status:** Other material. **Occurrence:** recordedBy: R. Kostova; individualCount: 14; **Location:** countryCode: BG; locality: Mladezhko Vill., The springs of Mladezhka River; verbatimElevation: 231; verbatimCoordinates: N42°09'04.5", E27°21'26.1"; geodeticDatum: WGS84; **Event:** eventDate: 09.05-07.09.2009; habitat: black alder and hornbeam forest
- **Type status:** Other material. **Occurrence:** recordedBy: R. Kostova; individualCount: 3; **Location:** countryCode: BG; locality: Stoilovo Vill., "Petrova niva" Place; verbatimElevation: 234; verbatimCoordinates: N42°03'40.3", E27°31'42.3"; geodeticDatum: WGS84; **Event:** eventDate: 09.06-02.07.2009; habitat: meadow, single shrubs
- **Type status:** Other material. **Occurrence:** recordedBy: R. Kostova; individualCount: 13; **Location:** countryCode: BG; locality: Sinemorets Vill., PA"Silistar"; verbatimElevation: 23; verbatimCoordinates: N42°01'30.7", E28°00'25.4"; geodeticDatum: WGS84; **Event:** eventDate: 09.05-08.06.2009; habitat: oak forest
- **Type status:** Other material. **Occurrence:** recordedBy: R. Kostova; individualCount: 3; **Location:** countryCode: TR; locality: Igneada surroundings; verbatimElevation: 15; verbatimCoordinates: N41°51'51.0", E27°56'54.1"; geodeticDatum: WGS84; **Event:** eventDate: 06.07-29.09.2009; habitat: swamp forest
- **Type status:** Other material. **Occurrence:** recordedBy: R. Kostova; individualCount: 3; **Location:** countryCode: TR; locality: Gokyaka Vill., Road to Dupnisa Cave; verbatimElevation: 337; verbatimCoordinates: N41°52'20.0", E27°37'06.4"; geodeticDatum: WGS84; **Event:** eventDate: 06.07 - 29.09.2009; habitat: oak-hornbeam mixed forest
- **Type status:** Other material. **Location:** countryCode: BG; locality: Malko Tarnovo; **Record Level:** bibliographicCitation: Buresch & Kantardjieva (1928: 73)
- **Type status:** Other material. **Location:** countryCode: BG; locality: Strandzha; **Record Level:** bibliographicCitation: Breuning (1928: 110)
- **Type status:** Other material. **Location:** countryCode: BG; locality: Strandzha; **Record Level:** bibliographicCitation: Mařan (1949: 8)
- **Type status:** Other material. **Location:** countryCode: BG; locality: Maslen nos Cape; **Record Level:** bibliographicCitation: Mařan (1949: 8)
- **Type status:** Other material. **Location:** countryCode: BG; locality: Kiten; **Record Level:** bibliographicCitation: Hieke & Wrase (1988: 14)
- **Type status:** Other material. **Location:** countryCode: BG; locality: Tsarevo (= Micurin); **Record Level:** bibliographicCitation: Hieke & Wrase (1988: 14)
- **Type status:** Other material. **Location:** countryCode: BG; locality: Primorsko; **Record Level:** bibliographicCitation: Hieke & Wrase (1988: 14)
- **Type status:** Other material. **Location:** countryCode: BG; locality: Ropotamo; **Record Level:** bibliographicCitation: Hieke & Wrase (1988: 14)
- **Type status:** Other material. **Occurrence:** recordedBy: P. Drenski; individualCount: 1; **Location:** countryCode: BG; locality: Rezovo Vill.; **Event:** eventDate: 15/05/1931
- **Type status:** Other material. **Occurrence:** recordedBy: P. Drenski; individualCount: 4; **Location:** countryCode: BG; locality: Brodilovo Vill., Papiya Peak; **Event:** eventDate: 10/06/1933
- **Type status:** Other material. **Occurrence:** recordedBy: K. Tuleshkov; individualCount: 3; **Location:** countryCode: BG; locality: Maslen nos Cape; **Event:** eventDate: 16/07/1933
- **Type status:** Other material. **Occurrence:** individualCount: 1; **Location:** countryCode: BG; locality: Kondros; **Event:** eventDate: 01/05/1940
- **Type status:** Other material. **Occurrence:** recordedBy: Vl. Lazarov; individualCount: 1; **Location:** countryCode: BG; locality: Primorsko; **Event:** eventDate: VI.1957
- **Type status:** Other material. **Occurrence:** recordedBy: S. Bocharov; individualCount: 3; **Location:** countryCode: BG; locality: Primorsko; **Event:** eventDate: 05-06.10.1977
- **Type status:** Other material. **Occurrence:** recordedBy: T. Shtirkov; individualCount: 1; **Location:** countryCode: BG; locality: "Oasis" Camping site; **Event:** eventDate: 16/08/1984
- **Type status:** Other material. **Occurrence:** recordedBy: T. Shtirkov; individualCount: 1; **Location:** countryCode: BG; locality: Tsarevo (= Micurin); **Event:** eventDate: 01-05.09.1978
- **Type status:** Other material. **Occurrence:** recordedBy: T. Shtirkov; individualCount: 2; **Location:** countryCode: BG; locality: Tsarevo (= Micurin); **Event:** eventDate: 10-15.09.1979
- **Type status:** Other material. **Occurrence:** recordedBy: T. Shtirkov; individualCount: 6; **Location:** countryCode: BG; locality: Tsarevo (= Micurin); **Event:** eventDate: 21-25.09.1979
- **Type status:** Other material. **Occurrence:** recordedBy: B. Petrov; individualCount: 2; **Location:** countryCode: BG; locality: Brodilovo Vill.; **Event:** eventDate: 08/04/1990; habitat: dense forest
- **Type status:** Other material. **Occurrence:** recordedBy: B. Petrov; individualCount: 2; **Location:** countryCode: BG; locality: Mladezhko Vill.; verbatimElevation: 250; **Event:** eventDate: 01/05/1990; habitat: forest
- **Type status:** Other material. **Occurrence:** recordedBy: B. Petrov; individualCount: 2; **Location:** countryCode: BG; locality: Maslen nos Cape; **Event:** eventDate: 07/05/1991; habitat: forest
- **Type status:** Other material. **Occurrence:** recordedBy: M. Josifov; individualCount: 1; **Location:** countryCode: BG; locality: Gramatikovo Vill.; **Event:** eventDate: 28/05/1973; **Record Level:** institutionCode: NMNHS
- **Type status:** Other material. **Occurrence:** recordedBy: B. Guéorguiev; individualCount: 3; **Location:** countryCode: BG; locality: Sinemorets Village, south bay; **Event:** eventDate: 27/05/1995; **Record Level:** institutionCode: NMNHS
- **Type status:** Other material. **Occurrence:** recordedBy: B. Guéorguiev; individualCount: 1; **Location:** countryCode: BG; locality: Nature Park Ropotamo; **Event:** eventDate: 29/05/1995; **Record Level:** institutionCode: NMNHS

#### Carabus (Tachypus) cancellatus intermedius

Dejean, 1826

##### Materials

- **Type status:** Other material. **Occurrence:** recordedBy: Z. Hubenov; individualCount: 1; **Location:** countryCode: BG; locality: Ahtopol; **Event:** eventDate: 27/07/1970

#### Carabus (Tomocarabus) convexus gracilior

Géhin, 1885

##### Materials

- **Type status:** Other material. **Occurrence:** recordedBy: R. Kostova; individualCount: 20; **Location:** countryCode: BG; locality: Bliznak Vill., PA"Bataka"; verbatimElevation: 324; verbatimCoordinates: N42°11'37.3", E27°19'35.8"; geodeticDatum: WGS84; **Event:** eventDate: 15.04-07.09.2009; habitat: oak forest
- **Type status:** Other material. **Occurrence:** recordedBy: R. Kostova; individualCount: 79; **Location:** countryCode: BG; locality: Malko Tarnovo, "Propada" Place; verbatimElevation: 401; verbatimCoordinates: N41°58'49.6", E27°29'25.7"; geodeticDatum: WGS84; **Event:** eventDate: 15.04-07.09.2009; habitat: oriental beech forest
- **Type status:** Other material. **Occurrence:** recordedBy: R. Kostova; individualCount: 7; **Location:** countryCode: BG; locality: Slivarovo Vill., "Shafariitsa" Place; verbatimElevation: 224; verbatimCoordinates: N41°57'37.8", E27°39'34.8"; geodeticDatum: WGS84; **Event:** eventDate: 15.04-07.09.2009; habitat: black alder-oak forest
- **Type status:** Other material. **Occurrence:** recordedBy: R. Kostova; individualCount: 2; **Location:** countryCode: BG; locality: Kondolovo Vill., "Byalata prast" Place; verbatimElevation: 271; verbatimCoordinates: N42°05'47.7", E27°39'55.0"; geodeticDatum: WGS84; **Event:** eventDate: 03.07-02.08.2009; habitat: oak-beech mixed forest
- **Type status:** Other material. **Occurrence:** recordedBy: R. Kostova; individualCount: 1; **Location:** countryCode: BG; locality: Kondolovo Vill., "Byalata prast" Place; verbatimElevation: 283; verbatimCoordinates: N42°05'49.7", E27°39'56.3"; geodeticDatum: WGS84; **Event:** eventDate: 03.07-02.08.2009; habitat: beech forest with Vaccinium arctostaphylos
- **Type status:** Other material. **Occurrence:** recordedBy: R. Kostova; individualCount: 2; **Location:** countryCode: BG; locality: Kosti Vill., "St. Ilia" Place; verbatimElevation: 35; verbatimCoordinates: N42°03'23.2", E27°45'51.6"; geodeticDatum: WGS84; **Event:** eventDate: 09.06-02.08.2009; habitat: meadow with single trees
- **Type status:** Other material. **Occurrence:** recordedBy: R. Kostova; individualCount: 2; **Location:** countryCode: BG; locality: Bulgari Vill., PA "Marina reka"; verbatimElevation: 183; verbatimCoordinates: N42°06'41.7", E27°45'53.0"; geodeticDatum: WGS84; **Event:** eventDate: 03.07-02.08.2009; habitat: oriental beech-oak forest with Rododendron ponticum
- **Type status:** Other material. **Occurrence:** recordedBy: R. Kostova; individualCount: 2; **Location:** countryCode: BG; locality: Brodilovo Vill., under Papiya peak; verbatimElevation: 336; verbatimCoordinates: N42°06'23.7", E27°50'36.1"; geodeticDatum: WGS84; **Event:** eventDate: 15.04-08.05.2009; habitat: oak forest
- **Type status:** Other material. **Occurrence:** recordedBy: R. Kostova; individualCount: 1; **Location:** countryCode: BG; locality: Brodilovo Vill., under Papiya peak; verbatimElevation: 336; verbatimCoordinates: N42°06'23.7", E27°50'36.1"; geodeticDatum: WGS84; **Event:** eventDate: 03.07-02.08.2009; habitat: oak forest
- **Type status:** Other material. **Occurrence:** recordedBy: R. Kostova; individualCount: 1; **Location:** countryCode: BG; locality: Brodilovo Vill., under Papiya peak; verbatimElevation: 296; verbatimCoordinates: N42°06'21.7", E27°50'32.6"; geodeticDatum: WGS84; **Event:** eventDate: 15.04-08.05.2009; habitat: shrubs, Phyllyrea latifolia
- **Type status:** Other material. **Occurrence:** recordedBy: R. Kostova; individualCount: 3; **Location:** countryCode: BG; locality: Sinemorets Vill., PA"Silistar", "Butamyata" Place; verbatimElevation: 17; verbatimCoordinates: N42°03'10.3", E27°59'13.4"; geodeticDatum: WGS84; **Event:** eventDate: 15.04-08.06.2009; habitat: oak forest
- **Type status:** Other material. **Occurrence:** recordedBy: R. Kostova; individualCount: 14; **Location:** countryCode: BG; locality: Mladezhko Vill., The springs of Mladezhka River; verbatimElevation: 231; verbatimCoordinates: N42°09'04.5", E27°21'26.1"; geodeticDatum: WGS84; **Event:** eventDate: 09.05-02.08.2009; habitat: black alder and hornbeam forest
- **Type status:** Other material. **Occurrence:** recordedBy: R. Kostova; individualCount: 29; **Location:** countryCode: TR; locality: Igneada surroundings; verbatimElevation: 15; verbatimCoordinates: N41°51'51.0", E27°56'54.1"; geodeticDatum: WGS84; **Event:** eventDate: 06.07-29.09.2009; habitat: swamp forest
- **Type status:** Other material. **Occurrence:** recordedBy: R. Kostova; individualCount: 1; **Location:** countryCode: TR; locality: Gokyaka Vill., Road to Dupnisa Cave; verbatimElevation: 337; verbatimCoordinates: N41°52'20.0", E27°37'06.4"; geodeticDatum: WGS84; **Event:** eventDate: 06.07 - 29.09.2009; habitat: oak-hornbeam mixed forest
- **Type status:** Other material. **Occurrence:** recordedBy: R. Radev; individualCount: 1; **Location:** countryCode: BG; locality: Strandzha; **Event:** eventDate: 6.VIII.1989; **Record Level:** collectionID: R. Radev coll.
- **Type status:** Other material. **Occurrence:** recordedBy: K. Tuleshkov; individualCount: 1; **Location:** countryCode: BG; locality: Maslen nos Cape; **Event:** eventDate: 16/07/1933
- **Type status:** Other material. **Occurrence:** recordedBy: K. Tuleshkov; individualCount: 1; **Location:** countryCode: BG; locality: Maslen nos Cape; **Event:** eventDate: 16/08/1933
- **Type status:** Other material. **Occurrence:** recordedBy: S. Zagorchinov; individualCount: 1; **Location:** countryCode: BG; locality: Strandzha; **Event:** eventDate: 18/06/1973
- **Type status:** Other material. **Location:** countryCode: BG; locality: Strandzha; **Record Level:** bibliographicCitation: Breuning (1928: 112)
- **Type status:** Other material. **Location:** countryCode: BG; locality: Tsarevo (= Micurin); **Record Level:** bibliographicCitation: Hieke & Wrase (1988: 19)
- **Type status:** Other material. **Occurrence:** recordedBy: B. Guéorguiev; individualCount: 1; **Location:** countryCode: BG; locality: Veleka River near Mladezko Village; **Event:** eventDate: 25/05/1995; **Record Level:** institutionCode: NMNHS
- **Type status:** Other material. **Occurrence:** recordedBy: B. Guéorguiev; individualCount: 1; **Location:** countryCode: BG; locality: Aydere River; verbatimElevation: 350-450; **Event:** eventDate: 26/05/1995; **Record Level:** institutionCode: NMNHS

#### Carabus (Trachycarabus) scabriusculus bulgarus

Lapouge, 1908

##### Materials

- **Type status:** Other material. **Occurrence:** recordedBy: S. Zagorchinov; individualCount: 2; **Location:** countryCode: BG; locality: Strandzha; **Event:** eventDate: 18/06/1973
- **Type status:** Other material. **Occurrence:** recordedBy: T. Shtirkov; individualCount: 1; **Location:** countryCode: BG; locality: "Oasis" Camping site; **Event:** eventDate: 20/07/1982
- **Type status:** Other material. **Location:** countryCode: BG; locality: Brodilovo Vill., Papiya Peak; verbatimElevation: 500; **Record Level:** bibliographicCitation: Guéorguiev & Guéorguiev (1995: 42)
- **Type status:** Other material. **Location:** countryCode: BG; locality: Brodilovo Vill., Papiya Peak; **Record Level:** bibliographicCitation: Guéorguiev & Guéorguiev (1995: 42)
- **Type status:** Other material. **Location:** countryCode: BG; locality: Isperets Peak; **Record Level:** bibliographicCitation: Guéorguiev & Guéorguiev (1995: 42)

#### Omophron (Omophron) limbatum

(Fabricius, 1777)

##### Materials

- **Type status:** Other material. **Occurrence:** recordedBy: R. Kostova; individualCount: 1; **Location:** countryCode: BG; locality: Stoilovo Vill., "Sredoka" Reserve, along Mechi dol River; verbatimElevation: 206; verbatimCoordinates: N42°01'51.1", E27°30'50.1"; geodeticDatum: WGS84; **Event:** eventDate: 19/04/2009
- **Type status:** Other material. **Occurrence:** recordedBy: R. Kostova; individualCount: 1; **Location:** countryCode: BG; locality: Primorsko, Perla Beach; verbatimElevation: 1; verbatimCoordinates: N42°17'10.2", E27°45'13.6"; geodeticDatum: WGS84; **Event:** eventDate: 10/05/2009
- **Type status:** Other material. **Location:** countryCode: BG; locality: Yasna Polyana Vill.; **Record Level:** bibliographicCitation: Hieke & Wrase (1988: 23)

#### Paussus turcicus

I. Frivaldszky von Frivald, 1835

##### Materials

- **Type status:** Other material. **Location:** countryCode: TR; locality: Kiyiköy surroundings; verbatimElevation: 38; verbatimCoordinates: N41°39'25.7", E28°05'11.2"; geodeticDatum: WGS84; **Record Level:** bibliographicCitation: Lapeva-Gjonova et al. (2011)
- **Type status:** Other material. **Occurrence:** recordedBy: R. Kostova; individualCount: 1; **Location:** countryCode: TR; locality: Kiyiköy surroundings; verbatimElevation: 38; verbatimCoordinates: N41°39'25.7", E28°05'11.2"; geodeticDatum: WGS84; **Event:** eventDate: 22/05/2011
- **Type status:** Other material. **Occurrence:** recordedBy: R. Kostova; individualCount: 1; **Location:** countryCode: TR; locality: Sarpdere Vill., Dupnisa Cave surroundings; verbatimElevation: 440; verbatimCoordinates: N41°49'56.3", E27°33'24.0"; geodeticDatum: WGS84; **Event:** eventDate: 26/05/2011; habitat: meadow
- **Type status:** Other material. **Occurrence:** individualCount: 52; **Location:** countryCode: BG; locality: Strandzha; **Event:** eventDate: no date; **Record Level:** institutionCode: NPM
- **Type status:** Other material. **Location:** countryCode: BG; locality: Strandzha; **Record Level:** bibliographicCitation: Guéorguiev (1992: 61)
- **Type status:** Other material. **Location:** countryCode: BG; locality: Maslen nos Cape; **Record Level:** bibliographicCitation: Guéorguiev (1992: 61)
- **Type status:** Other material. **Location:** countryCode: BG; locality: Maslen nos Cape; **Record Level:** bibliographicCitation: Guéorguiev & Guéorguiev (1995: 34)

#### Aptinus (Aptinus) cordicollis

Chaudoir, 1843

##### Materials

- **Type status:** Other material. **Occurrence:** recordedBy: R. Bekchiev; individualCount: 1; **Location:** countryCode: BG; locality: Malko Tarnovo, "Valchanov most" Place; verbatimElevation: 281; verbatimCoordinates: N41°56'39.0", E27°36'00.2"; geodeticDatum: WGS84; **Event:** eventDate: 18/04/2010; habitat: oak forest
- **Type status:** Other material. **Occurrence:** recordedBy: R. Kostova; individualCount: 1; **Location:** countryCode: BG; locality: Bliznak Vill., PA"Bataka"; verbatimElevation: 324; verbatimCoordinates: N42°11'37.3", E27°19'35.8"; geodeticDatum: WGS84; **Event:** eventDate: 03.08-07.09.2009; habitat: oak forest
- **Type status:** Other material. **Occurrence:** recordedBy: R. Kostova; individualCount: 1721; **Location:** countryCode: BG; locality: Malko Tarnovo, "Propada" Place; verbatimElevation: 401; verbatimCoordinates: N41°58'49.6", E27°29'25.7"; geodeticDatum: WGS84; **Event:** eventDate: 15.04-07.09.2009; habitat: oriental beech forest
- **Type status:** Other material. **Occurrence:** recordedBy: R. Kostova; individualCount: 80; **Location:** countryCode: BG; locality: Slivarovo Vill., "Shafariitsa" Place; verbatimElevation: 224; verbatimCoordinates: N41°57'37.8", E27°39'34.8"; geodeticDatum: WGS84; **Event:** eventDate: 15.04-02.08.2009; habitat: black alder-oak forest
- **Type status:** Other material. **Occurrence:** recordedBy: R. Kostova; individualCount: 1; **Location:** countryCode: BG; locality: Kosti Vill., "St. Ilia" Place; verbatimElevation: 35; verbatimCoordinates: N42°03'23.2", E27°45'51.6"; geodeticDatum: WGS84; **Event:** eventDate: 09.05-08.06.2009; habitat: meadow with single trees
- **Type status:** Other material. **Occurrence:** recordedBy: R. Kostova; individualCount: 1; **Location:** countryCode: BG; locality: Bulgari Vill., PA "Marina reka"; verbatimElevation: 183; verbatimCoordinates: N42°06'41.7", E27°45'53.0"; geodeticDatum: WGS84; **Event:** eventDate: 03.07-02.08.2009; habitat: oriental beech-oak forest with Rododendron ponticum
- **Type status:** Other material. **Occurrence:** recordedBy: R. Kostova; individualCount: 1; **Location:** countryCode: BG; locality: Brodilovo Vill., under Papiya peak; verbatimElevation: 336; verbatimCoordinates: N42°06'23.7", E27°50'36.1"; geodeticDatum: WGS84; **Event:** eventDate: 15.04-08.05.2009; habitat: oak forest
- **Type status:** Other material. **Occurrence:** recordedBy: R. Kostova; individualCount: 1; **Location:** countryCode: BG; locality: Sinemorets Vill., PA"Silistar", "Butamyata" Place; verbatimElevation: 17; verbatimCoordinates: N42°03'10.3", E27°59'13.4"; geodeticDatum: WGS84; **Event:** eventDate: 09.05-08.06.2009; habitat: oak forest
- **Type status:** Other material. **Occurrence:** recordedBy: R. Kostova; individualCount: 17; **Location:** countryCode: BG; locality: Mladezhko Vill., The springs of Mladezhka River; verbatimElevation: 231; verbatimCoordinates: N42°09'04.5", E27°21'26.1"; geodeticDatum: WGS84; **Event:** eventDate: 09.05-07.09.2009; habitat: black alder and hornbeam forest
- **Type status:** Other material. **Occurrence:** recordedBy: R. Kostova; individualCount: 65; **Location:** countryCode: TR; locality: Igneada surroundings; verbatimElevation: 15; verbatimCoordinates: N41°51'51.0", E27°56'54.1"; geodeticDatum: WGS84; **Event:** eventDate: 06.07-29.09.2009; habitat: swamp forest
- **Type status:** Other material. **Occurrence:** recordedBy: R. Kostova; individualCount: 33; **Location:** countryCode: TR; locality: Gokyaka Vill., Road to Dupnisa Cave; verbatimElevation: 337; verbatimCoordinates: N41°52'20.0", E27°37'06.4"; geodeticDatum: WGS84; **Event:** eventDate: 06.07 - 29.09.2009; habitat: oak-hornbeam mixed forest
- **Type status:** Other material. **Location:** countryCode: BG; locality: Maslen nos Cape (= Zejtinburun); **Record Level:** bibliographicCitation: Hůrka (1988: 297)
- **Type status:** Other material. **Location:** countryCode: BG; locality: Bosna Ridge; **Record Level:** bibliographicCitation: Hůrka (1988: 297)
- **Type status:** Other material. **Location:** countryCode: BG; locality: Maslen nos Cape; **Record Level:** bibliographicCitation: Hůrka (1988: 297)
- **Type status:** Other material. **Location:** countryCode: BG; locality: Bosna Peak; **Record Level:** bibliographicCitation: Hůrka (1988: 297)
- **Type status:** Other material. **Occurrence:** recordedBy: B. Guéorguiev; individualCount: 4; **Location:** countryCode: BG; locality: Veleka River near Mladezko Village; **Event:** eventDate: 25/05/1995; **Record Level:** institutionCode: NMNHS
- **Type status:** Other material. **Occurrence:** recordedBy: B. Guéorguiev; individualCount: 5; **Location:** countryCode: BG; locality: Aydere River; verbatimElevation: 350-450; **Event:** eventDate: 26/05/1995; **Record Level:** institutionCode: NMNHS
- **Type status:** Other material. **Occurrence:** recordedBy: B. Guéorguiev; individualCount: 2; **Location:** countryCode: BG; locality: Primorsko Town env.; **Event:** eventDate: 20/08/2004; habitat: oak forest; fieldNumber: un; **Record Level:** institutionCode: NMNHS

#### Brachinus (Brachinus) alexandri

F. Battoni, 1984

##### Materials

- **Type status:** Other material. **Location:** countryCode: BG; locality: Primorsko; **Record Level:** bibliographicCitation: Hieke & Wrase (1988: 165)
- **Type status:** Other material. **Location:** countryCode: BG; locality: Tsarevo (= Micurin); **Record Level:** bibliographicCitation: Hůrka (1988: 305)
- **Type status:** Other material. **Occurrence:** recordedBy: V. Lazarov; individualCount: 2; **Location:** countryCode: BG; locality: Primorsko; **Event:** eventDate: VI.1957; **Record Level:** institutionCode: NMNHS

#### Brachinus (Brachinus) berytensis

Reiche, 1855

##### Materials

- **Type status:** Other material. **Location:** countryCode: BG; locality: Lozenets; **Record Level:** bibliographicCitation: Hůrka (1988: 305)
- **Type status:** Other material. **Location:** countryCode: BG; locality: Ahtopol; **Record Level:** bibliographicCitation: Wrase (1991: 18)

#### Brachinus (Brachinus) crepitans

(Linnaeus, 1758)

##### Materials

- **Type status:** Other material. **Occurrence:** recordedBy: R. Kostova; individualCount: 4; **Location:** countryCode: BG; locality: Rezovo Vill. surroundings; verbatimElevation: 5; verbatimCoordinates: N41°58'54.4", E28°01'29.6"; geodeticDatum: WGS84; **Event:** eventDate: 09/05/2009; habitat: meadows
- **Type status:** Other material. **Occurrence:** recordedBy: P. Mitov; individualCount: 1; **Location:** countryCode: BG; locality: Rezovo Vill. surroundings; verbatimElevation: 5; verbatimCoordinates: N41°59'06.1", E28°02'04.2"; geodeticDatum: WGS84; **Event:** eventDate: VI-IX.2009; habitat: meadows
- **Type status:** Other material. **Occurrence:** recordedBy: R. Kostova; individualCount: 1; **Location:** countryCode: BG; locality: Sinemorets Vill., PA "Estuary of Veleka river"; verbatimElevation: 6; verbatimCoordinates: N42°03'39.6", E27°57'55.2"; geodeticDatum: WGS84; **Event:** eventDate: 30/05/2010
- **Type status:** Other material. **Occurrence:** recordedBy: R. Kostova; individualCount: 1; **Location:** countryCode: TR; locality: Sarpdere Vill., Dupnisa Cave surroundings; verbatimElevation: 356; verbatimCoordinates: N41°50'26.0", E27°33'22.4"; geodeticDatum: WGS84; **Event:** eventDate: 01/10/2009
- **Type status:** Other material. **Occurrence:** recordedBy: R. Kostova; individualCount: 2; **Location:** countryCode: BG; locality: Kosti Vill., "St. Ilia" Place; verbatimElevation: 35; verbatimCoordinates: N42°03'23.2", E27°45'51.6"; geodeticDatum: WGS84; **Event:** eventDate: 03.07-07.09.2009; habitat: meadow with single trees
- **Type status:** Other material. **Occurrence:** recordedBy: R. Kostova; individualCount: 1; **Location:** countryCode: BG; locality: Sinemorets Vill., PA"Silistar", "Butamyata" Place; verbatimElevation: 10; verbatimCoordinates: N42°03'11.2", E27°59'19.7"; geodeticDatum: WGS84; **Event:** eventDate: 03.08-07.09.2009; habitat: meadow, single shrubs
- **Type status:** Other material. **Location:** countryCode: BG; locality: Strandzha; **Record Level:** bibliographicCitation: Rambousek (1912: 102)
- **Type status:** Other material. **Location:** countryCode: BG; locality: Ahtopol; **Record Level:** bibliographicCitation: Hieke & Wrase (1988: 164)
- **Type status:** Other material. **Location:** countryCode: BG; locality: Ropotamo; **Record Level:** bibliographicCitation: Hieke & Wrase (1988: 164)
- **Type status:** Other material. **Occurrence:** recordedBy: B. Guéorguiev; individualCount: 1; **Location:** countryCode: BG; locality: Sinemorets Village, south bay; **Event:** eventDate: 27/05/1995; **Record Level:** institutionCode: NMNHS
- **Type status:** Other material. **Occurrence:** recordedBy: B. Guéorguiev; individualCount: 1; **Location:** countryCode: BG; locality: route Primorsko Town - Pismenovo Vill.; verbatimElevation: 50-90; **Event:** eventDate: 26/08/2004; fieldNumber: un; **Record Level:** institutionCode: NMNHS

#### Brachinus (Brachinus) ejaculans

Fischer von Waldheim, 1828

##### Materials

- **Type status:** Other material. **Occurrence:** recordedBy: J. Mařan & K. Taborsky; individualCount: 2; **Location:** countryCode: BG; locality: Maslen nos Cape; **Record Level:** institutionCode: NMP
- **Type status:** Other material. **Location:** countryCode: BG; locality: Kiten; **Record Level:** bibliographicCitation: Hieke & Wrase (1988: 165)
- **Type status:** Other material. **Location:** countryCode: BG; locality: Ropotamo; **Record Level:** bibliographicCitation: Hieke & Wrase (1988: 165)
- **Type status:** Other material. **Occurrence:** recordedBy: B. Guéorguiev; individualCount: 2; **Location:** countryCode: BG; locality: Sinemorets Village, south bay; **Event:** eventDate: 27/05/1995; **Record Level:** institutionCode: NMNHS

#### Brachinus (Brachinus) elegans

Chaudoir, 1842

##### Materials

- **Type status:** Other material. **Occurrence:** recordedBy: R. Kostova; individualCount: 2; **Location:** countryCode: BG; locality: Brodilovo Vill, along Eleshnitsa River; verbatimElevation: 17; verbatimCoordinates: N42°05'35.4", E27°49'53.0"; geodeticDatum: WGS84; **Event:** eventDate: 05/07/2009
- **Type status:** Other material. **Occurrence:** recordedBy: R. Kostova; individualCount: 1; **Location:** countryCode: BG; locality: Kosti Vill., "St. Ilia" Place; verbatimElevation: 35; verbatimCoordinates: N42°03'23.2", E27°45'51.6"; geodeticDatum: WGS84; **Event:** eventDate: 09.05-08.06.2009; habitat: meadow with single trees
- **Type status:** Other material. **Occurrence:** recordedBy: R. Kostova; individualCount: 2; **Location:** countryCode: BG; locality: Sinemorets Vill., PA "Estuary of Veleka river"; verbatimElevation: 6; verbatimCoordinates: N42°03'39.6", E27°57'55.2"; geodeticDatum: WGS84; **Event:** eventDate: 09.06-02.07.2009; habitat: swamp forest
- **Type status:** Other material. **Location:** countryCode: BG; locality: Ropotamo; **Record Level:** bibliographicCitation: Hieke & Wrase (1988: 164, as B. ganglbaueri)
- **Type status:** Other material. **Occurrence:** recordedBy: B. Guéorguiev; individualCount: 2; **Location:** countryCode: BG; locality: mouth of Veleka River near Sinemorets Vill.; **Event:** eventDate: 28/05/1995; **Record Level:** institutionCode: NMNHS

#### Brachinus (Brachinus) plagiatus

Reiche, 1868

##### Materials

- **Type status:** Other material. **Occurrence:** recordedBy: R. Bekchiev; individualCount: 1; **Location:** countryCode: BG; locality: Primorsko, Perla Beach; verbatimElevation: 1; verbatimCoordinates: N42°17'10.2", E27°45'13.6"; geodeticDatum: WGS84; **Event:** eventDate: 30/06/2009
- **Type status:** Other material. **Occurrence:** recordedBy: J. Mařan & K. Taborsky; individualCount: 2; **Location:** countryCode: BG; locality: Maslen nos Cape; **Record Level:** institutionCode: NMP
- **Type status:** Other material. **Occurrence:** recordedBy: P. Beron; individualCount: 1; **Location:** countryCode: BG; locality: Kiten; **Event:** eventDate: 16-22.12.1984; **Record Level:** institutionCode: NMNHS
- **Type status:** Other material. **Location:** countryCode: BG; locality: Ropotamo; **Record Level:** bibliographicCitation: Hieke & Wrase (1988: 165)

#### Brachinus (Brachinus) psophia

Audinet-Serville, 1821

##### Materials

- **Type status:** Other material. **Occurrence:** recordedBy: R. Kostova; individualCount: 1; **Location:** countryCode: BG; locality: Rezovo Vill. surroundings; verbatimElevation: 5; verbatimCoordinates: N41°58'54.4", E28°01'29.6"; geodeticDatum: WGS84; **Event:** eventDate: 09/05/2009; habitat: meadows
- **Type status:** Other material. **Occurrence:** recordedBy: J. Mařan & K. Taborsky; individualCount: 6; **Location:** countryCode: BG; locality: Maslen nos Cape; **Event:** eventDate: VI.1933; **Record Level:** institutionCode: NMP
- **Type status:** Other material. **Location:** countryCode: BG; locality: Primorsko; **Record Level:** bibliographicCitation: Hieke & Wrase (1988: 164)
- **Type status:** Other material. **Location:** countryCode: BG; locality: Ropotamo; **Record Level:** bibliographicCitation: Hieke & Wrase (1988: 164)

#### Brachinus (Brachynidius) bodemeyeri

Apfelbeck, 1904

##### Materials

- **Type status:** Other material. **Occurrence:** recordedBy: R. Kostova; individualCount: 1; **Location:** countryCode: BG; locality: Malko Tarnovo, "Propada" Place; verbatimElevation: 388; verbatimCoordinates: N41°58'52.5", E27°29'28.6"; geodeticDatum: WGS84; **Event:** eventDate: 17/04/2009; habitat: meadow
- **Type status:** Other material. **Occurrence:** recordedBy: P. Mitov; individualCount: 6; **Location:** countryCode: BG; locality: Rezovo Vill. surroundings; verbatimElevation: 5; verbatimCoordinates: N41°59'06.1", E28°02'04.2"; geodeticDatum: WGS84; **Event:** eventDate: VI-IX.2009; habitat: meadows
- **Type status:** Other material. **Location:** countryCode: BG; locality: Kosti Vill.; **Record Level:** bibliographicCitation: Guéorguiev & Guéorguiev (1995: 240)
- **Type status:** Other material. **Location:** countryCode: BG; locality: Rezovo Vill.; **Record Level:** bibliographicCitation: Guéorguiev & Guéorguiev (1995: 240)
- **Type status:** Other material. **Occurrence:** recordedBy: J. Mařan & K. Taborsky; individualCount: 14; **Location:** countryCode: BG; locality: Maslen nos Cape; **Event:** eventDate: VI.1933; **Record Level:** institutionCode: NMP
- **Type status:** Other material. **Location:** countryCode: BG; locality: Tsarevo (= Micurin); **Record Level:** bibliographicCitation: Hieke & Wrase (1988: 166)
- **Type status:** Other material. **Location:** countryCode: BG; locality: Ropotamo; **Record Level:** bibliographicCitation: Hieke & Wrase (1988: 166)
- **Type status:** Other material. **Occurrence:** recordedBy: P. Beron; individualCount: 2; **Location:** countryCode: BG; locality: Kiten; **Event:** eventDate: 16-22.12.1984; **Record Level:** institutionCode: NMNHS
- **Type status:** Other material. **Occurrence:** recordedBy: B. Petrov; individualCount: 3; **Location:** countryCode: BG; locality: Maslen nos Cape; **Event:** eventDate: 19/04/2002; habitat: litter of Quercus sp.; **Record Level:** institutionCode: NMNHS

#### Brachinus (Brachynidius) brevicollis

Motschulsky, 1844

##### Materials

- **Type status:** Other material. **Occurrence:** recordedBy: J. Mařan & K. Taborsky; individualCount: 1; **Location:** countryCode: BG; locality: Maslen nos Cape; **Event:** eventDate: VI.1933
- **Type status:** Other material. **Location:** countryCode: BG; locality: Kiten; **Record Level:** bibliographicCitation: Hieke & Wrase (1988: 165, as B. peregrinus)
- **Type status:** Other material. **Location:** countryCode: BG; locality: Tsarevo (= Micurin); **Record Level:** bibliographicCitation: Hieke & Wrase (1988: 165, as B. peregrinus)
- **Type status:** Other material. **Location:** countryCode: BG; locality: Ropotamo; **Record Level:** bibliographicCitation: Hieke & Wrase (1988: 165, as B. peregrinus)

#### Brachinus (Brachynidius) explodens

Duftschmid, 1812

##### Materials

- **Type status:** Other material. **Occurrence:** recordedBy: R. Kostova; individualCount: 1; **Location:** countryCode: BG; locality: Stoilovo Vill., "Petrova niva" Place; verbatimElevation: 132; verbatimCoordinates: N42°03'41.9", E27°31'58.9"; geodeticDatum: WGS84; **Event:** eventDate: 06/05/2009; habitat: meadow, single shrubs
- **Type status:** Other material. **Occurrence:** recordedBy: R. Kostova; individualCount: 1; **Location:** countryCode: BG; locality: Primorsko, Perla Beach; verbatimElevation: 1; verbatimCoordinates: N42°17'10.2", E27°45'13.6"; geodeticDatum: WGS84; **Event:** eventDate: 10/05/2009
- **Type status:** Other material. **Occurrence:** recordedBy: R. Kostova; individualCount: 2; **Location:** countryCode: TR; locality: Sarpdere Vill., Dupnisa Cave surroundings; verbatimElevation: 356; verbatimCoordinates: N41°50'26.0", E27°33'22.4"; geodeticDatum: WGS84; **Event:** eventDate: 01/10/2009
- **Type status:** Other material. **Occurrence:** recordedBy: R. Kostova; individualCount: 1; **Location:** countryCode: TR; locality: Kiyiköy surroundings; verbatimElevation: 38; verbatimCoordinates: N41°39'25.7", E28°05'11.2"; geodeticDatum: WGS84; **Event:** eventDate: 24/05/2010
- **Type status:** Other material. **Occurrence:** recordedBy: R. Kostova; individualCount: 2; **Location:** countryCode: TR; locality: Kiyiköy surroundings; verbatimElevation: 38; verbatimCoordinates: N41°39'25.7", E28°05'11.2"; geodeticDatum: WGS84; **Event:** eventDate: 22/05/2011
- **Type status:** Other material. **Occurrence:** recordedBy: R. Kostova; individualCount: 1; **Location:** countryCode: TR; locality: Yalikŏy, river estuary; verbatimElevation: 9; verbatimCoordinates: N41°29'27.7", E28°16'40.0"; geodeticDatum: WGS84; **Event:** eventDate: 23/05/2011
- **Type status:** Other material. **Occurrence:** recordedBy: J. Mařan & K. Taborsky; individualCount: 14; **Location:** countryCode: BG; locality: Maslen nos Cape; **Event:** eventDate: VI.1933; **Record Level:** institutionCode: NMP
- **Type status:** Other material. **Occurrence:** recordedBy: K. Taborsky; individualCount: 2; **Location:** countryCode: BG; locality: Primorsko; **Event:** eventDate: VII.1934; **Record Level:** institutionCode: NMP
- **Type status:** Other material. **Location:** countryCode: BG; locality: Rezovo Vill.; **Record Level:** bibliographicCitation: Guéorguiev & Guéorguiev (1995: 241)
- **Type status:** Other material. **Location:** countryCode: BG; locality: Velika Papiya; **Record Level:** bibliographicCitation: Guéorguiev & Guéorguiev (1995: 241)
- **Type status:** Other material. **Location:** countryCode: BG; locality: Kalovo Vill.; **Record Level:** bibliographicCitation: Guéorguiev & Guéorguiev (1995: 241)
- **Type status:** Other material. **Location:** countryCode: BG; locality: Kiten; **Record Level:** bibliographicCitation: Hieke & Wrase (1988: 166)
- **Type status:** Other material. **Occurrence:** recordedBy: P. Beron; individualCount: 5; **Location:** countryCode: BG; locality: Kiten; **Event:** eventDate: 16-22.12.1984; **Record Level:** institutionCode: NMNHS
- **Type status:** Other material. **Occurrence:** recordedBy: B. Guéorguiev; individualCount: 1; **Location:** countryCode: BG; locality: Sinemorets Village, south bay; **Event:** eventDate: 27/05/1995; **Record Level:** institutionCode: NMNHS
- **Type status:** Other material. **Occurrence:** recordedBy: B. Guéorguiev; individualCount: 1; **Location:** countryCode: BG; locality: mouth of Veleka River near Sinemorets Vill.; **Event:** eventDate: 28/05/1995; **Record Level:** institutionCode: NMNHS
- **Type status:** Other material. **Occurrence:** recordedBy: B. Guéorguiev; individualCount: 1; **Location:** countryCode: BG; locality: Ahtopol; **Event:** eventDate: 28/05/1995; **Record Level:** institutionCode: NMNHS

#### Clivina collaris

(Herbst, 1784)

##### Materials

- **Type status:** Other material. **Occurrence:** recordedBy: R. Kostova; individualCount: 1; **Location:** countryCode: BG; locality: Malko Tarnovo, "Propada" Place; verbatimElevation: 388; verbatimCoordinates: N41°58'52.5", E27°29'28.6"; geodeticDatum: WGS84; **Event:** eventDate: 17/04/2009; habitat: meadow
- **Type status:** Other material. **Occurrence:** recordedBy: R. Kostova; individualCount: 1; **Location:** countryCode: BG; locality: Malko Tarnovo, "Indipasqua" Place; verbatimElevation: 211; verbatimCoordinates: N42°00'16.9", E27°39'09.2"; geodeticDatum: WGS84; **Event:** eventDate: 18/04/2009
- **Type status:** Other material. **Occurrence:** recordedBy: R. Kostova; individualCount: 1; **Location:** countryCode: BG; locality: Kirovo Vill., along Selska River; verbatimElevation: 138; verbatimCoordinates: N42°10'43.0", E27°10'58.2"; geodeticDatum: WGS84; **Event:** eventDate: 05/05/2009
- **Type status:** Other material. **Occurrence:** recordedBy: R. Bekchiev; individualCount: 6; **Location:** countryCode: BG; locality: Kirovo Vill., along Selska River; verbatimElevation: 138; verbatimCoordinates: N42°10'43.0", E27°10'58.2"; geodeticDatum: WGS84; **Event:** eventDate: 05/05/2009
- **Type status:** Other material. **Occurrence:** recordedBy: R. Bekchiev; individualCount: 2; **Location:** countryCode: BG; locality: Varovnik Vill., near the bridge at Fakiiska River; verbatimElevation: 125; verbatimCoordinates: N42°13'33.7", E27°11'33.7"; geodeticDatum: WGS84; **Event:** eventDate: 09/06/2009
- **Type status:** Other material. **Occurrence:** recordedBy: R. Kostova; individualCount: 1; **Location:** countryCode: BG; locality: Demirköy, along Degirmen River; verbatimElevation: 289; verbatimCoordinates: N41°49'17.2", E27°45'07.7"; geodeticDatum: WGS84; **Event:** eventDate: 06/07/2009

#### Clivina fossor fossor

(Linnaeus, 1758)

##### Materials

- **Type status:** Other material. **Occurrence:** recordedBy: R. Bekchiev; individualCount: 1; **Location:** countryCode: TR; locality: Yalikŏy, river estuary; verbatimElevation: 9; verbatimCoordinates: N41°29'27.7", E28°16'40.0"; geodeticDatum: WGS84; **Event:** eventDate: 23/05/2011
- **Type status:** Other material. **Occurrence:** recordedBy: R. Kostova; individualCount: 1; **Location:** countryCode: TR; locality: Kizilagač, along river; verbatimElevation: 115; verbatimCoordinates: N41°41'01.9", E27°52'51.4"; geodeticDatum: WGS84; **Event:** eventDate: 24/05/2011

#### Clivina laevifrons

Chaudoir, 1842

##### Materials

- **Type status:** Other material. **Occurrence:** recordedBy: R. Bekchiev; individualCount: 1; **Location:** countryCode: BG; locality: Primorsko, Perla Beach; verbatimElevation: 1; verbatimCoordinates: N42°17'10.2", E27°45'13.6"; geodeticDatum: WGS84; **Event:** eventDate: 30/06/2009
- **Type status:** Other material. **Occurrence:** recordedBy: R. Bekchiev; individualCount: 4; **Location:** countryCode: BG; locality: Sinemorets Vill., PA "Estuary of Veleka river"; verbatimElevation: 6; verbatimCoordinates: N42°03'39.6", E27°57'55.2"; geodeticDatum: WGS84; **Event:** eventDate: 01/07/2009
- **Type status:** Other material. **Occurrence:** recordedBy: R. Kostova; individualCount: 7; **Location:** countryCode: TR; locality: Igneada surroundings; verbatimElevation: 3; verbatimCoordinates: N41°51'27.4", E27°57'32.2"; geodeticDatum: WGS84; **Event:** eventDate: 05/07/2009; habitat: marsh

#### Dyschirius (Dyschiriodes) agnatus

Motschulsky, 1844

##### Materials

- **Type status:** Other material. **Location:** countryCode: BG; locality: Strandzha; **Record Level:** bibliographicCitation: Guéorguiev (1992: 62)

#### Dyschirius (Dyschiriodes) chalceus

Erichson, 1837

##### Materials

- **Type status:** Other material. **Location:** countryCode: BG; locality: Primorsko; **Record Level:** bibliographicCitation: Guéorguiev (1992: 63)

#### Dyschirius (Dyschiriodes) chalybaeus gibbifrons

Apfelbeck, 1899

##### Materials

- **Type status:** Other material. **Location:** countryCode: BG; locality: Tsarevo (= Micurin); **Record Level:** bibliographicCitation: Hieke & Wrase (1988: 31)
- **Type status:** Other material. **Location:** countryCode: BG; locality: Ropotamo; **Record Level:** bibliographicCitation: Hieke & Wrase (1988: 31)

#### Dyschirius (Dyschiriodes) politus politus

(Dejean, 1825)

##### Materials

- **Type status:** Other material. **Location:** countryCode: BG; locality: Primorsko; **Record Level:** bibliographicCitation: Guéorguiev (1992: 62)
- **Type status:** Other material. **Location:** countryCode: BG; locality: Primorsko; **Record Level:** bibliographicCitation: Guéorguiev & Guéorguiev (1995: 69, as D. politus)

#### Dyschirius (Dyschiriodes) salinus striatopunctatus

Putzeys, 1846

##### Materials

- **Type status:** Other material. **Location:** countryCode: BG; locality: Primorsko; **Record Level:** bibliographicCitation: Guéorguiev (1992: 63)

#### Dyschirius (Dyschiriodes) tristis

Stephens, 1827

##### Materials

- **Type status:** Other material. **Occurrence:** recordedBy: R. Bekchiev; individualCount: 1; **Location:** countryCode: TR; locality: Along Demirköy - Igneada road; verbatimElevation: 12; verbatimCoordinates: N41°52'39.3", E27°56'29.2"; geodeticDatum: WGS84; **Event:** eventDate: 05/07/2009
- **Type status:** Other material. **Occurrence:** recordedBy: R. Bekchiev; individualCount: 2; **Location:** countryCode: TR; locality: Igneada surroundings; verbatimElevation: 3; verbatimCoordinates: N41°51'27.4", E27°57'32.2"; geodeticDatum: WGS84; **Event:** eventDate: 05/07/2009; habitat: marsh
- **Type status:** Other material. **Location:** countryCode: BG; locality: Primorsko; **Record Level:** bibliographicCitation: Guéorguiev & Guéorguiev (1995: 67)

#### Dyschirius (Dyschirius) caspius

Putzeys, 1866

##### Materials

- **Type status:** Other material. **Location:** countryCode: BG; locality: Primorsko, Ropotamo River; **Record Level:** bibliographicCitation: Guéorguiev (1992: 62)

#### Dyschirius (Dyschirius) thoracicus

(P. Rossi, 1790)

##### Materials

- **Type status:** Other material. **Location:** countryCode: BG; locality: Sinemorets Vill. env.; **Record Level:** bibliographicCitation: Guéorguiev & Muilwijk (2001: 113-114, as Dyschirius arenosus Stephens)

##### Notes

[= arenosus Stephens, 1827]

#### Dyschirius (Eudyschirius) globosus

(Herbst, 1784)

##### Materials

- **Type status:** Other material. **Occurrence:** recordedBy: R. Bekchiev; individualCount: 3; **Location:** countryCode: BG; locality: Primorsko, Perla Beach; verbatimElevation: 1; verbatimCoordinates: N42°17'10.2", E27°45'13.6"; geodeticDatum: WGS84; **Event:** eventDate: 10/05/2009
- **Type status:** Other material. **Occurrence:** recordedBy: R. Bekchiev; individualCount: 2; **Location:** countryCode: TR; locality: Igneada, Hamam Gölü; verbatimElevation: 5; verbatimCoordinates: N41°49'43.2", E27°57'31.1"; geodeticDatum: WGS84; **Event:** eventDate: 02/10/2009
- **Type status:** Other material. **Occurrence:** recordedBy: R. Kostova; individualCount: 59; **Location:** countryCode: BG; locality: Sinemorets Vill., PA "Estuary of Veleka river"; verbatimElevation: 6; verbatimCoordinates: N42°03'39.6", E27°57'55.2"; geodeticDatum: WGS84; **Event:** eventDate: 15.04-02.07.2009; habitat: swamp forest
- **Type status:** Other material. **Occurrence:** recordedBy: B. Guéorguiev; individualCount: 1; **Location:** countryCode: BG; locality: Sinemorets, Veleka River; **Event:** eventDate: 28/05/1995; **Record Level:** institutionCode: NMNHS

#### Scarites (Parallelomorphus) laevigatus

Fabricius, 1792

##### Materials

- **Type status:** Other material. **Occurrence:** recordedBy: D. Iltchev; individualCount: 2; **Location:** countryCode: BG; locality: "Vasiliko", Tsarevo (= Micurin); **Event:** eventDate: 24/06/1921; **Record Level:** institutionCode: NMNHS

#### Scarites (Parallelomorphus) terricola terricola

Bonelli, 1813

##### Materials

- **Type status:** Other material. **Occurrence:** recordedBy: J. Mařan & K. Taborsky; individualCount: 1; **Location:** countryCode: BG; locality: Maslen nos Cape; **Event:** eventDate: VI.1933; **Record Level:** institutionCode: NMP
- **Type status:** Other material. **Location:** countryCode: BG; locality: Kiten; **Record Level:** bibliographicCitation: Hieke & Wrase (1988: 27)
- **Type status:** Other material. **Location:** countryCode: BG; locality: Tsarevo (= Micurin); **Record Level:** bibliographicCitation: Hieke & Wrase (1988: 27)

#### Broscus nobilis

(Dejean, 1828)

##### Materials

- **Type status:** Other material. **Location:** countryCode: BG; locality: Sinemorets Vill.; **Record Level:** bibliographicCitation: Guéorguiev & Guéorguiev (1995: 71)

#### Apotomus clypeonitens adanensis

Jedlička, 1961

##### Materials

- **Type status:** Other material. **Location:** countryCode: BG; locality: Ahtopol; **Record Level:** bibliographicCitation: Wrase (1991: 5)
- **Type status:** Other material. **Location:** countryCode: BG; locality: Ahtopol; **Record Level:** bibliographicCitation: Guéorguiev & Guéorguievv (1995: 72)
- **Type status:** Other material. **Occurrence:** recordedBy: B. Guéorguiev; individualCount: 2; **Location:** countryCode: BG; locality: Primorsko Town env.; **Event:** eventDate: 20/08/2004; habitat: oak forest; fieldNumber: un; **Record Level:** institutionCode: NMNHS

#### Apotomus rufus

(P. Rossi, 1790)

##### Materials

- **Type status:** Other material. **Location:** countryCode: BG; locality: Ahtopol, 50 м; **Record Level:** bibliographicCitation: Vassilev & Necheva (1989: 50)
- **Type status:** Other material. **Location:** countryCode: BG; locality: Ahtopol; **Record Level:** bibliographicCitation: Guéorguiev & Guéorguiev (1995: 72)

##### Notes

We consider that the identification of this taxon may be doubtful. However, we cannot find and revise this material, so the species is tretaed here as questionable for both the regional fauna and the fauna of Bulgaria.

#### Apotomus testaceus

Dejean, 1825

##### Materials

- **Type status:** Other material. **Location:** countryCode: BG; locality: Maslen nos Cape; **Record Level:** bibliographicCitation: Guéorguiev (1992: 63)
- **Type status:** Other material. **Location:** countryCode: BG; locality: Maslen nos Cape; **Record Level:** bibliographicCitation: Guéorguiev & Guéorguiev (1995: 72)

##### Notes

The only specimen published by Guéorguiev (1992: 63) has been revised and proved to be Apotomus clypeonitens adanensis Jedlička. Hence, Apotomus testaceus Dejean is removed from both the list of the Bulgarian and Balkan fauna.

#### Asaphidion flavicorne

(Solsky, 1874)

##### Materials

- **Type status:** Other material. **Occurrence:** recordedBy: R. Kostova; individualCount: 1; **Location:** countryCode: BG; locality: Brodilovo Vill, along Eleshnitsa River; verbatimElevation: 17; verbatimCoordinates: N42°05'35.4", E27°49'53.0"; geodeticDatum: WGS84; **Event:** eventDate: 04/07/2009

#### Asaphidion flavipes

(Linnaeus, 1761)

##### Materials

- **Type status:** Other material. **Occurrence:** recordedBy: R. Kostova; individualCount: 1; **Location:** countryCode: BG; locality: Varvara Vill. surroundings; verbatimElevation: 46; verbatimCoordinates: N42°06'49.1", E27°53'18.9"; geodeticDatum: WGS84; **Event:** eventDate: 10/05/2009
- **Type status:** Other material. **Occurrence:** recordedBy: R. Kostova; individualCount: 3; **Location:** countryCode: TR; locality: Demirköy, to Mahya Dağı Peak; verbatimElevation: 488; verbatimCoordinates: N41°52'41.1", E27°54'27.0"; geodeticDatum: WGS84; **Event:** eventDate: 05/07/2009
- **Type status:** Other material. **Occurrence:** recordedBy: R. Kostova; individualCount: 1; **Location:** countryCode: BG; locality: Brodilovo Vill, along Eleshnitsa River; verbatimElevation: 17; verbatimCoordinates: N42°05'35.4", E27°49'53.0"; geodeticDatum: WGS84; **Event:** eventDate: 05/07/2009
- **Type status:** Other material. **Occurrence:** recordedBy: A. Gjonova; individualCount: 1; **Location:** countryCode: TR; locality: Yalikŏy, river estuary; verbatimElevation: 9; verbatimCoordinates: N41°29'27.7", E28°16'40.0"; geodeticDatum: WGS84; **Event:** eventDate: 23/05/2011
- **Type status:** Other material. **Occurrence:** recordedBy: R. Kostova; individualCount: 16; **Location:** countryCode: BG; locality: Slivarovo Vill., "Shafariitsa" Place; verbatimElevation: 224; verbatimCoordinates: N41°57'37.8", E27°39'34.8"; geodeticDatum: WGS84; **Event:** eventDate: 15.04-02.07.2009; habitat: black alder-oak forest
- **Type status:** Other material. **Occurrence:** recordedBy: R. Kostova; individualCount: 24; **Location:** countryCode: BG; locality: Kosti Vill., "St. Ilia" Place; verbatimElevation: 35; verbatimCoordinates: N42°03'23.2", E27°45'51.6"; geodeticDatum: WGS84; **Event:** eventDate: 15.04-08.06.2009; habitat: meadow with single trees
- **Type status:** Other material. **Occurrence:** recordedBy: R. Kostova & R. Bekchiev; individualCount: 23; **Location:** countryCode: BG; locality: Sinemorets Vill., PA "Estuary of Veleka river"; verbatimElevation: 6; verbatimCoordinates: N42°03'39.6", E27°57'55.2"; geodeticDatum: WGS84; **Event:** eventDate: 15.04-02.07.2009; habitat: swamp forest
- **Type status:** Other material. **Occurrence:** recordedBy: B. Guéorguiev; individualCount: 2; **Location:** countryCode: BG; locality: Rezovska River at Uzunbudzhak Reserve; **Event:** eventDate: 26/05/1995; **Record Level:** institutionCode: NMNHS

#### Bembidion (Bembidionetolitzkya) tibiale

(Duftschmid, 1812)

##### Materials

- **Type status:** Other material. **Occurrence:** recordedBy: R. Kostova; individualCount: 2; **Location:** countryCode: BG; locality: Malko Tarnovo, "Indipasqua" Place; verbatimElevation: 211; verbatimCoordinates: N42°00'16.9", E27°39'09.2"; geodeticDatum: WGS84; **Event:** eventDate: 17/04/2009
- **Type status:** Other material. **Occurrence:** recordedBy: R. Kostova; individualCount: 2; **Location:** countryCode: TR; locality: Demirköy, along Degirmen River; verbatimElevation: 289; verbatimCoordinates: N41°49'17.2", E27°45'07.7"; geodeticDatum: WGS84; **Event:** eventDate: 06/07/2009
- **Type status:** Other material. **Occurrence:** recordedBy: R. Kostova; individualCount: 2; **Location:** countryCode: TR; locality: Sergen Vill. surroundings; verbatimElevation: 818; verbatimCoordinates: N41°44'37.0", E27°42'24.8"; geodeticDatum: WGS84; **Event:** eventDate: 25/05/2011
- **Type status:** Other material. **Location:** countryCode: BG; locality: 15 km north of Malko Tarnovo; **Record Level:** bibliographicCitation: Hieke & Wrase (1988: 47)
- **Type status:** Other material. **Occurrence:** recordedBy: B. Guéorguiev; individualCount: 1; **Location:** countryCode: BG; locality: Veleka River near Mladezko Village; **Event:** eventDate: 25/05/1995; **Record Level:** institutionCode: NMNHS

#### Bembidion (Diplocampa) assimile

Gyllenhal, 1810

##### Materials

- **Type status:** Other material. **Occurrence:** recordedBy: R. Kostova; individualCount: 1; **Location:** countryCode: BG; locality: Sinemorets Vill., PA"Silistar"; verbatimElevation: 29; verbatimCoordinates: N42°01'33.6", E28°00'48.0"; geodeticDatum: WGS84; **Event:** eventDate: 09/05/2009; habitat: oak forest
- **Type status:** Other material. **Occurrence:** recordedBy: I. Gijonov; individualCount: 1; **Location:** countryCode: BG; locality: Rezovo Vill. surroundings; verbatimElevation: 5; verbatimCoordinates: N41°58'54.4", E28°01'29.6"; geodeticDatum: WGS84; **Event:** eventDate: 09/05/2009; habitat: meadows
- **Type status:** Other material. **Occurrence:** recordedBy: R. Bekchiev; individualCount: 5; **Location:** countryCode: BG; locality: Primorsko, Perla Beach; verbatimElevation: 1; verbatimCoordinates: N42°17'10.2", E27°45'13.6"; geodeticDatum: WGS84; **Event:** eventDate: 30/06/2009
- **Type status:** Other material. **Occurrence:** recordedBy: A. Gijonova; individualCount: 3; **Location:** countryCode: TR; locality: Igneada, Hamam Gölü; verbatimElevation: 11; verbatimCoordinates: N41°49'31.6", E27°57'33.8"; geodeticDatum: WGS84; **Event:** eventDate: 02/10/2009
- **Type status:** Other material. **Occurrence:** recordedBy: A. Gijonova; individualCount: 1; **Location:** countryCode: TR; locality: Igneada surroundings; verbatimElevation: 3; verbatimCoordinates: N41°51'27.4", E27°57'32.2"; geodeticDatum: WGS84; **Event:** eventDate: 02/10/2009; habitat: marsh
- **Type status:** Other material. **Occurrence:** recordedBy: R. Bekchiev; individualCount: 1; **Location:** countryCode: TR; locality: Igneada; verbatimElevation: 3; verbatimCoordinates: N41°52'51.3", E27°59'25.5"; geodeticDatum: WGS84; **Event:** eventDate: 02/10/2009
- **Type status:** Other material. **Location:** countryCode: BG; locality: Kiten; **Record Level:** bibliographicCitation: Hieke & Wrase (1988: 57)
- **Type status:** Other material. **Location:** countryCode: BG; locality: Tsarevo (= Micurin); **Record Level:** bibliographicCitation: Hieke & Wrase (1988: 57)

#### Bembidion (Diplocampa) fumigatum

(Duftschmid, 1812)

##### Materials

- **Type status:** Other material. **Occurrence:** recordedBy: R. Bekchiev; individualCount: 1; **Location:** countryCode: BG; locality: Fazanovo Vill., "Popovi skali" Place; verbatimElevation: 26; verbatimCoordinates: N42°09'45.9", E27°44'15.6"; geodeticDatum: WGS84; **Event:** eventDate: 01/07/2009
- **Type status:** Other material. **Location:** countryCode: BG; locality: Tsarevo (= Micurin); **Record Level:** bibliographicCitation: Hieke & Wrase (1988: 57)

#### Bembidion (Emphanes) azurescens azurescens

Dalla Torre, 1877

##### Materials

- **Type status:** Other material. **Location:** countryCode: BG; locality: Strandzha; **Record Level:** bibliographicCitation: Guéorguiev & Guéorguiev (1995: 103)

#### Bembidion (Emphanes) latiplaga latiplaga

Chaudoir, 1850

##### Materials

- **Type status:** Other material. **Location:** countryCode: BG; locality: Tsarevo (= Micurin); **Record Level:** bibliographicCitation: Hieke & Wrase (1988: 59)

#### Bembidion (Emphanes) minimum

(Fabricius, 1792)

##### Materials

- **Type status:** Other material. **Location:** countryCode: BG; locality: Tsarevo (= Micurin); **Record Level:** bibliographicCitation: Hieke & Wrase (1988: 58)

#### Bembidion (Emphanes) normannum apfelbecki

Müller-Motzfeld, 1986

##### Materials

- **Type status:** Other material. **Occurrence:** recordedBy: R. Kostova; individualCount: 1; **Location:** countryCode: TR; locality: Igneada; verbatimElevation: 3; verbatimCoordinates: N41°52'51.3", E27°59'25.5"; geodeticDatum: WGS84; **Event:** eventDate: 02/10/2009

#### Bembidion (Emphanes) tenellum tenellum

Erichson, 1837

##### Materials

- **Type status:** Other material. **Occurrence:** recordedBy: R. Kostova; individualCount: 1; **Location:** countryCode: BG; locality: Sinemorets Vill., PA"Silistar", Estuary of Silistar River; verbatimElevation: 12; verbatimCoordinates: N42°01'26.8", E28°00'32.9"; geodeticDatum: WGS84; **Event:** eventDate: 27/05/2011
- **Type status:** Other material. **Location:** countryCode: BG; locality: Ahtopol; **Record Level:** bibliographicCitation: Hieke & Wrase (1988: 58)
- **Type status:** Other material. **Location:** countryCode: BG; locality: Tsarevo (= Micurin); **Record Level:** bibliographicCitation: Hieke & Wrase (1988: 58)
- **Type status:** Other material. **Location:** countryCode: BG; locality: Ropotamo; **Record Level:** bibliographicCitation: Hieke & Wrase (1988: 58)
- **Type status:** Other material. **Location:** countryCode: BG; locality: 5 km West of Yasna Polyana Vill.; **Record Level:** bibliographicCitation: Hieke & Wrase (1988: 58)

#### Bembidion (Euperyphus) combustum combustum

Ménétriés, 1832

##### Materials

- **Type status:** Other material. **Location:** countryCode: BG; locality: Maslen nos Cape; **Record Level:** bibliographicCitation: Hieke & Wrase (1988: 49)
- **Type status:** Other material. **Location:** countryCode: BG; locality: Maslen nos Cape; **Record Level:** bibliographicCitation: Guéorguiev & Guéorguiev(1995: 112)

#### Bembidion (Metallina) lampros

(Herbst, 1784)

##### Materials

- **Type status:** Other material. **Occurrence:** recordedBy: R. Kostova; individualCount: 1; **Location:** countryCode: BG; locality: Mladezhko Vill., The springs of Mladezhka River; verbatimElevation: 231; verbatimCoordinates: N42°09'04.5", E27°21'26.1"; geodeticDatum: WGS84; **Event:** eventDate: 17/04/2009; habitat: black alder and hornbeam forest
- **Type status:** Other material. **Occurrence:** recordedBy: R. Kostova; individualCount: 3; **Location:** countryCode: BG; locality: Slivarovo Vill., "Shafariitsa" Place; verbatimElevation: 224; verbatimCoordinates: N41°57'37.8", E27°39'34.8"; geodeticDatum: WGS84; **Event:** eventDate: 15.04-02.07.2009; habitat: black alder-oak forest
- **Type status:** Other material. **Occurrence:** recordedBy: R. Kostova; individualCount: 3; **Location:** countryCode: TR; locality: Along Demirköy - Igneada road; verbatimElevation: 30; verbatimCoordinates: N41°52'41.1", E27°54'27.0"; geodeticDatum: WGS84; **Event:** eventDate: 05/07/2009
- **Type status:** Other material. **Occurrence:** recordedBy: R. Kostova; individualCount: 1; **Location:** countryCode: TR; locality: Igneada surroundings; verbatimElevation: 3; verbatimCoordinates: N41°51'27.3", E27°57'28.7"; geodeticDatum: WGS84; **Event:** eventDate: 06/07/2009; habitat: marsh
- **Type status:** Other material. **Occurrence:** recordedBy: R. Kostova; individualCount: 1; **Location:** countryCode: TR; locality: Demirköy, along Degirmen River; verbatimElevation: 289; verbatimCoordinates: N41°49'17.2", E27°45'07.7"; geodeticDatum: WGS84; **Event:** eventDate: 06/07/2009
- **Type status:** Other material. **Occurrence:** recordedBy: R. Kostova; individualCount: 1; **Location:** countryCode: BG; locality: Malko Tarnovo, Entrance to Vitanovo Reserve; verbatimElevation: 285; verbatimCoordinates: N42°01'22.9", E27°27'22.9"; geodeticDatum: WGS84; **Event:** eventDate: 27/05/2010
- **Type status:** Other material. **Occurrence:** recordedBy: R. Kostova; individualCount: 3; **Location:** countryCode: TR; locality: Yalikŏy, river estuary; verbatimElevation: 9; verbatimCoordinates: N41°29'27.7", E28°16'40.0"; geodeticDatum: WGS84; **Event:** eventDate: 23/05/2011
- **Type status:** Other material. **Occurrence:** recordedBy: R. Kostova; individualCount: 1; **Location:** countryCode: TR; locality: Sivriler Vill. suroundings; verbatimElevation: 379; verbatimCoordinates: N41°47'30.2", E27°50'00.9"; geodeticDatum: WGS84; **Event:** eventDate: 24/05/2011

#### Bembidion (Metallina) properans

(Stephens, 1828)

##### Materials

- **Type status:** Other material. **Location:** countryCode: BG; locality: Tsarevo (= Micurin); **Record Level:** bibliographicCitation: Hieke & Wrase (1988: 44)
- **Type status:** Other material. **Location:** countryCode: BG; locality: Primorsko; **Record Level:** bibliographicCitation: Hieke & Wrase (1988: 44)
- **Type status:** Other material. **Location:** countryCode: BG; locality: Ropotamo; **Record Level:** bibliographicCitation: Hieke & Wrase (1988: 44)

#### Bembidion (Nepha) vseteckai dissimile

J. Müller, 1943

##### Materials

- **Type status:** Other material. **Location:** countryCode: BG; locality: Malko Tarnovo; **Record Level:** bibliographicCitation: Guéorguiev & Guéorguiev (1995: 105, as Bembidion tetragrammum illigeri)
- **Type status:** Other material. **Occurrence:** recordedBy: C. Purkyně; individualCount: 1; **Location:** countryCode: BG; locality: Maslen nos Cape; **Record Level:** institutionCode: NMP (det. as Bembidion genei illigeri)

#### Bembidion (Notaphocampa) niloticum niloticum

Dejean, 1831

##### Materials

- **Type status:** Other material. **Location:** countryCode: BG; locality: Ropotamo River (= Carska reka), near Maslen nos Cape (= Zejtinborun); **Record Level:** bibliographicCitation: Mařan (1933: 89)
- **Type status:** Other material. **Location:** countryCode: BG; locality: Maslen nos Cape; **Record Level:** bibliographicCitation: Guéorguiev & Guéorguiev (1995: 97)

#### Bembidion (Notaphus) varium

(Olivier, 1795)

##### Materials

- **Type status:** Other material. **Location:** countryCode: BG; locality: Tsarevo (= Micurin); **Record Level:** bibliographicCitation: Hieke & Wrase (1988: 46)
- **Type status:** Other material. **Location:** countryCode: BG; locality: Primorsko; **Record Level:** bibliographicCitation: Hieke & Wrase (1988: 46)
- **Type status:** Other material. **Occurrence:** recordedBy: B. Guéorguiev; individualCount: 1; **Location:** countryCode: BG; locality: Veleka River near Mladezko Village; **Event:** eventDate: 25/05/1995; **Record Level:** institutionCode: NMNHS
- **Type status:** Other material. **Occurrence:** recordedBy: B. Guéorguiev; individualCount: 1; **Location:** countryCode: BG; locality: Arapya, near Tsarevo; **Event:** eventDate: 28/05/1995; **Record Level:** institutionCode: NMNHS

#### Bembidion (Ocydromus) decorum bodemeyeri

K. Daniel & J. Daniel, 1902

##### Materials

- **Type status:** Other material. **Location:** countryCode: BG; locality: Primorsko, Ropotamo Reserve, "Valley"; **Record Level:** bibliographicCitation: Müller-Motzfeld (1986b: 154)
- **Type status:** Other material. **Occurrence:** recordedBy: B. Guéorguiev; individualCount: 1; **Location:** countryCode: BG; locality: Veleka River near Mladezko Village; **Event:** eventDate: 25/05/1995; **Record Level:** institutionCode: NMNHS
- **Type status:** Other material. **Occurrence:** recordedBy: B. Guéorguiev; individualCount: 3; **Location:** countryCode: BG; locality: Primorsko Town env.; **Event:** eventDate: 20/08/2004; habitat: oak forest; fieldNumber: un; **Record Level:** institutionCode: NMNHS
- **Type status:** Other material. **Occurrence:** recordedBy: B. Guéorguiev; individualCount: 1; **Location:** countryCode: BG; locality: route Primorsko Town - Pismenovo Vill.; verbatimElevation: 50-90; **Event:** eventDate: 26/08/2004; fieldNumber: un; **Record Level:** institutionCode: NMNHS

#### Bembidion (Ocydromus) decorum decorum

(Panzer, 1799)

##### Materials

- **Type status:** Other material. **Occurrence:** recordedBy: R. Kostova; individualCount: 1; **Location:** countryCode: BG; locality: Brodilovo Vill, along Eleshnitsa River; verbatimElevation: 17; verbatimCoordinates: N42°05'35.4", E27°49'53.0"; geodeticDatum: WGS84; **Event:** eventDate: 05/07/2009
- **Type status:** Other material. **Location:** countryCode: BG; locality: Yasna Polyana Vill.; **Record Level:** bibliographicCitation: Hieke & Wrase (1988: 49)
- **Type status:** Other material. **Location:** countryCode: BG; locality: Primorsko, Ropotamo Reserve, "Coast"; **Record Level:** bibliographicCitation: Müller-Motzfeld (1986b: 154)

#### Bembidion (Ocydromus) siculum smyrnense

Apfelbeck, 1904

##### Materials

- **Type status:** Other material. **Occurrence:** recordedBy: R. Kostova; individualCount: 1; **Location:** countryCode: BG; locality: Malko Tarnovo, "Indipasqua" Place; verbatimElevation: 211; verbatimCoordinates: N42°00'16.9", E27°39'09.2"; geodeticDatum: WGS84; **Event:** eventDate: 17/04/2009
- **Type status:** Other material. **Occurrence:** recordedBy: R. Kostova; individualCount: 4; **Location:** countryCode: BG; locality: Kirovo Vill., along Selska River; verbatimElevation: 138; verbatimCoordinates: N42°10'43.0", E27°10'58.2"; geodeticDatum: WGS84; **Event:** eventDate: 05/05/2009
- **Type status:** Other material. **Occurrence:** recordedBy: R. Kostova; individualCount: 1; **Location:** countryCode: BG; locality: Stoilovo Vill., "Petrova niva" Place, along Veleka River; verbatimElevation: 96; verbatimCoordinates: N42°03'50.2", E27°32'46.0"; geodeticDatum: WGS84; **Event:** eventDate: 11/06/2009
- **Type status:** Other material. **Occurrence:** recordedBy: R. Bekchiev; individualCount: 4; **Location:** countryCode: BG; locality: Varvara Vill. surroundings; verbatimElevation: 46; verbatimCoordinates: N42°06'49.1", E27°53'18.9"; geodeticDatum: WGS84; **Event:** eventDate: 02/07/2009
- **Type status:** Other material. **Occurrence:** recordedBy: R. Kostova; individualCount: 6; **Location:** countryCode: TR; locality: Demirköy, along Degirmen River; verbatimElevation: 289; verbatimCoordinates: N41°49'17.2", E27°45'07.7"; geodeticDatum: WGS84; **Event:** eventDate: 06/07/2009
- **Type status:** Other material. **Occurrence:** recordedBy: R. Bekchiev; individualCount: 1; **Location:** countryCode: BG; locality: Fazanovo Vill., "Popovi skali" Place; verbatimElevation: 26; verbatimCoordinates: N42°09'45.9", E27°44'15.6"; geodeticDatum: WGS84; **Event:** eventDate: 26/07/2009
- **Type status:** Other material. **Occurrence:** recordedBy: R. Kostova; individualCount: 2; **Location:** countryCode: TR; locality: Kiyiköy surroundings; verbatimElevation: 38; verbatimCoordinates: N41°39'25.7", E28°05'11.2"; geodeticDatum: WGS84; **Event:** eventDate: 22/05/2011
- **Type status:** Other material. **Occurrence:** recordedBy: R. Kostova; individualCount: 1; **Location:** countryCode: TR; locality: Kizilagač, along river; verbatimElevation: 115; verbatimCoordinates: N41°41'01.9", E27°52'51.4"; geodeticDatum: WGS84; **Event:** eventDate: 24/05/2011
- **Type status:** Other material. **Occurrence:** recordedBy: R. Kostova; individualCount: 1; **Location:** countryCode: TR; locality: Sergen Vill. surroundings; verbatimElevation: 818; verbatimCoordinates: N41°44'37.0", E27°42'24.8"; geodeticDatum: WGS84; **Event:** eventDate: 25/05/2011
- **Type status:** Other material. **Occurrence:** recordedBy: R. Kostova; individualCount: 1; **Location:** countryCode: TR; locality: Sarpdere Vill., Dupnisa Cave surroundings; verbatimElevation: 356; verbatimCoordinates: N41°50'26.0", E27°33'22.4"; geodeticDatum: WGS84; **Event:** eventDate: 26/05/2011

#### Bembidion (Ocyturanes) praeustum

Dejean, 1831

##### Materials

- **Type status:** Other material. **Occurrence:** recordedBy: R. Bekchiev; individualCount: 1; **Location:** countryCode: BG; locality: Fazanovo Vill., "Popovi skali" Place; verbatimElevation: 26; verbatimCoordinates: N42°09'45.9", E27°44'15.6"; geodeticDatum: WGS84; **Event:** eventDate: 01/07/2009
- **Type status:** Other material. **Occurrence:** recordedBy: A. Gijonova; individualCount: 2; **Location:** countryCode: TR; locality: Demirköy, to Mahya Dağı Peak; verbatimElevation: 488; verbatimCoordinates: N41°52'41.1", E27°54'27.0"; geodeticDatum: WGS84; **Event:** eventDate: 05/07/2009

#### Bembidion (Ocyturanes) signatipenne

Jacquelin du Val, 1852

##### Materials

- **Type status:** Other material. **Occurrence:** recordedBy: R. Bekchiev; individualCount: 1; **Location:** countryCode: BG; locality: Fazanovo Vill., "Popovi skali" Place; verbatimElevation: 26; verbatimCoordinates: N42°09'45.9", E27°44'15.6"; geodeticDatum: WGS84; **Event:** eventDate: 01/07/2009

#### Bembidion (Omoperyphus) semibraccatum

Netolitzky, 1911

##### Materials

- **Type status:** Other material. **Occurrence:** recordedBy: R. Kostova; individualCount: 1; **Location:** countryCode: BG; locality: Brodilovo Vill, along Eleshnitsa River; verbatimElevation: 17; verbatimCoordinates: N42°05'35.4", E27°49'53.0"; geodeticDatum: WGS84; **Event:** eventDate: 04/07/2009

#### Bembidion (Peryphanes) dalmatinum dalmatinum

Dejean, 1831

##### Materials

- **Type status:** Other material. **Occurrence:** recordedBy: R. Kostova; individualCount: 2; **Location:** countryCode: TR; locality: Along Demirköy - Igneada road; verbatimElevation: 30; verbatimCoordinates: N41°52'41.1", E27°54'27.0"; geodeticDatum: WGS84; **Event:** eventDate: 05/07/2009
- **Type status:** Other material. **Occurrence:** recordedBy: R. Kostova; individualCount: 1; **Location:** countryCode: BG; locality: Brodilovo Vill, along Eleshnitsa River; verbatimElevation: 17; verbatimCoordinates: N42°05'35.4", E27°49'53.0"; geodeticDatum: WGS84; **Event:** eventDate: 04/07/2009
- **Type status:** Other material. **Occurrence:** recordedBy: R. Kostova; individualCount: 1; **Location:** countryCode: BG; locality: Malko Tarnovo surroundings; verbatimElevation: 375; verbatimCoordinates: N41°59'01.7", E27°29'49.4"; geodeticDatum: WGS84; **Event:** eventDate: 27/05/2010
- **Type status:** Other material. **Location:** countryCode: BG; locality: Kiten; **Record Level:** bibliographicCitation: Hieke & Wrase (1988: 53)
- **Type status:** Other material. **Location:** countryCode: BG; locality: Tsarevo (= Micurin); **Record Level:** bibliographicCitation: Hieke & Wrase (1988: 53)
- **Type status:** Other material. **Location:** countryCode: BG; locality: 5 km West of Yasna Polyana Vill.; **Record Level:** bibliographicCitation: Hieke & Wrase (1988: 53)
- **Type status:** Other material. **Occurrence:** recordedBy: B. Guéorguiev; individualCount: 3; **Location:** countryCode: BG; locality: Tisovitsa Reserve near Bulgari Vill.; **Event:** eventDate: 27/05/1995; **Record Level:** institutionCode: NMNHS
- **Type status:** Other material. **Occurrence:** recordedBy: B. Guéorguiev; individualCount: 1; **Location:** countryCode: BG; locality: Arapya, near Tsarevo; **Event:** eventDate: 28/05/1995; **Record Level:** institutionCode: NMNHS
- **Type status:** Other material. **Occurrence:** recordedBy: B. Guéorguiev; individualCount: 1; **Location:** countryCode: BG; locality: Ahtopol; **Event:** eventDate: 28/05/1995; **Record Level:** institutionCode: NMNHS
- **Type status:** Other material. **Occurrence:** recordedBy: B. Guéorguiev; individualCount: 1; **Location:** countryCode: BG; locality: Nature Park Ropotamo; **Event:** eventDate: 29/05/1995; **Record Level:** institutionCode: NMNHS

#### Bembidion (Peryphus) bualei

Jacquelin du Val, 1852

##### Materials

- **Type status:** Other material. **Occurrence:** recordedBy: R. Kostova; individualCount: 1; **Location:** countryCode: BG; locality: Slivarovo Vill., "Shafariitsa" Place; verbatimElevation: 224; verbatimCoordinates: N41°57'37.8", E27°39'34.8"; geodeticDatum: WGS84; **Event:** eventDate: 07/05/2009
- **Type status:** Other material. **Location:** countryCode: BG; locality: Tsarevo (= Micurin); **Record Level:** bibliographicCitation: Hieke & Wrase (1988: 51, as B. andreae bualei)

#### Bembidion (Peryphus) subcostatum vau

Netolitzky, 1913

##### Materials

- **Type status:** Other material. **Location:** countryCode: BG; locality: Kiten; **Record Level:** bibliographicCitation: Hieke & Wrase (1988: 52)
- **Type status:** Other material. **Location:** countryCode: BG; locality: Tsarevo (= Micurin); **Record Level:** bibliographicCitation: Hieke & Wrase (1988: 52)
- **Type status:** Other material. **Occurrence:** recordedBy: R. Bekchiev; individualCount: 1; **Location:** countryCode: BG; locality: Sinemorets Vill., PA "Estuary of Veleka river"; verbatimElevation: 6; verbatimCoordinates: N42°03'39.6", E27°57'55.2"; geodeticDatum: WGS84; **Event:** eventDate: 18/04/2009
- **Type status:** Other material. **Occurrence:** recordedBy: R. Kostova; individualCount: 8; **Location:** countryCode: BG; locality: Stoilovo Vill., "Sredoka" Reserve, along Mechi dol River; verbatimElevation: 206; verbatimCoordinates: N42°01'51.1", E27°30'50.1"; geodeticDatum: WGS84; **Event:** eventDate: 19/04/2009
- **Type status:** Other material. **Occurrence:** recordedBy: R. Kostova; individualCount: 2; **Location:** countryCode: BG; locality: Brodilovo Vill, along Eleshnitsa River; verbatimElevation: 17; verbatimCoordinates: N42°05'35.4", E27°49'53.0"; geodeticDatum: WGS84; **Event:** eventDate: 04/07/2009
- **Type status:** Other material. **Occurrence:** recordedBy: R. Kostova; individualCount: 1; **Location:** countryCode: TR; locality: Along Demirköy - Igneada road; verbatimElevation: 12; verbatimCoordinates: N41°52'39.3", E27°56'29.2"; geodeticDatum: WGS84; **Event:** eventDate: 05/07/2009
- **Type status:** Other material. **Occurrence:** recordedBy: R. Kostova; individualCount: 1; **Location:** countryCode: TR; locality: Demirköy - along Degirmen river; verbatimElevation: 289; verbatimCoordinates: N41°49'17.2", E27°45'07.7"; geodeticDatum: WGS84; **Event:** eventDate: 06/07/2009
- **Type status:** Other material. **Occurrence:** recordedBy: R. Kostova; individualCount: 1; **Location:** countryCode: TR; locality: Hamam golu; verbatimElevation: 11; verbatimCoordinates: N41°49'31.6", E27°57'33.8"; geodeticDatum: WGS84; **Event:** eventDate: 25/05/2010
- **Type status:** Other material. **Occurrence:** recordedBy: R. Kostova; individualCount: 4; **Location:** countryCode: BG; locality: Malko Tarnovo, Entrance to Vitanovo Reserve; verbatimElevation: 285; verbatimCoordinates: N42°01'22.9", E27°27'22.9"; geodeticDatum: WGS84; **Event:** eventDate: 27/05/2010
- **Type status:** Other material. **Occurrence:** recordedBy: R. Bekchiev; individualCount: 1; **Location:** countryCode: TR; locality: Kiyiköy surroundings; verbatimElevation: 32; verbatimCoordinates: N41°38'12.8", E28°04'08.7"; geodeticDatum: WGS84; **Event:** eventDate: 22/05/2011
- **Type status:** Other material. **Occurrence:** recordedBy: R. Kostova; individualCount: 2; **Location:** countryCode: TR; locality: Kizilagač, along river; verbatimElevation: 115; verbatimCoordinates: N41°41'01.9", E27°52'51.4"; geodeticDatum: WGS84; **Event:** eventDate: 24/05/2011
- **Type status:** Other material. **Occurrence:** recordedBy: R. Kostova; individualCount: 10; **Location:** countryCode: BG; locality: Kosti Vill., "St. Ilia" Place; verbatimElevation: 35; verbatimCoordinates: N42°03'23.2", E27°45'51.6"; geodeticDatum: WGS84; **Event:** eventDate: 15.04-08.06.2009; habitat: meadow with single trees
- **Type status:** Other material. **Occurrence:** recordedBy: B. Guéorguiev; individualCount: 2; **Location:** countryCode: BG; locality: Veleka River near Mladezko Village; **Event:** eventDate: 25/05/1995; **Record Level:** institutionCode: NMNHS
- **Type status:** Other material. **Occurrence:** recordedBy: B. Guéorguiev; individualCount: 2; **Location:** countryCode: BG; locality: Rezovska River at Uzunbudzhak Reserve; **Event:** eventDate: 26/05/1995; **Record Level:** institutionCode: NMNHS

#### Bembidion (Philochthus) escherichi escherichi

Ganglbauer, 1897

##### Materials

- **Type status:** Other material. **Occurrence:** recordedBy: R. Kostova; individualCount: 1; **Location:** countryCode: BG; locality: Slivarovo Vill., "Shafariitsa" Place; verbatimElevation: 224; verbatimCoordinates: N41°57'37.8", E27°39'34.8"; geodeticDatum: WGS84; **Event:** eventDate: 07/05/2009

#### Bembidion (Philochthus) guttula

(Fabricius, 1792)

##### Materials

- **Type status:** Other material. **Occurrence:** recordedBy: R. Kostova; individualCount: 1; **Location:** countryCode: TR; locality: Along Demirköy - Igneada road; verbatimElevation: 12; verbatimCoordinates: N41°52'39.3", E27°56'29.2"; geodeticDatum: WGS84; **Event:** eventDate: 05/07/2009
- **Type status:** Other material. **Occurrence:** recordedBy: R. Kostova; individualCount: 1; **Location:** countryCode: TR; locality: Sislioba Vill. surroundings; verbatimElevation: 52; verbatimCoordinates: N41°57'43.3", E27°54'35.2"; geodeticDatum: WGS84; **Event:** eventDate: 08/07/2009

#### Bembidion (Philochthus) guttuloides

De Monte, 1953

##### Materials

- **Type status:** Other material. **Occurrence:** recordedBy: R. Kostova; individualCount: 2; **Location:** countryCode: TR; locality: Along Demirköy - Igneada road; verbatimElevation: 30; verbatimCoordinates: N41°52'41.1", E27°54'27.0"; geodeticDatum: WGS84; **Event:** eventDate: 05/07/2009

#### Bembidion (Philochthus) inoptatum

Schaum, 1857

##### Materials

- **Type status:** Other material. **Occurrence:** recordedBy: R. Kostova; individualCount: 1; **Location:** countryCode: BG; locality: Sinemorets Vill., PA "Estuary of Veleka river"; verbatimElevation: 6; verbatimCoordinates: N42°03'39.6", E27°57'55.2"; geodeticDatum: WGS84; **Event:** eventDate: 16/04/2009; habitat: forest
- **Type status:** Other material. **Occurrence:** recordedBy: R. Bekchiev; individualCount: 1; **Location:** countryCode: BG; locality: Sinemorets Vill., PA "Estuary of Veleka river"; verbatimElevation: 6; verbatimCoordinates: N42°03'39.6", E27°57'55.2"; geodeticDatum: WGS84; **Event:** eventDate: 01/07/2009; habitat: forest
- **Type status:** Other material. **Occurrence:** recordedBy: R. Bekchiev; individualCount: 1; **Location:** countryCode: BG; locality: Sinemorets Vill., PA "Estuary of Veleka river"; verbatimElevation: 5; verbatimCoordinates: N42°03'39.6", E27°57'55.2"; geodeticDatum: WGS84; **Event:** eventDate: 02/07/2009; habitat: meadow
- **Type status:** Other material. **Occurrence:** recordedBy: R. Kostova; individualCount: 1; **Location:** countryCode: BG; locality: Mladezhko Vill., The springs of Mladezhka River; verbatimElevation: 231; verbatimCoordinates: N42°09'04.5", E27°21'26.1"; geodeticDatum: WGS84; **Event:** eventDate: 17/04/2009; habitat: black alder and hornbeam forest
- **Type status:** Other material. **Location:** countryCode: BG; locality: Tsarevo (= Micurin); **Record Level:** bibliographicCitation: Hieke & Wrase (1988: 62)
- **Type status:** Other material. **Occurrence:** recordedBy: E. Migliaccio; individualCount: 1; **Location:** countryCode: BG; locality: Veleka Valley, Kachul Place; verbatimElevation: 80; **Event:** eventDate: 30.06-26.08.2004; **Record Level:** institutionCode: NMNHS

#### Bembidion (Philochthus) lunulatum

(Geoffroy in Fourcroy, 1785)

##### Materials

- **Type status:** Other material. **Occurrence:** recordedBy: R. Kostova; individualCount: 2; **Location:** countryCode: BG; locality: Sinemorets Vill., PA "Estuary of Veleka river"; verbatimElevation: 6; verbatimCoordinates: N42°03'39.6", E27°57'55.2"; geodeticDatum: WGS84; **Event:** eventDate: 15.04-08.05.2009; habitat: swamp forest
- **Type status:** Other material. **Location:** countryCode: BG; locality: Tsarevo (= Micurin); **Record Level:** bibliographicCitation: Hieke & Wrase (1988: 62)

#### Bembidion (Phyla) tethys

Netolitzky, 1926

##### Materials

- **Type status:** Other material. **Occurrence:** recordedBy: R. Kostova; individualCount: 1; **Location:** countryCode: BG; locality: Kosti Vill., "St. Ilia" Place; verbatimElevation: 35; verbatimCoordinates: N42°03'23.2", E27°45'51.6"; geodeticDatum: WGS84; **Event:** eventDate: 09.06-02.07.2009; habitat: meadow with single trees
- **Type status:** Other material. **Occurrence:** recordedBy: R. Kostova; individualCount: 1; **Location:** countryCode: BG; locality: Sinemorets Vill., PA"Silistar", "Butamyata" Place; verbatimElevation: 10; verbatimCoordinates: N42°03'11.2", E27°59'19.7"; geodeticDatum: WGS84; **Event:** eventDate: 15.04-08.05.2009; habitat: meadow, single shrubs
- **Type status:** Other material. **Occurrence:** recordedBy: R. Kostova; individualCount: 1; **Location:** countryCode: BG; locality: Sinemorets Vill., PA"Silistar", "Butamyata" Place; verbatimElevation: 10; verbatimCoordinates: N42°03'11.2", E27°59'19.7"; geodeticDatum: WGS84; **Event:** eventDate: 03.07-02.08.2009; habitat: meadow, single shrubs

#### Bembidion (Princidium) punctulatum punctulatum

Drapiez, 1820

##### Materials

- **Type status:** Other material. **Location:** countryCode: BG; locality: Tsarevo (= Micurin); **Record Level:** bibliographicCitation: Hieke & Wrase (1988: 45)

#### Bembidion (Talanes) subfaciatum

Chaudoir, 1850

##### Materials

- **Type status:** Other material. **Occurrence:** recordedBy: R. Bekchiev; individualCount: 1; **Location:** countryCode: BG; locality: Primorsko, "Arcutino" Place; verbatimElevation: 60; verbatimCoordinates: N42°20'16.0", E27°43'18.1"; geodeticDatum: WGS84; **Event:** eventDate: 30/06/2009
- **Type status:** Other material. **Occurrence:** recordedBy: R. Bekchiev; individualCount: 2; **Location:** countryCode: TR; locality: Igneada surroundings; verbatimElevation: 3; verbatimCoordinates: N41°51'27.3", E27°57'28.7"; geodeticDatum: WGS84; **Event:** eventDate: 29/09/2009; habitat: marsh
- **Type status:** Other material. **Occurrence:** recordedBy: R. Kostova; individualCount: 3; **Location:** countryCode: TR; locality: Igneada surroundings; verbatimElevation: 3; verbatimCoordinates: N41°51'27.3", E27°57'28.7"; geodeticDatum: WGS84; **Event:** eventDate: 02/10/2009; habitat: marsh
- **Type status:** Other material. **Occurrence:** recordedBy: R. Kostova; individualCount: 1; **Location:** countryCode: TR; locality: Igneada; verbatimElevation: 3; verbatimCoordinates: N41°52'51.3", E27°59'25.5"; geodeticDatum: WGS84; **Event:** eventDate: 02/10/2009

#### Bembidion (Trepanes) articulatum

(Panzer, 1796)

##### Materials

- **Type status:** Other material. **Occurrence:** recordedBy: R. Kostova; individualCount: 1; **Location:** countryCode: TR; locality: Along Demirköy - Igneada road; verbatimElevation: 12; verbatimCoordinates: N41°52'39.3", E27°56'29.2"; geodeticDatum: WGS84; **Event:** eventDate: 05/07/2009
- **Type status:** Other material. **Location:** countryCode: BG; locality: Tsarevo (= Micurin); **Record Level:** bibliographicCitation: Hieke & Wrase (1988: 60)
- **Type status:** Other material. **Location:** countryCode: BG; locality: 5 km West of Yasna Polyana Vill.; **Record Level:** bibliographicCitation: Hieke & Wrase (1988: 60)
- **Type status:** Other material. **Occurrence:** recordedBy: P. Beron; individualCount: 1; **Location:** countryCode: BG; locality: Kiten; **Event:** eventDate: 16-22.12.1984; **Record Level:** institutionCode: NMNHS

#### Bembidion (Trepanes) octomaculatum

(Goeze, 1777)

##### Materials

- **Type status:** Other material. **Occurrence:** recordedBy: R. Bekchiev; individualCount: 1; **Location:** countryCode: BG; locality: Sinemorets Vill., PA "Estuary of Veleka river"; verbatimElevation: 5; verbatimCoordinates: N42°03'39.6", E27°57'55.2"; geodeticDatum: WGS84; **Event:** eventDate: 02/07/2009; habitat: meadow
- **Type status:** Other material. **Occurrence:** recordedBy: R. Kostova & R. Bekchiev; individualCount: 1; **Location:** countryCode: TR; locality: Igneada surroundings; verbatimElevation: 3; verbatimCoordinates: N41°51'27.3", E27°57'28.7"; geodeticDatum: WGS84; **Event:** eventDate: 29/09/2009; habitat: marsh
- **Type status:** Other material. **Occurrence:** recordedBy: R. Kostova & R. Bekchiev; individualCount: 1; **Location:** countryCode: TR; locality: Igneada surroundings; verbatimElevation: 3; verbatimCoordinates: N41°51'27.3", E27°57'28.7"; geodeticDatum: WGS84; **Event:** eventDate: 02/10/2009; habitat: marsh
- **Type status:** Other material. **Location:** countryCode: BG; locality: Tsarevo (= Micurin); **Record Level:** bibliographicCitation: Hieke & Wrase (1988: 61)
- **Type status:** Other material. **Occurrence:** recordedBy: B. Guéorguiev; individualCount: 1; **Location:** countryCode: BG; locality: Rezovska River at Uzunbudzhak Reserve; **Event:** eventDate: 26/05/1995; **Record Level:** institutionCode: NMNHS

#### Ocys harpaloides

(Audinet-Serville, 1821)

##### Materials

- **Type status:** Other material. **Location:** countryCode: BG; locality: Rezovska River at Uzunbudzhak Reserve; **Record Level:** bibliographicCitation: Guéorguiev & Muilwijk (2000: 81)
- **Type status:** Other material. **Location:** countryCode: BG; locality: Ahtopol; **Record Level:** bibliographicCitation: Guéorguiev & Muilwijk (2000: 81)

#### Sinechostictus (Sinechostictus) tarsicus

(Peyron, 1858)

##### Materials

- **Type status:** Other material. **Occurrence:** recordedBy: R. Kostova; individualCount: 3; **Location:** countryCode: BG; locality: Stoilovo Vill., "Sredoka" Reserve, along Mechi dol River; verbatimElevation: 206; verbatimCoordinates: N42°01'51.1", E27°30'50.1"; geodeticDatum: WGS84; **Event:** eventDate: 19/04/2009
- **Type status:** Other material. **Occurrence:** recordedBy: R. Kostova; individualCount: 4; **Location:** countryCode: BG; locality: Stoilovo Vill., "Sredoka" Reserve, along Mechi dol River; verbatimElevation: 206; verbatimCoordinates: N42°01'51.1", E27°30'50.1"; geodeticDatum: WGS84; **Event:** eventDate: 19/04/2009
- **Type status:** Other material. **Occurrence:** recordedBy: R. Kostova; individualCount: 1; **Location:** countryCode: BG; locality: Kirovo Vill., along Selska River; verbatimElevation: 138; verbatimCoordinates: N42°10'43.0", E27°10'58.2"; geodeticDatum: WGS84; **Event:** eventDate: 05/05/2009
- **Type status:** Other material. **Occurrence:** recordedBy: R. Kostova; individualCount: 2; **Location:** countryCode: BG; locality: Varovnik Vill., near the bridge at Fakiiska River; verbatimElevation: 125; verbatimCoordinates: N42°13'33.7", E27°11'33.7"; geodeticDatum: WGS84; **Event:** eventDate: 09/06/2009
- **Type status:** Other material. **Occurrence:** recordedBy: R. Kostova; individualCount: 1; **Location:** countryCode: BG; locality: Brodilovo Vill, along Eleshnitsa River; verbatimElevation: 17; verbatimCoordinates: N42°05'35.4", E27°49'53.0"; geodeticDatum: WGS84; **Event:** eventDate: 05/07/2009
- **Type status:** Other material. **Occurrence:** recordedBy: R. Kostova; individualCount: 1; **Location:** countryCode: BG; locality: Brodilovo Vill, along Eleshnitsa River; verbatimElevation: 17; verbatimCoordinates: N42°05'35.4", E27°49'53.0"; geodeticDatum: WGS84; **Event:** eventDate: 04/07/2009
- **Type status:** Other material. **Occurrence:** recordedBy: R. Kostova; individualCount: 6; **Location:** countryCode: BG; locality: Malko Tarnovo, Entrance to Vitanovo Reserve; verbatimElevation: 285; verbatimCoordinates: N42°01'22.9", E27°27'22.9"; geodeticDatum: WGS84; **Event:** eventDate: 27/05/2010
- **Type status:** Other material. **Occurrence:** recordedBy: A. Gijonova; individualCount: 1; **Location:** countryCode: TR; locality: Kizilagač, along river; verbatimElevation: 115; verbatimCoordinates: N41°41'01.9", E27°52'51.4"; geodeticDatum: WGS84; **Event:** eventDate: 24/05/2011
- **Type status:** Other material. **Occurrence:** recordedBy: R. Kostova; individualCount: 1; **Location:** countryCode: BG; locality: Kosti Vill., "St. Ilia" Place; verbatimElevation: 35; verbatimCoordinates: N42°03'23.2", E27°45'51.6"; geodeticDatum: WGS84; **Event:** eventDate: 09.05-08.06.2009; habitat: meadow with single trees
- **Type status:** Other material. **Location:** countryCode: BG; locality: Tsarevo (= Micurin); **Record Level:** bibliographicCitation: Hieke & Wrase (1988: 56)
- **Type status:** Other material. **Location:** countryCode: BG; locality: Ropotamo; **Record Level:** bibliographicCitation: Hieke & Wrase (1988: 56)
- **Type status:** Other material. **Location:** countryCode: BG; locality: 5 km West of Yasna Polyana Vill.; **Record Level:** bibliographicCitation: Hieke & Wrase (1988: 56)
- **Type status:** Other material. **Location:** countryCode: BG; locality: Gramatikovo Vill.; **Record Level:** bibliographicCitation: Guéorguiev (2011: 519)
- **Type status:** Other material. **Location:** countryCode: BG; locality: Gramatikovo Vill.; **Record Level:** bibliographicCitation: Guéorguiev (2011: 519)
- **Type status:** Other material. **Occurrence:** recordedBy: B. Guéorguiev; individualCount: 4; **Location:** countryCode: BG; locality: Veleka River near Mladezko Village; **Event:** eventDate: 25/05/1995; **Record Level:** institutionCode: NMNHS
- **Type status:** Other material. **Occurrence:** recordedBy: B. Guéorguiev; individualCount: 4; **Location:** countryCode: BG; locality: Rezovska River at Uzunbudzhak Reserve; **Event:** eventDate: 26/05/1995; **Record Level:** institutionCode: NMNHS

#### Paratachys bistriatus

(Duftschmid, 1812)

##### Materials

- **Type status:** Other material. **Occurrence:** recordedBy: R. Kostova; individualCount: 5; **Location:** countryCode: BG; locality: Kirovo Vill., along Selska River; verbatimElevation: 138; verbatimCoordinates: N42°10'43.0", E27°10'58.2"; geodeticDatum: WGS84; **Event:** eventDate: 05/05/2009
- **Type status:** Other material. **Occurrence:** recordedBy: R. Kostova; individualCount: 1; **Location:** countryCode: BG; locality: Sinemorets Vill., PA"Silistar", Estuary of Silistar River; verbatimElevation: 12; verbatimCoordinates: N42°01'26.8", E28°00'32.9"; geodeticDatum: WGS84; **Event:** eventDate: 09/05/2009
- **Type status:** Other material. **Occurrence:** recordedBy: R. Kostova; individualCount: 1; **Location:** countryCode: BG; locality: Rezovo Vill. surroundings; verbatimElevation: 5; verbatimCoordinates: N41°58'54.4", E28°01'29.6"; geodeticDatum: WGS84; **Event:** eventDate: 09/05/2009; habitat: meadows
- **Type status:** Other material. **Occurrence:** recordedBy: R. Kostova; individualCount: 2; **Location:** countryCode: BG; locality: Sinemorets Vill., PA"Silistar", "Butamyata" Place; verbatimElevation: 17; verbatimCoordinates: N42°03'10.3", E27°59'13.4"; geodeticDatum: WGS84; **Event:** eventDate: 13/06/2009; habitat: oak forest
- **Type status:** Other material. **Occurrence:** recordedBy: R. Bekchiev; individualCount: 4; **Location:** countryCode: BG; locality: Primorsko, Perla Beach; verbatimElevation: 1; verbatimCoordinates: N42°17'10.2", E27°45'13.6"; geodeticDatum: WGS84; **Event:** eventDate: 30/06/2009
- **Type status:** Other material. **Occurrence:** recordedBy: R. Bekchiev; individualCount: 1; **Location:** countryCode: BG; locality: Primorsko, "Arcutino" Place; verbatimElevation: 60; verbatimCoordinates: N42°20'16.0", E27°43'18.1"; geodeticDatum: WGS84; **Event:** eventDate: 30/06/2009
- **Type status:** Other material. **Occurrence:** recordedBy: R. Bekchiev; individualCount: 1; **Location:** countryCode: BG; locality: Varvara Vill. surroundings; verbatimElevation: 46; verbatimCoordinates: N42°06'49.1", E27°53'18.9"; geodeticDatum: WGS84; **Event:** eventDate: 02/07/2009
- **Type status:** Other material. **Occurrence:** recordedBy: R. Bekchiev; individualCount: 1; **Location:** countryCode: TR; locality: Along Demirköy - Igneada road; verbatimElevation: 12; verbatimCoordinates: N41°52'39.3", E27°56'29.2"; geodeticDatum: WGS84; **Event:** eventDate: 05/07/2009
- **Type status:** Other material. **Occurrence:** recordedBy: R. Bekchiev; individualCount: 5; **Location:** countryCode: TR; locality: Igneada, Hamam Gölü; verbatimElevation: 11; verbatimCoordinates: N41°49'31.6", E27°57'33.8"; geodeticDatum: WGS84; **Event:** eventDate: 02/10/2009
- **Type status:** Other material. **Occurrence:** recordedBy: I. Gijonov; individualCount: 1; **Location:** countryCode: BG; locality: Sinemorets Vill., PA"Silistar", "Lipite" Beach; verbatimElevation: 54; verbatimCoordinates: N42°02'40.2", E27°59'21.8"; geodeticDatum: WGS84; **Event:** eventDate: 29/05/2010
- **Type status:** Other material. **Occurrence:** recordedBy: R. Kostova; individualCount: 1; **Location:** countryCode: TR; locality: Yalikŏy, river estuary; verbatimElevation: 9; verbatimCoordinates: N41°29'27.7", E28°16'40.0"; geodeticDatum: WGS84; **Event:** eventDate: 23/05/2011
- **Type status:** Other material. **Occurrence:** recordedBy: R. Kostova; individualCount: 1; **Location:** countryCode: TR; locality: Kizilagač, along river; verbatimElevation: 115; verbatimCoordinates: N41°41'01.9", E27°52'51.4"; geodeticDatum: WGS84; **Event:** eventDate: 24/05/2011
- **Type status:** Other material. **Occurrence:** recordedBy: R. Bekchiev; individualCount: 2; **Location:** countryCode: BG; locality: Sinemorets Vill., PA"Silistar", Estuary of Silistar River; verbatimElevation: 12; verbatimCoordinates: N42°01'26.8", E28°00'32.9"; geodeticDatum: WGS84; **Event:** eventDate: 27/05/2011
- **Type status:** Other material. **Occurrence:** recordedBy: R. Kostova & R. Bekchiev; individualCount: 7; **Location:** countryCode: BG; locality: Sinemorets Vill., PA "Estuary of Veleka river"; verbatimElevation: 6; verbatimCoordinates: N42°03'39.6", E27°57'55.2"; geodeticDatum: WGS84; **Event:** eventDate: 15.04-02.07.2009; habitat: swamp forest
- **Type status:** Other material. **Location:** countryCode: BG; locality: Kiten; **Record Level:** bibliographicCitation: Hieke & Wrase (1988: 63, as Tachys bistriatus)
- **Type status:** Other material. **Location:** countryCode: BG; locality: Tsarevo (= Micurin); **Record Level:** bibliographicCitation: Hieke & Wrase (1988: 63, as Tachys bistriatus)
- **Type status:** Other material. **Location:** countryCode: BG; locality: Ropotamo; **Record Level:** bibliographicCitation: Hieke & Wrase (1988: 63, as Tachys bistriatus)
- **Type status:** Other material. **Location:** countryCode: BG; locality: Yasna polyana Vill., 5 km west; **Record Level:** bibliographicCitation: Hieke & Wrase (1988: 63, as Tachys bistriatus)

#### Paratachys fulvicollis

(Dejean, 1831)

##### Materials

- **Type status:** Other material. **Occurrence:** recordedBy: R. Kostova; individualCount: 2; **Location:** countryCode: TR; locality: Igneada surroundings; verbatimElevation: 3; verbatimCoordinates: N41°51'27.4", E27°57'32.2"; geodeticDatum: WGS84; **Event:** eventDate: 05/07/2009; habitat: marsh
- **Type status:** Other material. **Occurrence:** recordedBy: R. Kostova; individualCount: 2; **Location:** countryCode: TR; locality: Igneada, Hamam Gölü; verbatimElevation: 5; verbatimCoordinates: N41°49'43.2", E27°57'31.1"; geodeticDatum: WGS84; **Event:** eventDate: 02/10/2009

#### Porotachys ottomanus ottomanus

Schweiger, 1968

##### Materials

- **Type status:** Other material. **Occurrence:** recordedBy: R. Bekchiev; individualCount: 3; **Location:** countryCode: BG; locality: Brodilovo Vill., under Papiya peak; verbatimElevation: 336; verbatimCoordinates: N42°06'23.7", E27°50'36.1"; geodeticDatum: WGS84; **Event:** eventDate: 16/04/2009; habitat: oak forest
- **Type status:** Other material. **Occurrence:** recordedBy: R. Bekchiev; individualCount: 1; **Location:** countryCode: BG; locality: Malko Tarnovo, "Indipasqua" Place; verbatimElevation: 211; verbatimCoordinates: N42°00'16.9", E27°39'09.2"; geodeticDatum: WGS84; **Event:** eventDate: 25/05/2009
- **Type status:** Other material. **Occurrence:** recordedBy: R. Bekchiev; individualCount: 2; **Location:** countryCode: BG; locality: Stoilovo Vill., "Sredoka" Reserve, along Mechi dol River; verbatimElevation: 206; verbatimCoordinates: N42°01'51.1", E27°30'50.1"; geodeticDatum: WGS84; **Event:** eventDate: 10/06/2009
- **Type status:** Other material. **Occurrence:** recordedBy: R. Bekchiev; individualCount: 9; **Location:** countryCode: BG; locality: Varvara Vill. surroundings; verbatimElevation: 46; verbatimCoordinates: N42°06'49.1", E27°53'18.9"; geodeticDatum: WGS84; **Event:** eventDate: 26/08/2009
- **Type status:** Other material. **Occurrence:** recordedBy: R. Bekchiev; individualCount: 5; **Location:** countryCode: BG; locality: Fazanovo Vill., "Popovi skali" Place; verbatimElevation: 26; verbatimCoordinates: N42°09'45.9", E27°44'15.6"; geodeticDatum: WGS84; **Event:** eventDate: 01/07/2009
- **Type status:** Other material. **Occurrence:** recordedBy: R. Bekchiev; individualCount: 2; **Location:** countryCode: BG; locality: Bulgari Vill., PA "Marina reka"; verbatimElevation: 183; verbatimCoordinates: N42°06'41.7", E27°45'53.0"; geodeticDatum: WGS84; **Event:** eventDate: 11/06/2009
- **Type status:** Other material. **Occurrence:** recordedBy: R. Kostova; individualCount: 1; **Location:** countryCode: BG; locality: Bulgari Vill., PA "Marina reka"; verbatimElevation: 183; verbatimCoordinates: N42°06'41.7", E27°45'53.0"; geodeticDatum: WGS84; **Event:** eventDate: 03/07/2009
- **Type status:** Other material. **Occurrence:** recordedBy: R. Bekchiev; individualCount: 10; **Location:** countryCode: BG; locality: Brodilovo Vill.; verbatimElevation: 13; verbatimCoordinates: N42°05'18.0", E27°51'06.2"; geodeticDatum: WGS84; **Event:** eventDate: 04/07/2009
- **Type status:** Other material. **Occurrence:** recordedBy: R. Kostova; individualCount: 1; **Location:** countryCode: TR; locality: Demirköy, along Degirmen River; verbatimElevation: 289; verbatimCoordinates: N41°49'17.2", E27°45'07.7"; geodeticDatum: WGS84; **Event:** eventDate: 06/07/2009
- **Type status:** Other material. **Occurrence:** recordedBy: R. Kostova; individualCount: 1; **Location:** countryCode: TR; locality: Demirköy, Dökümhanesi; verbatimElevation: 179; verbatimCoordinates: N41°49'09.6", E27°48'43.2"; geodeticDatum: WGS84; **Event:** eventDate: 03/10/2009
- **Type status:** Other material. **Occurrence:** recordedBy: R. Bekchiev; individualCount: 1; **Location:** countryCode: TR; locality: Kizilagač, along river; verbatimElevation: 115; verbatimCoordinates: N41°41'01.9", E27°52'51.4"; geodeticDatum: WGS84; **Event:** eventDate: 24/05/2011
- **Type status:** Other material. **Occurrence:** recordedBy: J. Hlisnikovský; individualCount: 4; **Location:** countryCode: BG; locality: Malko Tarnovo; **Event:** eventDate: 17/05/1939; **Record Level:** institutionCode: NMP (det. as Tachys bisulcatus)
- **Type status:** Other material. **Occurrence:** recordedBy: A. Pfeffer; individualCount: 1; **Location:** countryCode: BG; locality: Strandzha; **Event:** eventDate: VII.1934; **Record Level:** institutionCode: NMP (det. as Tachys bisulcatus)
- **Type status:** Other material. **Location:** countryCode: BG; locality: Yasna polyana Vill., 5 km west; **Record Level:** bibliographicCitation: Hieke & Wrase (1988: 65, as P. bisulcatus)

#### Sphaerotachys hoemorroidalis

(Ponza, 1805)

##### Materials

- **Type status:** Other material. **Occurrence:** recordedBy: R. Kostova; individualCount: 1; **Location:** countryCode: BG; locality: Primorsko, Dunes; verbatimElevation: 6; verbatimCoordinates: N41°47'44.0", E27°59'50.4"; geodeticDatum: WGS84; **Event:** eventDate: 10/05/2009
- **Type status:** Other material. **Occurrence:** recordedBy: R. Kostova; individualCount: 1; **Location:** countryCode: TR; locality: Igneada surroundings; verbatimElevation: 3; verbatimCoordinates: N41°51'27.4", E27°57'32.2"; geodeticDatum: WGS84; **Event:** eventDate: 05/07/2009; habitat: marsh
- **Type status:** Other material. **Occurrence:** recordedBy: R. Kostova; individualCount: 1; **Location:** countryCode: TR; locality: Kiyiköy surroundings; verbatimElevation: 62; verbatimCoordinates: N41°37'40.7", E28°04'05.0"; geodeticDatum: WGS84; **Event:** eventDate: 22/05/2011
- **Type status:** Other material. **Occurrence:** recordedBy: R. Kostova; individualCount: 1; **Location:** countryCode: TR; locality: Kiyiköy surroundings; verbatimElevation: 38; verbatimCoordinates: N41°39'25.7", E28°05'11.2"; geodeticDatum: WGS84; **Event:** eventDate: 22/05/2011
- **Type status:** Other material. **Occurrence:** recordedBy: R. Kostova; individualCount: 1; **Location:** countryCode: TR; locality: Yalikŏy, river estuary; verbatimElevation: 9; verbatimCoordinates: N41°29'27.7", E28°16'40.0"; geodeticDatum: WGS84; **Event:** eventDate: 23/05/2011
- **Type status:** Other material. **Occurrence:** recordedBy: R. Kostova; individualCount: 1; **Location:** countryCode: TR; locality: Kizilagač, along river; verbatimElevation: 115; verbatimCoordinates: N41°41'01.9", E27°52'51.4"; geodeticDatum: WGS84; **Event:** eventDate: 24/05/2011
- **Type status:** Other material. **Location:** countryCode: BG; locality: Tsarevo (= Micurin); **Record Level:** bibliographicCitation: Hieke & Wrase (1988: 65, as Elaphropus h.)

#### Tachys scutellaris

Stephens, 1828

##### Materials

- **Type status:** Other material. **Occurrence:** recordedBy: J. Mařan & K. Taborsky; individualCount: 1; **Location:** countryCode: BG; locality: Maslen nos Cape; **Record Level:** institutionCode: NMP

#### Tachyta (Tachyta) nana nana

(Gyllenhal, 1810)

##### Materials

- **Type status:** Other material. **Occurrence:** recordedBy: R. Kostova; individualCount: 1; **Location:** countryCode: BG; locality: Mladezhko Vill., The springs of Mladezhka River; verbatimElevation: 231; verbatimCoordinates: N42°09'04.5", E27°21'26.1"; geodeticDatum: WGS84; **Event:** eventDate: 21/08/2009; habitat: black alder and hornbeam forest
- **Type status:** Other material. **Location:** countryCode: BG; locality: Bosna Ridge; **Record Level:** bibliographicCitation: Guéorguiev (1992: 64, as Tachys nana)
- **Type status:** Other material. **Location:** countryCode: BG; locality: Brodilovo Vill., Papiya Peak; **Record Level:** bibliographicCitation: Guéorguiev (1992: 63, as Tachys nana)

#### Tachyura (Tachyura) diabrachys

(Kolenenati, 1845)

##### Materials

- **Type status:** Other material. **Occurrence:** recordedBy: R. Kostova; individualCount: 2; **Location:** countryCode: BG; locality: Sinemorets Vill., PA"Silistar", Estuary of Silistar River; verbatimElevation: 12; verbatimCoordinates: N42°01'26.8", E28°00'32.9"; geodeticDatum: WGS84; **Event:** eventDate: 27/05/2011
- **Type status:** Other material. **Occurrence:** recordedBy: R. Kostova; individualCount: 3; **Location:** countryCode: TR; locality: Yalikŏy, river estuary; verbatimElevation: 9; verbatimCoordinates: N41°29'27.7", E28°16'40.0"; geodeticDatum: WGS84; **Event:** eventDate: 23/05/2011
- **Type status:** Other material. **Location:** countryCode: BG; locality: Ahtopol; **Record Level:** bibliographicCitation: Hieke & Wrase (1988: 65, as Elaphropus d. bisbimaculatus)
- **Type status:** Other material. **Location:** countryCode: BG; locality: Yasna polyana Vill., 5 km west; **Record Level:** bibliographicCitation: Hieke & Wrase (1988: 65, as Elaphropus d. bisbimaculatus)

#### Tachyura (Tachyura) parvula

(Dejean, 1831)

##### Materials

- **Type status:** Other material. **Occurrence:** recordedBy: R. Kostova; individualCount: 1; **Location:** countryCode: BG; locality: Varvara Vill. surroundings; verbatimElevation: 46; verbatimCoordinates: N42°06'49.1", E27°53'18.9"; geodeticDatum: WGS84; **Event:** eventDate: 10/05/2009
- **Type status:** Other material. **Occurrence:** recordedBy: R. Kostova; individualCount: 1; **Location:** countryCode: TR; locality: Along Demirköy - Kıyıköy road; verbatimElevation: 199; verbatimCoordinates: N41°39'43.2", E27°55'25.8"; geodeticDatum: WGS84; **Event:** eventDate: 24/05/2010
- **Type status:** Other material. **Occurrence:** recordedBy: R. Kostova; individualCount: 1; **Location:** countryCode: TR; locality: Yalikŏy, river estuary; verbatimElevation: 9; verbatimCoordinates: N41°29'27.7", E28°16'40.0"; geodeticDatum: WGS84; **Event:** eventDate: 23/05/2011
- **Type status:** Other material. **Location:** countryCode: BG; locality: Tsarevo (= Micurin); **Record Level:** bibliographicCitation: Hieke & Wrase (1988: 64, as Elaphropus p.)
- **Type status:** Other material. **Location:** countryCode: BG; locality: Yasna polyana Vill., 5 km west; **Record Level:** bibliographicCitation: Hieke & Wrase (1988: 64, as Elaphropus p.)

#### Tachyura (Tachyura) quadrisignata

(Duftschmid, 1812)

##### Materials

- **Type status:** Other material. **Occurrence:** recordedBy: R. Kostova; individualCount: 3; **Location:** countryCode: TR; locality: Kizilagač, along river; verbatimElevation: 115; verbatimCoordinates: N41°41'01.9", E27°52'51.4"; geodeticDatum: WGS84; **Event:** eventDate: 24/05/2011

#### Cardiaderus chloroticus

(Fischer von Waldheim, 1823)

##### Materials

- **Type status:** Other material. **Location:** countryCode: BG; locality: Ropotamo; **Record Level:** bibliographicCitation: Guéorguiev (1992: 65, as Cardioderus! c.)
- **Type status:** Other material. **Location:** countryCode: BG; locality: Ropotamo; **Record Level:** bibliographicCitation: Guéorguiev & Guéorguiev (1995: 114, as Cardioderus chloroticus)

#### Pogonistes rufoaeneus

(Dejean, 1828)

##### Materials

- **Type status:** Other material. **Location:** countryCode: BG; locality: Maslen nos Cape; **Record Level:** bibliographicCitation: Guéorguiev (1992: 65, as Pogonus r.)

#### Pogonus (Pogonoidius) meridionalis

Dejean, 1828

##### Materials

- **Type status:** Other material. **Location:** countryCode: BG; locality: Tsarevo; **Record Level:** bibliographicCitation: Guéorguiev & Guéorguiev (1995: 116)

#### Pogonus (Pogonus) iridipennis

Nicolai, 1822

##### Materials

- **Type status:** Other material. **Location:** countryCode: BG; locality: Maslen nos Cape; **Record Level:** bibliographicCitation: Guéorguiev (1992: 65)
- **Type status:** Other material. **Occurrence:** recordedBy: V. Lazarov; individualCount: 1; **Location:** countryCode: BG; locality: Primorsko; **Event:** eventDate: VI.1957; **Record Level:** institutionCode: NMNHS

#### Pogonus (Pogonus) littoralis

(Duftschmid, 1812)

##### Materials

- **Type status:** Other material. **Location:** countryCode: BG; locality: Primorsko; **Record Level:** bibliographicCitation: Hieke & Wrase (1988: 67)

#### Pogonus (Pogonus) riparius

Dejean, 1828

##### Materials

- **Type status:** Other material. **Occurrence:** recordedBy: V. Lazarov; individualCount: 2; **Location:** countryCode: BG; locality: Primorsko; **Event:** eventDate: VI.1957; **Record Level:** institutionCode: NMNHS
- **Type status:** Other material. **Occurrence:** recordedBy: B. Guéorguiev; individualCount: 4; **Location:** countryCode: BG; locality: Veleka River near Mladezko Village; **Event:** eventDate: 25/05/1995; **Record Level:** institutionCode: NMNHS

#### Pogonus (Pogonus) transfuga

Chaudoir, 1871

##### Materials

- **Type status:** Other material. **Location:** countryCode: BG; locality: "Topolite" Camping site; **Record Level:** bibliographicCitation: Hieke & Wrase (1988: 68, as Pogonus persicus)

#### Perileptus (Perileptus) areolatus areolatus

(Creutzer, 1799)

##### Materials

- **Type status:** Other material. **Occurrence:** recordedBy: R. Bekchiev; individualCount: 1; **Location:** countryCode: BG; locality: Sinemorets Vill., PA"Silistar", Estuary of Silistar River; verbatimElevation: 12; verbatimCoordinates: N42°01'26.8", E28°00'32.9"; geodeticDatum: WGS84; **Event:** eventDate: 09/05/2009
- **Type status:** Other material. **Occurrence:** recordedBy: R. Kostova; individualCount: 1; **Location:** countryCode: BG; locality: Sinemorets Vill., PA"Silistar", Estuary of Silistar River; verbatimElevation: 12; verbatimCoordinates: N42°01'26.8", E28°00'32.9"; geodeticDatum: WGS84; **Event:** eventDate: 27/05/2009
- **Type status:** Other material. **Occurrence:** recordedBy: R. Bekchiev; individualCount: 2; **Location:** countryCode: BG; locality: Fazanovo Vill., "Popovi skali" Place; verbatimElevation: 26; verbatimCoordinates: N42°09'45.9", E27°44'15.6"; geodeticDatum: WGS84; **Event:** eventDate: 01/07/2009
- **Type status:** Other material. **Occurrence:** recordedBy: R. Bekchiev; individualCount: 6; **Location:** countryCode: BG; locality: Varvara Vill. surroundings; verbatimElevation: 46; verbatimCoordinates: N42°06'49.1", E27°53'18.9"; geodeticDatum: WGS84; **Event:** eventDate: 02/07/2009
- **Type status:** Other material. **Occurrence:** recordedBy: R. Kostova; individualCount: 1; **Location:** countryCode: TR; locality: Yalikŏy, river estuary; verbatimElevation: 9; verbatimCoordinates: N41°29'27.7", E28°16'40.0"; geodeticDatum: WGS84; **Event:** eventDate: 23/05/2011
- **Type status:** Other material. **Occurrence:** recordedBy: R. Bekchiev; individualCount: 2; **Location:** countryCode: TR; locality: Kizilagač, along river; verbatimElevation: 115; verbatimCoordinates: N41°41'01.9", E27°52'51.4"; geodeticDatum: WGS84; **Event:** eventDate: 24/05/2011
- **Type status:** Other material. **Occurrence:** recordedBy: R. Kostova; individualCount: 1; **Location:** countryCode: BG; locality: Sinemorets Vill., PA"Silistar", Estuary of Silistar River; verbatimElevation: 12; verbatimCoordinates: N42°01'26.8", E28°00'32.9"; geodeticDatum: WGS84; **Event:** eventDate: 27/05/2011
- **Type status:** Other material. **Location:** countryCode: BG; locality: Yasna Polyana Vill.; **Record Level:** bibliographicCitation: Hieke & Wrase (1988: 34)

#### Duvalius (Biharotrechus) bekchievi

Guéorguiev, 2011

##### Materials

- **Type status:** Other material. **Location:** countryCode: BG; locality: Bliznak Vill., PA"Bataka"; verbatimElevation: 324; verbatimCoordinates: N42°11'37.3", E27°19'35.8"; **Event:** habitat: oak forest; **Record Level:** bibliographicCitation: Gueorguiev & Kostova (2011: 306)
- **Type status:** Other material. **Location:** countryCode: BG; locality: Kosti Vill., Maharata Cave; verbatimElevation: 160; **Record Level:** bibliographicCitation: Gueorguiev & Kostova (2011: 306)
- **Type status:** Other material. **Location:** countryCode: BG; locality: Golyama Vapa Cave near "Petrova niva" Place; verbatimElevation: 255; verbatimCoordinates: N42°04'33.6", E27°30'45.0"; **Record Level:** bibliographicCitation: Gueorguiev & Kostova (2011: 306)

#### Trechus (Trechus) asiaticus

Jeannel, 1927

##### Materials

- **Type status:** Other material. **Location:** countryCode: BG; locality: Maslen nos Cape (= Zejtinburun); **Record Level:** bibliographicCitation: Mařan (1933: 90, as T. subnotatus asiaticus)
- **Type status:** Other material. **Location:** countryCode: BG; locality: Strandzha; **Record Level:** bibliographicCitation: Pawlowski (1973: 232)

#### Trechus (Trechus) crucifer

Poichard de la Brûlerie, 1876

##### Materials

- **Type status:** Other material. **Occurrence:** recordedBy: R. Bekchiev; individualCount: 1; **Location:** countryCode: BG; locality: Varvara Vill. surroundings; verbatimElevation: 46; verbatimCoordinates: N42°06'49.1", E27°53'18.9"; geodeticDatum: WGS84; **Event:** eventDate: 16/04/2009
- **Type status:** Other material. **Occurrence:** recordedBy: R. Bekchiev; individualCount: 1; **Location:** countryCode: BG; locality: Brodilovo Vill., under Papiya peak; verbatimElevation: 336; verbatimCoordinates: N42°06'23.7", E27°50'36.1"; geodeticDatum: WGS84; **Event:** eventDate: 16/04/2009; habitat: oak forest
- **Type status:** Other material. **Occurrence:** recordedBy: R. Bekchiev; individualCount: 1; **Location:** countryCode: BG; locality: Bulgari Vill., PA "Marina reka"; verbatimElevation: 172; verbatimCoordinates: N42°06'41.7", E27°45'53.0"; geodeticDatum: WGS84; **Event:** eventDate: 16/04/2009
- **Type status:** Other material. **Occurrence:** recordedBy: R. Bekchiev; individualCount: 2; **Location:** countryCode: BG; locality: Malko Tarnovo, "Propada" Place; verbatimElevation: 401; verbatimCoordinates: N41°58'49.6", E27°29'25.7"; geodeticDatum: WGS84; **Event:** eventDate: 17/04/2009; habitat: oriental beech forest
- **Type status:** Other material. **Occurrence:** recordedBy: R. Kostova; individualCount: 1; **Location:** countryCode: BG; locality: Brashlyan Vill. Surroundings; verbatimElevation: 341; verbatimCoordinates: N42°02'41.0", E27°25'25.5"; geodeticDatum: WGS84; **Event:** eventDate: 17/04/2009
- **Type status:** Other material. **Occurrence:** recordedBy: R. Kostova; individualCount: 1; **Location:** countryCode: BG; locality: Rezovo Vill. surroundings; verbatimElevation: 5; verbatimCoordinates: N41°58'54.4", E28°01'29.6"; geodeticDatum: WGS84; **Event:** eventDate: 09/05/2009; habitat: meadows
- **Type status:** Other material. **Occurrence:** recordedBy: R. Bekchiev; individualCount: 2; **Location:** countryCode: BG; locality: Malko Tarnovo, "Indipasqua" Place; verbatimElevation: 211; verbatimCoordinates: N42°00'16.9", E27°39'09.2"; geodeticDatum: WGS84; **Event:** eventDate: 25/05/2009
- **Type status:** Other material. **Occurrence:** recordedBy: R. Bekchiev; individualCount: 1; **Location:** countryCode: BG; locality: Brodilovo Vill., under Papiya peak; verbatimElevation: 336; verbatimCoordinates: N42°06'23.7", E27°50'36.1"; geodeticDatum: WGS84; **Event:** eventDate: 03/07/2009; habitat: oak forest
- **Type status:** Other material. **Occurrence:** recordedBy: R. Bekchiev; individualCount: 1; **Location:** countryCode: BG; locality: Fazanovo Vill., "Popovi skali" Place; verbatimElevation: 26; verbatimCoordinates: N42°09'45.9", E27°44'15.6"; geodeticDatum: WGS84; **Event:** eventDate: 01/07/2009
- **Type status:** Other material. **Occurrence:** recordedBy: R. Bekchiev; individualCount: 1; **Location:** countryCode: BG; locality: 5 km of Bliznak Village; verbatimElevation: 303; verbatimCoordinates: N42°10'19.8", E27°18'35.5"; geodeticDatum: WGS84; **Event:** eventDate: 08/06/2009
- **Type status:** Other material. **Occurrence:** recordedBy: R. Bekchiev; individualCount: 1; **Location:** countryCode: BG; locality: Brodilovo Vill.; verbatimElevation: 13; verbatimCoordinates: N42°05'18.0", E27°51'06.2"; geodeticDatum: WGS84; **Event:** eventDate: 04/07/2009
- **Type status:** Other material. **Occurrence:** recordedBy: R. Bekchiev; individualCount: 3; **Location:** countryCode: BG; locality: Bulgari Vill., PA "Marina reka"; verbatimElevation: 183; verbatimCoordinates: N42°06'41.7", E27°45'53.0"; geodeticDatum: WGS84; **Event:** eventDate: 11/06/2009
- **Type status:** Other material. **Occurrence:** recordedBy: R. Kostova; individualCount: 1; **Location:** countryCode: TR; locality: Demirköy, Dökümhanesi; verbatimElevation: 179; verbatimCoordinates: N41°49'09.6", E27°48'43.2"; geodeticDatum: WGS84; **Event:** eventDate: 03/10/2009
- **Type status:** Other material. **Occurrence:** recordedBy: R. Kostova; individualCount: 2; **Location:** countryCode: TR; locality: Demirköy surroundings; verbatimElevation: 618; verbatimCoordinates: N41°47'57.7", E27°44'02.4"; geodeticDatum: WGS84; **Event:** eventDate: 06/09/2009
- **Type status:** Other material. **Occurrence:** recordedBy: R. Bekchiev; individualCount: 2; **Location:** countryCode: BG; locality: Slivarovo Vill., "Shafariitsa" Place; verbatimElevation: 224; verbatimCoordinates: N41°57'37.8", E27°39'34.8"; geodeticDatum: WGS84; **Event:** eventDate: 25/09/2009
- **Type status:** Other material. **Occurrence:** recordedBy: R. Kostova; individualCount: 1; **Location:** countryCode: BG; locality: Bulgari Vill., PA "Marina reka"; verbatimElevation: 172; verbatimCoordinates: N42°06'41.7", E27°45'53.0"; geodeticDatum: WGS84; **Event:** eventDate: 28/05/2010
- **Type status:** Other material. **Occurrence:** recordedBy: R. Kostova; individualCount: 1; **Location:** countryCode: BG; locality: Kondolovo Vill., "Byalata prast" Place; verbatimElevation: 271; verbatimCoordinates: N42°05'47.7", E27°39'55.0"; geodeticDatum: WGS84; **Event:** eventDate: 15.04-08.05.2009; habitat: oak-beech mixed forest
- **Type status:** Other material. **Location:** countryCode: BG; locality: Maslen nos Cape (= Zejtinburun); **Record Level:** bibliographicCitation: Pawlowski (1973: 238)
- **Type status:** Other material. **Location:** countryCode: BG; locality: Primorsko, near Dyavolska reka River; **Record Level:** bibliographicCitation: Pawlowski (1973: 238)
- **Type status:** Other material. **Location:** countryCode: BG; locality: Sinemorets Vill.; **Record Level:** bibliographicCitation: Pawlowski (1973: 238)
- **Type status:** Other material. **Location:** countryCode: BG; locality: Bosna Ridge; **Record Level:** bibliographicCitation: Pawlowski (1973: 238)
- **Type status:** Other material. **Occurrence:** recordedBy: S. Andreev; individualCount: 1; **Location:** countryCode: BG; locality: Aydere River; **Event:** eventDate: 30/05/1982; habitat: moist leaf litter near brook; **Record Level:** institutionCode: NMNHS

#### Trechus (Trechus) nigrinus

Putzeys, 1847

##### Materials

- **Type status:** Other material. **Location:** countryCode: BG; locality: Bosna Ridge; **Record Level:** bibliographicCitation: Pawlowski (1973: 250, as T. tristis)
- **Type status:** Other material. **Location:** countryCode: BG; locality: Kalovo Vill.; **Record Level:** bibliographicCitation: Pawlowski (1973: 250, as T. tristis)
- **Type status:** Other material. **Location:** countryCode: BG; locality: Malko Tarnovo; **Record Level:** bibliographicCitation: Wrase (1991: 6, as T. tristis)

#### Trechus (Trechus) obtusus thracicus

Pawlowski, 1973

##### Materials

- **Type status:** Other material. **Occurrence:** recordedBy: R. Bekchiev; individualCount: 1; **Location:** countryCode: BG; locality: Mladezhko Vill., The springs of Mladezhka River; verbatimElevation: 231; verbatimCoordinates: N42°09'04.5", E27°21'26.1"; geodeticDatum: WGS84; **Event:** eventDate: 21/08/2009; habitat: black alder and hornbeam forest
- **Type status:** Other material. **Occurrence:** recordedBy: R. Kostova; individualCount: 1; **Location:** countryCode: BG; locality: Kirovo Vill., along Selska River; verbatimElevation: 138; verbatimCoordinates: N42°10'43.0", E27°10'58.2"; geodeticDatum: WGS84; **Event:** eventDate: 05/05/2009
- **Type status:** Other material. **Occurrence:** recordedBy: R. Kostova; individualCount: 1; **Location:** countryCode: BG; locality: Malko Tarnovo, "Propada" Place; verbatimElevation: 401; verbatimCoordinates: N41°58'49.6", E27°29'25.7"; geodeticDatum: WGS84; **Event:** eventDate: 26/07/2009; habitat: oriental beech forest
- **Type status:** Other material. **Occurrence:** recordedBy: R. Bekchiev; individualCount: 2; **Location:** countryCode: TR; locality: Demirköy, along Degirmen River; verbatimElevation: 289; verbatimCoordinates: N41°49'17.2", E27°45'07.7"; geodeticDatum: WGS84; **Event:** eventDate: 06/07/2009
- **Type status:** Other material. **Occurrence:** recordedBy: R. Kostova; individualCount: 1; **Location:** countryCode: TR; locality: Sarpdere Vill., Dupnisa Cave surroundings; verbatimElevation: 356; verbatimCoordinates: N41°50'26.0", E27°33'22.4"; geodeticDatum: WGS84; **Event:** eventDate: 26/05/2011
- **Type status:** Other material. **Occurrence:** recordedBy: R. Kostova; individualCount: 1; **Location:** countryCode: BG; locality: Kosti Vill., "St. Ilia" Place; verbatimElevation: 35; verbatimCoordinates: N42°03'23.2", E27°45'51.6"; geodeticDatum: WGS84; **Event:** eventDate: 09.05-08.06.2009; habitat: meadow with single trees
- **Type status:** Other material. **Occurrence:** recordedBy: R. Kostova; individualCount: 2; **Location:** countryCode: BG; locality: Kosti Vill., "St. Ilia" Place; verbatimElevation: 35; verbatimCoordinates: N42°03'23.2", E27°45'51.6"; geodeticDatum: WGS84; **Event:** eventDate: 03.08-07.09.2009; habitat: meadow with single trees
- **Type status:** Other material. **Occurrence:** recordedBy: R. Kostova; individualCount: 1; **Location:** countryCode: BG; locality: Sinemorets Vill., PA"Silistar"; verbatimElevation: 23; verbatimCoordinates: N42°01'30.7", E28°00'25.4"; geodeticDatum: WGS84; **Event:** eventDate: 09.05-08.06.2009; habitat: oak forest
- **Type status:** Other material. **Location:** countryCode: BG; locality: Vizitsa Vill.; **Record Level:** bibliographicCitation: Pawlowski (1973: 227, loc. typ.)
- **Type status:** Other material. **Location:** countryCode: BG; locality: Krushevets Vill.; **Record Level:** bibliographicCitation: Pawlowski (1973: 227)
- **Type status:** Other material. **Location:** countryCode: BG; locality: Vizitsa Vill.; **Record Level:** bibliographicCitation: Guéorguiev & Guéorguiev (1995: 74)
- **Type status:** Other material. **Location:** countryCode: BG; locality: Krushevets Vill.; **Record Level:** bibliographicCitation: Guéorguiev & Guéorguiev (1995: 74)
- **Type status:** Other material. **Occurrence:** recordedBy: P. Beron; individualCount: 1; **Location:** countryCode: BG; locality: Kiten; **Event:** eventDate: 16-22.12.1984; **Record Level:** institutionCode: NMNHS

#### Trechus (Trechus) quadristriatus

(Schrank, 1781)

##### Materials

- **Type status:** Other material. **Occurrence:** recordedBy: R. Bekchiev; individualCount: 1; **Location:** countryCode: BG; locality: Mladezhko Vill., The springs of Mladezhka River; verbatimElevation: 231; verbatimCoordinates: N42°09'04.5", E27°21'26.1"; geodeticDatum: WGS84; **Event:** eventDate: 15/04/2009; habitat: black alder and hornbeam forest
- **Type status:** Other material. **Occurrence:** recordedBy: R. Bekchiev; individualCount: 1; **Location:** countryCode: BG; locality: Stoilovo Vill., "Sredoka" reserve, along Mechi dol river; verbatimElevation: 206; verbatimCoordinates: N42°01'51.1", E27°30'50.1"; geodeticDatum: WGS84; **Event:** eventDate: 19/04/2009
- **Type status:** Other material. **Occurrence:** recordedBy: R. Kostova; individualCount: 2; **Location:** countryCode: BG; locality: Stoilovo Vill. surroundings; verbatimElevation: 290; verbatimCoordinates: N42°00'24.0", E27°30'14.1"; geodeticDatum: WGS84; **Event:** eventDate: 07/05/2009
- **Type status:** Other material. **Occurrence:** recordedBy: R. Kostova; individualCount: 1; **Location:** countryCode: BG; locality: Brodilovo Vill., under Papiya peak; verbatimElevation: 336; verbatimCoordinates: N42°06'23.7", E27°50'36.1"; geodeticDatum: WGS84; **Event:** eventDate: 08/05/2009; habitat: oak forest
- **Type status:** Other material. **Occurrence:** recordedBy: R. Bekchiev; individualCount: 1; **Location:** countryCode: BG; locality: Malko Tarnovo, "Propada" Place; verbatimElevation: 401; verbatimCoordinates: N41°58'49.6", E27°29'25.7"; geodeticDatum: WGS84; **Event:** eventDate: 08/06/2009; habitat: oriental beech forest
- **Type status:** Other material. **Occurrence:** recordedBy: R. Kostova & R. Bekchiev; individualCount: 31; **Location:** countryCode: BG; locality: Varovnik Vill., near the bridge at Fakiiska River; verbatimElevation: 125; verbatimCoordinates: N42°13'33.7", E27°11'33.7"; geodeticDatum: WGS84; **Event:** eventDate: 09/06/2009
- **Type status:** Other material. **Occurrence:** recordedBy: R. Bekchiev; individualCount: 1; **Location:** countryCode: BG; locality: Stoilovo vill., "Sredoka" reserve, along Mechi dol river; verbatimElevation: 206; verbatimCoordinates: N42°01'51.1", E27°30'50.1"; geodeticDatum: WGS84; **Event:** eventDate: 10/06/2009
- **Type status:** Other material. **Occurrence:** recordedBy: R. Bekchiev; individualCount: 1; **Location:** countryCode: BG; locality: Brodilovo Vill., under Papiya peak; verbatimElevation: 336; verbatimCoordinates: N42°06'23.7", E27°50'36.1"; geodeticDatum: WGS84; **Event:** eventDate: 03/07/2009; habitat: oak forest
- **Type status:** Other material. **Occurrence:** recordedBy: A. Gijonova; individualCount: 3; **Location:** countryCode: TR; locality: Along Pinarhisar - Demirköy road; verbatimElevation: 618; verbatimCoordinates: N41°47'57.7", E27°44'02.4"; geodeticDatum: WGS84; **Event:** eventDate: 06/07/2009
- **Type status:** Other material. **Occurrence:** recordedBy: R. Kostova; individualCount: 2; **Location:** countryCode: TR; locality: Sarpdere Vill., Dupnisa Cave surroundings; verbatimElevation: 440; verbatimCoordinates: N41°49'56.3", E27°33'24.0"; geodeticDatum: WGS84; **Event:** eventDate: 08/07/2009
- **Type status:** Other material. **Occurrence:** recordedBy: R. Kostova; individualCount: 20; **Location:** countryCode: TR; locality: Sarpdere Vill., entrance of Dupnisa Cave; verbatimElevation: 359; verbatimCoordinates: N41°50'25.7", E27°33'21.9"; geodeticDatum: WGS84; **Event:** eventDate: 08/07/2009
- **Type status:** Other material. **Occurrence:** recordedBy: R. Bekchiev; individualCount: 1; **Location:** countryCode: BG; locality: Mladezhko Vill., The springs of Mladezhka River; verbatimElevation: 231; verbatimCoordinates: N42°09'04.5", E27°21'26.1"; geodeticDatum: WGS84; **Event:** eventDate: 21/08/2009; habitat: black alder and hornbeam forest
- **Type status:** Other material. **Occurrence:** recordedBy: R. Bekchiev; individualCount: 15; **Location:** countryCode: BG; locality: Malko Tarnovo, "Propada" Place; verbatimElevation: 401; verbatimCoordinates: N41°58'49.6", E27°29'25.7"; geodeticDatum: WGS84; **Event:** eventDate: 09/06/2009; habitat: oriental beech forest
- **Type status:** Other material. **Occurrence:** recordedBy: R. Bekchiev; individualCount: 3; **Location:** countryCode: BG; locality: Fazanovo Vill., "Popovi skali" Place; verbatimElevation: 26; verbatimCoordinates: N42°09'45.9", E27°44'15.6"; geodeticDatum: WGS84; **Event:** eventDate: 01/07/2009
- **Type status:** Other material. **Occurrence:** recordedBy: R. Bekchiev; individualCount: 2; **Location:** countryCode: BG; locality: 5 km of Bliznak Village; verbatimElevation: 303; verbatimCoordinates: N42°10'19.8", E27°18'35.5"; geodeticDatum: WGS84; **Event:** eventDate: 08/06/2009
- **Type status:** Other material. **Occurrence:** recordedBy: R. Bekchiev; individualCount: 2; **Location:** countryCode: BG; locality: Stoilovo vill., "Sredoka" reserve, along Mechi dol river; verbatimElevation: 206; verbatimCoordinates: N42°01'51.1", E27°30'50.1"; geodeticDatum: WGS84; **Event:** eventDate: 23/08/2009
- **Type status:** Other material. **Occurrence:** recordedBy: R. Bekchiev; individualCount: 1; **Location:** countryCode: BG; locality: Bulgari Vill., PA "Marina reka"; verbatimElevation: 183; verbatimCoordinates: N42°06'41.7", E27°45'53.0"; geodeticDatum: WGS84; **Event:** eventDate: 03/07/2009
- **Type status:** Other material. **Occurrence:** recordedBy: R. Bekchiev; individualCount: 4; **Location:** countryCode: TR; locality: Vize surroundings; verbatimElevation: 366; verbatimCoordinates: N41°35'06.1", E27°47'41.5"; geodeticDatum: WGS84; **Event:** eventDate: 25/05/2011
- **Type status:** Other material. **Occurrence:** recordedBy: R. Bekchiev; individualCount: 4; **Location:** countryCode: TR; locality: Sislioba Vill. surroundings; verbatimElevation: 49; verbatimCoordinates: N41°57'44.2", E27°54'36.1"; geodeticDatum: WGS84; **Event:** eventDate: 08/07/2009
- **Type status:** Other material. **Occurrence:** recordedBy: R. Kostova; individualCount: 8; **Location:** countryCode: TR; locality: Sarpdere Vill., entrance of Dupnisa Cave; verbatimElevation: 436; verbatimCoordinates: N41°50'26.0", E27°33'22.4"; geodeticDatum: WGS84; **Event:** eventDate: 08/07/2009
- **Type status:** Other material. **Occurrence:** recordedBy: R. Bekchiev; individualCount: 11; **Location:** countryCode: TR; locality: Demirköy surroundings; verbatimElevation: 618; verbatimCoordinates: N41°47'57.7", E27°44'02.4"; geodeticDatum: WGS84; **Event:** eventDate: 06/09/2009
- **Type status:** Other material. **Occurrence:** recordedBy: R. Bekchiev; individualCount: 1; **Location:** countryCode: TR; locality: Igneada surroundings; verbatimElevation: 3; verbatimCoordinates: N41°51'27.3", E27°57'28.7"; geodeticDatum: WGS84; **Event:** eventDate: 29/09/2009; habitat: marsh
- **Type status:** Other material. **Occurrence:** recordedBy: R. Bekchiev; individualCount: 5; **Location:** countryCode: TR; locality: Sarpdere Vill., entrance of Dupnisa Cave; verbatimElevation: 436; verbatimCoordinates: N41°50'26.0", E27°33'22.4"; geodeticDatum: WGS84; **Event:** eventDate: 29/09/2009
- **Type status:** Other material. **Occurrence:** recordedBy: R. Bekchiev; individualCount: 1; **Location:** countryCode: TR; locality: Sarpdere Vill., Dupnisa Cave surroundings; verbatimElevation: 356; verbatimCoordinates: N41°50'26.0", E27°33'22.4"; geodeticDatum: WGS84; **Event:** eventDate: 01/10/2009
- **Type status:** Other material. **Occurrence:** recordedBy: R. Bekchiev; individualCount: 1; **Location:** countryCode: TR; locality: Along Demirköy - Kıyıköy road; verbatimElevation: 199; verbatimCoordinates: N41°39'43.2", E27°55'25.8"; geodeticDatum: WGS84; **Event:** eventDate: 24/05/2010
- **Type status:** Other material. **Occurrence:** recordedBy: R. Bekchiev; individualCount: 1; **Location:** countryCode: TR; locality: Igneada, Hamam Gölü; verbatimElevation: 11; verbatimCoordinates: N41°49'31.6", E27°57'33.8"; geodeticDatum: WGS84; **Event:** eventDate: 25/05/2010
- **Type status:** Other material. **Occurrence:** recordedBy: R. Kostova; individualCount: 1; **Location:** countryCode: BG; locality: Malko Tarnovo surroundings; verbatimElevation: 375; verbatimCoordinates: N41°59'01.7", E27°29'49.4"; geodeticDatum: WGS84; **Event:** eventDate: 27/05/2010
- **Type status:** Other material. **Occurrence:** recordedBy: I. Gjonov; individualCount: 1; **Location:** countryCode: BG; locality: Sinemorets Vill., PA"Silistar", "Lipite" Beach; verbatimElevation: 54; verbatimCoordinates: N42°02'40.2", E27°59'21.8"; geodeticDatum: WGS84; **Event:** eventDate: 29/05/2010
- **Type status:** Other material. **Occurrence:** recordedBy: R. Bekchiev; individualCount: 1; **Location:** countryCode: TR; locality: Kiyiköy surroundings; verbatimElevation: 38; verbatimCoordinates: N41°39'25.7", E28°05'11.2"; geodeticDatum: WGS84; **Event:** eventDate: 22/05/2011
- **Type status:** Other material. **Occurrence:** recordedBy: R. Bekchiev; individualCount: 3; **Location:** countryCode: TR; locality: Sergen Vill. surroundings; verbatimElevation: 818; verbatimCoordinates: N41°44'37.0", E27°42'24.8"; geodeticDatum: WGS84; **Event:** eventDate: 25/05/2011
- **Type status:** Other material. **Occurrence:** recordedBy: R. Kostova; individualCount: 5; **Location:** countryCode: BG; locality: Bliznak Vill., PA"Bataka"; verbatimElevation: 324; verbatimCoordinates: N42°11'37.3", E27°19'35.8"; geodeticDatum: WGS84; **Event:** eventDate: 15.04-02.07.2009; habitat: oak forest
- **Type status:** Other material. **Occurrence:** recordedBy: R. Kostova; individualCount: 9; **Location:** countryCode: BG; locality: Malko Tarnovo, "Propada" Place; verbatimElevation: 401; verbatimCoordinates: N41°58'49.6", E27°29'25.7"; geodeticDatum: WGS84; **Event:** eventDate: 09.05-02.07.2009; habitat: oriental beech forest
- **Type status:** Other material. **Occurrence:** recordedBy: R. Kostova; individualCount: 2; **Location:** countryCode: BG; locality: Slivarovo Vill., "Shafariitsa" Place; verbatimElevation: 224; verbatimCoordinates: N41°57'37.8", E27°39'34.8"; geodeticDatum: WGS84; **Event:** eventDate: 09.05-08.06.2009; habitat: black alder-oak forest
- **Type status:** Other material. **Occurrence:** recordedBy: R. Kostova; individualCount: 2; **Location:** countryCode: BG; locality: Kosti Vill., "St. Ilia" Place; verbatimElevation: 35; verbatimCoordinates: N42°03'23.2", E27°45'51.6"; geodeticDatum: WGS84; **Event:** eventDate: 09.05-08.06.2009; habitat: meadow with single trees
- **Type status:** Other material. **Occurrence:** recordedBy: R. Kostova; individualCount: 1; **Location:** countryCode: BG; locality: Sinemorets Vill., PA"Silistar", "Butamyata" Place; verbatimElevation: 17; verbatimCoordinates: N42°03'10.3", E27°59'13.4"; geodeticDatum: WGS84; **Event:** eventDate: 09.05-08.06.2009; habitat: oak forest
- **Type status:** Other material. **Location:** countryCode: BG; locality: Strandzha; **Record Level:** bibliographicCitation: Rambousek (1912: 76)
- **Type status:** Other material. **Location:** countryCode: BG; locality: Valey of Ropotamo River; **Record Level:** bibliographicCitation: Pawlowski (1973: 226)
- **Type status:** Other material. **Location:** countryCode: BG; locality: Primorsko; **Record Level:** bibliographicCitation: Pawlowski (1973: 226)
- **Type status:** Other material. **Location:** countryCode: BG; locality: Sinemorets Vill.; **Record Level:** bibliographicCitation: Pawlowski (1973: 226)
- **Type status:** Other material. **Location:** countryCode: BG; locality: Vizitsa Vill.; **Record Level:** bibliographicCitation: Pawlowski (1973: 226)
- **Type status:** Other material. **Location:** countryCode: BG; locality: Bosna Vill. - Krushevets Vill.; **Record Level:** bibliographicCitation: Pawlowski (1973: 226)
- **Type status:** Other material. **Location:** countryCode: BG; locality: Yasna polyana Vill., 5 km west; **Record Level:** bibliographicCitation: Hieke & Wrase (1988: 35)
- **Type status:** Other material. **Occurrence:** recordedBy: D. Ioakimov; individualCount: 1; **Location:** countryCode: BG; locality: Strandzha; verbatimElevation: 260; **Event:** eventDate: 09/06/1927; **Record Level:** institutionCode: NMNHS
- **Type status:** Other material. **Occurrence:** recordedBy: B. Guéorguiev; individualCount: 7; **Location:** countryCode: BG; locality: Sinemorets Village, south bay; **Event:** eventDate: 27/05/1995; **Record Level:** institutionCode: NMNHS
- **Type status:** Other material. **Occurrence:** recordedBy: B. Guéorguiev; individualCount: 1; **Location:** countryCode: BG; locality: Tisovitsa Reserve near Bulgari Vill.; **Event:** eventDate: 27/05/1995; **Record Level:** institutionCode: NMNHS
- **Type status:** Other material. **Occurrence:** recordedBy: B. Guéorguiev; individualCount: 1; **Location:** countryCode: BG; locality: Arapya, near Tsarevo; **Event:** eventDate: 28/05/1995; **Record Level:** institutionCode: NMNHS
- **Type status:** Other material. **Occurrence:** recordedBy: P. Stoev & S. Lazarov; individualCount: 1; **Location:** countryCode: TR; locality: Near Dupnitsa Cave, 4 km SW Sardere Vill.; verbatimElevation: 348; verbatimCoordinates: N41°50'26.8", E27°33'21.5"; **Event:** eventDate: 25/07/2006; habitat: Fagus & Quercus mixed forest, leaf litter; **Record Level:** institutionCode: NMNHS

#### Trechus (Trechus) sp. aff. irenis

Csiki, 1912

##### Materials

- **Type status:** Other material. **Occurrence:** recordedBy: R. Bekchiev; individualCount: 1; **Location:** countryCode: BG; locality: Bliznak Vill., PA"Bataka"; verbatimElevation: 324; verbatimCoordinates: N42°11'37.3", E27°19'35.8"; geodeticDatum: WGS84; **Event:** eventDate: 05/05/2009; habitat: oak forest
- **Type status:** Other material. **Occurrence:** recordedBy: R. Kostova & R. Bekchiev; individualCount: 5; **Location:** countryCode: BG; locality: Malko Tarnovo, "Indipasqua" Place; verbatimElevation: 211; verbatimCoordinates: N42°00'16.9", E27°39'09.2"; geodeticDatum: WGS84; **Event:** eventDate: 25/05/2009
- **Type status:** Other material. **Occurrence:** recordedBy: R. Bekchiev; individualCount: 4; **Location:** countryCode: BG; locality: Malko Tarnovo, "Propada" Place; verbatimElevation: 401; verbatimCoordinates: N41°58'49.6", E27°29'25.7"; geodeticDatum: WGS84; **Event:** eventDate: 09/06/2009; habitat: oriental beech forest
- **Type status:** Other material. **Occurrence:** recordedBy: R. Bekchiev; individualCount: 1; **Location:** countryCode: BG; locality: Bulgari Vill., PA "Marina reka"; verbatimElevation: 183; verbatimCoordinates: N42°06'41.7", E27°45'53.0"; geodeticDatum: WGS84; **Event:** eventDate: 11/06/2009
- **Type status:** Other material. **Occurrence:** recordedBy: R. Bekchiev; individualCount: 1; **Location:** countryCode: BG; locality: Bulgari Vill., PA "Marina reka"; verbatimElevation: 183; verbatimCoordinates: N42°06'41.7", E27°45'53.0"; geodeticDatum: WGS84; **Event:** eventDate: 03/07/2009
- **Type status:** Other material. **Occurrence:** recordedBy: R. Bekchiev; individualCount: 1; **Location:** countryCode: BG; locality: Malko Tarnovo, "Propada" Place; verbatimElevation: 401; verbatimCoordinates: N41°58'49.6", E27°29'25.7"; geodeticDatum: WGS84; **Event:** eventDate: 26/07/2009; habitat: oriental beech forest
- **Type status:** Other material. **Occurrence:** recordedBy: R. Bekchiev; individualCount: 2; **Location:** countryCode: BG; locality: Stoilovo vill., "Sredoka" Reserve, along Mechi dol River; verbatimElevation: 206; verbatimCoordinates: N42°01'51.1", E27°30'50.1"; geodeticDatum: WGS84; **Event:** eventDate: 23/08/2009
- **Type status:** Other material. **Occurrence:** recordedBy: R. Bekchiev; individualCount: 2; **Location:** countryCode: BG; locality: Brashlyan Vill.; verbatimElevation: 341; verbatimCoordinates: N42°02'41.0", E27°25'25.5"; geodeticDatum: WGS84; **Event:** eventDate: 22/08/2009
- **Type status:** Other material. **Occurrence:** recordedBy: R. Bekchiev; individualCount: 4; **Location:** countryCode: TR; locality: Demirköy, Dökümhanesi; verbatimElevation: 179; verbatimCoordinates: N41°49'09.6", E27°48'43.2"; geodeticDatum: WGS84; **Event:** eventDate: 30/05/2009
- **Type status:** Other material. **Occurrence:** recordedBy: R. Bekchiev; individualCount: 6; **Location:** countryCode: TR; locality: Demirköy, to Mahya Dağı Peak; verbatimElevation: 670; verbatimCoordinates: N41°47'44.6", E27°44'13.2"; geodeticDatum: WGS84; **Event:** eventDate: 29/09/2009
- **Type status:** Other material. **Occurrence:** recordedBy: R. Bekchiev; individualCount: 1; **Location:** countryCode: TR; locality: Sarpdere Vill., entrance of Dupnisa Cave; verbatimElevation: 436; verbatimCoordinates: N41°50'26.0", E27°33'22.4"; geodeticDatum: WGS84; **Event:** eventDate: 08/07/2009
- **Type status:** Other material. **Occurrence:** recordedBy: R. Kostova; individualID: R.Kostova; individualCount: 7; **Location:** countryCode: TR; locality: Demirköy surroundings; verbatimElevation: 618; verbatimCoordinates: N41°47'57.7", E27°44'02.4"; geodeticDatum: WGS84; **Event:** eventDate: 06/09/2009
- **Type status:** Other material. **Occurrence:** recordedBy: R. Kostova; individualCount: 1; **Location:** countryCode: BG; locality: Slivarovo Vill., "Shafariitsa" Place; verbatimElevation: 224; verbatimCoordinates: N41°57'37.8", E27°39'34.8"; geodeticDatum: WGS84; **Event:** eventDate: 25/09/2009
- **Type status:** Other material. **Occurrence:** recordedBy: R. Bekchiev; individualCount: 1; **Location:** countryCode: TR; locality: Sarpdere Vill., Dupnisa Cave surroundings; verbatimElevation: 356; verbatimCoordinates: N41°50'26.0", E27°33'22.4"; geodeticDatum: WGS84; **Event:** eventDate: 23/05/2010
- **Type status:** Other material. **Occurrence:** recordedBy: R. Bekchiev; individualCount: 1; **Location:** countryCode: TR; locality: Sarpdere Vill., Dupnisa Cave; verbatimElevation: 359; verbatimCoordinates: N41°50'25.7", E27°33'21.9"; geodeticDatum: WGS84; **Event:** eventDate: 29/09/2009
- **Type status:** Other material. **Occurrence:** recordedBy: R. Bekchiev; individualCount: 1; **Location:** countryCode: TR; locality: Sarpdere Vill., Dupnisa Cave surroundings; verbatimElevation: 356; verbatimCoordinates: N41°50'26.0", E27°33'22.4"; geodeticDatum: WGS84; **Event:** eventDate: 01/10/2009
- **Type status:** Other material. **Occurrence:** recordedBy: R. Bekchiev; individualCount: 1; **Location:** countryCode: TR; locality: Igneada, Hamam Gölü; verbatimElevation: 5; verbatimCoordinates: N41°49'43.2", E27°57'31.1"; geodeticDatum: WGS84; **Event:** eventDate: 02/10/2009
- **Type status:** Other material. **Occurrence:** recordedBy: R. Kostova; individualCount: 1; **Location:** countryCode: TR; locality: Sarpdere Vill., Dupnisa Cave surroundings; verbatimElevation: 356; verbatimCoordinates: N41°50'26.0", E27°33'22.4"; geodeticDatum: WGS84; **Event:** eventDate: 24/05/2011
- **Type status:** Other material. **Occurrence:** recordedBy: R. Kostova; individualCount: 9; **Location:** countryCode: BG; locality: Slivarovo Vill., "Shafariitsa" Place; verbatimElevation: 224; verbatimCoordinates: N41°57'37.8", E27°39'34.8"; geodeticDatum: WGS84; **Event:** eventDate: 15.04-02.07.2009; habitat: black alder-oak forest
- **Type status:** Other material. **Occurrence:** recordedBy: R. Kostova; individualCount: 1; **Location:** countryCode: BG; locality: Sinemorets Vill., PA "Estuary of Veleka river"; verbatimElevation: 6; verbatimCoordinates: N42°03'39.6", E27°57'55.2"; geodeticDatum: WGS84; **Event:** eventDate: 15.04-08.05.2009; habitat: swamp forest
- **Type status:** Other material. **Location:** countryCode: BG; locality: Tsarevo (= Micurin); **Record Level:** bibliographicCitation: Hieke & Wrase (1988: 36, as T. cardioderus balcanicus)
- **Type status:** Other material. **Occurrence:** recordedBy: S. Andreev; individualCount: 3; **Location:** countryCode: BG; locality: Aydere River; **Event:** eventDate: 30/05/1982; habitat: moist leaf litter near brook; **Record Level:** institutionCode: NMNHS

#### Chlaenius (Chlaeniellus) flavipes

Ménétriés, 1832

##### Materials

- **Type status:** Other material. **Location:** countryCode: BG; locality: Arcutino; **Record Level:** bibliographicCitation: Hieke & Wrase (1988: 149)

#### Chlaenius (Chlaeniellus) nigricornis

(Fabricius, 1787)

##### Materials

- **Type status:** Other material. **Occurrence:** recordedBy: R. Kostova; individualCount: 1; **Location:** countryCode: TR; locality: Kiyiköy surroundings; verbatimElevation: 62; verbatimCoordinates: N41°37'40.7", E28°04'05.0"; geodeticDatum: WGS84; **Event:** eventDate: 22/05/2011
- **Type status:** Other material. **Occurrence:** recordedBy: R. Kostova; individualCount: 1; **Location:** countryCode: TR; locality: Kiyiköy surroundings; verbatimElevation: 38; verbatimCoordinates: N41°39'25.7", E28°05'11.2"; geodeticDatum: WGS84; **Event:** eventDate: 22/05/2011
- **Type status:** Other material. **Occurrence:** recordedBy: R. Kostova & R. Bekchiev; individualCount: 15; **Location:** countryCode: BG; locality: Sinemorets Vill., PA "Estuary of Veleka river"; verbatimElevation: 6; verbatimCoordinates: N42°03'39.6", E27°57'55.2"; geodeticDatum: WGS84; **Event:** eventDate: 09.05-02.07.2009; habitat: swamp forest

#### Chlaenius (Chlaeniellus) nitidulus

(Schrank, 1781)

##### Materials

- **Type status:** Other material. **Occurrence:** recordedBy: R. Bekchiev; individualCount: 1; **Location:** countryCode: TR; locality: Kiyiköy surroundings; verbatimElevation: 32; verbatimCoordinates: N41°38'12.8", E28°04'08.7"; geodeticDatum: WGS84; **Event:** eventDate: 22/05/2011

#### Chlaenius (Chlaeniellus) tristis tristis

(Schaller, 1783)

##### Materials

- **Type status:** Other material. **Occurrence:** recordedBy: R. Kostova; **Location:** countryCode: TR; locality: Igneada surroundings; verbatimElevation: 3; verbatimCoordinates: N41°51'27.3", E27°57'28.7"; geodeticDatum: WGS84; **Event:** eventDate: 06/07/2009; habitat: marsh
- **Type status:** Other material. **Occurrence:** recordedBy: R. Radev; **Location:** countryCode: BG; locality: Sinemorets Vill.; **Event:** eventDate: 7.VIII.1989; **Record Level:** collectionID: R. Radev coll.

#### Chlaenius (Chlaeniellus) vestitus

(Paykull, 1790)

##### Materials

- **Type status:** Other material. **Occurrence:** recordedBy: R. Bekchiev; individualCount: 1; **Location:** countryCode: BG; locality: Stoilovo Vill., "Petrova niva" Place; verbatimElevation: 132; verbatimCoordinates: N42°03'41.9", E27°31'58.9"; geodeticDatum: WGS84; **Event:** eventDate: 06/05/2009; habitat: meadow, single shrubs
- **Type status:** Other material. **Occurrence:** recordedBy: R. Bekchiev; individualCount: 1; **Location:** countryCode: BG; locality: Fazanovo Vill., "Popovi skali" Place; verbatimElevation: 26; verbatimCoordinates: N42°09'45.9", E27°44'15.6"; geodeticDatum: WGS84; **Event:** eventDate: 01/07/2009
- **Type status:** Other material. **Occurrence:** recordedBy: R. Kostova; individualCount: 2; **Location:** countryCode: TR; locality: Igneada surroundings; verbatimElevation: 3; verbatimCoordinates: N41°51'27.3", E27°57'28.7"; geodeticDatum: WGS84; **Event:** eventDate: 06/07/2009; habitat: marsh
- **Type status:** Other material. **Occurrence:** recordedBy: I. Gijonov; individualCount: 2; **Location:** countryCode: BG; locality: Sinemorets Vill., PA"Silistar", "Lipite" Beach; verbatimElevation: 54; verbatimCoordinates: N42°02'40.2", E27°59'21.8"; geodeticDatum: WGS84; **Event:** eventDate: 29/05/2010
- **Type status:** Other material. **Occurrence:** recordedBy: R. Kostova; individualCount: 2; **Location:** countryCode: BG; locality: Sinemorets Vill., PA"Silistar", Estuary of Silistar River; verbatimElevation: 12; verbatimCoordinates: N42°01'26.8", E28°00'32.9"; geodeticDatum: WGS84; **Event:** eventDate: 27/05/2011
- **Type status:** Other material. **Occurrence:** recordedBy: J. Mařan & K. Taborsky; individualCount: 8; **Location:** countryCode: BG; locality: Maslen nos Cape; **Event:** eventDate: VI.1933; **Record Level:** institutionCode: NMP
- **Type status:** Other material. **Location:** countryCode: BG; locality: Rezovo Vill.; **Record Level:** bibliographicCitation: Guéorguiev & Guéorguiev (1995: 215)
- **Type status:** Other material. **Location:** countryCode: BG; locality: Ahtopol; **Record Level:** bibliographicCitation: Hieke & Wrase (1988: 148)
- **Type status:** Other material. **Location:** countryCode: BG; locality: Tsarevo (= Micurin); **Record Level:** bibliographicCitation: Hieke & Wrase (1988: 148)
- **Type status:** Other material. **Location:** countryCode: BG; locality: Mouth of Ropotamo River; **Record Level:** bibliographicCitation: Hieke & Wrase (1988: 148)
- **Type status:** Other material. **Occurrence:** recordedBy: B. Guéorguiev; individualCount: 1; **Location:** countryCode: BG; locality: Nature Park Ropotamo; **Event:** eventDate: 29/05/1995; **Record Level:** institutionCode: NMNHS

#### Chlaenius (Chlaenites) spoliatus spoliatus

(P. Rossi, 1790)

##### Materials

- **Type status:** Other material. **Occurrence:** recordedBy: R. Bekchiev; individualCount: 1; **Location:** countryCode: TR; locality: Kiyiköy surroundings; verbatimElevation: 32; verbatimCoordinates: N41°38'12.8", E28°04'08.7"; geodeticDatum: WGS84; **Event:** eventDate: 22/05/2011
- **Type status:** Other material. **Occurrence:** recordedBy: J. Mařan & K. Taborsky; individualCount: 3; **Location:** countryCode: BG; locality: Maslen nos Cape; **Event:** eventDate: VI.1933; **Record Level:** institutionCode: NMP
- **Type status:** Other material. **Location:** countryCode: BG; locality: Ahtopol; **Record Level:** bibliographicCitation: Hieke & Wrase (1988: 147)

#### Chlaenius (Chlaenius) festivus festivus

(Panzer, 1796)

##### Materials

- **Type status:** Other material. **Occurrence:** recordedBy: R. Kostova; individualCount: 1; **Location:** countryCode: BG; locality: Brodilovo Vill, along Eleshnitsa River; verbatimElevation: 17; verbatimCoordinates: N42°05'35.4", E27°49'53.0"; geodeticDatum: WGS84; **Event:** eventDate: 05/07/2009
- **Type status:** Other material. **Occurrence:** recordedBy: W. Beier; individualCount: 1; **Location:** countryCode: BG; locality: Mouth of Veleka River near Sinemorets Vill.; verbatimElevation: 3; verbatimCoordinates: N42°03'51.0", E27°58'14.0"; geodeticDatum: WGS84; **Event:** eventDate: 27/04/2001; **Record Level:** collectionCode: cWB
- **Type status:** Other material. **Occurrence:** recordedBy: J. Mařan & K. Taborsky; individualCount: 7; **Location:** countryCode: BG; locality: Maslen nos Cape; **Event:** eventDate: VI.1933; **Record Level:** institutionCode: NMP
- **Type status:** Other material. **Location:** countryCode: BG; locality: Ropotamo; **Record Level:** bibliographicCitation: Hieke & Wrase (1988: 148)

#### Chlaenius (Dinodes) decipiens

(L. Dufour, 1820)

##### Materials

- **Type status:** Other material. **Location:** countryCode: BG; locality: Kosti Vill.; **Record Level:** bibliographicCitation: Guéorguiev & Guéorguiev (1995: 212)
- **Type status:** Other material. **Location:** countryCode: BG; locality: Rezovo Vill.; **Record Level:** bibliographicCitation: Guéorguiev & Guéorguiev (1995: 212)
- **Type status:** Other material. **Location:** countryCode: BG; locality: Vizitsa Vill.; **Record Level:** bibliographicCitation: Guéorguiev & Guéorguiev (1995: 212)
- **Type status:** Other material. **Occurrence:** recordedBy: J. Mařan & K. Taborsky; individualCount: 2; **Location:** countryCode: BG; locality: Maslen nos Cape; **Event:** eventDate: VI.1933; **Record Level:** institutionCode: NMP (det. as Chlaenius decipiens ambiguus)
- **Type status:** Other material. **Occurrence:** recordedBy: K. Tuleshkov; individualCount: 1; **Location:** countryCode: BG; locality: Ahtopol; **Event:** eventDate: 15/05/1930; **Record Level:** institutionCode: NMNHS
- **Type status:** Other material. **Occurrence:** recordedBy: P. Drenski; individualCount: 1; **Location:** countryCode: BG; locality: Tsarevo (= Micurin); **Event:** eventDate: 07/06/1954; **Record Level:** institutionCode: NMNHS

#### Chlaenius (Trichochlaenius) aeneocephalus aeneocephalus

Dejean, 1826

##### Materials

- **Type status:** Other material. **Occurrence:** recordedBy: R. Kostova; individualCount: 1; **Location:** countryCode: BG; locality: Stoilovo Vill. surroundings; verbatimElevation: 290; verbatimCoordinates: N42°00'24.0", E27°30'14.1"; geodeticDatum: WGS84; **Event:** eventDate: 07/05/2009
- **Type status:** Other material. **Occurrence:** recordedBy: R. Kostova; individualCount: 1; **Location:** countryCode: BG; locality: Sinemorets Vill., PA"Silistar", "Butamyata" Place; verbatimElevation: 17; verbatimCoordinates: N42°03'10.3", E27°59'13.4"; geodeticDatum: WGS84; **Event:** eventDate: 09.06-02.07.2009; habitat: oak forest
- **Type status:** Other material. **Occurrence:** recordedBy: J. Mařan & K. Taborsky; individualCount: 3; **Location:** countryCode: BG; locality: Maslen nos Cape; **Event:** eventDate: VI.1933; **Record Level:** institutionCode: NMP (det. as Ch. gracilis Dej.)
- **Type status:** Other material. **Location:** countryCode: BG; locality: Strandzha; **Record Level:** bibliographicCitation: Guéorguiev & Guéorguiev (1995: 212)
- **Type status:** Other material. **Location:** countryCode: BG; locality: Kiten; **Record Level:** bibliographicCitation: Hieke & Wrase (1988: 148)
- **Type status:** Other material. **Occurrence:** recordedBy: V. Lazarov; individualCount: 2; **Location:** countryCode: BG; locality: Primorsko; **Event:** eventDate: VI.1957; **Record Level:** institutionCode: NMNHS
- **Type status:** Other material. **Occurrence:** recordedBy: K. Sevar; individualCount: 1; **Location:** countryCode: BG; locality: Tsarevo (= Micurin); **Event:** eventDate: 14/06/1973; **Record Level:** institutionCode: NMNHS
- **Type status:** Other material. **Occurrence:** recordedBy: B. Guéorguiev; individualCount: 1; **Location:** countryCode: BG; locality: Primorsko Town env.; **Event:** eventDate: 20/08/2004; habitat: oak forest; fieldNumber: un; **Record Level:** institutionCode: NMNHS

#### Drypta (Drypta) dentata

(P. Rossi, 1790)

##### Materials

- **Type status:** Other material. **Occurrence:** recordedBy: R. Kostova; individualCount: 1; **Location:** countryCode: BG; locality: Near "Kachul" Place; verbatimElevation: 91; verbatimCoordinates: N42°01'57.1", E27°37'12.3"; geodeticDatum: WGS84; **Event:** eventDate: 17/04/2009; habitat: meadow
- **Type status:** Other material. **Location:** countryCode: BG; locality: Strandzha; **Record Level:** bibliographicCitation: Rambousek (1912: 102)
- **Type status:** Other material. **Occurrence:** recordedBy: P. Beron; individualCount: 3; **Location:** countryCode: BG; locality: Kiten; **Event:** eventDate: 16-22.12.1984; **Record Level:** institutionCode: NMNHS
- **Type status:** Other material. **Occurrence:** recordedBy: B. Petrov; individualCount: 1; **Location:** countryCode: BG; locality: Harmanyata Place near Maharata Cave, close to Kosti Vill.; verbatimElevation: 160; verbatimCoordinates: N42°00'20.1", E27°49'25.1"; **Event:** eventDate: 26/09/2006; habitat: Quercus sp. litter; **Record Level:** institutionCode: NMNHS

#### Amblystomus metallescens

(Dejean, 1829)

##### Materials

- **Type status:** Other material. **Location:** countryCode: BG; locality: Maslen nos Cape; **Record Level:** bibliographicCitation: Guéorguiev & Guéorguiev (1995: 210)
- **Type status:** Other material. **Occurrence:** recordedBy: P. Beron; individualCount: 1; **Location:** countryCode: BG; locality: Kiten; **Event:** eventDate: 16-22.12.1984; **Record Level:** institutionCode: NMNHS

#### Amblystomus niger

(Heer, 1841)

##### Materials

- **Type status:** Other material. **Location:** countryCode: BG; locality: Tsarevo (= Micurin); **Record Level:** bibliographicCitation: Hieke & Wrase (1988: 146)
- **Type status:** Other material. **Location:** countryCode: BG; locality: Ropotamo; **Record Level:** bibliographicCitation: Hieke & Wrase (1988: 146)

#### Anisodactylus (Anisodactylus) binotatus

(Fabricius, 1787)

##### Materials

- **Type status:** Other material. **Occurrence:** recordedBy: R. Kostova; individualCount: 1; **Location:** countryCode: BG; locality: Slivarovo Vill., "Shafariitsa" Place; verbatimElevation: 224; verbatimCoordinates: N41°57'37.8", E27°39'34.8"; geodeticDatum: WGS84; **Event:** eventDate: 07/05/2009
- **Type status:** Other material. **Occurrence:** recordedBy: R. Bekchiev; individualCount: 1; **Location:** countryCode: BG; locality: Sinemorets Vill., PA"Silistar"; verbatimElevation: 23; verbatimCoordinates: N42°01'30.7", E28°00'25.4"; geodeticDatum: WGS84; **Event:** eventDate: 13/06/2009; habitat: oak forest
- **Type status:** Other material. **Occurrence:** recordedBy: R. Kostova; individualCount: 14; **Location:** countryCode: BG; locality: Sinemorets Vill., PA "Estuary of Veleka river"; verbatimElevation: 6; verbatimCoordinates: N42°03'39.6", E27°57'55.2"; geodeticDatum: WGS84; **Event:** eventDate: 15.04-02.07.2009; habitat: swamp forest
- **Type status:** Other material. **Occurrence:** recordedBy: R. Kostova; individualCount: 1; **Location:** countryCode: BG; locality: Sinemorets Vill., PA "Estuary of Veleka river"; verbatimElevation: 6; verbatimCoordinates: N42°03'39.6", E27°57'55.2"; geodeticDatum: WGS84; **Event:** eventDate: 03.08-07.09.2009; habitat: swamp forest
- **Type status:** Other material. **Location:** countryCode: BG; locality: Primorsko; **Record Level:** bibliographicCitation: Hieke & Wrase (1988: 112)
- **Type status:** Other material. **Location:** countryCode: BG; locality: Ropotamo; **Record Level:** bibliographicCitation: Hieke & Wrase (1988: 112)
- **Type status:** Other material. **Location:** countryCode: BG; locality: Strandzha; **Record Level:** bibliographicCitation: Guéorguiev & Guéorguiev (1995: 170)
- **Type status:** Other material. **Occurrence:** recordedBy: D. Iltchev; individualCount: 1; **Location:** countryCode: BG; locality: Kosti Vill.; **Event:** eventDate: 29/06/1921; **Record Level:** institutionCode: NMNHS
- **Type status:** Other material. **Occurrence:** recordedBy: B. Guéorguiev; individualCount: 1; **Location:** countryCode: BG; locality: Aydere River; verbatimElevation: 350-450; **Event:** eventDate: 26/05/1995; **Record Level:** institutionCode: NMNHS

#### Anisodactylus (Anisodactylus) pueli pueli

Schauberger, 1933

##### Materials

- **Type status:** Other material. **Location:** countryCode: BG; locality: Ahtopol; **Record Level:** bibliographicCitation: Wrase (1991: 12)
- **Type status:** Other material. **Location:** countryCode: BG; locality: Ahtopol; **Record Level:** bibliographicCitation: Guéorguiev & Guéorguiev (1995: 170, as A. pueli)
- **Type status:** Other material. **Occurrence:** recordedBy: R. Kostova; individualCount: 1; **Location:** countryCode: BG; locality: Brodilovo Vill, along Eleshnitsa River; verbatimElevation: 17; verbatimCoordinates: N42°05'35.4", E27°49'53.0"; geodeticDatum: WGS84; **Event:** eventDate: 04/07/2009

#### Anisodactylus (Pseudanisodactylus) signatus

(Panzer, 1796)

##### Materials

- **Type status:** Other material. **Occurrence:** recordedBy: W. Beier; individualCount: 1; **Location:** countryCode: BG; locality: Primorsko; **Event:** eventDate: 15/05/2003; **Record Level:** collectionCode: cWB
- **Type status:** Other material. **Occurrence:** recordedBy: R. Kostova; individualCount: 1; **Location:** countryCode: BG; locality: Kosti Vill., "St. Ilia" Place; verbatimElevation: 35; verbatimCoordinates: N42°03'23.2", E27°45'51.6"; geodeticDatum: WGS84; **Event:** eventDate: 09.06-02.07.2009; habitat: meadow with single trees

#### Diachromus germanus

(Linnaeus, 1758)

##### Materials

- **Type status:** Other material. **Occurrence:** recordedBy: A. Gijonova; individualCount: 1; **Location:** countryCode: TR; locality: Kiyiköy surroundings; verbatimElevation: 38; verbatimCoordinates: N41°39'25.7", E28°05'11.2"; geodeticDatum: WGS84; **Event:** eventDate: 24/05/2010
- **Type status:** Other material. **Occurrence:** recordedBy: R. Kostova; individualCount: 3; **Location:** countryCode: BG; locality: Sinemorets Vill., PA "Estuary of Veleka river"; verbatimElevation: 6; verbatimCoordinates: N42°03'39.6", E27°57'55.2"; geodeticDatum: WGS84; **Event:** eventDate: 09.06-02.07.2009; habitat: swamp forest
- **Type status:** Other material. **Location:** countryCode: BG; locality: Kiten; **Record Level:** bibliographicCitation: Hieke & Wrase (1988: 114)
- **Type status:** Other material. **Location:** countryCode: BG; locality: Ropotamo; **Record Level:** bibliographicCitation: Hieke & Wrase (1988: 114)
- **Type status:** Other material. **Occurrence:** recordedBy: P. Beron; individualCount: 1; **Location:** countryCode: BG; locality: Kiten; **Event:** eventDate: 16-22.12.1984; **Record Level:** institutionCode: NMNHS

#### Gynandromorphus etruscus etruscus

(Quensel, 1806)

##### Materials

- **Type status:** Other material. **Occurrence:** recordedBy: R. Kostova; individualCount: 1; **Location:** countryCode: BG; locality: Varovnik Vill., near the bridge at Fakiiska River; verbatimElevation: 125; verbatimCoordinates: N42°13'33.7", E27°11'33.7"; geodeticDatum: WGS84; **Event:** eventDate: 09/06/2009
- **Type status:** Other material. **Occurrence:** recordedBy: R. Kostova; individualCount: 1; **Location:** countryCode: BG; locality: Bulgari Vill., PA "Marina reka"; verbatimElevation: 172; verbatimCoordinates: N42°06'41.7", E27°45'53.0"; geodeticDatum: WGS84; **Event:** eventDate: 11/06/2009
- **Type status:** Other material. **Occurrence:** recordedBy: R. Kostova; individualCount: 3; **Location:** countryCode: TR; locality: Kiyiköy surroundings; verbatimElevation: 38; verbatimCoordinates: N41°39'25.7", E28°05'11.2"; geodeticDatum: WGS84; **Event:** eventDate: 24/05/2010
- **Type status:** Other material. **Occurrence:** recordedBy: R. Kostova; individualCount: 1; **Location:** countryCode: BG; locality: Kosti Vill., "St. Ilia" Place; verbatimElevation: 35; verbatimCoordinates: N42°03'23.2", E27°45'51.6"; geodeticDatum: WGS84; **Event:** eventDate: 09.06-02.07.2009; habitat: meadow with single trees
- **Type status:** Other material. **Occurrence:** recordedBy: J. Mařan & K. Taborsky; individualCount: 1; **Location:** countryCode: BG; locality: Maslen nos Cape; **Event:** eventDate: VI.1933; **Record Level:** institutionCode: NMP
- **Type status:** Other material. **Location:** countryCode: BG; locality: Kiten; **Record Level:** bibliographicCitation: Hieke & Wrase (1988: 113)
- **Type status:** Other material. **Location:** countryCode: BG; locality: Ropotamo; **Record Level:** bibliographicCitation: Hieke & Wrase (1988: 113)

#### Carterus (Carterus) dama

(P. Rossi, 1792)

##### Materials

- **Type status:** Other material. **Occurrence:** recordedBy: J. Mařan & K. Taborsky; individualCount: 4; **Location:** countryCode: BG; locality: Maslen nos Cape; **Event:** eventDate: VI.1933; **Record Level:** institutionCode: NMNHS & NMP
- **Type status:** Other material. **Location:** countryCode: BG; locality: Ahtopol; **Record Level:** bibliographicCitation: Hieke & Wrase (1988: 145)
- **Type status:** Other material. **Location:** countryCode: BG; locality: Ahtopol; **Record Level:** bibliographicCitation: Wrase (1989: 39)

#### Carterus (Carterus) gilvipes

(Piochard de la Brûlerie, 1873)

##### Materials

- **Type status:** Other material. **Location:** countryCode: BG; locality: Tsarevo (= Micurin); **Record Level:** bibliographicCitation: Wrase (1991: 16)

#### Carterus (Carterus) rufipes

(Chaudoir, 1843)

##### Materials

- **Type status:** Other material. **Location:** countryCode: BG; locality: Primorsko; **Record Level:** bibliographicCitation: Hieke & Wrase (1988: 144)
- **Type status:** Other material. **Occurrence:** recordedBy: K. Iwanow; individualCount: 1; **Location:** countryCode: BG; locality: "Vasiliko", Tsarevo (= Micurin); **Event:** eventDate: 23-28.05.1923; **Record Level:** institutionCode: NMNHS
- **Type status:** Other material. **Occurrence:** recordedBy: B. Guéorguiev; individualCount: 1; **Location:** countryCode: BG; locality: Ahtopol; **Event:** eventDate: 28/05/1995; **Record Level:** institutionCode: NMNHS

#### Carterus (Pristocarterus) angustus

(Ménétriés, 1832)

##### Materials

- **Type status:** Other material. **Location:** countryCode: BG; locality: Ahtopol; **Record Level:** bibliographicCitation: Hieke & Wrase (1988: 145, as C. longipennis Chaudoir)

#### Dixus clypeatus

(P. Rossi, 1790)

##### Materials

- **Type status:** Other material. **Location:** countryCode: BG; locality: Strandzha; **Record Level:** bibliographicCitation: Rambousek (1912: 81)

#### Dixus obscurus

(Dejean, 1825)

##### Materials

- **Type status:** Other material. **Occurrence:** recordedBy: R. Kostova; individualCount: 2; **Location:** countryCode: TR; locality: Along Demirköy - Kıyıköy road; verbatimElevation: 199; verbatimCoordinates: N41°39'43.2", E27°55'25.8"; geodeticDatum: WGS84; **Event:** eventDate: 24/05/2010
- **Type status:** Other material. **Occurrence:** recordedBy: R. Kostova; individualCount: 1; **Location:** countryCode: TR; locality: Sarpdere Vill., Dupnisa Cave surroundings; verbatimElevation: 440; verbatimCoordinates: N41°49'56.3", E27°33'24.0"; geodeticDatum: WGS84; **Event:** eventDate: 26/05/2011
- **Type status:** Other material. **Occurrence:** recordedBy: R. Kostova; individualCount: 1; **Location:** countryCode: BG; locality: Sinemorets Vill., PA"Silistar", "Butamyata" Place; verbatimElevation: 10; verbatimCoordinates: N42°03'11.2", E27°59'19.7"; geodeticDatum: WGS84; **Event:** eventDate: 09.05-08.06.2009; habitat: meadow, single shrubs
- **Type status:** Other material. **Location:** countryCode: BG; locality: "Vurchali" Place; **Record Level:** bibliographicCitation: Guéorguiev & Guéorguiev (1995: 209)
- **Type status:** Other material. **Location:** countryCode: BG; locality: Kosti Vill.; **Record Level:** bibliographicCitation: Guéorguiev & Guéorguiev (1995: 209)
- **Type status:** Other material. **Occurrence:** recordedBy: J. Mařan & K. Taborsky; individualCount: 3; **Location:** countryCode: BG; locality: Maslen nos Cape; **Event:** eventDate: VI.1933; **Record Level:** institutionCode: NMP
- **Type status:** Other material. **Location:** countryCode: BG; locality: Velika Papiya; **Record Level:** bibliographicCitation: Guéorguiev & Guéorguiev (1995: 209)
- **Type status:** Other material. **Location:** countryCode: BG; locality: Kiten; **Record Level:** bibliographicCitation: Hieke & Wrase (1988: 145)
- **Type status:** Other material. **Location:** countryCode: BG; locality: Tsarevo (= Micurin); **Record Level:** bibliographicCitation: Hieke & Wrase (1988: 145)
- **Type status:** Other material. **Location:** countryCode: BG; locality: Ropotamo; **Record Level:** bibliographicCitation: Hieke & Wrase (1988: 145)
- **Type status:** Other material. **Location:** countryCode: BG; locality: Rezovo Vill.; **Record Level:** bibliographicCitation: Guéorguiev & Guéorguiev (1995: 209)
- **Type status:** Other material. **Location:** countryCode: BG; locality: Rezovo Vill.; **Record Level:** bibliographicCitation: Guéorguiev & Guéorguiev (1995: 209)
- **Type status:** Other material. **Occurrence:** recordedBy: M. Josifov; individualCount: 2; **Location:** countryCode: BG; locality: Gramatikovo Vill.; **Event:** eventDate: 28/05/1973; **Record Level:** institutionCode: NMNHS
- **Type status:** Other material. **Occurrence:** recordedBy: B. Guéorguiev; individualCount: 2; **Location:** countryCode: BG; locality: Sinemorets Village, south bay; **Event:** eventDate: 27/05/1995; **Record Level:** institutionCode: NMNHS

#### Graniger cordicollis

(Audinet-Serville, 1821)

##### Materials

- **Type status:** Other material. **Location:** countryCode: BG; locality: Ahtopol; **Record Level:** bibliographicCitation: Hieke & Wrase (1988: 144)

#### Acinopus (Acinopus) picipes

(Olivier, 1795)

##### Materials

- **Type status:** Other material. **Occurrence:** recordedBy: R. Kostova; individualCount: 1; **Location:** countryCode: TR; locality: Kiyiköy surroundings; verbatimElevation: 32; verbatimCoordinates: N41°38'12.8", E28°04'08.7"; geodeticDatum: WGS84; **Event:** eventDate: 07/07/2009
- **Type status:** Other material. **Location:** countryCode: BG; locality: Malko Tarnovo; **Record Level:** bibliographicCitation: Guéorguiev & Guéorguiev (1995: 205)
- **Type status:** Other material. **Location:** countryCode: BG; locality: Malko Tarnovo; **Record Level:** bibliographicCitation: Guéorguiev & Guéorguiev (1995: 205)
- **Type status:** Other material. **Location:** countryCode: BG; locality: Kosti Vill.; **Record Level:** bibliographicCitation: Guéorguiev & Guéorguiev (1995: 205)
- **Type status:** Other material. **Location:** countryCode: BG; locality: Kalovo Vill.; **Record Level:** bibliographicCitation: Guéorguiev & Guéorguiev (1995: 205)
- **Type status:** Other material. **Occurrence:** recordedBy: J. Mařan & K. Taborsky; individualCount: 1; **Location:** countryCode: BG; locality: Maslen nos Cape; **Event:** eventDate: VI.1933; **Record Level:** institutionCode: NMP
- **Type status:** Other material. **Location:** countryCode: BG; locality: Ropotamo; **Record Level:** bibliographicCitation: Hieke & Wrase (1988: 143)
- **Type status:** Other material. **Occurrence:** recordedBy: D. Iltchev; individualCount: 1; **Location:** countryCode: BG; locality: Gramatikovo Vill.; **Event:** eventDate: 30/06/1921; **Record Level:** institutionCode: NMNHS

#### Acinopus (Oedematicus) megacephalus

(P. Rossi, 1794)

##### Materials

- **Type status:** Other material. **Occurrence:** recordedBy: D. Iltchev; individualCount: 1; **Location:** countryCode: BG; locality: Ahtopol; **Event:** eventDate: 12-15.07.1920; **Record Level:** institutionCode: NMNHS
- **Type status:** Other material. **Occurrence:** recordedBy: B. Guéorguiev; individualCount: 2; **Location:** countryCode: BG; locality: route Primorsko Town - Pismenovo Vill.; verbatimElevation: 50-90; **Event:** eventDate: 26/08/2004; fieldNumber: un; **Record Level:** institutionCode: NMNHS
- **Type status:** Other material. **Location:** countryCode: BG; locality: Novo Panicharevo Vill.; **Record Level:** bibliographicCitation: Guéorguiev & Guéorguiev (1995: 205)
- **Type status:** Other material. **Location:** countryCode: BG; locality: Rezovo Vill.; **Record Level:** bibliographicCitation: Guéorguiev & Guéorguiev (1995: 205)
- **Type status:** Other material. **Location:** countryCode: BG; locality: Rezovo (= Novo selo); **Record Level:** bibliographicCitation: Guéorguiev & Guéorguiev (1995: 205)
- **Type status:** Other material. **Occurrence:** recordedBy: J. Mařan & K. Taborsky; individualCount: 1; **Location:** countryCode: BG; locality: Maslen nos Cape; **Event:** eventDate: VI.1933; **Record Level:** institutionCode: NMP
- **Type status:** Other material. **Location:** countryCode: BG; locality: Kiten; **Record Level:** bibliographicCitation: Hieke & Wrase (1988: 144)
- **Type status:** Other material. **Location:** countryCode: BG; locality: Ropotamo; **Record Level:** bibliographicCitation: Hieke & Wrase (1988: 144)

##### Notes

1 s. from Novo Panicharevo belongs to this species (published as A. picipes by Guéorguiev & Guéorguiev, 1995: 205)

#### Acinopus (Osimus) ammophilus

Dejean, 1829

##### Materials

- **Type status:** Other material. **Occurrence:** recordedBy: J. Mařan & K. Taborsky; individualCount: 1; **Location:** countryCode: BG; locality: Maslen nos Cape; **Event:** eventDate: VI.1933; **Record Level:** institutionCode: NMP
- **Type status:** Other material. **Location:** countryCode: BG; locality: Ahtopol; **Record Level:** bibliographicCitation: Hieke & Wrase (1988: 143)
- **Type status:** Other material. **Occurrence:** recordedBy: Z. Hubenov; individualCount: 1; **Location:** countryCode: BG; locality: Ahtopol; **Event:** eventDate: 29.07-12.08.1976; **Record Level:** institutionCode: NMNHS

#### Harpalus (Cephalophonus) cephalotes cephalotes

Fairmaire & Laboulbène, 1854

##### Materials

- **Type status:** Other material. **Location:** countryCode: BG; locality: Ropotamo; **Record Level:** bibliographicCitation: Hieke & Wrase (1988: 120, as Ophonus cephalotes)

#### Harpalus (Cryptophonus) melancholicus melancholicus

Dejean, 1829

##### Materials

- **Type status:** Other material. **Location:** countryCode: BG; locality: Ahtopol; **Record Level:** bibliographicCitation: Hieke & Wrase (1988: 137)

#### Harpalus (Cryptophonus) tenebrosus tenebrosus

Dejean, 1829

##### Materials

- **Type status:** Other material. **Occurrence:** recordedBy: R. Bekchiev; individualCount: 1; **Location:** countryCode: TR; locality: Sarpdere Vill., Dupnisa Cave surroundings; verbatimElevation: 440; verbatimCoordinates: N41°49'56.3", E27°33'24.0"; geodeticDatum: WGS84; **Event:** eventDate: 08/07/2009
- **Type status:** Other material. **Occurrence:** recordedBy: R. Kostova; individualCount: 1; **Location:** countryCode: BG; locality: Kosti Vill., "St. Ilia" Place; verbatimElevation: 35; verbatimCoordinates: N42°03'23.2", E27°45'51.6"; geodeticDatum: WGS84; **Event:** eventDate: 03.07-02.08.2009; habitat: meadow with single trees
- **Type status:** Other material. **Location:** countryCode: BG; locality: Ahtopol; **Record Level:** bibliographicCitation: Hieke & Wrase (1988: 138)

#### Harpalus (Harpalus) affinis

(Schrank, 1781)

##### Materials

- **Type status:** Other material. **Occurrence:** recordedBy: R. Kostova; individualCount: 1; **Location:** countryCode: BG; locality: Kosti Vill., "St. Ilia" Place; verbatimElevation: 35; verbatimCoordinates: N42°03'23.2", E27°45'51.6"; geodeticDatum: WGS84; **Event:** eventDate: 09.06-02.07.2009; habitat: meadow with single trees
- **Type status:** Other material. **Location:** countryCode: BG; locality: Ahtopol; **Record Level:** bibliographicCitation: Hieke & Wrase (1988: 129, as Harpalus aeneus F.)
- **Type status:** Other material. **Location:** countryCode: BG; locality: Kiten; **Record Level:** bibliographicCitation: Hieke & Wrase (1988: 129, as Harpalus aeneus F.)
- **Type status:** Other material. **Occurrence:** recordedBy: P. Petcoff; individualCount: 4; **Location:** countryCode: BG; locality: Kosti Vill.; **Event:** eventDate: 29-30.04.1921; **Record Level:** institutionCode: NMNHS
- **Type status:** Other material. **Occurrence:** recordedBy: K. Tuleshkov; individualCount: 1; **Location:** countryCode: BG; locality: Maslen nos Cape (= Zejtinburun); **Event:** eventDate: 16/07/1933; **Record Level:** institutionCode: NMNHS

#### Harpalus (Harpalus) albanicus

Reitter, 1900

##### Materials

- **Type status:** Other material. **Occurrence:** recordedBy: R. Kostova; individualCount: 1; **Location:** countryCode: BG; locality: Slivarovo Vill., "Shafariitsa" Place; verbatimElevation: 224; verbatimCoordinates: N41°57'37.8", E27°39'34.8"; geodeticDatum: WGS84; **Event:** eventDate: 15.04-08.05.2009; habitat: black alder-oak forest
- **Type status:** Other material. **Occurrence:** recordedBy: R. Kostova; individualCount: 1; **Location:** countryCode: BG; locality: Kosti Vill., "St. Ilia" Place; verbatimElevation: 35; verbatimCoordinates: N42°03'23.2", E27°45'51.6"; geodeticDatum: WGS84; **Event:** eventDate: 09.05-08.06.2009; habitat: meadow with single trees
- **Type status:** Other material. **Location:** countryCode: BG; locality: Primorsko; **Record Level:** bibliographicCitation: Hieke & Wrase (1988: 134)

#### Harpalus (Harpalus) amplicollis

Ménétriés, 1848

##### Materials

- **Type status:** Other material. **Location:** countryCode: BG; locality: Varvara Vill.; **Record Level:** bibliographicCitation: Hieke & Wrase (1988: 140)

#### Harpalus (Harpalus) atratus

Latreille, 1804

##### Materials

- **Type status:** Other material. **Occurrence:** recordedBy: R. Kostova; individualCount: 1; **Location:** countryCode: TR; locality: Kizilagač, along river; verbatimElevation: 115; verbatimCoordinates: N41°41'01.9", E27°52'51.4"; geodeticDatum: WGS84; **Event:** eventDate: 24/05/2011
- **Type status:** Other material. **Occurrence:** recordedBy: R. Kostova; individualCount: 1; **Location:** countryCode: BG; locality: Bliznak Vill., PA"Bataka"; verbatimElevation: 324; verbatimCoordinates: N42°11'37.3", E27°19'35.8"; geodeticDatum: WGS84; **Event:** eventDate: 09.06-02.07.2009; habitat: oak forest
- **Type status:** Other material. **Occurrence:** recordedBy: R. Kostova; individualCount: 9; **Location:** countryCode: BG; locality: Malko Tarnovo, "Propada" Place; verbatimElevation: 401; verbatimCoordinates: N41°58'49.6", E27°29'25.7"; geodeticDatum: WGS84; **Event:** eventDate: 09.05-08.06.2009; habitat: oriental beech forest
- **Type status:** Other material. **Occurrence:** recordedBy: R. Kostova; individualCount: 1; **Location:** countryCode: BG; locality: Malko Tarnovo, "Propada" Place; verbatimElevation: 401; verbatimCoordinates: N41°58'49.6", E27°29'25.7"; geodeticDatum: WGS84; **Event:** eventDate: 03.08-07.09.2009; habitat: oriental beech forest
- **Type status:** Other material. **Occurrence:** recordedBy: R. Kostova; individualCount: 12; **Location:** countryCode: BG; locality: Slivarovo Vill., "Shafariitsa" Place; verbatimElevation: 224; verbatimCoordinates: N41°57'37.8", E27°39'34.8"; geodeticDatum: WGS84; **Event:** eventDate: 09.05-02.08.2009; habitat: black alder-oak forest
- **Type status:** Other material. **Occurrence:** recordedBy: R. Kostova; individualCount: 10; **Location:** countryCode: BG; locality: Kondolovo Vill., "Byalata prast" Place; verbatimElevation: 271; verbatimCoordinates: N42°05'47.7", E27°39'55.0"; geodeticDatum: WGS84; **Event:** eventDate: 15.04-02.07.2009; habitat: oak-beech mixed forest
- **Type status:** Other material. **Occurrence:** recordedBy: R. Kostova; individualCount: 4; **Location:** countryCode: BG; locality: Mladezhko Vill., The springs of Mladezhka River; verbatimElevation: 231; verbatimCoordinates: N42°09'04.5", E27°21'26.1"; geodeticDatum: WGS84; **Event:** eventDate: 09.05-02.08.2009; habitat: black alder and hornbeam forest
- **Type status:** Other material. **Location:** countryCode: BG; locality: Kalovo Vill.; **Record Level:** bibliographicCitation: Guéorguiev & Guéorguiev (1995: 197)
- **Type status:** Other material. **Occurrence:** recordedBy: P. Petcoff; individualCount: 1; **Location:** countryCode: BG; locality: Kosti Vill.; **Event:** eventDate: 29-30.04.1921; **Record Level:** institutionCode: NMNHS
- **Type status:** Other material. **Occurrence:** recordedBy: B. Guéorguiev; individualCount: 3; **Location:** countryCode: BG; locality: Veleka River near Mladezko Village; **Event:** eventDate: 25/05/1995; **Record Level:** institutionCode: NMNHS

#### Harpalus (Harpalus) attenuatus

Stephens, 1828

##### Materials

- **Type status:** Other material. **Occurrence:** recordedBy: R. Kostova; individualCount: 1; **Location:** countryCode: BG; locality: Malko Tarnovo, "Propada" Place; verbatimElevation: 388; verbatimCoordinates: N41°58'52.5", E27°29'28.6"; geodeticDatum: WGS84; **Event:** eventDate: 17/04/2009; habitat: meadow
- **Type status:** Other material. **Occurrence:** recordedBy: R. Kostova; individualCount: 1; **Location:** countryCode: TR; locality: Sarpdere Vill., Dupnisa Cave surroundings; verbatimElevation: 356; verbatimCoordinates: N41°50'26.0", E27°33'22.4"; geodeticDatum: WGS84; **Event:** eventDate: 23/05/2010
- **Type status:** Other material. **Occurrence:** recordedBy: R. Kostova; individualCount: 1; **Location:** countryCode: TR; locality: Kiyiköy surroundings; verbatimElevation: 38; verbatimCoordinates: N41°39'25.7", E28°05'11.2"; geodeticDatum: WGS84; **Event:** eventDate: 22/05/2011
- **Type status:** Other material. **Occurrence:** recordedBy: R. Kostova; individualCount: 6; **Location:** countryCode: BG; locality: Slivarovo Vll. surroundings; verbatimElevation: 331; verbatimCoordinates: N41°58'11.9", E27°39'31.1"; geodeticDatum: WGS84; **Event:** eventDate: 09.05-07.09.2009; habitat: shrubs, mainly Calluna vulgaris
- **Type status:** Other material. **Occurrence:** recordedBy: R. Kostova; individualCount: 1; **Location:** countryCode: BG; locality: Kosti Vill., "St. Ilia" Place; verbatimElevation: 35; verbatimCoordinates: N42°03'23.2", E27°45'51.6"; geodeticDatum: WGS84; **Event:** eventDate: 09.06-02.07.2009; habitat: meadow with single trees
- **Type status:** Other material. **Location:** countryCode: BG; locality: Kiten; **Record Level:** bibliographicCitation: Hieke & Wrase (1988: 139)
- **Type status:** Other material. **Location:** countryCode: BG; locality: Tsarevo (= Micurin); **Record Level:** bibliographicCitation: Hieke & Wrase (1988: 139)
- **Type status:** Other material. **Location:** countryCode: BG; locality: Primorsko; **Record Level:** bibliographicCitation: Hieke & Wrase (1988: 139)
- **Type status:** Other material. **Location:** countryCode: BG; locality: Varvara Vill.; **Record Level:** bibliographicCitation: Hieke & Wrase (1988: 139)
- **Type status:** Other material. **Occurrence:** recordedBy: D. Iltchev; individualCount: 1; **Location:** countryCode: BG; locality: Bulgari Vill.; **Event:** eventDate: 28.05-01.06.1923; **Record Level:** institutionCode: NMNHS

#### Harpalus (Harpalus) autumnalis

(Duftschmid, 1812)

##### Materials

- **Type status:** Other material. **Occurrence:** recordedBy: R. Kostova; individualCount: 1; **Location:** countryCode: BG; locality: Primorsko, Dunes; verbatimElevation: 6; verbatimCoordinates: N41°47'44.0", E27°59'50.4"; geodeticDatum: WGS84; **Event:** eventDate: 10/05/2009
- **Type status:** Other material. **Occurrence:** recordedBy: R. Kostova; individualCount: 1; **Location:** countryCode: BG; locality: Kosti Vill., "St. Ilia" Place; verbatimElevation: 35; verbatimCoordinates: N42°03'23.2", E27°45'51.6"; geodeticDatum: WGS84; **Event:** eventDate: 09.06-02.07.2009
- **Type status:** Other material. **Location:** countryCode: BG; locality: Tsarevo (= Micurin); **Record Level:** bibliographicCitation: Hieke & Wrase (1988: 136)
- **Type status:** Other material. **Location:** countryCode: BG; locality: Primorsko; **Record Level:** bibliographicCitation: Hieke & Wrase (1988: 136)
- **Type status:** Other material. **Location:** countryCode: BG; locality: Ropotamo; **Record Level:** bibliographicCitation: Hieke & Wrase (1988: 136)

#### Harpalus (Harpalus) cupreus fastuosus

Faldermann, 1836

##### Materials

- **Type status:** Other material. **Occurrence:** recordedBy: R. Kostova; individualCount: 1; **Location:** countryCode: TR; locality: Kiyiköy surroundings; verbatimElevation: 32; verbatimCoordinates: N41°38'12.8", E28°04'08.7"; geodeticDatum: WGS84; **Event:** eventDate: 22/05/2011
- **Type status:** Other material. **Occurrence:** recordedBy: R. Kostova; individualCount: 20; **Location:** countryCode: BG; locality: Kosti Vill., "St. Ilia" Place; verbatimElevation: 35; verbatimCoordinates: N42°03'23.2", E27°45'51.6"; geodeticDatum: WGS84; **Event:** eventDate: 15.04-02.08.2009; habitat: meadow with single trees
- **Type status:** Other material. **Occurrence:** recordedBy: W. Beier; individualCount: 1; **Location:** countryCode: BG; locality: Mouth of Veleka River near Sinemorets Vill.; verbatimElevation: 3; verbatimCoordinates: N42°03'51.0", E27°58'14.0"; geodeticDatum: WGS84; **Event:** eventDate: 27/04/2001; **Record Level:** collectionCode: cWB
- **Type status:** Other material. **Location:** countryCode: BG; locality: Ropotamo; **Record Level:** bibliographicCitation: Hieke & Wrase (1988: 139)

#### Harpalus (Harpalus) dimidiatus

(P. Rossi, 1790)

##### Materials

- **Type status:** Other material. **Occurrence:** recordedBy: R. Kostova; individualCount: 1; **Location:** countryCode: BG; locality: Near "Kachul" Place; verbatimElevation: 91; verbatimCoordinates: N42°01'57.1", E27°37'12.3"; geodeticDatum: WGS84; **Event:** eventDate: 17/04/2009; habitat: meadow
- **Type status:** Other material. **Occurrence:** recordedBy: R. Kostova; individualCount: 1; **Location:** countryCode: BG; locality: Kosti Vill., "St. Ilia" Place; verbatimElevation: 35; verbatimCoordinates: N42°03'23.2", E27°45'51.6"; geodeticDatum: WGS84; **Event:** eventDate: 19/04/2009
- **Type status:** Other material. **Occurrence:** recordedBy: R. Kostova; individualCount: 1; **Location:** countryCode: BG; locality: Malko Tarnovo, the Tracian tumbs; verbatimElevation: 383; verbatimCoordinates: N41°58'54.6", E27°29'27.8"; geodeticDatum: WGS84; **Event:** eventDate: 27/05/2010
- **Type status:** Other material. **Occurrence:** recordedBy: R. Kostova; individualCount: 1; **Location:** countryCode: BG; locality: Malko Tarnovo, Entrance to Vitanovo Reserve; verbatimElevation: 285; verbatimCoordinates: N42°01'22.9", E27°27'22.9"; geodeticDatum: WGS84; **Event:** eventDate: 27/05/2010
- **Type status:** Other material. **Occurrence:** recordedBy: R. Kostova; individualCount: 1; **Location:** countryCode: BG; locality: Malko Tarnovo, "Propada" Place; verbatimElevation: 401; verbatimCoordinates: N41°58'49.6", E27°29'25.7"; geodeticDatum: WGS84; **Event:** eventDate: 09.05-08.06.2009; habitat: oriental beech forest
- **Type status:** Other material. **Occurrence:** recordedBy: R. Kostova; individualCount: 1; **Location:** countryCode: BG; locality: Slivarovo Vll. surroundings; verbatimElevation: 331; verbatimCoordinates: N41°58'11.9", E27°39'31.1"; geodeticDatum: WGS84; **Event:** eventDate: 15.04-08.05.2009; habitat: shrubs, mainly Calluna vulgaris
- **Type status:** Other material. **Occurrence:** recordedBy: R. Kostova; individualCount: 1; **Location:** countryCode: BG; locality: Slivarovo Vll. surroundings; verbatimElevation: 331; verbatimCoordinates: N41°58'11.9", E27°39'31.1"; geodeticDatum: WGS84; **Event:** eventDate: 09.06-02.07.2009; habitat: shrubs, mainly Calluna vulgaris
- **Type status:** Other material. **Occurrence:** recordedBy: R. Kostova; individualCount: 13; **Location:** countryCode: BG; locality: Kosti Vill., "St. Ilia" Place; verbatimElevation: 35; verbatimCoordinates: N42°03'23.2", E27°45'51.6"; geodeticDatum: WGS84; **Event:** eventDate: 15.04-08.06.2009; habitat: meadow with single trees
- **Type status:** Other material. **Occurrence:** recordedBy: R. Kostova; individualCount: 4; **Location:** countryCode: BG; locality: Kosti Vill., "St. Ilia" Place; verbatimElevation: 35; verbatimCoordinates: N42°03'23.2", E27°45'51.6"; geodeticDatum: WGS84; **Event:** eventDate: 03.07-02.08.2009; habitat: meadow with single trees
- **Type status:** Other material. **Occurrence:** recordedBy: R. Kostova; individualCount: 1; **Location:** countryCode: BG; locality: Bulgari Vill., PA "Marina reka"; verbatimElevation: 183; verbatimCoordinates: N42°06'41.7", E27°45'53.0"; geodeticDatum: WGS84; **Event:** eventDate: 09.05-08.06.2009; habitat: oriental beech-oak forest with Rododendron ponticum
- **Type status:** Other material. **Occurrence:** recordedBy: R. Kostova; individualCount: 5; **Location:** countryCode: BG; locality: Stoilovo Vill., "Petrova niva" Place; verbatimElevation: 234; verbatimCoordinates: N42°03'40.3", E27°31'42.3"; geodeticDatum: WGS84; **Event:** eventDate: 09.05-02.07.2009; habitat: meadow, single shrubs
- **Type status:** Other material. **Occurrence:** recordedBy: J. Mařan & K. Taborsky; individualCount: 1; **Location:** countryCode: BG; locality: Maslen nos Cape; **Event:** eventDate: VI.1933; **Record Level:** institutionCode: NMP (det. as Ab. depressus Duft.)
- **Type status:** Other material. **Location:** countryCode: BG; locality: Kiten; **Record Level:** bibliographicCitation: Hieke & Wrase (1988: 132)
- **Type status:** Other material. **Location:** countryCode: BG; locality: Tsarevo (= Micurin); **Record Level:** bibliographicCitation: Hieke & Wrase (1988: 132)
- **Type status:** Other material. **Location:** countryCode: BG; locality: Primorsko; **Record Level:** bibliographicCitation: Hieke & Wrase (1988: 132)
- **Type status:** Other material. **Location:** countryCode: BG; locality: Ropotamo; **Record Level:** bibliographicCitation: Hieke & Wrase (1988: 132)
- **Type status:** Other material. **Occurrence:** recordedBy: P. Petcoff; individualCount: 1; **Location:** countryCode: BG; locality: Malko Tarnovo; **Event:** eventDate: 3-5.05.1921; **Record Level:** institutionCode: NMNHS
- **Type status:** Other material. **Occurrence:** recordedBy: D. Iltchev; individualCount: 1; **Location:** countryCode: BG; locality: Vizitsa Vill.; **Event:** eventDate: 1-7.06.1923; **Record Level:** institutionCode: NMNHS
- **Type status:** Other material. **Occurrence:** recordedBy: B. Guéorguiev; individualCount: 1; **Location:** countryCode: BG; locality: Veleka River near Mladezko Village; **Event:** eventDate: 25/05/1995; **Record Level:** institutionCode: NMNHS

#### Harpalus (Harpalus) distinguendus distinguendus

(Duftschmid, 1812)

##### Materials

- **Type status:** Other material. **Occurrence:** recordedBy: R. Kostova; individualCount: 1; **Location:** countryCode: BG; locality: Slivarovo Village; verbatimElevation: 338; verbatimCoordinates: N41°58'11.9", E27°39'31.1"; geodeticDatum: WGS84; **Event:** eventDate: 19/04/2009
- **Type status:** Other material. **Location:** countryCode: BG; locality: Kiten; **Record Level:** bibliographicCitation: Hieke & Wrase (1988: 131)
- **Type status:** Other material. **Location:** countryCode: BG; locality: Tsarevo (= Micurin); **Record Level:** bibliographicCitation: Hieke & Wrase (1988: 131)
- **Type status:** Other material. **Occurrence:** recordedBy: D. Iltchev; individualCount: 1; **Location:** countryCode: BG; locality: Brodilovo Vill.; **Event:** eventDate: 29/05/1923; **Record Level:** institutionCode: NMNHS
- **Type status:** Other material. **Occurrence:** recordedBy: P. Beron; individualCount: 1; **Location:** countryCode: BG; locality: Kiten; **Event:** eventDate: 16-22.12.1984; **Record Level:** institutionCode: NMNHS
- **Type status:** Other material. **Occurrence:** recordedBy: B. Guéorguiev; individualCount: 1; **Location:** countryCode: BG; locality: Arapya, near Tsarevo; **Event:** eventDate: 28/05/1995; **Record Level:** institutionCode: NMNHS

#### Harpalus (Harpalus) flavicornis flavicornis

Dejean, 1829

##### Materials

- **Type status:** Other material. **Occurrence:** recordedBy: R. Kostova; individualCount: 2; **Location:** countryCode: BG; locality: Stoilovo Vill., "Petrova niva" Place; verbatimElevation: 132; verbatimCoordinates: N42°03'41.9", E27°31'58.9"; geodeticDatum: WGS84; **Event:** eventDate: 06/05/2010; habitat: meadow, single shrubs
- **Type status:** Other material. **Occurrence:** recordedBy: R. Kostova; individualCount: 1; **Location:** countryCode: TR; locality: Sarpdere Vill., Dupnisa Cave surroundings; verbatimElevation: 356; verbatimCoordinates: N41°50'26.0", E27°33'22.4"; geodeticDatum: WGS84; **Event:** eventDate: 23/05/2010
- **Type status:** Other material. **Occurrence:** recordedBy: R. Kostova; individualCount: 2; **Location:** countryCode: BG; locality: Slivarovo Vll. surroundings; verbatimElevation: 331; verbatimCoordinates: N41°58'11.9", E27°39'31.1"; geodeticDatum: WGS84; **Event:** eventDate: 09.05-08.06.2009; habitat: shrubs, mainly Calluna vulgaris
- **Type status:** Other material. **Occurrence:** recordedBy: R. Kostova; individualCount: 2; **Location:** countryCode: BG; locality: Slivarovo Vll. surroundings; verbatimElevation: 331; verbatimCoordinates: N41°58'11.9", E27°39'31.1"; geodeticDatum: WGS84; **Event:** eventDate: 03.07-02.08.2009; habitat: shrubs, mainly Calluna vulgaris
- **Type status:** Other material. **Occurrence:** recordedBy: R. Kostova; individualCount: 2; **Location:** countryCode: BG; locality: Slivarovo Vill., "Shafariitsa" Place; verbatimElevation: 224; verbatimCoordinates: N41°57'37.8", E27°39'34.8"; geodeticDatum: WGS84; **Event:** eventDate: 07.05-08.06.2009; habitat: black alder-oak forest
- **Type status:** Other material. **Occurrence:** recordedBy: R. Kostova; individualCount: 2; **Location:** countryCode: BG; locality: Stoilovo Vill., "Petrova niva" Place; verbatimElevation: 234; verbatimCoordinates: N42°03'40.3", E27°31'42.3"; geodeticDatum: WGS84; **Event:** eventDate: 09.05-02.07.2009; habitat: meadow, single shrubs
- **Type status:** Other material. **Occurrence:** recordedBy: V. Novotny; individualCount: 4; **Location:** countryCode: BG; locality: Stomoplo; **Event:** eventDate: VI.1967; **Record Level:** institutionCode: NMP
- **Type status:** Other material. **Location:** countryCode: BG; locality: Kiten; **Record Level:** bibliographicCitation: Hieke & Wrase (1988: 136)
- **Type status:** Other material. **Location:** countryCode: BG; locality: Tsarevo (= Micurin); **Record Level:** bibliographicCitation: Hieke & Wrase (1988: 136)
- **Type status:** Other material. **Location:** countryCode: BG; locality: Primorsko; **Record Level:** bibliographicCitation: Hieke & Wrase (1988: 136)
- **Type status:** Other material. **Location:** countryCode: BG; locality: Ropotamo; **Record Level:** bibliographicCitation: Hieke & Wrase (1988: 136)
- **Type status:** Other material. **Occurrence:** recordedBy: B. Guéorguiev; individualCount: 1; **Location:** countryCode: BG; locality: Tisovitsa Reserve near Bulgari Vill.; **Event:** eventDate: 27/05/1995; **Record Level:** institutionCode: NMNHS

#### Harpalus (Harpalus) froelichii

Sturm, 1818

##### Materials

- **Type status:** Other material. **Occurrence:** recordedBy: R. Kostova; individualCount: 1; **Location:** countryCode: BG; locality: Sinemorets Vill., PA"Silistar", "Butamyata" Place; verbatimElevation: 17; verbatimCoordinates: N42°03'10.3", E27°59'13.4"; geodeticDatum: WGS84; **Event:** eventDate: 03.08-07.09.2009; habitat: oak forest
- **Type status:** Other material. **Occurrence:** recordedBy: J. Mařan & K. Taborsky; individualCount: 1; **Location:** countryCode: BG; locality: Maslen nos Cape; **Event:** eventDate: VI.1933; **Record Level:** institutionCode: NMP
- **Type status:** Other material. **Location:** countryCode: BG; locality: Ahtopol; **Record Level:** bibliographicCitation: Hieke & Wrase (1988: 129)
- **Type status:** Other material. **Location:** countryCode: BG; locality: Tsarevo (= Micurin); **Record Level:** bibliographicCitation: Hieke & Wrase (1988: 129)

#### Harpalus (Harpalus) honestus

(Duftschmid, 1812)

##### Materials

- **Type status:** Other material. **Occurrence:** recordedBy: R. Kostova; individualCount: 4; **Location:** countryCode: BG; locality: Brodilovo Vill., under Papiya peak; verbatimElevation: 296; verbatimCoordinates: N42°06'21.7", E27°50'32.6"; geodeticDatum: WGS84; **Event:** eventDate: 09.05-08.06.2009; habitat: shrubs, Phyllyrea latifolia
- **Type status:** Other material. **Occurrence:** recordedBy: R. Kostova; individualCount: 1; **Location:** countryCode: BG; locality: Brodilovo Vill., under Papiya peak; verbatimElevation: 296; verbatimCoordinates: N42°06'21.7", E27°50'32.6"; geodeticDatum: WGS84; **Event:** eventDate: 09.06-02.07.2009; habitat: shrubs, Phyllyrea latifolia
- **Type status:** Other material. **Occurrence:** recordedBy: R. Kostova; individualCount: 1; **Location:** countryCode: BG; locality: Sinemorets Vill., PA"Silistar", "Butamyata" Place; verbatimElevation: 17; verbatimCoordinates: N42°03'10.3", E27°59'13.4"; geodeticDatum: WGS84; **Event:** eventDate: 09.05-08.06.2009; habitat: oak forest
- **Type status:** Other material. **Location:** countryCode: BG; locality: Strandzha; **Record Level:** bibliographicCitation: Guéorguiev & Guéorguiev (1995: 198)

#### Harpalus (Harpalus) hospes hospes

Sturm, 1818

##### Materials

- **Type status:** Other material. **Location:** countryCode: BG; locality: Tsarevo (= Micurin); **Record Level:** bibliographicCitation: Hieke & Wrase (1988: 128)

#### Harpalus (Harpalus) pumilus

Sturm, 1818

##### Materials

- **Type status:** Other material. **Location:** countryCode: BG; locality: Ahtopol; **Record Level:** bibliographicCitation: Hieke & Wrase (1988: 141)
- **Type status:** Other material. **Location:** countryCode: BG; locality: Kiten; **Record Level:** bibliographicCitation: Hieke & Wrase (1988: 141)

#### Harpalus (Harpalus) punctatostriatus

Dejean, 1829

##### Materials

- **Type status:** Other material. **Location:** countryCode: BG; locality: Ropotamo; **Record Level:** bibliographicCitation: Hieke & Wrase (1988: 128)

#### Harpalus (Harpalus) pygmaeus

Dejean, 1829

##### Materials

- **Type status:** Other material. **Occurrence:** recordedBy: R. Kostova; individualCount: 1; **Location:** countryCode: BG; locality: Sinemorets Vill., PA"Silistar", "Butamyata" Place; verbatimElevation: 10; verbatimCoordinates: N42°03'11.2", E27°59'19.7"; geodeticDatum: WGS84; **Event:** eventDate: 15.04-08.05.2009; habitat: meadow, single shrubs
- **Type status:** Other material. **Location:** countryCode: BG; locality: Strandzha; **Record Level:** bibliographicCitation: Rambousek (1912: 84)
- **Type status:** Other material. **Location:** countryCode: BG; locality: Kiten; **Record Level:** bibliographicCitation: Hieke & Wrase (1988: 139)

#### Harpalus (Harpalus) rubripes

(Duftschmid, 1812)

##### Materials

- **Type status:** Other material. **Occurrence:** recordedBy: R. Kostova; individualCount: 1; **Location:** countryCode: BG; locality: Kosti Vill., "St. Ilia" Place; verbatimElevation: 35; verbatimCoordinates: N42°03'23.2", E27°45'51.6"; geodeticDatum: WGS84; **Event:** eventDate: 09.05-08.06.2009; habitat: meadow with single trees
- **Type status:** Other material. **Occurrence:** recordedBy: R. Kostova; individualCount: 1; **Location:** countryCode: BG; locality: Sinemorets Vill., PA"Silistar", "Butamyata" Place; verbatimElevation: 17; verbatimCoordinates: N42°03'10.3", E27°59'13.4"; geodeticDatum: WGS84; **Event:** eventDate: 03.07-02.08.2009; habitat: oak forest
- **Type status:** Other material. **Occurrence:** recordedBy: J. Mařan & K. Taborsky; individualCount: 1; **Location:** countryCode: BG; locality: Maslen nos Cape; **Event:** eventDate: VI.1933; **Record Level:** institutionCode: NMP
- **Type status:** Other material. **Location:** countryCode: BG; locality: Kiten; **Record Level:** bibliographicCitation: Hieke & Wrase (1988: 133)
- **Type status:** Other material. **Location:** countryCode: BG; locality: Tsarevo (= Micurin); **Record Level:** bibliographicCitation: Hieke & Wrase (1988: 133)
- **Type status:** Other material. **Occurrence:** recordedBy: P. Petcoff; individualCount: 1; **Location:** countryCode: BG; locality: Primorsko (= Kyupriya); **Event:** eventDate: 27/04/1921; **Record Level:** institutionCode: NMNHS
- **Type status:** Other material. **Occurrence:** recordedBy: B. Guéorguiev; individualCount: 1; **Location:** countryCode: BG; locality: Arapya, near Tsarevo; **Event:** eventDate: 28/05/1995; **Record Level:** institutionCode: NMNHS

#### Harpalus (Harpalus) rufipalpis rufipalpis

Sturm, 1818

##### Materials

- **Type status:** Other material. **Occurrence:** recordedBy: R. Kostova; individualCount: 1; **Location:** countryCode: BG; locality: Stoilovo Vill., "Sredoka" Reserve, along Mechi dol River; verbatimElevation: 206; verbatimCoordinates: N42°01'51.1", E27°30'50.1"; geodeticDatum: WGS84; **Event:** eventDate: 19/04/2009
- **Type status:** Other material. **Occurrence:** recordedBy: R. Kostova; individualCount: 1; **Location:** countryCode: BG; locality: Slivarovo Vll. surroundings; verbatimElevation: 331; verbatimCoordinates: N41°58'11.9", E27°39'31.1"; geodeticDatum: WGS84; **Event:** eventDate: 15.04-08.05.2009; habitat: shrubs, mainly Calluna vulgaris
- **Type status:** Other material. **Occurrence:** recordedBy: P. Drenski; individualCount: 1; **Location:** countryCode: BG; locality: Rezovo; **Event:** eventDate: 11/06/1933; **Record Level:** institutionCode: NMNHS
- **Type status:** Other material. **Occurrence:** recordedBy: K. Tuleshkov; individualCount: 2; **Location:** countryCode: BG; locality: Maslen nos Cape (= Zejtinburun); **Event:** eventDate: 16/07/1933; **Record Level:** institutionCode: NMNHS
- **Type status:** Other material. **Occurrence:** recordedBy: B. Guéorguiev; individualCount: 1; **Location:** countryCode: BG; locality: Aydere River; verbatimElevation: 350-450; **Event:** eventDate: 26/05/1995; **Record Level:** institutionCode: NMNHS

#### Harpalus (Harpalus) saxicola

Dejean, 1829

##### Materials

- **Type status:** Other material. **Location:** countryCode: BG; locality: Ropotamo; **Record Level:** bibliographicCitation: Hieke & Wrase (1988: 130)
- **Type status:** Other material. **Occurrence:** recordedBy: W. Beier; individualCount: 1; **Location:** countryCode: BG; locality: Stoilovo Vill.; verbatimElevation: 340; verbatimCoordinates: N42°02'23.0", E27°31'15.0"; geodeticDatum: WGS84; **Event:** eventDate: 25/04/2001; **Record Level:** collectionCode: cWB

#### Harpalus (Harpalus) serripes serripes

(Quensel, 1806)

##### Materials

- **Type status:** Other material. **Occurrence:** recordedBy: R. Kostova; individualCount: 1; **Location:** countryCode: BG; locality: Rezovo Vill. surroundings; verbatimElevation: 5; verbatimCoordinates: N41°58'54.4", E28°01'29.6"; geodeticDatum: WGS84; **Event:** eventDate: 09/05/2009
- **Type status:** Other material. **Occurrence:** recordedBy: R. Kostova; individualCount: 1; **Location:** countryCode: BG; locality: Kosti Vill., "St. Ilia" Place; verbatimElevation: 35; verbatimCoordinates: N42°03'23.2", E27°45'51.6"; geodeticDatum: WGS84; **Event:** eventDate: 15.04-08.05.2009; habitat: meadow with single trees
- **Type status:** Other material. **Occurrence:** recordedBy: R. Kostova; individualCount: 1; **Location:** countryCode: BG; locality: Kosti Vill., "St. Ilia" Place; verbatimElevation: 35; verbatimCoordinates: N42°03'23.2", E27°45'51.6"; geodeticDatum: WGS84; **Event:** eventDate: 09.06-02.07.2009; habitat: meadow with single trees
- **Type status:** Other material. **Location:** countryCode: BG; locality: Primorsko; **Record Level:** bibliographicCitation: Hieke & Wrase (1988: 135)
- **Type status:** Other material. **Location:** countryCode: BG; locality: Ropotamo; **Record Level:** bibliographicCitation: Hieke & Wrase (1988: 135)

#### Harpalus (Harpalus) smaragdinus

(Duftschmid, 1812)

##### Materials

- **Type status:** Other material. **Occurrence:** recordedBy: R. Kostova; individualCount: 1; **Location:** countryCode: BG; locality: Kondolovo Vill., "Byalata prast" Place; verbatimElevation: 271; verbatimCoordinates: N42°05'47.7", E27°39'55.0"; geodeticDatum: WGS84; **Event:** eventDate: 09.06-02.07.2009; habitat: oak-beech mixed forest
- **Type status:** Other material. **Location:** countryCode: BG; locality: Ahtopol; **Record Level:** bibliographicCitation: Hieke & Wrase (1988: 132)

#### Harpalus (Harpalus) sulphuripes sulphuripes

Germar, 1824

##### Materials

- **Type status:** Other material. **Occurrence:** recordedBy: R. Kostova; individualCount: 1; **Location:** countryCode: BG; locality: Malko Tarnovo surroundings; verbatimElevation: 375; verbatimCoordinates: N41°59'01.7", E27°29'49.4"; geodeticDatum: WGS84; **Event:** eventDate: 27/05/2010
- **Type status:** Other material. **Occurrence:** recordedBy: R. Kostova; individualCount: 1; **Location:** countryCode: TR; locality: Vize surroundings; verbatimElevation: 366; verbatimCoordinates: N41°35'06.1", E27°47'41.5"; geodeticDatum: WGS84; **Event:** eventDate: 25/05/2011
- **Type status:** Other material. **Occurrence:** recordedBy: R. Kostova; individualCount: 1; **Location:** countryCode: BG; locality: Malko Tarnovo, "Propada" Place; verbatimElevation: 401; verbatimCoordinates: N41°58'49.6", E27°29'25.7"; geodeticDatum: WGS84; **Event:** eventDate: 09.05-08.06.2009; habitat: oriental beech forest
- **Type status:** Other material. **Occurrence:** recordedBy: R. Kostova; individualCount: 2; **Location:** countryCode: BG; locality: Slivarovo Vll. surroundings; verbatimElevation: 331; verbatimCoordinates: N41°58'11.9", E27°39'31.1"; geodeticDatum: WGS84; **Event:** eventDate: 09.05-08.06.2009; habitat: shrubs, mainly Calluna vulgaris
- **Type status:** Other material. **Occurrence:** recordedBy: R. Kostova; individualCount: 1; **Location:** countryCode: BG; locality: Slivarovo Vll. surroundings; verbatimElevation: 331; verbatimCoordinates: N41°58'11.9", E27°39'31.1"; geodeticDatum: WGS84; **Event:** eventDate: 03.07-02.08.2009; habitat: shrubs, mainly Calluna vulgaris
- **Type status:** Other material. **Occurrence:** recordedBy: R. Kostova; individualCount: 1; **Location:** countryCode: BG; locality: Brodilovo Vill., under Papiya peak; verbatimElevation: 296; verbatimCoordinates: N42°06'21.7", E27°50'32.6"; geodeticDatum: WGS84; **Event:** eventDate: 09.05-08.06.2009; habitat: shrubs, Phyllyrea latifolia
- **Type status:** Other material. **Occurrence:** recordedBy: R. Kostova; individualCount: 1; **Location:** countryCode: BG; locality: Sinemorets Vill., PA"Silistar", "Butamyata" Place; verbatimElevation: 17; verbatimCoordinates: N42°03'10.3", E27°59'13.4"; geodeticDatum: WGS84; **Event:** eventDate: 09.05-08.06.2009; habitat: oak forest
- **Type status:** Other material. **Occurrence:** recordedBy: R. Kostova; individualCount: 1; **Location:** countryCode: BG; locality: Sinemorets Vill., PA"Silistar", "Butamyata" Place; verbatimElevation: 10; verbatimCoordinates: N42°03'11.2", E27°59'19.7"; geodeticDatum: WGS84; **Event:** eventDate: 15.04-08.05.2009; habitat: meadow, single shrubs
- **Type status:** Other material. **Occurrence:** recordedBy: R. Kostova; individualCount: 1; **Location:** countryCode: BG; locality: Stoilovo Vill., "Petrova niva" Place; verbatimElevation: 234; verbatimCoordinates: N42°03'40.3", E27°31'42.3"; geodeticDatum: WGS84; **Event:** eventDate: 09.05-08.06.2009; habitat: meadow, single shrubs
- **Type status:** Other material. **Occurrence:** recordedBy: J. Mařan & K. Taborsky; individualCount: 5; **Location:** countryCode: BG; locality: Maslen nos Cape; **Event:** eventDate: VI.1933; **Record Level:** institutionCode: NMP
- **Type status:** Other material. **Location:** countryCode: BG; locality: Ropotamo; **Record Level:** bibliographicCitation: Hieke & Wrase (1988: 139)

#### Harpalus (Harpalus) tardus

(Panzer, 1796)

##### Materials

- **Type status:** Other material. **Occurrence:** recordedBy: R. Kostova; individualCount: 1; **Location:** countryCode: TR; locality: Demirköy, to Mahya Dağı peak; verbatimElevation: 488; verbatimCoordinates: N41°52'41.1", E27°54'27.0"; geodeticDatum: WGS84; **Event:** eventDate: 05/07/2009
- **Type status:** Other material. **Occurrence:** recordedBy: R. Kostova; individualCount: 1; **Location:** countryCode: TR; locality: Sergen Vill. surroundings; verbatimElevation: 818; verbatimCoordinates: N41°44'37.0", E27°42'24.8"; geodeticDatum: WGS84; **Event:** eventDate: 25/05/2011
- **Type status:** Other material. **Occurrence:** recordedBy: R. Kostova; individualCount: 1; **Location:** countryCode: BG; locality: Sinemorets Vill., PA "Estuary of Veleka river"; verbatimElevation: 6; verbatimCoordinates: N42°03'39.6", E27°57'55.2"; geodeticDatum: WGS84; **Event:** eventDate: 16/04/2009
- **Type status:** Other material. **Occurrence:** recordedBy: R. Kostova; individualCount: 1; **Location:** countryCode: TR; locality: Demirköy surroundings; verbatimElevation: 655; verbatimCoordinates: N41°47'43.1", E27°44'13.3"; geodeticDatum: WGS84; **Event:** eventDate: 25/05/2010
- **Type status:** Other material. **Occurrence:** recordedBy: R. Kostova; individualCount: 1; **Location:** countryCode: TR; locality: Yalikŏy, river estuary; verbatimElevation: 9; verbatimCoordinates: N41°29'27.7", E28°16'40.0"; geodeticDatum: WGS84; **Event:** eventDate: 23/05/2011
- **Type status:** Other material. **Occurrence:** recordedBy: R. Kostova; individualCount: 1; **Location:** countryCode: BG; locality: Bliznak Vill., PA"Bataka"; verbatimElevation: 324; verbatimCoordinates: N42°11'37.3", E27°19'35.8"; geodeticDatum: WGS84; **Event:** eventDate: 09.05-08.06.2009; habitat: oak forest
- **Type status:** Other material. **Occurrence:** recordedBy: R. Kostova; individualCount: 2; **Location:** countryCode: BG; locality: Malko Tarnovo, "Propada" Place; verbatimElevation: 401; verbatimCoordinates: N41°58'49.6", E27°29'25.7"; geodeticDatum: WGS84; **Event:** eventDate: 09.05-08.06.2009; habitat: oriental beech forest
- **Type status:** Other material. **Occurrence:** recordedBy: R. Kostova; individualCount: 19; **Location:** countryCode: BG; locality: Slivarovo Vill., "Shafariitsa" Place; verbatimElevation: 224; verbatimCoordinates: N41°57'37.8", E27°39'34.8"; geodeticDatum: WGS84; **Event:** eventDate: 15.04-02.08.2009; habitat: black alder-oak forest
- **Type status:** Other material. **Occurrence:** recordedBy: R. Kostova; individualCount: 71; **Location:** countryCode: BG; locality: Kondolovo Vill., "Byalata prast" Place; verbatimElevation: 271; verbatimCoordinates: N42°05'47.7", E27°39'55.0"; geodeticDatum: WGS84; **Event:** eventDate: 15.04-02.08.2009; habitat: oak-beech mixed forest
- **Type status:** Other material. **Occurrence:** recordedBy: R. Kostova; individualCount: 9; **Location:** countryCode: BG; locality: Kosti Vill., "St. Ilia" Place; verbatimElevation: 35; verbatimCoordinates: N42°03'23.2", E27°45'51.6"; geodeticDatum: WGS84; **Event:** eventDate: 15.04-08.06.2009; habitat: meadow with single trees
- **Type status:** Other material. **Occurrence:** recordedBy: R. Kostova; individualCount: 2; **Location:** countryCode: BG; locality: Bulgari Vill., PA "Marina reka"; verbatimElevation: 183; verbatimCoordinates: N42°06'41.7", E27°45'53.0"; geodeticDatum: WGS84; **Event:** eventDate: 09.05-02.07.2009; habitat: oriental beech-oak forest with Rododendron ponticum
- **Type status:** Other material. **Occurrence:** recordedBy: R. Kostova; individualCount: 12; **Location:** countryCode: BG; locality: Brodilovo Vill., under Papiya peak; verbatimElevation: 336; verbatimCoordinates: N42°06'23.7", E27°50'36.1"; geodeticDatum: WGS84; **Event:** eventDate: 15.04-08.06.2009; habitat: oak forest
- **Type status:** Other material. **Occurrence:** recordedBy: R. Kostova; individualCount: 2; **Location:** countryCode: BG; locality: Brodilovo Vill., under Papiya peak; verbatimElevation: 296; verbatimCoordinates: N42°06'21.7", E27°50'32.6"; geodeticDatum: WGS84; **Event:** eventDate: 09.05-08.06.2009; habitat: shrubs, Phyllyrea latifolia
- **Type status:** Other material. **Occurrence:** recordedBy: R. Kostova; individualCount: 5; **Location:** countryCode: BG; locality: Sinemorets Vill., PA"Silistar", "Butamyata" Place; verbatimElevation: 17; verbatimCoordinates: N42°03'10.3", E27°59'13.4"; geodeticDatum: WGS84; **Event:** eventDate: 09.05-08.06.2009; habitat: oak forest
- **Type status:** Other material. **Occurrence:** recordedBy: R. Kostova; individualCount: 1; **Location:** countryCode: BG; locality: Mladezhko Vill., The springs of Mladezhka River; verbatimElevation: 231; verbatimCoordinates: N42°09'04.5", E27°21'26.1"; geodeticDatum: WGS84; **Event:** eventDate: 09.05-08.06.2009; habitat: black alder and hornbeam forest
- **Type status:** Other material. **Occurrence:** recordedBy: J. Mařan & K. Taborsky; individualCount: 2; **Location:** countryCode: BG; locality: Maslen nos Cape; **Event:** eventDate: VI.1933; **Record Level:** institutionCode: NMP
- **Type status:** Other material. **Location:** countryCode: BG; locality: Ropotamo; **Record Level:** bibliographicCitation: Hieke & Wrase (1988: 135)

#### Harpalus (Pseudoophonus) calceatus

(Duftschmid, 1812)

##### Materials

- **Type status:** Other material. **Occurrence:** recordedBy: R. Kostova; individualCount: 1; **Location:** countryCode: BG; locality: Bliznak Vill., PA"Bataka"; verbatimElevation: 324; verbatimCoordinates: N42°11'37.3", E27°19'35.8"; geodeticDatum: WGS84; **Event:** eventDate: 09.06-02.07.2009; habitat: oak forest
- **Type status:** Other material. **Occurrence:** recordedBy: R. Kostova; individualCount: 1; **Location:** countryCode: BG; locality: Bliznak Vill., PA"Bataka"; verbatimElevation: 324; verbatimCoordinates: N42°11'37.3", E27°19'35.8"; geodeticDatum: WGS84; **Event:** eventDate: 03.08-07.09.2009; habitat: oak forest
- **Type status:** Other material. **Occurrence:** recordedBy: R. Radev; individualCount: 1; **Location:** countryCode: BG; locality: Sinemorets Vill.; **Event:** eventDate: 07/08/1989; **Record Level:** collectionID: R. Radev coll.
- **Type status:** Other material. **Location:** countryCode: BG; locality: Ahtopol; **Record Level:** bibliographicCitation: Hieke & Wrase (1988: 128)
- **Type status:** Other material. **Location:** countryCode: BG; locality: Primorsko; **Record Level:** bibliographicCitation: Hieke & Wrase (1988: 128)
- **Type status:** Other material. **Occurrence:** recordedBy: B. Guéorguiev; individualCount: 1; **Location:** countryCode: BG; locality: Sinemorets Village, south bay; **Event:** eventDate: 27/05/1995; **Record Level:** institutionCode: NMNHS

#### Harpalus (Pseudoophonus) griseus

(Panzer, 1796)

##### Materials

- **Type status:** Other material. **Occurrence:** recordedBy: W. Beier; individualCount: 1; **Location:** countryCode: BG; locality: Zvezdets Vill.; verbatimElevation: 300; verbatimCoordinates: N42°05'49.0", E27°25'47.0"; geodeticDatum: WGS84; **Record Level:** collectionCode: cWB
- **Type status:** Other material. **Occurrence:** recordedBy: R. Kostova; individualCount: 1; **Location:** countryCode: BG; locality: Sinemorets Vill., PA"Silistar", "Butamyata" Place; verbatimElevation: 10; verbatimCoordinates: N42°03'11.2", E27°59'19.7"; geodeticDatum: WGS84; **Event:** eventDate: 03.07-02.08.2009; habitat: meadow, single shrubs
- **Type status:** Other material. **Location:** countryCode: BG; locality: Ahtopol; **Record Level:** bibliographicCitation: Hieke & Wrase (1988: 127)
- **Type status:** Other material. **Location:** countryCode: BG; locality: Varvara Vill.; **Record Level:** bibliographicCitation: Hieke & Wrase (1988: 127)
- **Type status:** Other material. **Location:** countryCode: BG; locality: Strandzha; **Record Level:** bibliographicCitation: Guéorguiev & Guéorguiev (1995: 187, as P. griseus)
- **Type status:** Other material. **Occurrence:** recordedBy: R. Radev; individualCount: 1; **Location:** countryCode: BG; locality: Sinemorets Vill.; **Record Level:** collectionID: R. Radev coll.

#### Harpalus (Pseudoophonus) rufipes

(De Geer, 1774)

##### Materials

- **Type status:** Other material. **Occurrence:** recordedBy: R. Bekchiev; individualCount: 1; **Location:** countryCode: BG; locality: Primorsko, Perla Beach; verbatimElevation: 1; verbatimCoordinates: N42°17'10.2", E27°45'13.6"; geodeticDatum: WGS84; **Event:** eventDate: 30/06/2009
- **Type status:** Other material. **Occurrence:** recordedBy: R. Bekchiev; individualCount: 1; **Location:** countryCode: BG; locality: Primorsko, "Arcutino" Place; verbatimElevation: 60; verbatimCoordinates: N42°20'16.0", E27°43'18.1"; geodeticDatum: WGS84; **Event:** eventDate: 30/06/2009
- **Type status:** Other material. **Occurrence:** recordedBy: R. Bekchiev; individualCount: 1; **Location:** countryCode: TR; locality: Along Demirköy - Igneada road; verbatimElevation: 12; verbatimCoordinates: N41°52'39.3", E27°56'29.2"; geodeticDatum: WGS84; **Event:** eventDate: 05/07/2009
- **Type status:** Other material. **Occurrence:** recordedBy: R. Bekchiev; individualCount: 1; **Location:** countryCode: TR; locality: Sarpdere Vill., Dupnisa Cave surroundings; verbatimElevation: 440; verbatimCoordinates: N41°49'56.3", E27°33'24.0"; geodeticDatum: WGS84; **Event:** eventDate: 08/07/2009
- **Type status:** Other material. **Occurrence:** recordedBy: R. Kostova; individualCount: 6; **Location:** countryCode: BG; locality: Kosti Vill., "St. Ilia" Place; verbatimElevation: 35; verbatimCoordinates: N42°03'23.2", E27°45'51.6"; geodeticDatum: WGS84; **Event:** eventDate: 09.05-02.07.2009; habitat: meadow with single trees
- **Type status:** Other material. **Occurrence:** recordedBy: R. Kostova; individualCount: 1; **Location:** countryCode: BG; locality: Bulgari Vill., PA "Marina reka"; verbatimElevation: 183; verbatimCoordinates: N42°06'41.7", E27°45'53.0"; geodeticDatum: WGS84; **Event:** eventDate: 03.07-02.08.2009; habitat: oriental beech-oak forest with Rododendron ponticum
- **Type status:** Other material. **Occurrence:** recordedBy: R. Kostova; individualCount: 1; **Location:** countryCode: BG; locality: Sinemorets Vill., PA "Estuary of Veleka river"; verbatimElevation: 6; verbatimCoordinates: N42°03'39.6", E27°57'55.2"; geodeticDatum: WGS84; **Event:** eventDate: 09.06-02.07.2009; habitat: swamp forest
- **Type status:** Other material. **Occurrence:** recordedBy: R. Kostova; individualCount: 1; **Location:** countryCode: BG; locality: Sinemorets Vill., PA"Silistar", "Butamyata" Place; verbatimElevation: 17; verbatimCoordinates: N42°03'10.3", E27°59'13.4"; geodeticDatum: WGS84; **Event:** eventDate: 03.07-02.08.2009; habitat: oak forest
- **Type status:** Other material. **Occurrence:** recordedBy: R. Kostova; individualCount: 1; **Location:** countryCode: BG; locality: Sinemorets Vill., PA"Silistar", "Butamyata" Place; verbatimElevation: 10; verbatimCoordinates: N42°03'11.2", E27°59'19.7"; geodeticDatum: WGS84; **Event:** eventDate: 03.07-02.08.2009; habitat: meadow, single shrubs
- **Type status:** Other material. **Occurrence:** recordedBy: R. Kostova; individualCount: 1; **Location:** countryCode: BG; locality: Sinemorets Vill., PA"Silistar"; verbatimElevation: 23; verbatimCoordinates: N42°01'30.7", E28°00'25.4"; geodeticDatum: WGS84; **Event:** eventDate: 03.08-07.09.2009; habitat: oak forest
- **Type status:** Other material. **Occurrence:** recordedBy: R. Kostova; individualCount: 1; **Location:** countryCode: TR; locality: Igneada surroundings; verbatimElevation: 15; verbatimCoordinates: N41°51'51.0", E27°56'54.1"; geodeticDatum: WGS84; **Event:** eventDate: 06.07-29.09.2009; habitat: swamp forest
- **Type status:** Other material. **Location:** countryCode: BG; locality: Ahtopol; **Record Level:** bibliographicCitation: Hieke & Wrase (1988: 127)
- **Type status:** Other material. **Location:** countryCode: BG; locality: Tsarevo (= Micurin); **Record Level:** bibliographicCitation: Hieke & Wrase (1988: 127)
- **Type status:** Other material. **Location:** countryCode: BG; locality: Ropotamo; **Record Level:** bibliographicCitation: Hieke & Wrase (1988: 127)
- **Type status:** Other material. **Location:** countryCode: BG; locality: Strandzha; **Record Level:** bibliographicCitation: Guéorguiev & Guéorguiev (1995: 187, as P. rufipes)
- **Type status:** Other material. **Occurrence:** recordedBy: R. Radev; individualCount: 1; **Location:** countryCode: BG; locality: Sinemorets Vill.; **Event:** eventDate: 07/08/1989; **Record Level:** collectionID: R. Radev coll.
- **Type status:** Other material. **Occurrence:** recordedBy: P. Petcoff; individualCount: 1; **Location:** countryCode: BG; locality: Malko Tarnovo; **Event:** eventDate: 3-5.05.1921; **Record Level:** institutionCode: NMNHS
- **Type status:** Other material. **Occurrence:** recordedBy: V. Lazarov; individualCount: 1; **Location:** countryCode: BG; locality: Primorsko; **Event:** eventDate: VI.1957; **Record Level:** institutionCode: NMNHS
- **Type status:** Other material. **Occurrence:** recordedBy: B. Guéorguiev; individualCount: 1; **Location:** countryCode: BG; locality: Veleka River near Mladezko Village; **Event:** eventDate: 25/05/1995; **Record Level:** institutionCode: NMNHS
- **Type status:** Other material. **Occurrence:** recordedBy: B. Guéorguiev; individualCount: 2; **Location:** countryCode: BG; locality: Aydere River; verbatimElevation: 350-450; **Event:** eventDate: 26/05/1995; **Record Level:** institutionCode: NMNHS
- **Type status:** Other material. **Occurrence:** recordedBy: B. Guéorguiev; individualCount: 2; **Location:** countryCode: BG; locality: Sinemorets Village, south bay; **Event:** eventDate: 27/05/1995; **Record Level:** institutionCode: NMNHS

#### Ophonus (Hesperophonus) azureus

(Fabricius, 1775)

##### Materials

- **Type status:** Other material. **Location:** countryCode: BG; locality: Ahtopol; **Record Level:** bibliographicCitation: Hieke & Wrase (1988: 125)
- **Type status:** Other material. **Location:** countryCode: BG; locality: Tsarevo (= Micurin); **Record Level:** bibliographicCitation: Hieke & Wrase (1988: 125)
- **Type status:** Other material. **Location:** countryCode: BG; locality: Malko Tarnovo; **Record Level:** bibliographicCitation: Guéorguiev & Guéorguiev (1995: 182)
- **Type status:** Other material. **Location:** countryCode: BG; locality: Primorsko (= Kyupriya); **Record Level:** bibliographicCitation: Guéorguiev & Guéorguiev (1995: 182)
- **Type status:** Other material. **Occurrence:** recordedBy: D. Iltchev; individualCount: 1; **Location:** countryCode: BG; locality: Vizitsa Vill.; **Event:** eventDate: 1-7.06.1923; **Record Level:** institutionCode: NMNHS

#### Ophonus (Hesperophonus) cribricollis

(Dejean, 1829)

##### Materials

- **Type status:** Other material. **Occurrence:** recordedBy: R. Kostova; individualCount: 1; **Location:** countryCode: BG; locality: Slivarovo Vll. surroundings; verbatimElevation: 331; verbatimCoordinates: N41°58'11.9", E27°39'31.1"; geodeticDatum: WGS84; **Event:** eventDate: 09.06-02.07.2009; habitat: shrubs, mainly Calluna vulgaris
- **Type status:** Other material. **Occurrence:** recordedBy: J. Mařan & K. Taborsky; individualCount: 5; **Location:** countryCode: BG; locality: Maslen nos Cape; **Event:** eventDate: VI.1933; **Record Level:** institutionCode: NMP
- **Type status:** Other material. **Occurrence:** recordedBy: W. Beier; individualCount: 3; **Location:** countryCode: BG; locality: Stoilovo Vill.; verbatimElevation: 340; verbatimCoordinates: N42°02'23.0", E27°31'15.0"; geodeticDatum: WGS84; **Record Level:** collectionCode: cWB
- **Type status:** Other material. **Location:** countryCode: BG; locality: Golyama Papiya Peak; **Record Level:** bibliographicCitation: Guéorguiev & Guéorguiev (1995: 183)
- **Type status:** Other material. **Location:** countryCode: BG; locality: Kiten; **Record Level:** bibliographicCitation: Hieke & Wrase (1988: 126)
- **Type status:** Other material. **Location:** countryCode: BG; locality: Tsarevo (= Micurin); **Record Level:** bibliographicCitation: Hieke & Wrase (1988: 126)

#### Ophonus (Hesperophonus) jailensis

(Schauberger, 1926)

##### Materials

- **Type status:** Other material. **Location:** countryCode: BG; locality: Tsarevo (= Micurin); **Record Level:** bibliographicCitation: Wrase (1991: 14)
- **Type status:** Other material. **Location:** countryCode: BG; locality: Varvara Vill.; **Record Level:** bibliographicCitation: Wrase (1991: 14)
- **Type status:** Other material. **Location:** countryCode: BG; locality: Road from "Kavatsite" Camping site to Varvara Vill.; **Record Level:** bibliographicCitation: Guéorguiev & Guéorguiev (1995: 182)

#### Ophonus (Hesperophonus) similis

(Dejean, 1829)

##### Materials

- **Type status:** Other material. **Occurrence:** recordedBy: R. Kostova; individualCount: 1; **Location:** countryCode: BG; locality: Slivarovo Vill., "Shafariitsa" Place; verbatimElevation: 224; verbatimCoordinates: N41°57'37.8", E27°39'34.8"; geodeticDatum: WGS84; **Event:** eventDate: 03.07-02.08.2009; habitat: black alder-oak forest

#### Ophonus (Hesperophonus) subquadratus

(Dejean, 1829)

##### Materials

- **Type status:** Other material. **Occurrence:** recordedBy: R. Kostova; individualCount: 1; **Location:** countryCode: BG; locality: Brodilovo Vill., under Papiya peak; verbatimElevation: 296; verbatimCoordinates: N42°06'21.7", E27°50'32.6"; geodeticDatum: WGS84; **Event:** eventDate: 03.07-02.08.2009; habitat: shrubs, Phyllyrea latifolia
- **Type status:** Other material. **Occurrence:** recordedBy: R. Kostova; individualCount: 1; **Location:** countryCode: BG; locality: Stoilovo Vill., "Petrova niva" Place; verbatimElevation: 234; verbatimCoordinates: N42°03'40.3", E27°31'42.3"; geodeticDatum: WGS84; **Event:** eventDate: 09.06-02.07.2009; habitat: meadow, single shrubs
- **Type status:** Other material. **Location:** countryCode: BG; locality: Ahtopol; **Record Level:** bibliographicCitation: Hieke & Wrase (1988: 126)
- **Type status:** Other material. **Location:** countryCode: BG; locality: Kiten; **Record Level:** bibliographicCitation: Hieke & Wrase (1988: 126)
- **Type status:** Other material. **Location:** countryCode: BG; locality: Tsarevo (= Micurin); **Record Level:** bibliographicCitation: Hieke & Wrase (1988: 126)
- **Type status:** Other material. **Location:** countryCode: BG; locality: Ropotamo; **Record Level:** bibliographicCitation: Hieke & Wrase (1988: 126)

#### Ophonus (Metophonus) brevicollis

(Audinet-Serville, 1821)

##### Materials

- **Type status:** Other material. **Location:** countryCode: BG; locality: Ahtopol; **Record Level:** bibliographicCitation: Hieke & Wrase (1988: 124)
- **Type status:** Other material. **Location:** countryCode: BG; locality: Tsarevo (= Micurin); **Record Level:** bibliographicCitation: Hieke & Wrase (1988: 124)

#### Ophonus (Metophonus) gabrieleae

Wrase, 1987

##### Materials

- **Type status:** Other material. **Location:** countryCode: BG; locality: Tsarevo (= Micurin); **Record Level:** bibliographicCitation: Hieke & Wrase (1988: 125)
- **Type status:** Other material. **Location:** countryCode: BG; locality: Tsarevo (= Micurin); **Record Level:** bibliographicCitation: Wrase (1987: 63)

#### Ophonus (Metophonus) laticollis

Mannerheim, 1825

##### Materials

- **Type status:** Other material. **Occurrence:** recordedBy: P. Mitov; individualCount: 1; **Location:** countryCode: BG; locality: Rezovo Vill. surroundings; verbatimElevation: 5; verbatimCoordinates: N41°59'06.1", E28°02'04.2"; geodeticDatum: WGS84; **Event:** eventDate: VI-IX.2009; habitat: meadows
- **Type status:** Other material. **Occurrence:** recordedBy: R. Kostova; individualCount: 1; **Location:** countryCode: BG; locality: Varovnik Vill., near the bridge at Fakiiska River; verbatimElevation: 125; verbatimCoordinates: N42°13'33.7", E27°11'33.7"; geodeticDatum: WGS84; **Event:** eventDate: 09/06/2009
- **Type status:** Other material. **Occurrence:** recordedBy: R. Kostova; individualCount: 1; **Location:** countryCode: BG; locality: Malko Tarnovo, "Propada" Place; verbatimElevation: 401; verbatimCoordinates: N41°58'49.6", E27°29'25.7"; geodeticDatum: WGS84; **Event:** eventDate: 09.05-08.06.2009; habitat: oriental beech forest
- **Type status:** Other material. **Occurrence:** recordedBy: R. Kostova; individualCount: 2; **Location:** countryCode: BG; locality: Slivarovo Vill., "Shafariitsa" Place; verbatimElevation: 224; verbatimCoordinates: N41°57'37.8", E27°39'34.8"; geodeticDatum: WGS84; **Event:** eventDate: 09.06-02.07.2009; habitat: black alder-oak forest
- **Type status:** Other material. **Occurrence:** recordedBy: R. Kostova; individualCount: 3; **Location:** countryCode: BG; locality: Stoilovo Vill., "Petrova niva" Place; verbatimElevation: 234; verbatimCoordinates: N42°03'40.3", E27°31'42.3"; geodeticDatum: WGS84; **Event:** eventDate: 09.06-02.07.2009; habitat: meadow, single shrubs

#### Ophonus (Metophonus) melletii melletii

(Heer, 1837)

##### Materials

- **Type status:** Other material. **Occurrence:** recordedBy: R. Bekchiev; individualCount: 1; **Location:** countryCode: BG; locality: Sinemorets Vill., PA"Silistar"; verbatimElevation: 23; verbatimCoordinates: N42°01'30.7", E28°00'25.4"; geodeticDatum: WGS84; **Event:** eventDate: 13/06/2009
- **Type status:** Other material. **Occurrence:** recordedBy: R. Bekchiev; individualCount: 1; **Location:** countryCode: TR; locality: Sarpdere Vill., Dupnisa Cave surroundings; verbatimElevation: 440; verbatimCoordinates: N41°49'56.3", E27°33'24.0"; geodeticDatum: WGS84; **Event:** eventDate: 08/07/2009
- **Type status:** Other material. **Occurrence:** recordedBy: R. Kostova; individualCount: 1; **Location:** countryCode: BG; locality: Kosti Vill., "St. Ilia" Place; verbatimElevation: 35; verbatimCoordinates: N42°03'23.2", E27°45'51.6"; geodeticDatum: WGS84; **Event:** eventDate: 03.07-02.08.2009; habitat: meadow with single trees
- **Type status:** Other material. **Occurrence:** recordedBy: R. Kostova; individualCount: 6; **Location:** countryCode: BG; locality: Kosti Vill., "St. Ilia" Place; verbatimElevation: 35; verbatimCoordinates: N42°03'23.2", E27°45'51.6"; geodeticDatum: WGS84; **Event:** eventDate: 03.08-07.09.2009; habitat: meadow with single trees
- **Type status:** Other material. **Location:** countryCode: BG; locality: Ahtopol; **Record Level:** bibliographicCitation: Hieke & Wrase (1988: 123)
- **Type status:** Other material. **Location:** countryCode: BG; locality: Ropotamo; **Record Level:** bibliographicCitation: Hieke & Wrase (1988: 123)
- **Type status:** Other material. **Location:** countryCode: BG; locality: Varvara Vill.; **Record Level:** bibliographicCitation: Hieke & Wrase (1988: 123)

#### Ophonus (Metophonus) puncticeps

Stephens, 1828

##### Materials

- **Type status:** Other material. **Location:** countryCode: BG; locality: Ahtopol; **Record Level:** bibliographicCitation: Hieke & Wrase (1988: 123)
- **Type status:** Other material. **Location:** countryCode: BG; locality: Tsarevo (= Micurin); **Record Level:** bibliographicCitation: Hieke & Wrase (1988: 123)
- **Type status:** Other material. **Occurrence:** recordedBy: P. Beron; individualCount: 1; **Location:** countryCode: BG; locality: Kiten; **Event:** eventDate: 16-22.12.1984; **Record Level:** institutionCode: NMNHS

#### Ophonus (Metophonus) rufibarbis

(Fabricius, 1792)

##### Materials

- **Type status:** Other material. **Occurrence:** recordedBy: W. Beier; individualCount: 1; **Location:** countryCode: BG; locality: Mouth of Veleka River near Sinemorets Vill.; verbatimElevation: 20; verbatimCoordinates: N42°03'33.0", E27°58'04.0"; geodeticDatum: WGS84; **Record Level:** collectionCode: cWB
- **Type status:** Other material. **Occurrence:** recordedBy: R. Bekchiev; individualCount: 3; **Location:** countryCode: TR; locality: Sarpdere Vill., Dupnisa Cave surroundings; verbatimElevation: 440; verbatimCoordinates: N41°49'56.3", E27°33'24.0"; geodeticDatum: WGS84; **Event:** eventDate: 08/07/2009
- **Type status:** Other material. **Location:** countryCode: BG; locality: Ahtopol; **Record Level:** bibliographicCitation: Hieke & Wrase (1988: 124)

#### Ophonus (Metophonus) rupicola

(Sturm, 1818)

##### Materials

- **Type status:** Other material. **Location:** countryCode: BG; locality: Ahtopol; **Record Level:** bibliographicCitation: Hieke & Wrase (1988: 124)
- **Type status:** Other material. **Location:** countryCode: BG; locality: Tsarevo (= Micurin); **Record Level:** bibliographicCitation: Hieke & Wrase (1988: 124)
- **Type status:** Other material. **Occurrence:** recordedBy: B. Guéorguiev; individualCount: 1; **Location:** countryCode: BG; locality: route Primorsko Town - Pismenovo Vill.; verbatimElevation: 50-90; **Event:** eventDate: 26/08/2004; fieldNumber: un; **Record Level:** institutionCode: NMNHS

#### Ophonus (Metophonus) subsinuatus

Rey, 1886

##### Materials

- **Type status:** Other material. **Location:** countryCode: BG; locality: Ahtopol; **Record Level:** bibliographicCitation: Guéorguiev (2011: 527)

#### Ophonus (Ophonus) ardosiacus

(Lutshnik, 1922)

##### Materials

- **Type status:** Other material. **Location:** countryCode: BG; locality: Ropotamo; **Record Level:** bibliographicCitation: Hieke & Wrase (1988: 121)
- **Type status:** Other material. **Occurrence:** recordedBy: B. Guéorguiev; individualCount: 2; **Location:** countryCode: BG; locality: Tisovitsa Reserve near Bulgari Vill.; **Event:** eventDate: 27/05/1995; **Record Level:** institutionCode: NMNHS
- **Type status:** Other material. **Occurrence:** recordedBy: R. Kostova; individualCount: 1; **Location:** countryCode: BG; locality: Stoilovo Vill., "Petrova niva" Place; verbatimElevation: 132; verbatimCoordinates: N42°03'41.9", E27°31'58.9"; geodeticDatum: WGS84; **Event:** eventDate: 06/05/2009; habitat: meadow, single shrubs
- **Type status:** Other material. **Occurrence:** recordedBy: R. Kostova; individualCount: 6; **Location:** countryCode: BG; locality: Sinemorets Vill., PA"Silistar", "Butamyata" Place; verbatimElevation: 10; verbatimCoordinates: N42°03'11.2", E27°59'19.7"; geodeticDatum: WGS84; **Event:** eventDate: 09.06-07.09.2009; habitat: meadow, single shrubs
- **Type status:** Other material. **Occurrence:** recordedBy: R. Kostova; individualCount: 1; **Location:** countryCode: BG; locality: Stoilovo Vill., "Petrova niva" Place; verbatimElevation: 234; verbatimCoordinates: N42°03'40.3", E27°31'42.3"; geodeticDatum: WGS84; **Event:** eventDate: 09.06-02.07.2009; habitat: meadow, single shrubs

#### Ophonus (Ophonus) diffinis

(Dejean, 1829)

##### Materials

- **Type status:** Other material. **Location:** countryCode: BG; locality: Ahtopol; **Record Level:** bibliographicCitation: Hieke & Wrase (1988: 121)
- **Type status:** Other material. **Location:** countryCode: BG; locality: Tsarevo (= Micurin); **Record Level:** bibliographicCitation: Hieke & Wrase (1988: 121)

#### Ophonus (Ophonus) franziniorum

Sciaky, 1987

##### Materials

- **Type status:** Other material. **Location:** countryCode: BG; locality: Ahtopol; **Record Level:** bibliographicCitation: Wrase (1991: 14, as O. franzinii)
- **Type status:** Other material. **Location:** countryCode: BG; locality: Kiten; **Record Level:** bibliographicCitation: Wrase (1991: 14, as O. franzinii)
- **Type status:** Other material. **Location:** countryCode: BG; locality: Tsarevo (= Micurin); **Record Level:** bibliographicCitation: Wrase (1991: 14, as O. franzinii)
- **Type status:** Other material. **Location:** countryCode: BG; locality: Ropotamo; **Record Level:** bibliographicCitation: Wrase (1991: 14, as O. franzinii)
- **Type status:** Other material. **Location:** countryCode: BG; locality: Road from Sozopol to Ahtopol; **Record Level:** bibliographicCitation: Guéorguiev & Guéorguiev (1995: 181, as O. franzinii)
- **Type status:** Other material. **Occurrence:** recordedBy: W. Beier; individualCount: 1; **Location:** countryCode: BG; locality: Bulgari Vill.; **Event:** eventDate: 18/05/2003; **Record Level:** collectionCode: cWB

#### Ophonus (Ophonus) sabulicola

(Panzer, 1796)

##### Materials

- **Type status:** Other material. **Occurrence:** recordedBy: R. Bekchiev; individualCount: 2; **Location:** countryCode: TR; locality: Sarpdere Vill., Dupnisa Cave surroundings; verbatimElevation: 440; verbatimCoordinates: N41°49'56.3", E27°33'24.0"; geodeticDatum: WGS84; **Event:** eventDate: 08/07/2009
- **Type status:** Other material. **Occurrence:** recordedBy: R. Kostova; individualCount: 46; **Location:** countryCode: BG; locality: Kosti Vill., "St. Ilia" Place; verbatimElevation: 35; verbatimCoordinates: N42°03'23.2", E27°45'51.6"; geodeticDatum: WGS84; **Event:** eventDate: 03.07-07.09.2009; habitat: meadow with single trees
- **Type status:** Other material. **Location:** countryCode: BG; locality: Ahtopol; **Record Level:** bibliographicCitation: Hieke & Wrase (1988: 121)
- **Type status:** Other material. **Location:** countryCode: BG; locality: Tsarevo (= Micurin); **Record Level:** bibliographicCitation: Hieke & Wrase (1988: 121)
- **Type status:** Other material. **Location:** countryCode: BG; locality: Ropotamo; **Record Level:** bibliographicCitation: Hieke & Wrase (1988: 121)
- **Type status:** Other material. **Occurrence:** recordedBy: K. Tuleshkov; individualCount: 1; **Location:** countryCode: BG; locality: Ahtopol; **Event:** eventDate: 15/05/1930; **Record Level:** institutionCode: NMNHS
- **Type status:** Other material. **Location:** countryCode: BG; locality: Malko Tarnovo; **Record Level:** bibliographicCitation: Guéorguiev & Guéorguiev (1995: 181)
- **Type status:** Other material. **Occurrence:** recordedBy: B. Guéorguiev; individualCount: 1; **Location:** countryCode: BG; locality: Aydere River; verbatimElevation: 350-450; **Event:** eventDate: 26/05/1995; **Record Level:** institutionCode: NMNHS
- **Type status:** Other material. **Occurrence:** recordedBy: B. Guéorguiev; individualCount: 1; **Location:** countryCode: BG; locality: Sinemorets Village, south bay; **Event:** eventDate: 27/05/1995; **Record Level:** institutionCode: NMNHS

#### Pangus scaritides

(Sturm, 1818)

##### Materials

- **Type status:** Other material. **Location:** countryCode: BG; locality: Kiten; **Record Level:** bibliographicCitation: Hieke & Wrase (1988: 141)

#### Parophonus (Ophonomimus) hirsutulus

(Dejean, 1829)

##### Materials

- **Type status:** Other material. **Occurrence:** recordedBy: R. Kostova; individualCount: 1; **Location:** countryCode: BG; locality: Bulgari Vill., PA "Marina reka"; verbatimElevation: 172; verbatimCoordinates: N42°06'41.7", E27°45'53.0"; geodeticDatum: WGS84; **Event:** eventDate: 11/06/2009
- **Type status:** Other material. **Occurrence:** recordedBy: R. Kostova; individualCount: 2; **Location:** countryCode: BG; locality: Kosti Vill., "St. Ilia" Place; verbatimElevation: 35; verbatimCoordinates: N42°03'23.2", E27°45'51.6"; geodeticDatum: WGS84; **Event:** eventDate: 03.07-02.08.2009; habitat: meadow with single trees

#### Parophonus (Parophonus) laeviceps

(Ménétriés, 1832)

##### Materials

- **Type status:** Other material. **Occurrence:** recordedBy: W. Beier; individualCount: 1; **Location:** countryCode: BG; locality: Mouth of Ropotamo River, south riverside; **Event:** eventDate: 16/05/2003; **Record Level:** collectionCode: cWB

#### Parophonus (Parophonus) maculicornis

(Duftschmid, 1812)

##### Materials

- **Type status:** Other material. **Occurrence:** recordedBy: R. Kostova; individualCount: 1; **Location:** countryCode: TR; locality: Malko Tarnovo, Entrance to Vitanovo Reserve; verbatimElevation: 285; verbatimCoordinates: N42°01'22.9", E27°27'22.9"; geodeticDatum: WGS84; **Event:** eventDate: 27/05/2010
- **Type status:** Other material. **Occurrence:** recordedBy: R. Bekchiev; individualCount: 3; **Location:** countryCode: TR; locality: Yalikŏy, river estuary; verbatimElevation: 9; verbatimCoordinates: N41°29'27.7", E28°16'40.0"; geodeticDatum: WGS84; **Event:** eventDate: 23/05/2011
- **Type status:** Other material. **Occurrence:** recordedBy: R. Kostova; individualCount: 1; **Location:** countryCode: BG; locality: Slivarovo Vill., "Shafariitsa" Place; verbatimElevation: 224; verbatimCoordinates: N41°57'37.8", E27°39'34.8"; geodeticDatum: WGS84; **Event:** eventDate: 15.04-08.05.2009; habitat: black alder-oak forest
- **Type status:** Other material. **Occurrence:** recordedBy: R. Kostova; individualCount: 1; **Location:** countryCode: BG; locality: Slivarovo Vill., "Shafariitsa" Place; verbatimElevation: 224; verbatimCoordinates: N41°57'37.8", E27°39'34.8"; geodeticDatum: WGS84; **Event:** eventDate: 09.06-02.07.2009; habitat: black alder-oak forest
- **Type status:** Other material. **Location:** countryCode: BG; locality: Tsarevo (= Micurin); **Record Level:** bibliographicCitation: Hieke & Wrase (1988: 142)
- **Type status:** Other material. **Location:** countryCode: BG; locality: Primorsko; **Record Level:** bibliographicCitation: Hieke & Wrase (1988: 142)

#### Parophonus (Parophonus) mendax

(P. Rossi, 1790)

##### Materials

- **Type status:** Other material. **Occurrence:** recordedBy: A. Gijonova; **Location:** countryCode: BG; locality: Varvara Vill., "Garigi" Place, with ants; verbatimElevation: 60; verbatimCoordinates: N42°07'03.0", E27°53'35.0"; geodeticDatum: WGS84; **Event:** eventDate: 29/04/2011
- **Type status:** Other material. **Location:** countryCode: BG; locality: Mouth of Ropotamo River; **Record Level:** bibliographicCitation: Hieke & Wrase (1988: 143)
- **Type status:** Other material. **Occurrence:** recordedBy: V. Lazarov; individualCount: 1; **Location:** countryCode: BG; locality: Primorsko; **Event:** eventDate: VI.1957; **Record Level:** institutionCode: NMNHS
- **Type status:** Other material. **Occurrence:** recordedBy: B. Guéorguiev; individualCount: 1; **Location:** countryCode: BG; locality: Primorsko Town env.; **Event:** eventDate: 20/08/2004; habitat: oak forest; fieldNumber: un; **Record Level:** institutionCode: NMNHS

#### Parophonus (Parophonus) planicollis

(Dejean, 1829)

##### Materials

- **Type status:** Other material. **Occurrence:** recordedBy: V. Lazarov; individualCount: 1; **Location:** countryCode: BG; locality: Primorsko; **Event:** eventDate: VI.1957; **Record Level:** institutionCode: NMNHS

#### Acupalpus (Acupalpus) elegans

(Dejean, 1829)

##### Materials

- **Type status:** Other material. **Occurrence:** recordedBy: R. Bekchiev; individualCount: 2; **Location:** countryCode: TR; locality: Igneada surroundings; verbatimElevation: 3; verbatimCoordinates: N41°51'27.4", E27°57'32.2"; geodeticDatum: WGS84; **Event:** eventDate: 05/07/2009; habitat: marsh
- **Type status:** Other material. **Occurrence:** recordedBy: R. Bekchiev; individualCount: 2; **Location:** countryCode: TR; locality: Igneada; verbatimElevation: 3; verbatimCoordinates: N41°52'51.3", E27°59'25.5"; geodeticDatum: WGS84; **Event:** eventDate: 02/10/2009
- **Type status:** Other material. **Occurrence:** recordedBy: R. Bekchiev; individualCount: 1; **Location:** countryCode: TR; locality: Igneada; verbatimElevation: 3; verbatimCoordinates: N41°52'51.3", E27°59'25.5"; geodeticDatum: WGS84; **Event:** eventDate: 25/05/2010
- **Type status:** Other material. **Location:** countryCode: BG; locality: Tsarevo (= Micurin); **Record Level:** bibliographicCitation: Hieke & Wrase (1988: 116)

#### Acupalpus (Acupalpus) exiguus

Dejean, 1829

##### Materials

- **Type status:** Other material. **Occurrence:** recordedBy: R. Kostova; individualCount: 1; **Location:** countryCode: BG; locality: Sinemorets Vill., PA "Estuary of Veleka river"; verbatimElevation: 6; verbatimCoordinates: N42°03'39.6", E27°57'55.2"; geodeticDatum: WGS84; **Event:** eventDate: 09.06-02.07.2009
- **Type status:** Other material. **Occurrence:** recordedBy: R. Bekchiev; individualCount: 1; **Location:** countryCode: TR; locality: Igneada, Hamam Gölü; verbatimElevation: 11; verbatimCoordinates: N41°49'31.6", E27°57'33.8"; geodeticDatum: WGS84; **Event:** eventDate: 02/10/2009
- **Type status:** Other material. **Location:** countryCode: BG; locality: Tsarevo (= Micurin); **Record Level:** bibliographicCitation: Hieke & Wrase (1988: 118)
- **Type status:** Other material. **Location:** countryCode: BG; locality: Mouth of Ropotamo River; **Record Level:** bibliographicCitation: Hieke & Wrase (1988: 118)
- **Type status:** Other material. **Location:** countryCode: BG; locality: Strandzha; **Record Level:** bibliographicCitation: Guéorguiev & Guéorguiev (1995: 176)

#### Acupalpus (Acupalpus) flavicollis

(Sturm, 1825)

##### Materials

- **Type status:** Other material. **Occurrence:** recordedBy: R. Bekchiev; individualCount: 1; **Location:** countryCode: TR; locality: Demirköy, Dökümhanesi; verbatimElevation: 179; verbatimCoordinates: N41°49'09.6", E27°48'43.2"; geodeticDatum: WGS84; **Event:** eventDate: 30/05/2009

#### Acupalus (Acupalpus) luteatus

(Duftcshmid, 1812)

##### Materials

- **Type status:** Other material. **Occurrence:** recordedBy: R. Kostova; individualCount: 1; **Location:** countryCode: BG; locality: Rezovo Vill. surroundings; verbatimElevation: 5; verbatimCoordinates: N41°58'54.4", E28°01'29.6"; geodeticDatum: WGS84; **Event:** eventDate: 09/05/2009
- **Type status:** Other material. **Occurrence:** recordedBy: R. Kostova & R. Bekchiev; individualCount: 4; **Location:** countryCode: TR; locality: Igneada surroundings; verbatimElevation: 3; verbatimCoordinates: N41°51'27.4", E27°57'32.2"; geodeticDatum: WGS84; **Event:** eventDate: 05/07/2009; habitat: marsh
- **Type status:** Other material. **Occurrence:** recordedBy: R. Bekchiev; individualCount: 3; **Location:** countryCode: TR; locality: Igneada, Hamam Gölü; verbatimElevation: 11; verbatimCoordinates: N41°49'31.6", E27°57'33.8"; geodeticDatum: WGS84; **Event:** eventDate: 02/10/2009
- **Type status:** Other material. **Occurrence:** recordedBy: R. Bekchiev; individualCount: 1; **Location:** countryCode: TR; locality: Igneada; verbatimElevation: 3; verbatimCoordinates: N41°52'51.3", E27°59'25.5"; geodeticDatum: WGS84; **Event:** eventDate: 02/10/2009
- **Type status:** Other material. **Occurrence:** recordedBy: R. Kostova; individualCount: 3; **Location:** countryCode: BG; locality: Primorsko, Perla Beach; verbatimElevation: 1; verbatimCoordinates: N42°17'10.2", E27°45'13.6"; geodeticDatum: WGS84; **Event:** eventDate: 10/05/2009
- **Type status:** Other material. **Occurrence:** recordedBy: R. Bekchiev; individualCount: 55; **Location:** countryCode: BG; locality: Primorsko, Perla Beach; verbatimElevation: 1; verbatimCoordinates: N42°17'10.2", E27°45'13.6"; geodeticDatum: WGS84; **Event:** eventDate: 30/06/2009
- **Type status:** Other material. **Occurrence:** recordedBy: R. Bekchiev; individualCount: 2; **Location:** countryCode: BG; locality: Primorsko, "Arcutino" Place; verbatimElevation: 60; verbatimCoordinates: N42°20'16.0", E27°43'18.1"; geodeticDatum: WGS84; **Event:** eventDate: 30/06/2009
- **Type status:** Other material. **Occurrence:** recordedBy: R. Bekchiev; individualCount: 1; **Location:** countryCode: BG; locality: Fazanovo Vill., "Popovi skali" Place; verbatimElevation: 26; verbatimCoordinates: N42°09'45.9", E27°44'15.6"; geodeticDatum: WGS84; **Event:** eventDate: 01/07/2009
- **Type status:** Other material. **Occurrence:** recordedBy: R. Bekchiev; individualCount: 1; **Location:** countryCode: BG; locality: Sinemorets Vill., PA "Estuary of Veleka river"; verbatimElevation: 6; verbatimCoordinates: N42°03'39.6", E27°57'55.2"; geodeticDatum: WGS84; **Event:** eventDate: 02/07/2009
- **Type status:** Other material. **Occurrence:** recordedBy: R. Kostova; individualCount: 1; **Location:** countryCode: BG; locality: Sinemorets Vill., PA"Silistar", Estuary of Silistar River; verbatimElevation: 12; verbatimCoordinates: N42°01'26.8", E28°00'32.9"; geodeticDatum: WGS84; **Event:** eventDate: 27/05/2011
- **Type status:** Other material. **Location:** countryCode: BG; locality: Tsarevo (= Micurin); **Record Level:** bibliographicCitation: Hieke & Wrase (1988: 118)
- **Type status:** Other material. **Location:** countryCode: BG; locality: Mouth of Ropotamo River; **Record Level:** bibliographicCitation: Hieke & Wrase (1988: 118)

#### Acupalpus (Acupalpus) maculatus

(Schaum, 1860)

##### Materials

- **Type status:** Other material. **Occurrence:** recordedBy: I. Gijonov; individualCount: 1; **Location:** countryCode: BG; locality: Sinemorets Vill., PA "Estuary of Veleka river"; verbatimElevation: 6; verbatimCoordinates: N42°03'39.6", E27°57'55.2"; geodeticDatum: WGS84; **Event:** eventDate: 16/04/2009
- **Type status:** Other material. **Occurrence:** recordedBy: R. Bekchiev; individualCount: 4; **Location:** countryCode: BG; locality: Primorsko, Perla Beach; verbatimElevation: 1; verbatimCoordinates: N42°17'10.2", E27°45'13.6"; geodeticDatum: WGS84; **Event:** eventDate: 30/06/2009
- **Type status:** Other material. **Occurrence:** recordedBy: R. Bekchiev; individualCount: 12; **Location:** countryCode: BG; locality: Primorsko, "Arcutino" Place; verbatimElevation: 60; verbatimCoordinates: N42°20'16.0", E27°43'18.1"; geodeticDatum: WGS84; **Event:** eventDate: 30/06/2009
- **Type status:** Other material. **Occurrence:** recordedBy: R. Bekchiev; individualCount: 1; **Location:** countryCode: BG; locality: Fazanovo Vill., "Popovi skali" Place; verbatimElevation: 26; verbatimCoordinates: N42°09'45.9", E27°44'15.6"; geodeticDatum: WGS84; **Event:** eventDate: 01/07/2009
- **Type status:** Other material. **Occurrence:** recordedBy: R. Bekchiev; individualCount: 1; **Location:** countryCode: BG; locality: Sinemorets Vill., PA "Estuary of Veleka river"; verbatimElevation: 6; verbatimCoordinates: N42°03'39.6", E27°57'55.2"; geodeticDatum: WGS84; **Event:** eventDate: 02/07/2009
- **Type status:** Other material. **Occurrence:** recordedBy: R. Bekchiev; individualCount: 5; **Location:** countryCode: TR; locality: Igneada surroundings; verbatimElevation: 3; verbatimCoordinates: N41°51'27.4", E27°57'32.2"; geodeticDatum: WGS84; **Event:** eventDate: 05/07/2009; habitat: marsh
- **Type status:** Other material. **Occurrence:** recordedBy: R. Bekchiev; individualCount: 1; **Location:** countryCode: TR; locality: Kiyiköy surroundings; verbatimElevation: 38; verbatimCoordinates: N41°39'25.7", E28°05'11.2"; geodeticDatum: WGS84; **Event:** eventDate: 24/05/2010
- **Type status:** Other material. **Occurrence:** recordedBy: R. Bekchiev; individualCount: 1; **Location:** countryCode: TR; locality: Igneada; verbatimElevation: 3; verbatimCoordinates: N41°52'51.3", E27°59'25.5"; geodeticDatum: WGS84; **Event:** eventDate: 25/05/2010
- **Type status:** Other material. **Occurrence:** recordedBy: R. Kostova; individualCount: 1; **Location:** countryCode: BG; locality: Malko Tarnovo, Entrance to Vitanovo Reserve; verbatimElevation: 285; verbatimCoordinates: N42°01'22.9", E27°27'22.9"; geodeticDatum: WGS84; **Event:** eventDate: 27/05/2010
- **Type status:** Other material. **Location:** countryCode: BG; locality: Tsarevo (= Micurin); **Record Level:** bibliographicCitation: Hieke & Wrase (1988: 118)
- **Type status:** Other material. **Occurrence:** recordedBy: B. Guéorguiev; individualCount: 1; **Location:** countryCode: BG; locality: Ahtopol; **Event:** eventDate: 28/05/1995; **Record Level:** institutionCode: NMNHS
- **Type status:** Other material. **Occurrence:** recordedBy: B. Guéorguiev; individualCount: 1; **Location:** countryCode: BG; locality: mouth of Veleka River near Sinemorets Vill.; **Event:** eventDate: 28/05/1995; **Record Level:** institutionCode: NMNHS

#### Acupalpus (Acupalpus) meridianus

(Linnaeus, 1761)

##### Materials

- **Type status:** Other material. **Location:** countryCode: BG; locality: Primorsko; **Record Level:** bibliographicCitation: Hieke & Wrase (1988: 117)
- **Type status:** Other material. **Location:** countryCode: BG; locality: Mouth of Ropotamo River; **Record Level:** bibliographicCitation: Hieke & Wrase (1988: 117)

#### Acupalpus (Acupalpus) notatus

Mulsant & Rey, 1861

##### Materials

- **Type status:** Other material. **Location:** countryCode: BG; locality: Tsarevo (= Micurin); **Record Level:** bibliographicCitation: Hieke & Wrase (1988: 118)
- **Type status:** Other material. **Location:** countryCode: BG; locality: Mouth of Ropotamo River; **Record Level:** bibliographicCitation: Hieke & Wrase (1988: 118)
- **Type status:** Other material. **Occurrence:** recordedBy: V. Lazarov; individualCount: 5; **Location:** countryCode: BG; locality: Primorsko; **Event:** eventDate: VI.1957; **Record Level:** institutionCode: NMNHS

#### Acupalpus (Acupalpus) paludicola

Reitter, 1884

##### Materials

- **Type status:** Other material. **Occurrence:** recordedBy: R. Kostova; individualCount: 1; **Location:** countryCode: BG; locality: Bulgari Vill., PA "Marina reka"; verbatimElevation: 172; verbatimCoordinates: N42°06'41.7", E27°45'53.0"; geodeticDatum: WGS84; **Event:** eventDate: 28/05/2010
- **Type status:** Other material. **Location:** countryCode: BG; locality: Primorsko; **Record Level:** bibliographicCitation: Hieke & Wrase (1988: 117)
- **Type status:** Other material. **Location:** countryCode: BG; locality: Mouth of Ropotamo River; **Record Level:** bibliographicCitation: Hieke & Wrase (1988: 117)

#### Acupalpus (Acupalpus) parvulus

(Sturm, 1825)

##### Materials

- **Type status:** Other material. **Occurrence:** recordedBy: I. Gijonov; individualCount: 1; **Location:** countryCode: BG; locality: Sinemorets Vill., PA"Silistar", "Lipite" Beach; verbatimElevation: 54; verbatimCoordinates: N42°02'40.2", E27°59'21.8"; geodeticDatum: WGS84; **Event:** eventDate: 29/05/2010

#### Acupalpus (Acupalpus) suturalis

Dejean, 1829

##### Materials

- **Type status:** Other material. **Occurrence:** recordedBy: R. Bekchiev; individualCount: 1; **Location:** countryCode: TR; locality: Sarpdere Vill., Dupnisa Cave surroundings; verbatimElevation: 440; verbatimCoordinates: N41°49'56.3", E27°33'24.0"; geodeticDatum: WGS84; **Event:** eventDate: 08/07/2009
- **Type status:** Other material. **Location:** countryCode: BG; locality: Primorsko; **Record Level:** bibliographicCitation: Hieke & Wrase (1988: 117)
- **Type status:** Other material. **Location:** countryCode: BG; locality: Strandzha; **Record Level:** bibliographicCitation: Guéorguiev & Guéorguiev (1995: 174)

#### Anthracus consputus

(Duftschmid, 1812)

##### Materials

- **Type status:** Other material. **Occurrence:** recordedBy: R. Bekchiev; individualCount: 6; **Location:** countryCode: BG; locality: Sinemorets Vill., PA "Estuary of Veleka river"; verbatimElevation: 6; verbatimCoordinates: N42°03'39.6", E27°57'55.2"; geodeticDatum: WGS84; **Event:** eventDate: 01-02.07.2009; habitat: forest & meadow
- **Type status:** Other material. **Occurrence:** recordedBy: R. Kostova; individualCount: 1; **Location:** countryCode: TR; locality: Igneada; verbatimElevation: 3; verbatimCoordinates: N41°52'51.3", E27°59'25.5"; geodeticDatum: WGS84; **Event:** eventDate: 25/05/2010
- **Type status:** Other material. **Location:** countryCode: BG; locality: Primorsko; **Record Level:** bibliographicCitation: Hieke & Wrase (1988: 119)

#### Anthracus longicornis

(Schaum, 1857)

##### Materials

- **Type status:** Other material. **Occurrence:** recordedBy: R. Kostova; individualCount: 1; **Location:** countryCode: BG; locality: Sinemorets Vill., PA "Estuary of Veleka river"; verbatimElevation: 6; verbatimCoordinates: N42°03'39.6", E27°57'55.2"; geodeticDatum: WGS84; **Event:** eventDate: 16/04/2009; habitat: meadow
- **Type status:** Other material. **Occurrence:** recordedBy: R. Kostova; individualCount: 1; **Location:** countryCode: TR; locality: Igneada, Hamam Gölü; verbatimElevation: 11; verbatimCoordinates: N41°49'31.6", E27°57'33.8"; geodeticDatum: WGS84; **Event:** eventDate: 02/10/2009

#### Anthracus quarnerensis

(Reitter, 1884)

##### Materials

- **Type status:** Other material. **Location:** countryCode: BG; locality: Kiten; **Record Level:** bibliographicCitation: Hieke & Wrase (1988: 119)
- **Type status:** Other material. **Location:** countryCode: BG; locality: Kiten; **Record Level:** bibliographicCitation: Guéorguiev & Guéorguiev (1995: 177)

#### Stenolophus (Egardoma) marginatus

Dejean, 1829

##### Materials

- **Type status:** Other material. **Occurrence:** recordedBy: R. Bekchiev; individualCount: 1; **Location:** countryCode: BG; locality: Zvezdets Vill., "Kovach" Place; verbatimElevation: 189; verbatimCoordinates: N42°04'54.8", E27°25'55.9"; geodeticDatum: WGS84; **Event:** eventDate: 09/06/2009

#### Stenolophus (Stenolophus) abdominalis persicus

Mannerheim, 1844

##### Materials

- **Type status:** Other material. **Location:** countryCode: BG; locality: Tsarevo (= Micurin); **Record Level:** bibliographicCitation: Hieke & Wrase (1988: 115)
- **Type status:** Other material. **Occurrence:** recordedBy: P. Beron; individualCount: 1; **Location:** countryCode: BG; locality: Kiten; **Event:** eventDate: 16-22.12.1984; **Record Level:** institutionCode: NMNHS
- **Type status:** Other material. **Occurrence:** recordedBy: B. Guéorguiev; individualCount: 1; **Location:** countryCode: BG; locality: mouth of Veleka River near Sinemorets Vill.; **Event:** eventDate: 28/05/1995; **Record Level:** institutionCode: NMNHS

#### Stenolophus (Stenolophus) discophorus

(Fischer-Waldheim, 1823)

##### Materials

- **Type status:** Other material. **Occurrence:** recordedBy: R. Bekchiev; individualCount: 3; **Location:** countryCode: BG; locality: Fazanovo Vill., "Popovi skali" Place; verbatimElevation: 26; verbatimCoordinates: N42°09'45.9", E27°44'15.6"; geodeticDatum: WGS84; **Event:** eventDate: 01/07/2009
- **Type status:** Other material. **Occurrence:** recordedBy: R. Bekchiev; individualCount: 2; **Location:** countryCode: BG; locality: Sinemorets Vill., PA "Estuary of Veleka river"; verbatimElevation: 5; verbatimCoordinates: N42°03'39.6", E27°57'55.2"; geodeticDatum: WGS84; **Event:** eventDate: 02/07/2009
- **Type status:** Other material. **Occurrence:** recordedBy: R. Bekchiev; individualCount: 1; **Location:** countryCode: BG; locality: Varvara Vill. surroundings; verbatimElevation: 46; verbatimCoordinates: N42°06'49.1", E27°53'18.9"; geodeticDatum: WGS84; **Event:** eventDate: 02/07/2009
- **Type status:** Other material. **Occurrence:** recordedBy: R. Kostova; individualCount: 1; **Location:** countryCode: TR; locality: Sarpdere Vill., Dupnisa Cave surroundings; verbatimElevation: 440; verbatimCoordinates: N41°49'56.3", E27°33'24.0"; geodeticDatum: WGS84; **Event:** eventDate: 08/07/2009

#### Stenolophus (Stenolophus) mixtus

(Herbst, 1784)

##### Materials

- **Type status:** Other material. **Occurrence:** recordedBy: R. Kostova; individualCount: 1; **Location:** countryCode: BG; locality: Primorsko, Perla Beach; verbatimElevation: 1; verbatimCoordinates: N42°17'10.2", E27°45'13.6"; geodeticDatum: WGS84; **Event:** eventDate: 10/05/2009
- **Type status:** Other material. **Occurrence:** recordedBy: R. Bekchiev; individualCount: 3; **Location:** countryCode: BG; locality: Primorsko, "Arcutino" Place; verbatimElevation: 60; verbatimCoordinates: N42°20'16.0", E27°43'18.1"; geodeticDatum: WGS84; **Event:** eventDate: 30/06/2009
- **Type status:** Other material. **Occurrence:** recordedBy: R. Bekchiev; individualCount: 9; **Location:** countryCode: BG; locality: Primorsko, Perla Beach; verbatimElevation: 1; verbatimCoordinates: N42°17'10.2", E27°45'13.6"; geodeticDatum: WGS84; **Event:** eventDate: 30/06/2009
- **Type status:** Other material. **Occurrence:** recordedBy: R. Bekchiev; individualCount: 5; **Location:** countryCode: BG; locality: Sinemorets Vill., PA "Estuary of Veleka river"; verbatimElevation: 5; verbatimCoordinates: N42°03'39.6", E27°57'55.2"; geodeticDatum: WGS84; **Event:** eventDate: 02/07/2009
- **Type status:** Other material. **Occurrence:** recordedBy: R. Kostova; individualCount: 2; **Location:** countryCode: TR; locality: Igneada surroundings; verbatimElevation: 3; verbatimCoordinates: N41°51'27.3", E27°57'28.7"; geodeticDatum: WGS84; **Event:** eventDate: 06/07/2009; habitat: marsh
- **Type status:** Other material. **Occurrence:** recordedBy: R. Kostova; individualCount: 1; **Location:** countryCode: TR; locality: Igneada, Hamam Gölü; verbatimElevation: 11; verbatimCoordinates: N41°49'31.6", E27°57'33.8"; geodeticDatum: WGS84; **Event:** eventDate: 02/10/2009
- **Type status:** Other material. **Occurrence:** recordedBy: R. Kostova; individualCount: 1; **Location:** countryCode: TR; locality: Igneada surroundings; verbatimElevation: 3; verbatimCoordinates: N41°51'27.3", E27°57'28.7"; geodeticDatum: WGS84; **Event:** eventDate: 02/10/2009; habitat: marsh
- **Type status:** Other material. **Occurrence:** recordedBy: R. Kostova; individualCount: 1; **Location:** countryCode: TR; locality: Igneada, Hamam Gölü; verbatimElevation: 11; verbatimCoordinates: N41°49'31.6", E27°57'33.8"; geodeticDatum: WGS84; **Event:** eventDate: 25/05/2010
- **Type status:** Other material. **Occurrence:** recordedBy: R. Kostova; individualCount: 1; **Location:** countryCode: BG; locality: Sinemorets Vill., PA "Estuary of Veleka river"; verbatimElevation: 6; verbatimCoordinates: N42°03'39.6", E27°57'55.2"; geodeticDatum: WGS84; **Event:** eventDate: 15.04-08.05.2009; habitat: swamp forest
- **Type status:** Other material. **Occurrence:** recordedBy: R. Kostova; individualCount: 1; **Location:** countryCode: BG; locality: Sinemorets Vill., PA "Estuary of Veleka river"; verbatimElevation: 6; verbatimCoordinates: N42°03'39.6", E27°57'55.2"; geodeticDatum: WGS84; **Event:** eventDate: 09.05-08.06.2009; habitat: swamp forest
- **Type status:** Other material. **Location:** countryCode: BG; locality: Tsarevo (= Micurin); **Record Level:** bibliographicCitation: Hieke & Wrase (1988: 114)
- **Type status:** Other material. **Location:** countryCode: BG; locality: Mouth of Ropotamo River; **Record Level:** bibliographicCitation: Hieke & Wrase (1988: 114)
- **Type status:** Other material. **Occurrence:** recordedBy: B. Guéorguiev; individualCount: 2; **Location:** countryCode: BG; locality: mouth of Veleka River near Sinemorets Vill.; **Event:** eventDate: 28/05/1995; **Record Level:** institutionCode: NMNHS

#### Stenolophus (Stenolophus) proximus

Dejean, 1829

##### Materials

- **Type status:** Other material. **Occurrence:** recordedBy: R. Bekchiev; individualCount: 1; **Location:** countryCode: BG; locality: Zvezdets Vill., "Kovach" Place; verbatimElevation: 189; verbatimCoordinates: N42°04'54.8", E27°25'55.9"; geodeticDatum: WGS84; **Event:** eventDate: 09/06/2009
- **Type status:** Other material. **Occurrence:** recordedBy: R. Kostova; individualCount: 1; **Location:** countryCode: TR; locality: Igneada surroundings; verbatimElevation: 3; verbatimCoordinates: N41°51'27.4", E27°57'32.2"; geodeticDatum: WGS84; **Event:** eventDate: 05/07/2009; habitat: marsh
- **Type status:** Other material. **Location:** countryCode: BG; locality: Primorsko; **Record Level:** bibliographicCitation: Hieke & Wrase (1988: 114)

#### Stenolophus (Stenolophus) skrimshiranus

Stephens, 1828

##### Materials

- **Type status:** Other material. **Occurrence:** recordedBy: R. Kostova; individualCount: 1; **Location:** countryCode: BG; locality: Sinemorets Vill., PA "Estuary of Veleka river"; verbatimElevation: 5; verbatimCoordinates: N42°03'39.6", E27°57'55.2"; geodeticDatum: WGS84; **Event:** eventDate: 16/04/2009
- **Type status:** Other material. **Occurrence:** recordedBy: R. Bekchiev; individualCount: 1; **Location:** countryCode: BG; locality: Primorsko, "Arcutino" Place; verbatimElevation: 60; verbatimCoordinates: N42°20'16.0", E27°43'18.1"; geodeticDatum: WGS84; **Event:** eventDate: 30/06/2009
- **Type status:** Other material. **Occurrence:** recordedBy: R. Bekchiev; individualCount: 1; **Location:** countryCode: BG; locality: Primorsko, Perla Beach; verbatimElevation: 1; verbatimCoordinates: N42°17'10.2", E27°45'13.6"; geodeticDatum: WGS84; **Event:** eventDate: 30/06/2009
- **Type status:** Other material. **Occurrence:** recordedBy: R. Bekchiev; individualCount: 1; **Location:** countryCode: BG; locality: Sinemorets Vill., PA "Estuary of Veleka river"; verbatimElevation: 5; verbatimCoordinates: N42°03'39.6", E27°57'55.2"; geodeticDatum: WGS84; **Event:** eventDate: 02/07/2009
- **Type status:** Other material. **Occurrence:** recordedBy: R. Bekchiev; individualCount: 1; **Location:** countryCode: TR; locality: Igneada surroundings; verbatimElevation: 3; verbatimCoordinates: N41°51'27.4", E27°57'32.2"; geodeticDatum: WGS84; **Event:** eventDate: 05/07/2009; habitat: marsh

#### Stenolophus (Stenolophus) steveni

Krynicki, 1832

##### Materials

- **Type status:** Other material. **Location:** countryCode: BG; locality: Mouth of Ropotamo River; **Record Level:** bibliographicCitation: Hieke & Wrase (1988: 114)

#### Stenolophus (Stenolophus) teutonus

(Schrank, 1781)

##### Materials

- **Type status:** Other material. **Occurrence:** recordedBy: R. Kostova; individualCount: 2; **Location:** countryCode: TR; locality: Kiyiköy surroundings; verbatimElevation: 38; verbatimCoordinates: N41°39'25.7", E28°05'11.2"; geodeticDatum: WGS84; **Event:** eventDate: 22/05/2011
- **Type status:** Other material. **Location:** countryCode: BG; locality: Ahtopol; **Record Level:** bibliographicCitation: Hieke & Wrase (1988: 115)
- **Type status:** Other material. **Location:** countryCode: BG; locality: Tsarevo (= Micurin); **Record Level:** bibliographicCitation: Hieke & Wrase (1988: 115)
- **Type status:** Other material. **Location:** countryCode: BG; locality: Ropotamo; **Record Level:** bibliographicCitation: Hieke & Wrase (1988: 115)
- **Type status:** Other material. **Location:** countryCode: BG; locality: 5 km West of Yasna Polyana Vill.; **Record Level:** bibliographicCitation: Hieke & Wrase (1988: 115)
- **Type status:** Other material. **Occurrence:** recordedBy: P. Beron; individualCount: 1; **Location:** countryCode: BG; locality: Kiten; **Event:** eventDate: 16-22.12.1984; **Record Level:** institutionCode: NMNHS
- **Type status:** Other material. **Occurrence:** recordedBy: V. Sakalian; individualCount: 1; **Location:** countryCode: BG; locality: Sinemorets Vill.; **Event:** eventDate: 20/06/1991; **Record Level:** institutionCode: NMNHS

#### Cymindis (Cymindis) axillaris axillaris

(Fabricius, 1794)

##### Materials

- **Type status:** Other material. **Occurrence:** recordedBy: R. Kostova; individualCount: 1; **Location:** countryCode: BG; locality: Slivarovo Vll. surroundings; verbatimElevation: 331; verbatimCoordinates: N41°58'11.9", E27°39'31.1"; geodeticDatum: WGS84; **Event:** eventDate: 15.04-08.05.2009; habitat: shrubs, mainly Calluna vulgaris
- **Type status:** Other material. **Occurrence:** recordedBy: R. Kostova; individualCount: 1; **Location:** countryCode: BG; locality: Brodilovo Vill., under Papiya peak; verbatimElevation: 296; verbatimCoordinates: N42°06'21.7", E27°50'32.6"; geodeticDatum: WGS84; **Event:** eventDate: 09.05-08.06.2009; habitat: shrubs, Phyllyrea latifolia

#### Cymindis (Cymindis) ornata

Fischer-Waldheim, 1823

##### Materials

- **Type status:** Other material. **Occurrence:** recordedBy: R. Kostova; individualCount: 1; **Location:** countryCode: BG; locality: Sinemorets Vill., PA"Silistar"; verbatimElevation: 23; verbatimCoordinates: N42°01'30.7", E28°00'25.4"; geodeticDatum: WGS84; **Event:** eventDate: 09.05-08.06.2009; habitat: oak forest
- **Type status:** Other material. **Location:** countryCode: BG; locality: Maslen nos Cape; **Record Level:** bibliographicCitation: Mařan (1933: 91)

#### Cymindis (Paracymindis) vassili

Guéorguiev, 2001

##### Materials

- **Type status:** Other material. **Location:** countryCode: BG; locality: Sinemorets Vill.; **Record Level:** bibliographicCitation: Guéorguiev (2001: 134)

#### Demetrias (Aetophorus) imperialis

(Germar, 1824)

##### Materials

- **Type status:** Other material. **Occurrence:** recordedBy: R. Bekchiev; individualCount: 1; **Location:** countryCode: BG; locality: Sinemorets Vill., PA "Estuary of Veleka river"; verbatimElevation: 5; verbatimCoordinates: N42°03'39.6", E27°57'55.2"; geodeticDatum: WGS84; **Event:** eventDate: 02/07/2009
- **Type status:** Other material. **Occurrence:** recordedBy: R. Kostova; individualCount: 3; **Location:** countryCode: TR; locality: Igneada surroundings; verbatimElevation: 3; verbatimCoordinates: N41°51'27.4", E27°57'32.2"; geodeticDatum: WGS84; **Event:** eventDate: 05/07/2009; habitat: marsh

#### Demetrias (Demetrias) atricapillus

(Linnaeus, 1758)

##### Materials

- **Type status:** Other material. **Occurrence:** recordedBy: I. Gijonov; individualCount: 1; **Location:** countryCode: BG; locality: Sinemorets Vill., PA "Estuary of Veleka river"; verbatimElevation: 6; verbatimCoordinates: N42°03'39.6", E27°57'55.2"; geodeticDatum: WGS84; **Event:** eventDate: 16/04/2009
- **Type status:** Other material. **Location:** countryCode: BG; locality: Ropotamo; **Record Level:** bibliographicCitation: Hieke & Wrase (1988: 155)

#### Dromius (Dromius) quadrimaculatus

(Linnaeus, 1758)

##### Materials

- **Type status:** Other material. **Occurrence:** recordedBy: P. Beron; individualCount: 1; **Location:** countryCode: BG; locality: Kiten; **Event:** eventDate: 16-22.12.1984; **Record Level:** institutionCode: NMNHS

#### Microlestes fissuralis

(Reitter, 1901)

##### Materials

- **Type status:** Other material. **Location:** countryCode: BG; locality: Ahtopol; **Record Level:** bibliographicCitation: Hieke & Wrase (1988: 159)
- **Type status:** Other material. **Location:** countryCode: BG; locality: Tsarevo (= Micurin); **Record Level:** bibliographicCitation: Hieke & Wrase (1988: 159)

#### Microlestes fulvibasis

(Reitter, 1901)

##### Materials

- **Type status:** Other material. **Occurrence:** recordedBy: R. Kostova; individualCount: 1; **Location:** countryCode: BG; locality: Sinemorets Vill., PA"Silistar", Estuary of Silistar River; verbatimElevation: 12; verbatimCoordinates: N42°01'26.8", E28°00'32.9"; geodeticDatum: WGS84; **Event:** eventDate: 27/05/2011
- **Type status:** Other material. **Occurrence:** recordedBy: R. Kostova; individualCount: 2; **Location:** countryCode: BG; locality: Sinemorets Vill., PA"Silistar", "Butamyata" Place; verbatimElevation: 10; verbatimCoordinates: N42°03'11.2", E27°59'19.7"; geodeticDatum: WGS84; **Event:** eventDate: 15.04-08.05.2009; habitat: meadow, single shrubs
- **Type status:** Other material. **Occurrence:** recordedBy: R. Kostova; individualCount: 1; **Location:** countryCode: TR; locality: Demirköy, along Degirmen River; verbatimElevation: 289; verbatimCoordinates: N41°49'17.2", E27°45'07.7"; geodeticDatum: WGS84; **Event:** eventDate: 06/07/2009
- **Type status:** Other material. **Occurrence:** recordedBy: R. Kostova; individualCount: 1; **Location:** countryCode: TR; locality: Kiyiköy surroundings; verbatimElevation: 32; verbatimCoordinates: N41°38'12.8", E28°04'08.7"; geodeticDatum: WGS84; **Event:** eventDate: 07/07/2009
- **Type status:** Other material. **Occurrence:** recordedBy: R. Kostova; individualCount: 2; **Location:** countryCode: TR; locality: Beğendik Vill. surroundings; verbatimElevation: 156; verbatimCoordinates: N41°56'42.7", E27°59'26.9"; geodeticDatum: WGS84; **Event:** eventDate: 28/09/2009
- **Type status:** Other material. **Occurrence:** recordedBy: R. Kostova; individualCount: 1; **Location:** countryCode: TR; locality: Avcilar Vill. surroundings; verbatimElevation: 187; verbatimCoordinates: N41°53'44.6", E27°51'55.7"; geodeticDatum: WGS84; **Event:** eventDate: 23/05/2010
- **Type status:** Other material. **Occurrence:** recordedBy: R. Kostova; individualCount: 2; **Location:** countryCode: TR; locality: Kiyiköy surroundings; verbatimElevation: 38; verbatimCoordinates: N41°39'25.7", E28°05'11.2"; geodeticDatum: WGS84; **Event:** eventDate: 24/05/2010
- **Type status:** Other material. **Occurrence:** recordedBy: R. Kostova; individualCount: 1; **Location:** countryCode: TR; locality: Along Demirköy - Kıyıköy road; verbatimElevation: 199; verbatimCoordinates: N41°39'43.2", E27°55'25.8"; geodeticDatum: WGS84; **Event:** eventDate: 24/05/2010
- **Type status:** Other material. **Occurrence:** recordedBy: R. Kostova; individualCount: 1; **Location:** countryCode: TR; locality: Yoliköy surroundings; verbatimElevation: 9; verbatimCoordinates: N41°29'27.7", E28°16'40.0"; geodeticDatum: WGS84; **Event:** eventDate: 23/05/2011

#### Microlestes luctuosus luctuosus

Holdhaus, 1904

##### Materials

- **Type status:** Other material. **Occurrence:** recordedBy: R. Kostova; individualCount: 1; **Location:** countryCode: TR; locality: Kiyiköy surroundings; verbatimElevation: 38; verbatimCoordinates: N41°39'25.8", E28°05'10.0"; geodeticDatum: WGS84; **Event:** eventDate: 24/05/2010
- **Type status:** Other material. **Occurrence:** recordedBy: R. Kostova; individualCount: 2; **Location:** countryCode: TR; locality: Igneada, Hamam Gölü; verbatimElevation: 11; verbatimCoordinates: N41°49'31.6", E27°57'33.8"; geodeticDatum: WGS84; **Event:** eventDate: 25/05/2010
- **Type status:** Other material. **Occurrence:** recordedBy: R. Kostova; individualCount: 1; **Location:** countryCode: TR; locality: Sarpdere Vill., Dupnisa Cave surroundings; verbatimElevation: 440; verbatimCoordinates: N41°49'56.3", E27°33'24.0"; geodeticDatum: WGS84; **Event:** eventDate: 26/05/2011; habitat: meadow
- **Type status:** Other material. **Location:** countryCode: BG; locality: Tsarevo (= Micurin); **Record Level:** bibliographicCitation: Hieke & Wrase (1988: 159)

#### Microlestes maurus maurus

(Sturm, 1827)

##### Materials

- **Type status:** Other material. **Location:** countryCode: BG; locality: Ahtopol; **Record Level:** bibliographicCitation: Hieke & Wrase (1988: 159)

#### Microlestes minutulus

(Goeze, 1777)

##### Materials

- **Type status:** Other material. **Location:** countryCode: BG; locality: Malko Tarnovo; **Record Level:** bibliographicCitation: Guéorguiev & Guéorguiev (1995: 230)
- **Type status:** Other material. **Location:** countryCode: BG; locality: Tsarevo (= Micurin); **Record Level:** bibliographicCitation: Hieke & Wrase (1988: 158)

#### Microlestes negrita nеgrita

(Wollaston, 1854)

##### Materials

- **Type status:** Other material. **Occurrence:** recordedBy: R. Kostova; individualCount: 1; **Location:** countryCode: BG; locality: Stoilovo Vill., "Petrova niva" Place; verbatimElevation: 132; verbatimCoordinates: N42°03'41.9", E27°31'58.9"; geodeticDatum: WGS84; **Event:** eventDate: 06/05/2010; habitat: meadow, single shrubs
- **Type status:** Other material. **Occurrence:** recordedBy: R. Kostova; individualCount: 1; **Location:** countryCode: BG; locality: Rezovo Vill. surroundings; verbatimElevation: 5; verbatimCoordinates: N41°58'54.4", E28°01'29.6"; geodeticDatum: WGS84; **Event:** eventDate: 09/05/2009; habitat: meadows
- **Type status:** Other material. **Occurrence:** recordedBy: R. Kostova; individualCount: 1; **Location:** countryCode: TR; locality: Kiyiköy surroundings; verbatimElevation: 38; verbatimCoordinates: N41°39'25.7", E28°05'11.2"; geodeticDatum: WGS84; **Event:** eventDate: 30/05/2009
- **Type status:** Other material. **Occurrence:** recordedBy: R. Kostova; individualCount: 2; **Location:** countryCode: TR; locality: Kiyiköy surroundings; verbatimElevation: 62; verbatimCoordinates: N41°37'40.7", E28°04'05.0"; geodeticDatum: WGS84; **Event:** eventDate: 22/05/2011
- **Type status:** Other material. **Occurrence:** recordedBy: R. Kostova; individualCount: 1; **Location:** countryCode: TR; locality: Avcilar Vill. surroundings; verbatimElevation: 187; verbatimCoordinates: N41°53'44.6", E27°51'55.7"; geodeticDatum: WGS84; **Event:** eventDate: 23/05/2010
- **Type status:** Other material. **Occurrence:** recordedBy: R. Kostova; individualCount: 1; **Location:** countryCode: TR; locality: Kiyiköy surroundings; verbatimElevation: 38; verbatimCoordinates: N41°39'25.7", E28°05'11.2"; geodeticDatum: WGS84; **Event:** eventDate: 24/05/2010
- **Type status:** Other material. **Occurrence:** recordedBy: J. Mařan & K. Taborsky; individualCount: 2; **Location:** countryCode: BG; locality: Maslen nos Cape; **Event:** eventDate: VI.1933; **Record Level:** institutionCode: NMNHS & NMP
- **Type status:** Other material. **Location:** countryCode: BG; locality: Ahtopol; **Record Level:** bibliographicCitation: Hieke & Wrase (1988: 158)
- **Type status:** Other material. **Location:** countryCode: BG; locality: Tsarevo (= Micurin); **Record Level:** bibliographicCitation: Hieke & Wrase (1988: 158)
- **Type status:** Other material. **Location:** countryCode: BG; locality: Mouth of Ropotamo River; **Record Level:** bibliographicCitation: Hieke & Wrase (1988: 158)
- **Type status:** Other material. **Occurrence:** recordedBy: B. Guéorguiev; individualCount: 3; **Location:** countryCode: BG; locality: Veleka River near Mladezko Village; **Event:** eventDate: 25/05/1995; **Record Level:** institutionCode: NMNHS

#### Paradromius (Manodromius) linearis linearis

(Olivier, 1795)

##### Materials

- **Type status:** Other material. **Occurrence:** recordedBy: I. Gijonov; individualCount: 1; **Location:** countryCode: BG; locality: Rezovo Vill. surroundings; verbatimElevation: 5; verbatimCoordinates: N41°58'54.4", E28°01'29.6"; geodeticDatum: WGS84; **Event:** eventDate: 09/05/2009; habitat: meadows
- **Type status:** Other material. **Location:** countryCode: BG; locality: Strandzha; **Record Level:** bibliographicCitation: Rambousek (1912: 100, as Dromius linearis)
- **Type status:** Other material. **Location:** countryCode: BG; locality: Gramatikovo Vill.; **Record Level:** bibliographicCitation: Hieke & Wrase (1988: 155, as Dromius linearis)
- **Type status:** Other material. **Location:** countryCode: BG; locality: Tsarevo (= Micurin); **Record Level:** bibliographicCitation: Hieke & Wrase (1988: 155, as Dromius linearis)
- **Type status:** Other material. **Location:** countryCode: BG; locality: Primorsko; **Record Level:** bibliographicCitation: Hieke & Wrase (1988: 155, as Dromius linearis)
- **Type status:** Other material. **Occurrence:** recordedBy: S. Petrov; individualCount: 1; **Location:** countryCode: BG; locality: Vitanovo Forest Reserve; **Event:** eventDate: 23.09-5.10.1999; habitat: maialise trap; **Record Level:** institutionCode: NMNHS

#### Paradromius (Paradromius) longiceps

(Dejean, 1826)

##### Materials

- **Type status:** Other material. **Location:** countryCode: BG; locality: mouth of Veleka River near Sinemorets Vill.; **Record Level:** bibliographicCitation: Guéorguiev & Muilwijk (2000: 82)

#### Philorihzus notatus

(Stephens, 1827)

##### Materials

- **Type status:** Other material. **Occurrence:** recordedBy: R. Kostova; individualCount: 1; **Location:** countryCode: BG; locality: Brodilovo Vill., under Papiya peak; verbatimElevation: 296; verbatimCoordinates: N42°06'21.7", E27°50'32.6"; geodeticDatum: WGS84; **Event:** eventDate: 09.06-02.07.2009; habitat: shrubs, Phyllyrea latifolia

#### Lebia (Lamprias) cyanocephala cyanocephala

(Linnaeus, 1758)

##### Materials

- **Type status:** Other material. **Location:** countryCode: BG; locality: Kosti Vill.; **Record Level:** bibliographicCitation: Guéorguiev & Guéorguiev (1995: 222)
- **Type status:** Other material. **Location:** countryCode: BG; locality: Rezovo Vill.; **Record Level:** bibliographicCitation: Guéorguiev & Guéorguiev (1995: 222)

#### Lebia (Lebia) cruxminor cruxminor

(Linnaeus, 1758)

##### Materials

- **Type status:** Other material. **Occurrence:** recordedBy: R. Kostova; individualCount: 1; **Location:** countryCode: BG; locality: Primorsko, Perla Beach; verbatimElevation: 1; verbatimCoordinates: N42°17'10.2", E27°45'13.6"; geodeticDatum: WGS84; **Event:** eventDate: 10/05/2009
- **Type status:** Other material. **Location:** countryCode: BG; locality: Strandzha; **Record Level:** bibliographicCitation: Rambousek (1912: 99)

#### Lebia (Lebia) humeralis

Dejean, 1825

##### Materials

- **Type status:** Other material. **Occurrence:** recordedBy: R. Kostova; individualCount: 3; **Location:** countryCode: BG; locality: Primorsko, Perla Beach; verbatimElevation: 1; verbatimCoordinates: N42°17'10.2", E27°45'13.6"; geodeticDatum: WGS84; **Event:** eventDate: 10/05/2009
- **Type status:** Other material. **Occurrence:** recordedBy: W. Beier; individualCount: 1; **Location:** countryCode: BG; locality: Primorsko; **Event:** eventDate: 15/05/2003; **Record Level:** collectionCode: cWB
- **Type status:** Other material. **Location:** countryCode: BG; locality: Kiten; **Record Level:** bibliographicCitation: Hieke & Wrase (1988: 154)

#### Lebia (Lebia) marginata

(Geoffroy, 1785)

##### Materials

- **Type status:** Other material. **Occurrence:** recordedBy: W. Beier; individualCount: 1; **Location:** countryCode: BG; locality: Bogdanovo Vill.; verbatimElevation: 300; **Event:** eventDate: 06/05/2002; **Record Level:** collectionCode: cWB

#### Lebia (Lebia) trimaculata

(Villers, 1789)

##### Materials

- **Type status:** Other material. **Location:** countryCode: BG; locality: Strandzha; **Record Level:** bibliographicCitation: Rambousek (1912: 99)

#### Lionychus (Lionychus) quadrillum

(Duftschmid, 1812)

##### Materials

- **Type status:** Other material. **Occurrence:** recordedBy: R. Kostova; individualCount: 1; **Location:** countryCode: BG; locality: Slivarovo Vill., "Shafariitsa" Place; verbatimElevation: 224; verbatimCoordinates: N41°57'37.8", E27°39'34.8"; geodeticDatum: WGS84; **Event:** eventDate: 07/05/2009
- **Type status:** Other material. **Occurrence:** recordedBy: B. Guéorguiev; individualCount: 1; **Location:** countryCode: BG; locality: Sinemorets Village, south bay; **Event:** eventDate: 27/05/1995; **Record Level:** institutionCode: NMNHS

#### Syntomus impressus impressus

(Dejean, 1825)

##### Materials

- **Type status:** Other material. **Location:** countryCode: BG; locality: Primorsko; **Record Level:** bibliographicCitation: Hieke & Wrase (1988: 158)

#### Syntomus obscuroguttatus

(Duftshmid, 1812)

##### Materials

- **Type status:** Other material. **Occurrence:** recordedBy: R. Kostova; individualCount: 1; **Location:** countryCode: BG; locality: Sinemorets Vill., PA "Estuary of Veleka river"; verbatimElevation: 6; verbatimCoordinates: N42°03'39.6", E27°57'55.2"; geodeticDatum: WGS84; **Event:** eventDate: 16/04/2009
- **Type status:** Other material. **Occurrence:** recordedBy: R. Bekchiev; individualCount: 4; **Location:** countryCode: BG; locality: Sinemorets Vill., PA"Silistar", "Butamyata" Place; verbatimElevation: 17; verbatimCoordinates: N42°03'10.3", E27°59'13.4"; geodeticDatum: WGS84; **Event:** eventDate: 16/04/2009; habitat: oak forest
- **Type status:** Other material. **Occurrence:** recordedBy: R. Bekchiev; individualCount: 1; **Location:** countryCode: BG; locality: Kirovo Vill., along Selska River; verbatimElevation: 138; verbatimCoordinates: N42°10'43.0", E27°10'58.2"; geodeticDatum: WGS84; **Event:** eventDate: 05/05/2009
- **Type status:** Other material. **Occurrence:** recordedBy: R. Bekchiev; individualCount: 1; **Location:** countryCode: BG; locality: Sinemorets Vill., PA "Estuary of Veleka river"; verbatimElevation: 6; verbatimCoordinates: N42°03'39.6", E27°57'55.2"; geodeticDatum: WGS84; **Event:** eventDate: 01/07/2009
- **Type status:** Other material. **Occurrence:** recordedBy: A. Gijonova; individualCount: 1; **Location:** countryCode: TR; locality: Igneada surroundings; verbatimElevation: 3; verbatimCoordinates: N41°51'27.3", E27°57'28.7"; geodeticDatum: WGS84; **Event:** eventDate: 02/10/2009; habitat: marsh
- **Type status:** Other material. **Occurrence:** recordedBy: R. Kostova; individualCount: 2; **Location:** countryCode: BG; locality: Kosti Vill., "St. Ilia" Place; verbatimElevation: 35; verbatimCoordinates: N42°03'23.2", E27°45'51.6"; geodeticDatum: WGS84; **Event:** eventDate: 09.06-02.08.2009; habitat: meadow with single trees
- **Type status:** Other material. **Occurrence:** recordedBy: J. Mařan & K. Taborsky; individualCount: 1; **Location:** countryCode: BG; locality: Maslen nos Cape; **Event:** eventDate: VI.1933; **Record Level:** institutionCode: NMP
- **Type status:** Other material. **Location:** countryCode: BG; locality: Mouth of Ropotamo River; **Record Level:** bibliographicCitation: Hieke & Wrase (1988: 157)
- **Type status:** Other material. **Occurrence:** recordedBy: P. Beron; individualCount: 7; **Location:** countryCode: BG; locality: Kiten; **Event:** eventDate: 16-22.12.1984; **Record Level:** institutionCode: NMNHS
- **Type status:** Other material. **Occurrence:** recordedBy: B. Guéorguiev; individualCount: 1; **Location:** countryCode: BG; locality: Veleka River near Mladezko Village; **Event:** eventDate: 25/05/1995; **Record Level:** institutionCode: NMNHS

#### Syntomus pallipes

(Dejean, 1825)

##### Materials

- **Type status:** Other material. **Occurrence:** recordedBy: R. Kostova & R. Bekchiev; individualCount: 2; **Location:** countryCode: BG; locality: Kirovo Vill., along Selska River; verbatimElevation: 138; verbatimCoordinates: N42°10'43.0", E27°10'58.2"; geodeticDatum: WGS84; **Event:** eventDate: 05/05/2009
- **Type status:** Other material. **Occurrence:** recordedBy: R. Kostova; individualCount: 1; **Location:** countryCode: BG; locality: Rezovo Vill. surroundings; verbatimElevation: 5; verbatimCoordinates: N41°58'54.4", E28°01'29.6"; geodeticDatum: WGS84; **Event:** eventDate: 09/05/2009; habitat: meadows
- **Type status:** Other material. **Occurrence:** recordedBy: R. Kostova; individualCount: 1; **Location:** countryCode: BG; locality: Primorsko, Dunes; verbatimElevation: 6; verbatimCoordinates: N41°47'44.0", E27°59'50.4"; geodeticDatum: WGS84; **Event:** eventDate: 10/05/2009
- **Type status:** Other material. **Occurrence:** recordedBy: R. Bekchiev; individualCount: 4; **Location:** countryCode: BG; locality: Slivarovo Vill., "Shafariitsa" Place; verbatimElevation: 224; verbatimCoordinates: N41°57'37.8", E27°39'34.8"; geodeticDatum: WGS84; **Event:** eventDate: 25/09/2009
- **Type status:** Other material. **Occurrence:** recordedBy: I. Gijonov; individualCount: 1; **Location:** countryCode: BG; locality: Kosti Vill., "St. Ilia" Place; verbatimElevation: 35; verbatimCoordinates: N42°03'23.2", E27°45'51.6"; geodeticDatum: WGS84; **Event:** eventDate: 27/09/2009; habitat: meadow with single trees
- **Type status:** Other material. **Occurrence:** recordedBy: A. Gijonova; individualCount: 1; **Location:** countryCode: TR; locality: Igneada surroundings; verbatimElevation: 3; verbatimCoordinates: N41°51'27.3", E27°57'28.7"; geodeticDatum: WGS84; **Event:** eventDate: 02/10/2009; habitat: marsh
- **Type status:** Other material. **Occurrence:** recordedBy: R. Kostova; individualCount: 3; **Location:** countryCode: TR; locality: Igneada, Hamam Gölü; verbatimElevation: 11; verbatimCoordinates: N41°49'31.6", E27°57'33.8"; geodeticDatum: WGS84; **Event:** eventDate: 25/05/2010
- **Type status:** Other material. **Occurrence:** recordedBy: R. Kostova; individualCount: 1; **Location:** countryCode: BG; locality: Stoilovo Vill., "Sredoka" Reserve, along Mechi dol River; verbatimElevation: 206; verbatimCoordinates: N42°01'51.1", E27°30'50.1"; geodeticDatum: WGS84; **Event:** eventDate: 27/05/2010
- **Type status:** Other material. **Occurrence:** recordedBy: R. Kostova; individualCount: 4; **Location:** countryCode: TR; locality: Yaliköy, river estuary; verbatimElevation: 9; verbatimCoordinates: N41°29'27.7", E28°16'40.0"; geodeticDatum: WGS84; **Event:** eventDate: 23/05/2011
- **Type status:** Other material. **Occurrence:** recordedBy: R. Bekchiev; individualCount: 5; **Location:** countryCode: TR; locality: Demirköy, Mahya Dağı Peak; verbatimElevation: 820; verbatimCoordinates: N41°46'15.6", E27°38'18.0"; geodeticDatum: WGS84; **Event:** eventDate: 30/09/2009
- **Type status:** Other material. **Occurrence:** recordedBy: R. Kostova; individualCount: 2; **Location:** countryCode: BG; locality: Sinemorets Vill., PA"Silistar", Estuary of Silistar River; verbatimElevation: 12; verbatimCoordinates: N42°01'26.8", E28°00'32.9"; geodeticDatum: WGS84; **Event:** eventDate: 27/05/2011
- **Type status:** Other material. **Occurrence:** recordedBy: R. Kostova; individualCount: 1; **Location:** countryCode: BG; locality: Slivarovo Vill., "Shafariitsa" Place; verbatimElevation: 224; verbatimCoordinates: N41°57'37.8", E27°39'34.8"; geodeticDatum: WGS84; **Event:** eventDate: 15.04-08.05.2009; habitat: black alder-oak forest
- **Type status:** Other material. **Occurrence:** recordedBy: R. Kostova; individualCount: 1; **Location:** countryCode: BG; locality: Slivarovo Vill., "Shafariitsa" Place; verbatimElevation: 224; verbatimCoordinates: N41°57'37.8", E27°39'34.8"; geodeticDatum: WGS84; **Event:** eventDate: 09.06-02.07.2009; habitat: black alder-oak forest
- **Type status:** Other material. **Occurrence:** recordedBy: R. Kostova; individualCount: 2; **Location:** countryCode: BG; locality: Kosti Vill., "St. Ilia" Place; verbatimElevation: 35; verbatimCoordinates: N42°03'23.2", E27°45'51.6"; geodeticDatum: WGS84; **Event:** eventDate: 09.05-08.06.2009; habitat: meadow with single trees
- **Type status:** Other material. **Occurrence:** recordedBy: R. Kostova; individualCount: 1; **Location:** countryCode: BG; locality: Stoilovo Vill., "Petrova niva" Place; verbatimElevation: 234; verbatimCoordinates: N42°03'40.3", E27°31'42.3"; geodeticDatum: WGS84; **Event:** eventDate: 09.05-08.06.2009; habitat: meadow, single shrubs
- **Type status:** Other material. **Location:** countryCode: BG; locality: Tsarevo (= Micurin); **Record Level:** bibliographicCitation: Hieke & Wrase (1988: 157)
- **Type status:** Other material. **Location:** countryCode: BG; locality: Primorsko; **Record Level:** bibliographicCitation: Hieke & Wrase (1988: 157)
- **Type status:** Other material. **Location:** countryCode: BG; locality: Mouth of Ropotamo River; **Record Level:** bibliographicCitation: Hieke & Wrase (1988: 157)

#### Badister (Badister) bullatus

(Schrank, 1798)

##### Materials

- **Type status:** Other material. **Occurrence:** recordedBy: R. Kostova; individualCount: 2; **Location:** countryCode: BG; locality: Kosti Vill., "St. Ilia" Place; verbatimElevation: 35; verbatimCoordinates: N42°03'23.2", E27°45'51.6"; geodeticDatum: WGS84; **Event:** eventDate: 15.04-08.06.2009; habitat: meadow with single trees
- **Type status:** Other material. **Occurrence:** recordedBy: R. Kostova; individualCount: 1; **Location:** countryCode: BG; locality: Sinemorets Vill., PA "Estuary of Veleka river"; verbatimElevation: 6; verbatimCoordinates: N42°03'39.6", E27°57'55.2"; geodeticDatum: WGS84; **Event:** eventDate: 03.08-07.09.2009; habitat: swamp forest

#### Badister (Badister) unipustulatus

Bonelli, 1813

##### Materials

- **Type status:** Other material. **Occurrence:** recordedBy: R. Bekchiev; individualCount: 2; **Location:** countryCode: BG; locality: Fazanovo Vill., "Popovi skali" Place; verbatimElevation: 26; verbatimCoordinates: N42°09'45.9", E27°44'15.6"; geodeticDatum: WGS84; **Event:** eventDate: 01/07/2009

#### Badister (Baudia) collaris

Motschulsky, 1844

##### Materials

- **Type status:** Other material. **Occurrence:** recordedBy: R. Bekchiev; individualCount: 1; **Location:** countryCode: BG; locality: Fazanovo Vill., "Popovi skali" Place; verbatimElevation: 26; verbatimCoordinates: N42°09'45.9", E27°44'15.6"; geodeticDatum: WGS84; **Event:** eventDate: 01/07/2009
- **Type status:** Other material. **Location:** countryCode: BG; locality: Mouth of Ropotamo River; **Record Level:** bibliographicCitation: Hieke & Wrase (1988: 151, as B. anomalus)
- **Type status:** Other material. **Occurrence:** recordedBy: P. Beron; individualCount: 1; **Location:** countryCode: BG; locality: Kiten; **Event:** eventDate: 16-22.12.1984; **Record Level:** institutionCode: NMNHS

#### Badister (Trimorphus) sodalis

(Duftschmid, 1812)

##### Materials

- **Type status:** Other material. **Occurrence:** recordedBy: R. Bekchiev; individualCount: 2; **Location:** countryCode: BG; locality: Kirovo Vill., along Selska River; verbatimElevation: 138; verbatimCoordinates: N42°10'43.0", E27°10'58.2"; geodeticDatum: WGS84; **Event:** eventDate: 05/05/2009
- **Type status:** Other material. **Occurrence:** recordedBy: R. Kostova; individualCount: 1; **Location:** countryCode: BG; locality: Brashlyan Vill.; verbatimElevation: 341; verbatimCoordinates: N42°02'41.0", E27°25'25.5"; geodeticDatum: WGS84; **Event:** eventDate: 22/08/2009
- **Type status:** Other material. **Occurrence:** recordedBy: B. Guéorguiev; individualCount: 1; **Location:** countryCode: BG; locality: Rezovska River at Uzunbudzhak Reserve; **Event:** eventDate: 26/05/1995; **Record Level:** institutionCode: NMNHS

#### Licinus (Licinus) cassideus cassideus

(Fabricius, 1792)

##### Materials

- **Type status:** Other material. **Occurrence:** recordedBy: R. Kostova; individualCount: 1; **Location:** countryCode: BG; locality: Bliznak Vill., PA"Bataka"; verbatimElevation: 324; verbatimCoordinates: N42°11'37.3", E27°19'35.8"; geodeticDatum: WGS84; **Event:** eventDate: 09.05-08.06.2009; habitat: oak forest
- **Type status:** Other material. **Occurrence:** recordedBy: R. Kostova; individualCount: 1; **Location:** countryCode: BG; locality: Kondolovo Vill., "Byalata prast" Place; verbatimElevation: 271; verbatimCoordinates: N42°05'47.7", E27°39'55.0"; geodeticDatum: WGS84; **Event:** eventDate: 03.08-07.09.2009; habitat: oak-beech mixed forest
- **Type status:** Other material. **Occurrence:** recordedBy: R. Kostova; individualCount: 1; **Location:** countryCode: BG; locality: Kondolovo Vill., "Byalata prast" Place; verbatimElevation: 283; verbatimCoordinates: N42°05'49.7", E27°39'56.3"; geodeticDatum: WGS84; **Event:** eventDate: 03.08-07.09.2009; habitat: beech forest with Vaccinium arctostaphylos
- **Type status:** Other material. **Occurrence:** recordedBy: R. Kostova; individualCount: 1; **Location:** countryCode: BG; locality: Brodilovo Vill., under Papiya peak; verbatimElevation: 336; verbatimCoordinates: N42°06'23.7", E27°50'36.1"; geodeticDatum: WGS84; **Event:** eventDate: 15.04-08.05.2009; habitat: oak forest
- **Type status:** Other material. **Occurrence:** recordedBy: R. Kostova; individualCount: 1; **Location:** countryCode: BG; locality: Brodilovo Vill., under Papiya peak; verbatimElevation: 296; verbatimCoordinates: N42°06'21.7", E27°50'32.6"; geodeticDatum: WGS84; **Event:** eventDate: 03.07-02.08.2009; habitat: shrubs, Phyllyrea latifolia
- **Type status:** Other material. **Occurrence:** recordedBy: R. Kostova; individualCount: 1; **Location:** countryCode: BG; locality: Sinemorets Vill., PA"Silistar", "Butamyata" Place; verbatimElevation: 17; verbatimCoordinates: N42°03'10.3", E27°59'13.4"; geodeticDatum: WGS84; **Event:** eventDate: 03.07-02.08.2009; habitat: oak forest
- **Type status:** Other material. **Location:** countryCode: BG; locality: Ropotamo; **Record Level:** bibliographicCitation: Hieke & Wrase (1988: 150)

#### Licinus (Licinus) depressus

(Paykull, 1790)

##### Materials

- **Type status:** Other material. **Occurrence:** recordedBy: R. Kostova; individualCount: 2; **Location:** countryCode: BG; locality: Kondolovo Vill., "Marsevski dol" Place; verbatimElevation: 284; verbatimCoordinates: N42°06'02.7", E27°40'53.5"; geodeticDatum: WGS84; **Event:** eventDate: 18/04/2009
- **Type status:** Other material. **Occurrence:** recordedBy: R. Kostova; individualCount: 2; **Location:** countryCode: BG; locality: Kondolovo Vill., "Byalata prast" Place; verbatimElevation: 271; verbatimCoordinates: N42°05'47.7", E27°39'55.0"; geodeticDatum: WGS84; **Event:** eventDate: 01/08/2009
- **Type status:** Other material. **Occurrence:** recordedBy: B. Guéorguiev; individualCount: 1; **Location:** countryCode: BG; locality: Aydere River; verbatimElevation: 350-450; **Event:** eventDate: 26/05/1995; **Record Level:** institutionCode: NMNHS

#### Odacantha (Odacantha) melanura

(Linnaeus, 1767)

##### Materials

- **Type status:** Other material. **Occurrence:** recordedBy: B. Petrov & I. Tasseva; individualCount: 1; **Location:** countryCode: BG; locality: Beech of residency "Perla", North of Primorsko Town; **Event:** eventDate: 18/04/1998; habitat: under stones on the sand; **Record Level:** institutionCode: NMNHS

#### Oodes helopioides helopioides

(Fabricius, 1792)

##### Materials

- **Type status:** Other material. **Occurrence:** recordedBy: R. Kostova; individualCount: 3; **Location:** countryCode: BG; locality: Sinemorets Vill., PA "Estuary of Veleka river"; verbatimElevation: 6; verbatimCoordinates: N42°03'39.6", E27°57'55.2"; geodeticDatum: WGS84; **Event:** eventDate: 16/04/2009
- **Type status:** Other material. **Occurrence:** recordedBy: R. Kostova; individualCount: 37; **Location:** countryCode: BG; locality: Sinemorets Vill., PA "Estuary of Veleka river"; verbatimElevation: 6; verbatimCoordinates: N42°03'39.6", E27°57'55.2"; geodeticDatum: WGS84; **Event:** eventDate: 15.04-02.07.2009; habitat: swamp forest
- **Type status:** Other material. **Occurrence:** recordedBy: R. Kostova; individualCount: 1; **Location:** countryCode: BG; locality: Malko Tarnovo, "Indipasqua" Place; verbatimElevation: 211; verbatimCoordinates: N42°00'16.9", E27°39'09.2"; geodeticDatum: WGS84; **Event:** eventDate: 18/04/2009

#### Panagaeus cruxmajor

(Linnaeus, 1758)

##### Materials

- **Type status:** Other material. **Occurrence:** recordedBy: R. Kostova; individualCount: 1; **Location:** countryCode: BG; locality: Sinemorets Vill., PA "Estuary of Veleka river"; verbatimElevation: 6; verbatimCoordinates: N42°03'39.6", E27°57'55.2"; geodeticDatum: WGS84; **Event:** eventDate: 09.06-02.07.2009; habitat: swamp forest
- **Type status:** Other material. **Occurrence:** recordedBy: W. Beier; individualCount: 1; **Location:** countryCode: BG; locality: Mouth of Veleka River near Sinemorets Vill.; verbatimElevation: 3; verbatimCoordinates: N42°03'51.0", E27°58'14.0"; geodeticDatum: WGS84; **Event:** eventDate: 27/04/2001; **Record Level:** collectionCode: cWB
- **Type status:** Other material. **Occurrence:** recordedBy: P. Beron; individualCount: 2; **Location:** countryCode: BG; locality: Kiten; **Event:** eventDate: 16-22.12.1984; **Record Level:** institutionCode: NMNHS

#### Agonum (Agonum) gisellae

Csiki, 1931

##### Materials

- **Type status:** Other material. **Occurrence:** recordedBy: R. Bekchiev; individualCount: 1; **Location:** countryCode: BG; locality: Kirovo Vill., along Selska River; verbatimElevation: 138; verbatimCoordinates: N42°10'43.0", E27°10'58.2"; geodeticDatum: WGS84; **Event:** eventDate: 05/05/2009
- **Type status:** Other material. **Location:** countryCode: BG; locality: Tsarevo (= Micurin); **Record Level:** bibliographicCitation: Hieke & Wrase (1988: 86, as A. angustatum)
- **Type status:** Other material. **Location:** countryCode: BG; locality: Mouth of Ropotamo River; **Record Level:** bibliographicCitation: Hieke & Wrase (1988: 86, as A. angustatum)

#### Agonum (Agonum) marginatum

(Linnaeus, 1758)

##### Materials

- **Type status:** Other material. **Occurrence:** recordedBy: R. Kostova; individualCount: 1; **Location:** countryCode: TR; locality: Kiyiköy surroundings; verbatimElevation: 32; verbatimCoordinates: N41°38'12.8", E28°04'08.7"; geodeticDatum: WGS84; **Event:** eventDate: 22/05/2011
- **Type status:** Other material. **Occurrence:** recordedBy: B. Guéorguiev; individualCount: 1; **Location:** countryCode: BG; locality: mouth of Veleka River near Sinemorets Vill.; **Event:** eventDate: 28/05/1995; **Record Level:** institutionCode: NMNHS
- **Type status:** Other material. **Occurrence:** recordedBy: W. Beier; individualCount: 1; **Location:** countryCode: BG; locality: Mouth of Veleka River near Sinemorets Vill.; verbatimElevation: 3; verbatimCoordinates: N42°03'51.0", E27°58'14.0"; geodeticDatum: WGS84; **Event:** eventDate: 27/04/2001; **Record Level:** collectionCode: cWB

#### Agonum (Agonum) muelleri

(Herbst, 1784)

##### Materials

- **Type status:** Other material. **Occurrence:** recordedBy: R. Kostova; individualCount: 4; **Location:** countryCode: BG; locality: Kosti Vill., "St. Ilia" Place; verbatimElevation: 35; verbatimCoordinates: N42°03'23.2", E27°45'51.6"; geodeticDatum: WGS84; **Event:** eventDate: 15.04-02.08.2009; habitat: meadow with single trees

#### Agonum (Agonum) sordidum sordidum

Dejean, 1828

##### Materials

- **Type status:** Other material. **Occurrence:** recordedBy: R. Bekchiev; individualCount: 1; **Location:** countryCode: TR; locality: Demirköy, Dökümhanesi; verbatimElevation: 179; verbatimCoordinates: N41°49'09.6", E27°48'43.2"; geodeticDatum: WGS84; **Event:** eventDate: 30/09/2009
- **Type status:** Other material. **Occurrence:** recordedBy: J. Mařan & K. Taborsky; individualCount: 1; **Location:** countryCode: BG; locality: Maslen nos Cape; **Event:** eventDate: VI.1933; **Record Level:** institutionCode: NMP
- **Type status:** Other material. **Location:** countryCode: BG; locality: Tsarevo (= Micurin); **Record Level:** bibliographicCitation: Hieke & Wrase (1988: 85)

#### Agonum (Europhilus) thoreyi thoreyi

Dejean, 1828

##### Materials

- **Type status:** Other material. **Occurrence:** recordedBy: R. Bekchiev; individualCount: 1; **Location:** countryCode: BG; locality: Primorsko, "Arcutino" Place; verbatimElevation: 60; verbatimCoordinates: N42°20'16.0", E27°43'18.1"; geodeticDatum: WGS84; **Event:** eventDate: 30/06/2009

#### Agonum (Olisares) longicorne

Chaudoir, 1846

##### Materials

- **Type status:** Other material. **Occurrence:** recordedBy: R. Kostova; individualCount: 1; **Location:** countryCode: BG; locality: Sinemorets Vill., PA "Estuary of Veleka river"; verbatimElevation: 6; verbatimCoordinates: N42°03'39.6", E27°57'55.2"; geodeticDatum: WGS84; **Event:** eventDate: 16/04/2009; habitat: swamp forest
- **Type status:** Other material. **Occurrence:** recordedBy: R. Kostova; individualCount: 4; **Location:** countryCode: BG; locality: Malko Tarnovo, "Indipasqua" Place; verbatimElevation: 211; verbatimCoordinates: N42°00'16.9", E27°39'09.2"; geodeticDatum: WGS84; **Event:** eventDate: 17/04/2009
- **Type status:** Other material. **Occurrence:** recordedBy: R. Kostova; individualCount: 1; **Location:** countryCode: BG; locality: Primorsko, Perla Beach; verbatimElevation: 1; verbatimCoordinates: N42°17'10.2", E27°45'13.6"; geodeticDatum: WGS84; **Event:** eventDate: 30/06/2009
- **Type status:** Other material. **Occurrence:** recordedBy: R. Bekchiev; individualCount: 1; **Location:** countryCode: BG; locality: Sinemorets Vill., PA "Estuary of Veleka river"; verbatimElevation: 5; verbatimCoordinates: N42°03'39.6", E27°57'55.2"; geodeticDatum: WGS84; **Event:** eventDate: 02/07/2009; habitat: meadow
- **Type status:** Other material. **Occurrence:** recordedBy: R. Kostova; individualCount: 5; **Location:** countryCode: BG; locality: Sinemorets Vill., PA "Estuary of Veleka river"; verbatimElevation: 6; verbatimCoordinates: N42°03'39.6", E27°57'55.2"; geodeticDatum: WGS84; **Event:** eventDate: 09.05-08.06.2009; habitat: swamp forest
- **Type status:** Other material. **Occurrence:** recordedBy: R. Kostova; individualCount: 4; **Location:** countryCode: BG; locality: Sinemorets Vill., PA "Estuary of Veleka river"; verbatimElevation: 6; verbatimCoordinates: N42°03'39.6", E27°57'55.2"; geodeticDatum: WGS84; **Event:** eventDate: 03.08-09.09.2009; habitat: swamp forest
- **Type status:** Other material. **Occurrence:** recordedBy: R. Kostova; individualCount: 2; **Location:** countryCode: TR; locality: Igneada; verbatimElevation: 15; verbatimCoordinates: N41°51'51.0", E27°56'54.1"; geodeticDatum: WGS84; **Event:** eventDate: 06/07/2009; habitat: Quercus forest
- **Type status:** Other material. **Occurrence:** recordedBy: R. Kostova; individualCount: 1; **Location:** countryCode: TR; locality: Igneada, Hamam Gölü; verbatimElevation: 5; verbatimCoordinates: N41°49'43.2", E27°57'31.1"; geodeticDatum: WGS84; **Event:** eventDate: 02/10/2009

#### Agonum (Olisares) lugens

(Duftcshmid, 1812)

##### Materials

- **Type status:** Other material. **Occurrence:** recordedBy: R. Bekchiev; individualCount: 1; **Location:** countryCode: BG; locality: Primorsko, Perla Beach; verbatimElevation: 1; verbatimCoordinates: N42°17'10.2", E27°45'13.6"; geodeticDatum: WGS84; **Event:** eventDate: 30/06/2009
- **Type status:** Other material. **Occurrence:** recordedBy: R. Bekchiev; individualCount: 4; **Location:** countryCode: BG; locality: Primorsko, "Arcutino" Place; verbatimElevation: 60; verbatimCoordinates: N42°20'16.0", E27°43'18.1"; geodeticDatum: WGS84; **Event:** eventDate: 30/06/2009
- **Type status:** Other material. **Location:** countryCode: BG; locality: Primorsko; **Record Level:** bibliographicCitation: Hieke & Wrase (1988: 85)

#### Agonum (Olisares) sexpunctatum

(Linnaeus, 1758)

##### Materials

- **Type status:** Other material. **Location:** countryCode: BG; locality: Maslen nos Cape; **Record Level:** bibliographicCitation: Guéorguiev & Guéorguiev (1995: 142)

##### Notes

This record is probably based on mislabelled specimens.

#### Agonum (Olisares) viduum

(Panzer, 1796)

##### Materials

- **Type status:** Other material. **Occurrence:** recordedBy: R. Kostova; individualCount: 4; **Location:** countryCode: BG; locality: Kirovo Vill., along Selska River; verbatimElevation: 138; verbatimCoordinates: N42°10'43.0", E27°10'58.2"; geodeticDatum: WGS84; **Event:** eventDate: 05/05/2009
- **Type status:** Other material. **Occurrence:** recordedBy: R. Kostova; individualCount: 2; **Location:** countryCode: BG; locality: Sinemorets Vill., PA "Estuary of Veleka river"; verbatimElevation: 6; verbatimCoordinates: N42°03'39.6", E27°57'55.2"; geodeticDatum: WGS84; **Event:** eventDate: 30/05/2010; habitat: swamp forest
- **Type status:** Other material. **Occurrence:** recordedBy: R. Kostova; individualCount: 2; **Location:** countryCode: BG; locality: Sinemorets Vill., PA"Silistar", Estuary of Silistar River; verbatimElevation: 12; verbatimCoordinates: N42°01'26.8", E28°00'32.9"; geodeticDatum: WGS84; **Event:** eventDate: 27/05/2011
- **Type status:** Other material. **Occurrence:** recordedBy: R. Kostova; individualCount: 432; **Location:** countryCode: BG; locality: Sinemorets Vill., PA "Estuary of Veleka river"; verbatimElevation: 6; verbatimCoordinates: N42°03'39.6", E27°57'55.2"; geodeticDatum: WGS84; **Event:** eventDate: 15.04-07.09.2009; habitat: swamp forest
- **Type status:** Other material. **Occurrence:** recordedBy: P. Beron; individualCount: 16; **Location:** countryCode: BG; locality: Kiten; **Event:** eventDate: 16-22.12.1984; **Record Level:** institutionCode: NMNHS

#### Anchomenus (Anchomenus) dorsalis

(Pontoppidan, 1763)

##### Materials

- **Type status:** Other material. **Occurrence:** recordedBy: A. Gijonova; individualCount: 1; **Location:** countryCode: TR; locality: Kiyiköy surroundings; verbatimElevation: 62; verbatimCoordinates: N41°37'40.7", E28°04'05.0"; geodeticDatum: WGS84; **Event:** eventDate: 22/05/2011
- **Type status:** Other material. **Occurrence:** recordedBy: R. Kostova; individualCount: 1; **Location:** countryCode: TR; locality: Kiyiköy surroundings; verbatimElevation: 32; verbatimCoordinates: N41°38'12.8", E28°04'08.7"; geodeticDatum: WGS84; **Event:** eventDate: 22/05/2011
- **Type status:** Other material. **Occurrence:** recordedBy: R. Kostova; individualCount: 12; **Location:** countryCode: BG; locality: Slivarovo Vill., "Shafariitsa" Place; verbatimElevation: 224; verbatimCoordinates: N41°57'37.8", E27°39'34.8"; geodeticDatum: WGS84; **Event:** eventDate: 09.05-02.07.2009; habitat: black alder-oak forest
- **Type status:** Other material. **Occurrence:** recordedBy: R. Kostova; individualCount: 140; **Location:** countryCode: BG; locality: Kosti Vill., "St. Ilia" Place; verbatimElevation: 35; verbatimCoordinates: N42°03'23.2", E27°45'51.6"; geodeticDatum: WGS84; **Event:** eventDate: 15.04-02.08.2009; habitat: meadow with single trees
- **Type status:** Other material. **Occurrence:** recordedBy: R. Kostova; individualCount: 14; **Location:** countryCode: BG; locality: Sinemorets Vill., PA "Estuary of Veleka river"; verbatimElevation: 6; verbatimCoordinates: N42°03'39.6", E27°57'55.2"; geodeticDatum: WGS84; **Event:** eventDate: 09.05-07.09.2009; habitat: swamp forest
- **Type status:** Other material. **Location:** countryCode: BG; locality: Malko Tarnovo; **Record Level:** bibliographicCitation: Guéorguiev & Guéorguiev (1995: 139)
- **Type status:** Other material. **Location:** countryCode: BG; locality: Tsarevo (= Micurin); **Record Level:** bibliographicCitation: Hieke & Wrase (1988: 83, as Platynus dorsalis)
- **Type status:** Other material. **Location:** countryCode: BG; locality: Ropotamo; **Record Level:** bibliographicCitation: Hieke & Wrase (1988: 83, as Platynus dorsalis)
- **Type status:** Other material. **Occurrence:** recordedBy: P. Petkov; individualCount: 1; **Location:** countryCode: BG; locality: Malko Tarnovo; **Event:** eventDate: 03-05.05.1921; **Record Level:** institutionCode: NMNHS
- **Type status:** Other material. **Occurrence:** recordedBy: J. Hlisnikovský; individualCount: 2; **Location:** countryCode: BG; locality: Maslen nos Cape; **Event:** eventDate: VII.1934; **Record Level:** institutionCode: NMP
- **Type status:** Other material. **Occurrence:** recordedBy: B. Guéorguiev; individualCount: 1; **Location:** countryCode: BG; locality: West of Varovnik Vill.; **Event:** eventDate: 24-25.05.1995; habitat: oak forest, in night; **Record Level:** institutionCode: NMNHS
- **Type status:** Other material. **Occurrence:** recordedBy: B. Guéorguiev; individualCount: 1; **Location:** countryCode: BG; locality: Veleka River near Mladezko Village; **Event:** eventDate: 25/05/1995; **Record Level:** institutionCode: NMNHS
- **Type status:** Other material. **Occurrence:** recordedBy: B. Guéorguiev; individualCount: 1; **Location:** countryCode: BG; locality: Tisovitsa Reserve near Bulgari Vill.; **Event:** eventDate: 27/05/1995; **Record Level:** institutionCode: NMNHS
- **Type status:** Other material. **Occurrence:** recordedBy: B. Guéorguiev; individualCount: 2; **Location:** countryCode: BG; locality: Nature Park Ropotamo; **Event:** eventDate: 29/05/1995; **Record Level:** institutionCode: NMNHS

#### Atranus ruficollis

(Gautier des Cottes, 1858)

##### Materials

- **Type status:** Other material. **Occurrence:** recordedBy: R. Bekchiev; individualCount: 1; **Location:** countryCode: BG; locality: Stoilovo Vill., "Sredoka" Reserve, along Mechi dol River; verbatimElevation: 206; verbatimCoordinates: N42°01'51.1", E27°30'50.1"; geodeticDatum: WGS84; **Event:** eventDate: 10/06/2009
- **Type status:** Other material. **Occurrence:** recordedBy: B. Guéorguiev; individualCount: 1; **Location:** countryCode: BG; locality: Rezovska River at Uzunbudzhak Reserve; **Event:** eventDate: 26/05/1995; **Record Level:** institutionCode: NMNHS

#### Limodromus assimilis

(Paykull, 1790)

##### Materials

- **Type status:** Other material. **Occurrence:** recordedBy: R. Kostova; individualCount: 1; **Location:** countryCode: BG; locality: Stoilovo Vill., "Sredoka" Reserve, along Mechi dol River; verbatimElevation: 206; verbatimCoordinates: N42°01'51.1", E27°30'50.1"; geodeticDatum: WGS84; **Event:** eventDate: 19/04/2009
- **Type status:** Other material. **Occurrence:** recordedBy: R. Bekchiev; individualCount: 3; **Location:** countryCode: TR; locality: Along Demirköy - Igneada road; verbatimElevation: 12; verbatimCoordinates: N41°52'39.3", E27°56'29.2"; geodeticDatum: WGS84; **Event:** eventDate: 05/07/2009
- **Type status:** Other material. **Occurrence:** recordedBy: R. Kostova; individualCount: 4; **Location:** countryCode: BG; locality: Stoilovo Vill., "Sredoka" Reserve, along Mechi dol River; verbatimElevation: 206; verbatimCoordinates: N42°01'51.1", E27°30'50.1"; geodeticDatum: WGS84; **Event:** eventDate: 27/05/2010
- **Type status:** Other material. **Occurrence:** recordedBy: R. Kostova; individualCount: 2; **Location:** countryCode: BG; locality: Malko Tarnovo surroundings; verbatimElevation: 375; verbatimCoordinates: N41°59'01.7", E27°29'49.4"; geodeticDatum: WGS84; **Event:** eventDate: 27/05/2010
- **Type status:** Other material. **Occurrence:** recordedBy: R. Kostova; individualCount: 3; **Location:** countryCode: BG; locality: Malko Tarnovo, "Propada" Place; verbatimElevation: 401; verbatimCoordinates: N41°58'49.6", E27°29'25.7"; geodeticDatum: WGS84; **Event:** eventDate: 09.06-02.07.2009; habitat: oriental beech forest
- **Type status:** Other material. **Occurrence:** recordedBy: R. Kostova; individualCount: 2; **Location:** countryCode: BG; locality: Slivarovo Vill., "Shafariitsa" Place; verbatimElevation: 224; verbatimCoordinates: N41°57'37.8", E27°39'34.8"; geodeticDatum: WGS84; **Event:** eventDate: 09.05-08.06.2009; habitat: black alder-oak forest
- **Type status:** Other material. **Occurrence:** recordedBy: R. Kostova; individualCount: 1; **Location:** countryCode: BG; locality: Slivarovo Vill., "Shafariitsa" Place; verbatimElevation: 224; verbatimCoordinates: N41°57'37.8", E27°39'34.8"; geodeticDatum: WGS84; **Event:** eventDate: 03.07-02.08.2009; habitat: black alder-oak forest
- **Type status:** Other material. **Occurrence:** recordedBy: R. Kostova; individualCount: 1; **Location:** countryCode: BG; locality: Kosti Vill., "St. Ilia" Place; verbatimElevation: 35; verbatimCoordinates: N42°03'23.2", E27°45'51.6"; geodeticDatum: WGS84; **Event:** eventDate: 15.04-08.05.2009; habitat: meadow with single trees
- **Type status:** Other material. **Occurrence:** recordedBy: R. Kostova; individualCount: 1; **Location:** countryCode: BG; locality: Kosti Vill., "St. Ilia" Place; verbatimElevation: 35; verbatimCoordinates: N42°03'23.2", E27°45'51.6"; geodeticDatum: WGS84; **Event:** eventDate: 03.07-02.08.2009; habitat: meadow with single trees
- **Type status:** Other material. **Occurrence:** recordedBy: R. Kostova; individualCount: 27; **Location:** countryCode: BG; locality: Sinemorets Vill., PA "Estuary of Veleka river"; verbatimElevation: 6; verbatimCoordinates: N42°03'39.6", E27°57'55.2"; geodeticDatum: WGS84; **Event:** eventDate: 15.04-07.09.2009; habitat: swamp forest
- **Type status:** Other material. **Occurrence:** recordedBy: R. Kostova; individualCount: 7; **Location:** countryCode: TR; locality: Igneada surroundings; verbatimElevation: 15; verbatimCoordinates: N41°51'51.0", E27°56'54.1"; geodeticDatum: WGS84; **Event:** eventDate: 06.07-29.09.2009; habitat: swamp forest
- **Type status:** Other material. **Occurrence:** recordedBy: K. Taborsky; individualCount: 1; **Location:** countryCode: BG; locality: Maslen nos Cape; **Event:** eventDate: VII.1934; **Record Level:** institutionCode: NMP
- **Type status:** Other material. **Occurrence:** recordedBy: B. Guéorguiev; individualCount: 6; **Location:** countryCode: BG; locality: Veleka River near Mladezko Village; **Event:** eventDate: 25/05/1995; **Record Level:** institutionCode: NMNHS
- **Type status:** Other material. **Occurrence:** recordedBy: B. Guéorguiev; individualCount: 6; **Location:** countryCode: BG; locality: Aydere River; verbatimElevation: 350-450; **Event:** eventDate: 26/05/1995; **Record Level:** institutionCode: NMNHS
- **Type status:** Other material. **Occurrence:** recordedBy: B. Guéorguiev; individualCount: 2; **Location:** countryCode: BG; locality: Nature Park Ropotamo; **Event:** eventDate: 29/05/1995; **Record Level:** institutionCode: NMNHS

#### Olisthopus fuscatus

Dejean, 1828

##### Materials

- **Type status:** Other material. **Location:** countryCode: BG; locality: Kiten; **Record Level:** bibliographicCitation: Hieke & Wrase (1988: 88)
- **Type status:** Other material. **Location:** countryCode: BG; locality: Kiten; **Record Level:** bibliographicCitation: Guéorguiev & Guéorguiev (1995: 139)

#### Olisthopus glabricollis

(Germar, 1817)

##### Materials

- **Type status:** Other material. **Occurrence:** recordedBy: R. Kostova; individualCount: 2; **Location:** countryCode: TR; locality: Avcilar Vill. Surroundings; verbatimElevation: 187; verbatimCoordinates: N41°53'44.6", E27°51'55.7"; geodeticDatum: WGS84; **Event:** eventDate: 23/05/2010
- **Type status:** Other material. **Location:** countryCode: BG; locality: Kiten; **Record Level:** bibliographicCitation: Hieke & Wrase (1988: 88)
- **Type status:** Other material. **Location:** countryCode: BG; locality: Ropotamo; **Record Level:** bibliographicCitation: Hieke & Wrase (1988: 88)

#### Oxypselaphus obscurus

(Herbst, 1784)

##### Materials

- **Type status:** Other material. **Occurrence:** recordedBy: A. Gijonova; individualCount: 1; **Location:** countryCode: TR; locality: Igneada; verbatimElevation: 5; verbatimCoordinates: N41°49'43.2", E27°57'31.1"; geodeticDatum: WGS84; **Event:** eventDate: 02/10/2009; habitat: Quercus forest
- **Type status:** Other material. **Occurrence:** recordedBy: R. Kostova; individualCount: 4; **Location:** countryCode: TR; locality: Igneada, Hamam Gölü; verbatimElevation: 11; verbatimCoordinates: N41°49'31.6", E27°57'33.8"; geodeticDatum: WGS84; **Event:** eventDate: 02/10/2009

#### Platynus proximus

(J. Frivaldszky, 1879)

##### Materials

- **Type status:** Other material. **Occurrence:** recordedBy: no collector; individualCount: 2; **Location:** countryCode: BG; locality: Strandzha; **Record Level:** institutionCode: NPM (it is a doubtful record!)

#### Abax (Abacopercus) carinatus carinatus

(Duftschmid, 1812)

##### Materials

- **Type status:** Other material. **Occurrence:** recordedBy: R. Kostova; individualCount: 1; **Location:** countryCode: BG; locality: Bliznak Vill., PA"Bataka"; verbatimElevation: 324; verbatimCoordinates: N42°11'37.3", E27°19'35.8"; geodeticDatum: WGS84; **Event:** eventDate: 15.04-08.05.2009; habitat: oak forest
- **Type status:** Other material. **Occurrence:** recordedBy: R. Kostova; individualCount: 1; **Location:** countryCode: BG; locality: Bliznak Vill., PA"Bataka"; verbatimElevation: 324; verbatimCoordinates: N42°11'37.3", E27°19'35.8"; geodeticDatum: WGS84; **Event:** eventDate: 03.08-07.09.2009; habitat: oak forest
- **Type status:** Other material. **Occurrence:** recordedBy: R. Kostova; individualCount: 5; **Location:** countryCode: TR; locality: Igneada surroundings; verbatimElevation: 15; verbatimCoordinates: N41°51'51.0", E27°56'54.1"; geodeticDatum: WGS84; **Event:** eventDate: 06.07-29.09.2009; habitat: swamp forest
- **Type status:** Other material. **Location:** countryCode: BG; locality: Ropotamo; **Record Level:** bibliographicCitation: Hieke & Wrase (1988: 79)
- **Type status:** Other material. **Location:** countryCode: TR; locality: Demirköy; verbatimElevation: 260; **Record Level:** bibliographicCitation: Guéorguiev (2011: 536)

#### Abax (Abax) ovalis

(Duftschmid, 1812)

##### Materials

- **Type status:** Other material. **Location:** countryCode: BG; locality: Maslen nos Cape; **Record Level:** bibliographicCitation: Guéorguiev & Guéorguiev (1995: 134)

##### Notes

This record is probably based on mislabelled specimens.

#### Molops (Molops) alpestris kalofericus

Mlynář, 1977

##### Materials

- **Type status:** Other material. **Occurrence:** recordedBy: K. Tuleshkov; individualCount: 2; **Location:** countryCode: BG; locality: Maslen nos Cape; **Event:** eventDate: 16/07/1933; **Record Level:** institutionCode: NMNHS

##### Notes

This record is probably based on mislabelled specimens.

#### Molops (Molops) dilatatus angulicollis

J. Müller, 1936

##### Materials

- **Type status:** Other material. **Location:** countryCode: BG; locality: Strandzha; **Record Level:** bibliographicCitation: Mlynář (1977: 70)
- **Type status:** Other material. **Occurrence:** recordedBy: K. Tuleshkov; individualCount: 2; **Location:** countryCode: BG; locality: Maslen nos Cape; **Event:** eventDate: 16/07/1933; **Record Level:** institutionCode: NMNHS

#### Molops (Molops) piceus byzantinus

Apfelbeck, 1902

##### Materials

- **Type status:** Other material. **Occurrence:** recordedBy: R. Kostova; individualCount: 1; **Location:** countryCode: TR; locality: Sivriler Vill. suroundings; verbatimElevation: 379; verbatimCoordinates: N41°47'30.2", E27°50'00.9"; geodeticDatum: WGS84; **Event:** eventDate: 24/05/2011
- **Type status:** Other material. **Occurrence:** recordedBy: R. Kostova; individualCount: 27; **Location:** countryCode: BG; locality: Malko Tarnovo, "Propada" Place; verbatimElevation: 401; verbatimCoordinates: N41°58'49.6", E27°29'25.7"; geodeticDatum: WGS84; **Event:** eventDate: 15.04-02.07.2009; habitat: oriental beech forest
- **Type status:** Other material. **Occurrence:** recordedBy: R. Kostova; individualCount: 6; **Location:** countryCode: BG; locality: Slivarovo Vill., "Shafariitsa" Place; verbatimElevation: 224; verbatimCoordinates: N41°57'37.8", E27°39'34.8"; geodeticDatum: WGS84; **Event:** eventDate: 15.04-08.06.2009; habitat: black alder-oak forest
- **Type status:** Other material. **Occurrence:** recordedBy: R. Kostova; individualCount: 1; **Location:** countryCode: BG; locality: Bulgari Vill., PA "Marina reka"; verbatimElevation: 183; verbatimCoordinates: N42°06'41.7", E27°45'53.0"; geodeticDatum: WGS84; **Event:** eventDate: 09.05-08.06.2009; habitat: oriental beech-oak forest with Rododendron ponticum
- **Type status:** Other material. **Occurrence:** recordedBy: R. Kostova; individualCount: 1; **Location:** countryCode: BG; locality: Brodilovo Vill., under Papiya peak; verbatimElevation: 336; verbatimCoordinates: N42°06'23.7", E27°50'36.1"; geodeticDatum: WGS84; **Event:** eventDate: 09.05-08.06.2009; habitat: oak forest
- **Type status:** Other material. **Occurrence:** recordedBy: R. Kostova; individualCount: 1; **Location:** countryCode: BG; locality: Brodilovo Vill., under Papiya peak; verbatimElevation: 296; verbatimCoordinates: N42°06'21.7", E27°50'32.6"; geodeticDatum: WGS84; **Event:** eventDate: 15.04-08.05.2009; habitat: shrubs, Phyllyrea latifolia
- **Type status:** Other material. **Occurrence:** recordedBy: R. Kostova; individualCount: 2; **Location:** countryCode: BG; locality: Sinemorets Vill., PA"Silistar", "Butamyata" Place; verbatimElevation: 17; verbatimCoordinates: N42°03'10.3", E27°59'13.4"; geodeticDatum: WGS84; **Event:** eventDate: 15.04-08.06.2009; habitat: oak forest
- **Type status:** Other material. **Occurrence:** recordedBy: R. Kostova; individualCount: 1; **Location:** countryCode: TR; locality: Kiyiköy surroundings; verbatimElevation: 38; verbatimCoordinates: N41°39'25.7", E28°05'11.2"; geodeticDatum: WGS84; **Event:** eventDate: 06.07 - 29.09.2009; habitat: oak with Arbutus unedo
- **Type status:** Other material. **Location:** countryCode: BG; locality: Malko Tarnovo; **Record Level:** bibliographicCitation: Mlynář (1977: 142)
- **Type status:** Other material. **Location:** countryCode: BG; locality: Strandzha; **Record Level:** bibliographicCitation: Guéorguiev & Guéorguiev (1995: 138)
- **Type status:** Other material. **Occurrence:** recordedBy: P. Petkov; individualCount: 2; **Location:** countryCode: BG; locality: Kosti Vill.; **Event:** eventDate: 29-30.04.1921; **Record Level:** institutionCode: NMNHS
- **Type status:** Other material. **Occurrence:** recordedBy: K. Tuleshkov; individualCount: 3; **Location:** countryCode: BG; locality: Maslen nos Cape; **Event:** eventDate: 16/07/1933; **Record Level:** institutionCode: NMNHS
- **Type status:** Other material. **Occurrence:** recordedBy: S. Andreev; individualCount: 1; **Location:** countryCode: BG; locality: Aydere River; **Event:** eventDate: 30/05/1982; habitat: moist leaf litter near brook; **Record Level:** institutionCode: NMNHS
- **Type status:** Other material. **Occurrence:** recordedBy: B. Guéorguiev; individualCount: 4; **Location:** countryCode: BG; locality: Aydere River; verbatimElevation: 350-450; **Event:** eventDate: 26/05/1995; **Record Level:** institutionCode: NMNHS

#### Myas (Myas) chalybaeus

(Palliardi, 1825)

##### Materials

- **Type status:** Other material. **Occurrence:** recordedBy: R. Kostova; individualCount: 1; **Location:** countryCode: BG; locality: Kirovo Vill., along Selska River; verbatimElevation: 138; verbatimCoordinates: N42°10'43.0", E27°10'58.2"; geodeticDatum: WGS84; **Event:** eventDate: 05/05/2009
- **Type status:** Other material. **Occurrence:** recordedBy: R. Kostova; individualCount: 9; **Location:** countryCode: BG; locality: Bliznak Vill., PA"Bataka"; verbatimElevation: 324; verbatimCoordinates: N42°11'37.3", E27°19'35.8"; geodeticDatum: WGS84; **Event:** eventDate: 09.05-02.07.2009; habitat: oak forest
- **Type status:** Other material. **Occurrence:** recordedBy: R. Kostova; individualCount: 2; **Location:** countryCode: BG; locality: Bliznak Vill., PA"Bataka"; verbatimElevation: 324; verbatimCoordinates: N42°11'37.3", E27°19'35.8"; geodeticDatum: WGS84; **Event:** eventDate: 03.08-07.09.2009; habitat: oak forest
- **Type status:** Other material. **Occurrence:** recordedBy: R. Kostova; individualCount: 10; **Location:** countryCode: BG; locality: Malko Tarnovo, "Propada" Place; verbatimElevation: 401; verbatimCoordinates: N41°58'49.6", E27°29'25.7"; geodeticDatum: WGS84; **Event:** eventDate: 09.05-07.09.2009; habitat: oriental beech forest
- **Type status:** Other material. **Occurrence:** recordedBy: R. Kostova; individualCount: 1; **Location:** countryCode: BG; locality: Slivarovo Vll. surroundings; verbatimElevation: 331; verbatimCoordinates: N41°58'11.9", E27°39'31.1"; geodeticDatum: WGS84; **Event:** eventDate: 09.05-08.06.2009; habitat: shrubs, mainly Calluna vulgaris
- **Type status:** Other material. **Occurrence:** recordedBy: R. Kostova; individualCount: 8; **Location:** countryCode: BG; locality: Kondolovo Vill., "Byalata prast" Place; verbatimElevation: 271; verbatimCoordinates: N42°05'47.7", E27°39'55.0"; geodeticDatum: WGS84; **Event:** eventDate: 15.04-08.06.2009; habitat: oak-beech mixed forest
- **Type status:** Other material. **Occurrence:** recordedBy: R. Kostova; individualCount: 5; **Location:** countryCode: BG; locality: Kondolovo Vill., "Byalata prast" Place; verbatimElevation: 283; verbatimCoordinates: N42°05'49.7", E27°39'56.3"; geodeticDatum: WGS84; **Event:** eventDate: 09.05-02.07.2009; habitat: beech forest with Vaccinium arctostaphylos
- **Type status:** Other material. **Occurrence:** recordedBy: R. Kostova; individualCount: 1; **Location:** countryCode: BG; locality: Kondolovo Vill., "Byalata prast" Place; verbatimElevation: 283; verbatimCoordinates: N42°05'49.7", E27°39'56.3"; geodeticDatum: WGS84; **Event:** eventDate: 03.08-07.09.2009; habitat: beech forest with Vaccinium arctostaphylos
- **Type status:** Other material. **Occurrence:** recordedBy: R. Kostova; individualCount: 18; **Location:** countryCode: BG; locality: Bulgari Vill., PA "Marina reka"; verbatimElevation: 183; verbatimCoordinates: N42°06'41.7", E27°45'53.0"; geodeticDatum: WGS84; **Event:** eventDate: 09.05-07.09.2009; habitat: oriental beech-oak forest with Rododendron ponticum
- **Type status:** Other material. **Occurrence:** recordedBy: R. Kostova; individualCount: 2; **Location:** countryCode: BG; locality: Brodilovo Vill., under Papiya peak; verbatimElevation: 336; verbatimCoordinates: N42°06'23.7", E27°50'36.1"; geodeticDatum: WGS84; **Event:** eventDate: 09.05-08.06.2009; habitat: oak forest
- **Type status:** Other material. **Occurrence:** recordedBy: R. Kostova; individualCount: 5; **Location:** countryCode: BG; locality: Sinemorets Vill., PA"Silistar", "Butamyata" Place; verbatimElevation: 17; verbatimCoordinates: N42°03'10.3", E27°59'13.4"; geodeticDatum: WGS84; **Event:** eventDate: 09.05-08.06.2009; habitat: oak forest
- **Type status:** Other material. **Occurrence:** recordedBy: R. Kostova; individualCount: 1; **Location:** countryCode: BG; locality: Mladezhko Vill., the springs of Mladezhka River; verbatimElevation: 231; verbatimCoordinates: N42°09'04.5", E27°21'26.1"; geodeticDatum: WGS84; **Event:** eventDate: 09.05-08.06.2009; habitat: black alder and hornbeam forest
- **Type status:** Other material. **Occurrence:** recordedBy: R. Kostova; individualCount: 2; **Location:** countryCode: TR; locality: Gokyaka Vill., Road to Dupnisa Cave; verbatimElevation: 337; verbatimCoordinates: N41°52'20.0", E27°37'06.4"; geodeticDatum: WGS84; **Event:** eventDate: 06.07 - 29.09.2009; habitat: oak-hornbeam mixed forest
- **Type status:** Other material. **Location:** countryCode: BG; locality: Velika Papiya; **Record Level:** bibliographicCitation: Guéorguiev & Guéorguiev (1995: 119)
- **Type status:** Other material. **Location:** countryCode: BG; locality: Valchi most; **Record Level:** bibliographicCitation: Guéorguiev & Guéorguiev (1995: 119)

#### Pedius inquinatus

(Sturm, 1824)

##### Materials

- **Type status:** Other material. **Occurrence:** recordedBy: P. Beron; individualCount: 1; **Location:** countryCode: BG; locality: Kiten; **Event:** eventDate: 16-22.12.1984; **Record Level:** institutionCode: NMNHS

#### Poecilus (Poecilus) anatolicus

(Chaudoir, 1850)

##### Materials

- **Type status:** Other material. **Occurrence:** recordedBy: P. Drenski; individualCount: 1; **Location:** countryCode: BG; locality: Izgrev Vill.; **Event:** eventDate: 17/05/1931; **Record Level:** institutionCode: NMNHS

#### Poecilus (Poecilus) cupreus cupreus

(Linnaeus, 1758)

##### Materials

- **Type status:** Other material. **Occurrence:** recordedBy: R. Kostova; individualCount: 1; **Location:** countryCode: BG; locality: Sinemorets Vill., PA "Estuary of Veleka river"; verbatimElevation: 6; verbatimCoordinates: N42°03'39.6", E27°57'55.2"; geodeticDatum: WGS84; **Event:** eventDate: 16/04/2009; habitat: swamp forest
- **Type status:** Other material. **Occurrence:** recordedBy: R. Kostova; individualCount: 39; **Location:** countryCode: BG; locality: Kosti Vill., "St. Ilia" Place; verbatimElevation: 35; verbatimCoordinates: N42°03'23.2", E27°45'51.6"; geodeticDatum: WGS84; **Event:** eventDate: 15.04-07.09.2009; habitat: meadow with single trees
- **Type status:** Other material. **Occurrence:** recordedBy: R. Kostova; individualCount: 54; **Location:** countryCode: BG; locality: Sinemorets Vill., PA "Estuary of Veleka river"; verbatimElevation: 6; verbatimCoordinates: N42°03'39.6", E27°57'55.2"; geodeticDatum: WGS84; **Event:** eventDate: 09.05-07.09.2009; habitat: swamp forest
- **Type status:** Other material. **Location:** countryCode: BG; locality: Kiten; **Record Level:** bibliographicCitation: Hieke & Wrase (1988: 70)
- **Type status:** Other material. **Location:** countryCode: BG; locality: Tsarevo (= Micurin); **Record Level:** bibliographicCitation: Hieke & Wrase (1988: 70)
- **Type status:** Other material. **Location:** countryCode: BG; locality: Ropotamo; **Record Level:** bibliographicCitation: Hieke & Wrase (1988: 70)
- **Type status:** Other material. **Location:** countryCode: BG; locality: Izgrev Vill.; **Record Level:** bibliographicCitation: Guéorguiev & Guéorguiev (1995: 121)

#### Poecilus (Poecilus) cursorius cursorius

(Dejean, 1828)

##### Materials

- **Type status:** Other material. **Occurrence:** recordedBy: W. Beier; individualCount: 1; **Location:** countryCode: BG; locality: Mouth of Veleka River near Sinemorets Vill.; verbatimElevation: 3; verbatimCoordinates: N42°03'51.0", E27°58'14.0"; geodeticDatum: WGS84; **Event:** eventDate: 27/04/2001; **Record Level:** collectionCode: cWB
- **Type status:** Other material. **Location:** countryCode: BG; locality: Ropotamo; **Record Level:** bibliographicCitation: Hieke & Wrase (1988: 71)

#### Poecilus (Poecilus) lepidus lepidus

(Leske, 1785)

##### Materials

- **Type status:** Other material. **Location:** countryCode: BG; locality: Strandzha; **Record Level:** bibliographicCitation: Guéorguiev & Guéorguiev (1995: 120)
- **Type status:** Other material. **Occurrence:** recordedBy: K. Tuleshkov; individualCount: 2; **Location:** countryCode: BG; locality: Maslen nos Cape; **Event:** eventDate: 16/07/1933

#### Poecilus (Poecilus) versicolor

(Sturm, 1824)

##### Materials

- **Type status:** Other material. **Occurrence:** recordedBy: R. Kostova; individualCount: 1; **Location:** countryCode: BG; locality: Kosti Vill., "St. Ilia" Place; verbatimElevation: 35; verbatimCoordinates: N42°03'23.2", E27°45'51.6"; geodeticDatum: WGS84; **Event:** eventDate: 19/04/2009; habitat: meadow with single trees

#### Pterostichus (Argutor) cursor

(Dejean, 1828)

##### Materials

- **Type status:** Other material. **Occurrence:** recordedBy: R. Kostova; individualCount: 1; **Location:** countryCode: BG; locality: Primorsko, Perla Beach; verbatimElevation: 1; verbatimCoordinates: N42°17'10.2", E27°45'13.6"; geodeticDatum: WGS84; **Event:** eventDate: 10/05/2009
- **Type status:** Other material. **Occurrence:** recordedBy: R. Bekchiev; individualCount: 1; **Location:** countryCode: TR; locality: Igneada surroundings; verbatimElevation: 3; verbatimCoordinates: N41°51'27.3", E27°57'28.7"; geodeticDatum: WGS84; **Event:** eventDate: 29/09/2009; habitat: marsh
- **Type status:** Other material. **Occurrence:** recordedBy: R. Kostova; individualCount: 1; **Location:** countryCode: TR; locality: Igneada, Hamam Gölü; verbatimElevation: 11; verbatimCoordinates: N41°49'31.6", E27°57'33.8"; geodeticDatum: WGS84; **Event:** eventDate: 02/10/2009
- **Type status:** Other material. **Location:** countryCode: BG; locality: Tsarevo (= Micurin); **Record Level:** bibliographicCitation: Hieke & Wrase (1988: 73)
- **Type status:** Other material. **Location:** countryCode: BG; locality: Mouth of Ropotamo River; **Record Level:** bibliographicCitation: Hieke & Wrase (1988: 73)

#### Pterostichus (Argutor) leonisi

Apfelbeck, 1904

##### Materials

- **Type status:** Other material. **Occurrence:** recordedBy: R. Bekchiev; individualCount: 1; **Location:** countryCode: BG; locality: Kirovo Vill., along Selska River; verbatimElevation: 138; verbatimCoordinates: N42°10'43.0", E27°10'58.2"; geodeticDatum: WGS84; **Event:** eventDate: 05/05/2009
- **Type status:** Other material. **Occurrence:** recordedBy: R. Kostova; individualCount: 30; **Location:** countryCode: BG; locality: Sinemorets Vill., PA "Estuary of Veleka river"; verbatimElevation: 6; verbatimCoordinates: N42°03'39.6", E27°57'55.2"; geodeticDatum: WGS84; **Event:** eventDate: 15.04-02.07.2009; habitat: swamp forest
- **Type status:** Other material. **Occurrence:** recordedBy: R. Kostova; individualCount: 1; **Location:** countryCode: TR; locality: Igneada, Hamam Gölü; verbatimElevation: 11; verbatimCoordinates: N41°49'31.6", E27°57'33.8"; geodeticDatum: WGS84; **Event:** eventDate: 09/10/2009
- **Type status:** Other material. **Location:** countryCode: BG; locality: Tsarevo (= Micurin); **Record Level:** bibliographicCitation: Hieke & Wrase (1988: 73)
- **Type status:** Other material. **Location:** countryCode: BG; locality: Primorsko; **Record Level:** bibliographicCitation: Hieke & Wrase (1988: 73)
- **Type status:** Other material. **Occurrence:** recordedBy: P. Beron; individualCount: 1; **Location:** countryCode: BG; locality: Kiten; **Event:** eventDate: 16-22.12.1984; **Record Level:** institutionCode: NMNHS

#### Pterostichus (Bothriopterus) quadrifoveolatus

Letzner, 1852

##### Materials

- **Type status:** Other material. **Location:** countryCode: BG; locality: Primorsko, bay of Stamopolu; **Record Level:** bibliographicCitation: Wrase (1991: 8)
- **Type status:** Other material. **Location:** countryCode: BG; locality: Primorsko, bay of Stamopolu; **Record Level:** bibliographicCitation: Guéorguiev & Guéorguiev (1995: 125)

#### Pterostichus (Cryobius) properans

(Chaudoir, 1868)

##### Materials

- **Type status:** Other material. **Occurrence:** recordedBy: R. Kostova; individualCount: 1; **Location:** countryCode: TR; locality: Kiyiköy surroundings; verbatimElevation: 38; verbatimCoordinates: N41°39'25.7", E28°05'11.2"; geodeticDatum: WGS84; **Event:** eventDate: 30/05/2009
- **Type status:** Other material. **Occurrence:** recordedBy: R. Kostova; individualCount: 2; **Location:** countryCode: TR; locality: Igneada, Hamam Gölü; verbatimElevation: 5; verbatimCoordinates: N41°49'43.2", E27°57'31.1"; geodeticDatum: WGS84; **Event:** eventDate: 02/10/2009
- **Type status:** Other material. **Occurrence:** recordedBy: R. Kostova; individualCount: 2; **Location:** countryCode: TR; locality: Sislioba Vill. surroundings; verbatimElevation: 49; verbatimCoordinates: N41°57'44.2", E27°54'36.1"; geodeticDatum: WGS84; **Event:** eventDate: 02/10/2009
- **Type status:** Other material. **Occurrence:** recordedBy: R. Kostova; individualCount: 1; **Location:** countryCode: TR; locality: Demirköy, Dökümhanesi; verbatimElevation: 179; verbatimCoordinates: N41°49'09.6", E27°48'43.2"; geodeticDatum: WGS84; **Event:** eventDate: 03/10/2009
- **Type status:** Other material. **Occurrence:** recordedBy: R. Kostova; individualCount: 4; **Location:** countryCode: BG; locality: Bliznak Vill., PA"Bataka"; verbatimElevation: 324; verbatimCoordinates: N42°11'37.3", E27°19'35.8"; geodeticDatum: WGS84; **Event:** eventDate: 03.08-07.09.2009; habitat: oak forest
- **Type status:** Other material. **Occurrence:** recordedBy: R. Kostova; individualCount: 1; **Location:** countryCode: BG; locality: Malko Tarnovo, "Propada" Place; verbatimElevation: 401; verbatimCoordinates: N41°58'49.6", E27°29'25.7"; geodeticDatum: WGS84; **Event:** eventDate: 09.06-02.07.2009; habitat: oriental beech forest
- **Type status:** Other material. **Occurrence:** recordedBy: R. Kostova; individualCount: 4; **Location:** countryCode: BG; locality: Malko Tarnovo, "Propada" Place; verbatimElevation: 401; verbatimCoordinates: N41°58'49.6", E27°29'25.7"; geodeticDatum: WGS84; **Event:** eventDate: 03.08-07.09.2009; habitat: oriental beech forest
- **Type status:** Other material. **Occurrence:** recordedBy: R. Kostova; individualCount: 2; **Location:** countryCode: BG; locality: Slivarovo Vill., "Shafariitsa" Place; verbatimElevation: 224; verbatimCoordinates: N41°57'37.8", E27°39'34.8"; geodeticDatum: WGS84; **Event:** eventDate: 03.08-07.09.2009; habitat: black alder-oak forest
- **Type status:** Other material. **Occurrence:** recordedBy: R. Kostova; individualCount: 4; **Location:** countryCode: BG; locality: Bulgari Vill., PA "Marina reka"; verbatimElevation: 183; verbatimCoordinates: N42°06'41.7", E27°45'53.0"; geodeticDatum: WGS84; **Event:** eventDate: 03.08-07.09.2009; habitat: oriental beech-oak forest with Rododendron ponticum
- **Type status:** Other material. **Occurrence:** recordedBy: R. Kostova; individualCount: 1; **Location:** countryCode: BG; locality: Brodilovo Vill., under Papiya peak; verbatimElevation: 336; verbatimCoordinates: N42°06'23.7", E27°50'36.1"; geodeticDatum: WGS84; **Event:** eventDate: 03.08-07.09.2009; habitat: oak forest
- **Type status:** Other material. **Occurrence:** recordedBy: R. Kostova; individualCount: 1; **Location:** countryCode: TR; locality: Gokyaka Vill., Road to Dupnisa Cave; verbatimElevation: 337; verbatimCoordinates: N41°52'20.0", E27°37'06.4"; geodeticDatum: WGS84; **Event:** eventDate: 06.07 - 29.09.2009; habitat: oak-hornbeam mixed forest
- **Type status:** Other material. **Occurrence:** recordedBy: A. Pfeffer; individualCount: 1; **Location:** countryCode: BG; locality: Strandzha; **Event:** eventDate: VII.1934; **Record Level:** institutionCode: NMP
- **Type status:** Other material. **Location:** countryCode: BG; locality: Strandzha; **Record Level:** bibliographicCitation: Mařan (1944: 101, as Haptoderus acrogonus properans)
- **Type status:** Other material. **Location:** countryCode: BG; locality: Strandzha; **Record Level:** bibliographicCitation: Guéorguiev & Guéorguiev (1995: 126)

#### Pterostichus (Feronidius) melas melas

(Creutzer, 1799)

##### Materials

- **Type status:** Other material. **Occurrence:** recordedBy: R. Kostova; individualCount: 1; **Location:** countryCode: TR; locality: Demirköy, to Mahya Dağı Peak; verbatimElevation: 488; verbatimCoordinates: N41°52'41.1", E27°54'27.0"; geodeticDatum: WGS84; **Event:** eventDate: 05/07/2009
- **Type status:** Other material. **Occurrence:** recordedBy: R. Kostova; individualCount: 1; **Location:** countryCode: TR; locality: Demirköy, along Degirmen River; verbatimElevation: 289; verbatimCoordinates: N41°49'17.2", E27°45'07.7"; geodeticDatum: WGS84; **Event:** eventDate: 06/07/2009
- **Type status:** Other material. **Occurrence:** recordedBy: R. Kostova; individualCount: 1; **Location:** countryCode: TR; locality: Igneada, Hamam Gölü; verbatimElevation: 11; verbatimCoordinates: N41°49'31.6", E27°57'33.8"; geodeticDatum: WGS84; **Event:** eventDate: 25/05/2010
- **Type status:** Other material. **Occurrence:** recordedBy: R. Kostova; individualCount: 1; **Location:** countryCode: BG; locality: Malko Tarnovo, "Propada" Place; verbatimElevation: 401; verbatimCoordinates: N41°58'49.6", E27°29'25.7"; geodeticDatum: WGS84; **Event:** eventDate: 03.08-07.09.2009; habitat: oriental beech forest
- **Type status:** Other material. **Occurrence:** recordedBy: R. Kostova; individualCount: 89; **Location:** countryCode: BG; locality: Kosti Vill., "St. Ilia" Place; verbatimElevation: 35; verbatimCoordinates: N42°03'23.2", E27°45'51.6"; geodeticDatum: WGS84; **Event:** eventDate: 09.05-07.09.2009; habitat: meadow with single trees
- **Type status:** Other material. **Occurrence:** recordedBy: R. Kostova; individualCount: 1; **Location:** countryCode: BG; locality: Sinemorets Vill., PA "Estuary of Veleka river"; verbatimElevation: 6; verbatimCoordinates: N42°03'39.6", E27°57'55.2"; geodeticDatum: WGS84; **Event:** eventDate: 03.07-02.08.2009
- **Type status:** Other material. **Occurrence:** recordedBy: R. Kostova; individualCount: 1; **Location:** countryCode: BG; locality: Mladezhko Vill., the springs of Mladezhka River; verbatimElevation: 231; verbatimCoordinates: N42°09'04.5", E27°21'26.1"; geodeticDatum: WGS84; **Event:** eventDate: 03.07-02.08.2009; habitat: black alder and hornbeam forest
- **Type status:** Other material. **Occurrence:** recordedBy: R. Kostova; individualCount: 39; **Location:** countryCode: TR; locality: Igneada surroundings; verbatimElevation: 15; verbatimCoordinates: N41°51'51.0", E27°56'54.1"; geodeticDatum: WGS84; **Event:** eventDate: 06.07-29.09.2009; habitat: swamp forest
- **Type status:** Other material. **Occurrence:** recordedBy: K. Taborsky; individualCount: 4; **Location:** countryCode: BG; locality: Maslen nos Cape; **Event:** eventDate: VII.1934; **Record Level:** institutionCode: NMP
- **Type status:** Other material. **Occurrence:** recordedBy: R. Radev; individualCount: 2; **Location:** countryCode: BG; locality: Sinemorets Vill.; **Event:** eventDate: 7.VIII.1989; **Record Level:** collectionID: R. Radev coll.
- **Type status:** Other material. **Location:** countryCode: BG; locality: Tsarevo (= Micurin); **Record Level:** bibliographicCitation: Hieke & Wrase (1988: 76, as P. melas depressus)

#### Pterostichus (Haptotapinus) crassiusculus

(Chaudoir, 1868) s.l.

##### Materials

- **Type status:** Other material. **Occurrence:** recordedBy: R. Kostova; individualCount: 1; **Location:** countryCode: BG; locality: Slivarovo Vill., "Shafariitsa" Place; verbatimElevation: 224; verbatimCoordinates: N41°57'37.8", E27°39'34.8"; geodeticDatum: WGS84; **Event:** eventDate: 09.05-08.06.2009; habitat: black alder-oak forest

#### Pterostichus (Melanius) elongatus

(Duftschmid, 1812)

##### Materials

- **Type status:** Other material. **Occurrence:** recordedBy: B. Guéorguiev; individualCount: 1; **Location:** countryCode: BG; locality: Nature Park Ropotamo; **Event:** eventDate: 29/05/1995; **Record Level:** institutionCode: NMNHS

#### Pterostichus (Parahaptoderus) vecors

(Tschitschérine, 1897)

##### Materials

- **Type status:** Other material. **Location:** countryCode: BG; locality: Strandzha; **Record Level:** bibliographicCitation: Guéorguiev (1992: 66)

#### Pterostichus (Petrophilus) melanarius melanarius

(Illiger, 1798)

##### Materials

- **Type status:** Other material. **Occurrence:** recordedBy: R. Kostova; individualCount: 177; **Location:** countryCode: BG; locality: Kosti Vill., "St. Ilia" Place; verbatimElevation: 35; verbatimCoordinates: N42°03'23.2", E27°45'51.6"; geodeticDatum: WGS84; **Event:** eventDate: 15.04-07.09.2009; habitat: meadow with single trees

#### Pterostichus (Phonias) strenuus

(Panzer, 1796)

##### Materials

- **Type status:** Other material. **Occurrence:** recordedBy: R. Kostova; individualCount: 1; **Location:** countryCode: BG; locality: Kirovo Vill., along Selska River; verbatimElevation: 138; verbatimCoordinates: N42°10'43.0", E27°10'58.2"; geodeticDatum: WGS84; **Event:** eventDate: 05/05/2009
- **Type status:** Other material. **Occurrence:** recordedBy: R. Kostova; individualCount: 1; **Location:** countryCode: BG; locality: Slivarovo Vill., "Shafariitsa" Place; verbatimElevation: 224; verbatimCoordinates: N41°57'37.8", E27°39'34.8"; geodeticDatum: WGS84; **Event:** eventDate: 07/05/2009
- **Type status:** Other material. **Occurrence:** recordedBy: R. Kostova; individualCount: 1; **Location:** countryCode: BG; locality: Malko Tarnovo surroundings; verbatimElevation: 375; verbatimCoordinates: N41°59'01.7", E27°29'49.4"; geodeticDatum: WGS84; **Event:** eventDate: 27/05/2010
- **Type status:** Other material. **Occurrence:** recordedBy: R. Kostova; individualCount: 2; **Location:** countryCode: BG; locality: Sinemorets Vill., PA "Estuary of Veleka river"; verbatimElevation: 6; verbatimCoordinates: N42°03'39.6", E27°57'55.2"; geodeticDatum: WGS84; **Event:** eventDate: 30/05/2010; habitat: swamp forest
- **Type status:** Other material. **Occurrence:** recordedBy: R. Bekchiev; individualCount: 1; **Location:** countryCode: BG; locality: 5 km of Bliznak Village; verbatimElevation: 303; verbatimCoordinates: N42°10'19.8", E27°18'35.5"; geodeticDatum: WGS84; **Event:** eventDate: 08/06/2009
- **Type status:** Other material. **Occurrence:** recordedBy: A. Gijonova; individualCount: 1; **Location:** countryCode: TR; locality: Kiyiköy surroundings; verbatimElevation: 32; verbatimCoordinates: N41°38'12.8", E28°04'08.7"; geodeticDatum: WGS84; **Event:** eventDate: 22/05/2011
- **Type status:** Other material. **Occurrence:** recordedBy: R. Kostova; individualCount: 2; **Location:** countryCode: BG; locality: Kosti Vill., "St. Ilia" Place; verbatimElevation: 35; verbatimCoordinates: N42°03'23.2", E27°45'51.6"; geodeticDatum: WGS84; **Event:** eventDate: 15.04-08.06.2009; habitat: meadow with single trees
- **Type status:** Other material. **Occurrence:** recordedBy: R. Kostova; individualCount: 3; **Location:** countryCode: BG; locality: Sinemorets Vill., PA "Estuary of Veleka river"; verbatimElevation: 6; verbatimCoordinates: N42°03'39.6", E27°57'55.2"; geodeticDatum: WGS84; **Event:** eventDate: 15.04-08.05.2009; habitat: swamp forest
- **Type status:** Other material. **Location:** countryCode: BG; locality: Tsarevo (= Micurin); **Record Level:** bibliographicCitation: Hieke & Wrase (1988: 72)
- **Type status:** Other material. **Occurrence:** recordedBy: P. Beron; individualCount: 1; **Location:** countryCode: BG; locality: Kiten; **Event:** eventDate: 16-22.12.1984; **Record Level:** institutionCode: NMNHS
- **Type status:** Other material. **Occurrence:** recordedBy: B. Guéorguiev; individualCount: 1; **Location:** countryCode: BG; locality: Rezovska River at Uzunbudzhak Reserve; **Event:** eventDate: 26/05/1995; **Record Level:** institutionCode: NMNHS
- **Type status:** Other material. **Occurrence:** recordedBy: B. Guéorguiev; individualCount: 1; **Location:** countryCode: BG; locality: mouth of Veleka River near Sinemorets Vill.; **Event:** eventDate: 28/05/1995; **Record Level:** institutionCode: NMNHS
- **Type status:** Other material. **Occurrence:** recordedBy: B. Guéorguiev; individualCount: 1; **Location:** countryCode: BG; locality: Nature Park Ropotamo; **Event:** eventDate: 29/05/1995; **Record Level:** institutionCode: NMNHS

#### Pterostichus (Platysma) niger niger

(Schaller, 1783)

##### Materials

- **Type status:** Other material. **Occurrence:** recordedBy: R. Kostova; individualCount: 2; **Location:** countryCode: BG; locality: Malko Tarnovo, "Propada" Place; verbatimElevation: 401; verbatimCoordinates: N41°58'49.6", E27°29'25.7"; geodeticDatum: WGS84; **Event:** eventDate: 03.07-07.09.2009; habitat: oriental beech forest
- **Type status:** Other material. **Occurrence:** recordedBy: R. Kostova; individualCount: 1; **Location:** countryCode: BG; locality: Sinemorets Vill., PA "Estuary of Veleka river"; verbatimElevation: 6; verbatimCoordinates: N42°03'39.6", E27°57'55.2"; geodeticDatum: WGS84; **Event:** eventDate: 09.06-02.07.2009; habitat: swamp forest
- **Type status:** Other material. **Location:** countryCode: BG; locality: Malko Tarnovo; **Record Level:** bibliographicCitation: Guéorguiev & Guéorguiev (1995: 127)

#### Pterostichus (Pseudomaseus) anthracinus anthracinus

(Illiger, 1798)

##### Materials

- **Type status:** Other material. **Occurrence:** recordedBy: P. Beron; individualCount: 1; **Location:** countryCode: BG; locality: Kiten; **Event:** eventDate: 16-22.12.1984; **Record Level:** institutionCode: NMNHS
- **Type status:** Other material. **Occurrence:** recordedBy: R. Bekchiev; individualCount: 2; **Location:** countryCode: BG; locality: Kirovo Vill., along Selska River; verbatimElevation: 138; verbatimCoordinates: N42°10'43.0", E27°10'58.2"; geodeticDatum: WGS84; **Event:** eventDate: 05/05/2009
- **Type status:** Other material. **Occurrence:** recordedBy: R. Kostova; individualCount: 1; **Location:** countryCode: BG; locality: Slivarovo Vill., "Shafariitsa" Place; verbatimElevation: 224; verbatimCoordinates: N41°57'37.8", E27°39'34.8"; geodeticDatum: WGS84; **Event:** eventDate: 07/05/2009
- **Type status:** Other material. **Occurrence:** recordedBy: R. Kostova; individualCount: 2; **Location:** countryCode: BG; locality: Malko Tarnovo, Entrance to Vitanovo Reserve; verbatimElevation: 285; verbatimCoordinates: N42°01'22.9", E27°27'22.9"; geodeticDatum: WGS84; **Event:** eventDate: 27/05/2010
- **Type status:** Other material. **Occurrence:** recordedBy: R. Kostova; individualCount: 3; **Location:** countryCode: BG; locality: Sinemorets Vill., PA "Estuary of Veleka river"; verbatimElevation: 6; verbatimCoordinates: N42°03'39.6", E27°57'55.2"; geodeticDatum: WGS84; **Event:** eventDate: 30/05/2010; habitat: swamp forest
- **Type status:** Other material. **Occurrence:** recordedBy: R. Kostova; individualCount: 1; **Location:** countryCode: BG; locality: Kosti Vill., "St. Ilia" Place; verbatimElevation: 35; verbatimCoordinates: N42°03'23.2", E27°45'51.6"; geodeticDatum: WGS84; **Event:** eventDate: 15.04-08.05.2009; habitat: meadow with single trees
- **Type status:** Other material. **Occurrence:** recordedBy: R. Kostova; individualCount: 167; **Location:** countryCode: BG; locality: Sinemorets Vill., PA "Estuary of Veleka river"; verbatimElevation: 6; verbatimCoordinates: N42°03'39.6", E27°57'55.2"; geodeticDatum: WGS84; **Event:** eventDate: 15.04-07.09.2009; habitat: swamp forest

#### Pterostichus (Pseudomaseus) minor minor

(Gyllenhal, 1827)

##### Materials

- **Type status:** Other material. **Occurrence:** recordedBy: R. Bekchiev; individualCount: 1; **Location:** countryCode: BG; locality: Primorsko, Perla Beach; verbatimElevation: 1; verbatimCoordinates: N42°17'10.2", E27°45'13.6"; geodeticDatum: WGS84; **Event:** eventDate: 30/06/2009

#### Pterostichus (Pseudomaseus) nigrita

(Paykull, 1790)

##### Materials

- **Type status:** Other material. **Occurrence:** recordedBy: R. Kostova; individualCount: 1; **Location:** countryCode: BG; locality: Malko Tarnovo, "Indipasqua" Place; verbatimElevation: 211; verbatimCoordinates: N42°00'16.9", E27°39'09.2"; geodeticDatum: WGS84; **Event:** eventDate: 18/04/2009
- **Type status:** Other material. **Occurrence:** recordedBy: R. Bekchiev; individualCount: 1; **Location:** countryCode: BG; locality: Malko Tarnovo, "Indipasqua" Place; verbatimElevation: 211; verbatimCoordinates: N42°00'16.9", E27°39'09.2"; geodeticDatum: WGS84; **Event:** eventDate: 25/05/2009
- **Type status:** Other material. **Occurrence:** recordedBy: R. Radev; individualCount: 1; **Location:** countryCode: BG; locality: Sinemorets Vill.; **Event:** eventDate: 07/08/1989; **Record Level:** collectionID: R. Radev coll.
- **Type status:** Other material. **Location:** countryCode: BG; locality: Tsarevo (= Micurin); **Record Level:** bibliographicCitation: Hieke & Wrase (1988: 73)
- **Type status:** Other material. **Occurrence:** recordedBy: S. Andreev; individualCount: 1; **Location:** countryCode: BG; locality: Aydere River; **Event:** eventDate: 30/05/1982; habitat: moist leaf litter near brook; **Record Level:** institutionCode: NMNHS
- **Type status:** Other material. **Occurrence:** recordedBy: P. Beron; individualCount: 1; **Location:** countryCode: BG; locality: Kiten; **Event:** eventDate: 16-22.12.1984; **Record Level:** institutionCode: NMNHS
- **Type status:** Other material. **Occurrence:** recordedBy: B. Guéorguiev; individualCount: 1; **Location:** countryCode: BG; locality: Veleka River near Mladezko Village; **Event:** eventDate: 25/05/1995; **Record Level:** institutionCode: NMNHS
- **Type status:** Other material. **Occurrence:** recordedBy: B. Guéorguiev; individualCount: 1; **Location:** countryCode: BG; locality: Aydere River; verbatimElevation: 350-450; **Event:** eventDate: 26/05/1995; **Record Level:** institutionCode: NMNHS
- **Type status:** Other material. **Occurrence:** recordedBy: B. Guéorguiev; individualCount: 1; **Location:** countryCode: BG; locality: Rezovska River at Uzunbudzhak Reserve; **Event:** eventDate: 26/05/1995; **Record Level:** institutionCode: NMNHS

#### Pterostichus (Pterostichus) merklii

(J. Frivaldszky, 1879)

##### Materials

- **Type status:** Other material. **Location:** countryCode: BG; locality: Maslen nos Cape; **Record Level:** bibliographicCitation: Guéorguiev (1992: 66); institutionCode: NMNHS
- **Type status:** Other material. **Location:** countryCode: BG; locality: Maslen nos Cape; **Record Level:** bibliographicCitation: Guéorguiev & Guéorguiev (1995: 130)

##### Notes

This record is probably based on mislabelled specimens.

#### Stomis (Stomis) pumicatus pumicatus

(Panzer, 1796)

##### Materials

- **Type status:** Other material. **Occurrence:** recordedBy: R. Bekchiev; individualCount: 1; **Location:** countryCode: BG; locality: Bulgari Vill., PA "Marina reka"; verbatimElevation: 183; verbatimCoordinates: N42°06'41.7", E27°45'53.0"; geodeticDatum: WGS84; **Event:** eventDate: 03/07/2009

#### Calathus (Calathus) distinguendus

Chaudoir, 1846

##### Materials

- **Type status:** Other material. **Occurrence:** recordedBy: R. Kostova; individualCount: 1; **Location:** countryCode: TR; locality: Vize surroundings; verbatimElevation: 366; verbatimCoordinates: N41°35'06.1", E27°47'41.5"; geodeticDatum: WGS84; **Event:** eventDate: 25/05/2011
- **Type status:** Other material. **Location:** countryCode: TR; locality: Poyralı Köyü, near Kirklareli; **Record Level:** bibliographicCitation: Battoni & Vereschagina (1984: 140)
- **Type status:** Other material. **Occurrence:** recordedBy: W. Beier; individualCount: 5; **Location:** countryCode: BG; locality: South of Zvezdets Vill.; verbatimElevation: 303; verbatimCoordinates: N42°05'49.0", E27°25'47.0"; geodeticDatum: WGS84; **Event:** eventDate: 12/10/2005; **Record Level:** collectionCode: cWB

#### Calathus (Calathus) fuscipes

(Goeze, 1777) s.l.

##### Materials

- **Type status:** Other material. **Occurrence:** recordedBy: R. Kostova; individualCount: 1; **Location:** countryCode: TR; locality: Demirköy, Dökümhanesi; verbatimElevation: 179; verbatimCoordinates: N41°49'09.6", E27°48'43.2"; geodeticDatum: WGS84; **Event:** eventDate: 30/09/2009
- **Type status:** Other material. **Occurrence:** recordedBy: R. Kostova; individualCount: 1; **Location:** countryCode: TR; locality: Kiyiköy surroundings; verbatimElevation: 38; verbatimCoordinates: N41°39'25.7", E28°05'11.2"; geodeticDatum: WGS84; **Event:** eventDate: 22/05/2011
- **Type status:** Other material. **Occurrence:** recordedBy: R. Kostova; individualCount: 1; **Location:** countryCode: BG; locality: Slivarovo Vill., "Shafariitsa" Place; verbatimElevation: 224; verbatimCoordinates: N41°57'37.8", E27°39'34.8"; geodeticDatum: WGS84; **Event:** eventDate: 09.06-02.07.2009; habitat: black alder-oak forest
- **Type status:** Other material. **Occurrence:** recordedBy: R. Kostova; individualCount: 1; **Location:** countryCode: BG; locality: Slivarovo Vill., "Shafariitsa" Place; verbatimElevation: 224; verbatimCoordinates: N41°57'37.8", E27°39'34.8"; geodeticDatum: WGS84; **Event:** eventDate: 03.08-07.09.2009; habitat: black alder-oak forest
- **Type status:** Other material. **Occurrence:** recordedBy: R. Kostova; individualCount: 1; **Location:** countryCode: BG; locality: Kondolovo Vill., "Byalata prast" Place; verbatimElevation: 283; verbatimCoordinates: N42°05'49.7", E27°39'56.3"; geodeticDatum: WGS84; **Event:** eventDate: 09.06-02.07.2009; habitat: beech forest with Vaccinium arctostaphylos
- **Type status:** Other material. **Occurrence:** recordedBy: R. Kostova; individualCount: 362; **Location:** countryCode: BG; locality: Kosti Vill., "St. Ilia" Place; verbatimElevation: 35; verbatimCoordinates: N42°03'23.2", E27°45'51.6"; geodeticDatum: WGS84; **Event:** eventDate: 15.04-07.09.2009; habitat: meadow with single trees
- **Type status:** Other material. **Occurrence:** recordedBy: R. Kostova; individualCount: 1; **Location:** countryCode: BG; locality: Bulgari Vill., PA "Marina reka"; verbatimElevation: 183; verbatimCoordinates: N42°06'41.7", E27°45'53.0"; geodeticDatum: WGS84; **Event:** eventDate: 03.08-07.09.2009; habitat: oriental beech-oak forest with Rododendron ponticum
- **Type status:** Other material. **Occurrence:** recordedBy: R. Kostova; individualCount: 1; **Location:** countryCode: BG; locality: Brodilovo Vill., under Papiya peak; verbatimElevation: 336; verbatimCoordinates: N42°06'23.7", E27°50'36.1"; geodeticDatum: WGS84; **Event:** eventDate: 03.07-02.08.2009; habitat: oak forest
- **Type status:** Other material. **Occurrence:** recordedBy: R. Kostova; individualCount: 2; **Location:** countryCode: BG; locality: Sinemorets Vill., PA"Silistar", "Butamyata" Place; verbatimElevation: 17; verbatimCoordinates: N42°03'10.3", E27°59'13.4"; geodeticDatum: WGS84; **Event:** eventDate: 09.05-08.06.2009; habitat: oak forest
- **Type status:** Other material. **Occurrence:** recordedBy: R. Kostova; individualCount: 4; **Location:** countryCode: BG; locality: Sinemorets Vill., PA"Silistar", "Butamyata" Place; verbatimElevation: 17; verbatimCoordinates: N42°03'10.3", E27°59'13.4"; geodeticDatum: WGS84; **Event:** eventDate: 03.08-07.09.2009; habitat: oak forest
- **Type status:** Other material. **Occurrence:** recordedBy: R. Kostova; individualCount: 1; **Location:** countryCode: BG; locality: Sinemorets Vill., PA"Silistar", "Butamyata" Place; verbatimElevation: 10; verbatimCoordinates: N42°03'11.2", E27°59'19.7"; geodeticDatum: WGS84; **Event:** eventDate: 09.05-08.06.2009; habitat: meadow, single shrubs
- **Type status:** Other material. **Occurrence:** recordedBy: R. Kostova; individualCount: 1; **Location:** countryCode: BG; locality: Sinemorets Vill., PA"Silistar", "Butamyata" Place; verbatimElevation: 10; verbatimCoordinates: N42°03'11.2", E27°59'19.7"; geodeticDatum: WGS84; **Event:** eventDate: 03.08-07.09.2009; habitat: meadow, single shrubs
- **Type status:** Other material. **Occurrence:** recordedBy: R. Kostova; individualCount: 2; **Location:** countryCode: BG; locality: Stoilovo Vill., "Petrova niva" Place; verbatimElevation: 234; verbatimCoordinates: N42°03'40.3", E27°31'42.3"; geodeticDatum: WGS84; **Event:** eventDate: 09.06-02.07.2009; habitat: meadow, single shrubs
- **Type status:** Other material. **Occurrence:** recordedBy: R. Kostova; individualCount: 2; **Location:** countryCode: BG; locality: Stoilovo Vill., "Petrova niva" Place; verbatimElevation: 234; verbatimCoordinates: N42°03'40.3", E27°31'42.3"; geodeticDatum: WGS84; **Event:** eventDate: 03.08-07.09.2009; habitat: meadow, single shrubs
- **Type status:** Other material. **Occurrence:** recordedBy: R. Kostova; individualCount: 1; **Location:** countryCode: BG; locality: Sinemorets Vill., PA"Silistar"; verbatimElevation: 23; verbatimCoordinates: N42°01'30.7", E28°00'25.4"; geodeticDatum: WGS84; **Event:** eventDate: 09.05-08.06.2009; habitat: oak forest
- **Type status:** Other material. **Occurrence:** recordedBy: R. Kostova; individualCount: 1; **Location:** countryCode: TR; locality: Igneada surroundings; verbatimElevation: 15; verbatimCoordinates: N41°51'51.0", E27°56'54.1"; geodeticDatum: WGS84; **Event:** eventDate: 06.07-29.09.2009; habitat: swamp forest
- **Type status:** Other material. **Occurrence:** recordedBy: R. Kostova; individualCount: 1; **Location:** countryCode: TR; locality: Gokyaka Vill., Road to Dupnisa Cave; verbatimElevation: 337; verbatimCoordinates: N41°52'20.0", E27°37'06.4"; geodeticDatum: WGS84; **Event:** eventDate: 06.07 - 29.09.2009
- **Type status:** Other material. **Occurrence:** recordedBy: J. Mařan & K. Taborsky; individualCount: 3; **Location:** countryCode: BG; locality: Maslen nos Cape; **Event:** eventDate: VI.1933; **Record Level:** institutionCode: NMP
- **Type status:** Other material. **Occurrence:** recordedBy: C. Purkyně; individualCount: 1; **Location:** countryCode: BG; locality: Strandzha; **Event:** eventDate: VII.1934; **Record Level:** institutionCode: NMP
- **Type status:** Other material. **Occurrence:** recordedBy: K. Taborsky; individualCount: 1; **Location:** countryCode: BG; locality: Kosti Vill.; **Event:** eventDate: no date; **Record Level:** institutionCode: NMP
- **Type status:** Other material. **Occurrence:** recordedBy: K. Taborsky; individualCount: 1; **Location:** countryCode: BG; locality: Velika Papiya; **Event:** eventDate: VII.1934; **Record Level:** institutionCode: NMP
- **Type status:** Other material. **Location:** countryCode: BG; locality: Brodilovo Vill., Papiya Peak; **Record Level:** bibliographicCitation: Guéorguiev & Guéorguiev (1995: 149)
- **Type status:** Other material. **Location:** countryCode: BG; locality: Rezovo Vill.; **Record Level:** bibliographicCitation: Guéorguiev & Guéorguiev (1995: 149)
- **Type status:** Other material. **Occurrence:** recordedBy: P. Drenski; individualCount: 5; **Location:** countryCode: BG; locality: Rezovo Vill.; **Event:** eventDate: 11/06/1933; **Record Level:** institutionCode: NMNHS
- **Type status:** Other material. **Occurrence:** recordedBy: P. Drenski; individualCount: 1; **Location:** countryCode: BG; locality: Rezovo Vill.; **Event:** eventDate: 15/05/1931; **Record Level:** institutionCode: NMNHS
- **Type status:** Other material. **Occurrence:** recordedBy: D. Iltchev; individualCount: 2; **Location:** countryCode: BG; locality: Rezovo Vill.; **Event:** eventDate: 27/06/1921; **Record Level:** institutionCode: NMNHS
- **Type status:** Other material. **Occurrence:** recordedBy: P. Drenski; **Location:** countryCode: BG; locality: Strandzha; **Event:** eventDate: 23/06/1924; **Record Level:** institutionCode: NMNHS
- **Type status:** Other material. **Occurrence:** recordedBy: K. Tuleshkov; individualCount: 6; **Location:** countryCode: BG; locality: Ahtopol; **Event:** eventDate: 15/05/1930; **Record Level:** institutionCode: NMNHS
- **Type status:** Other material. **Location:** countryCode: BG; locality: Ahtopol; **Record Level:** bibliographicCitation: Hieke & Wrase (1988: 89)
- **Type status:** Other material. **Location:** countryCode: BG; locality: Kiten; **Record Level:** bibliographicCitation: Hieke & Wrase (1988: 89)
- **Type status:** Other material. **Location:** countryCode: BG; locality: Kosti Vill.; **Record Level:** bibliographicCitation: Hieke & Wrase (1988: 89)
- **Type status:** Other material. **Location:** countryCode: BG; locality: Tsarevo (=Michurin); **Record Level:** bibliographicCitation: Hieke & Wrase (1988: 89)
- **Type status:** Other material. **Location:** countryCode: BG; locality: Mouth of Ropotamo River; **Record Level:** bibliographicCitation: Hieke & Wrase (1988: 89)
- **Type status:** Other material. **Location:** countryCode: BG; locality: 5 km West of Yasna Polyana Vill.; **Record Level:** bibliographicCitation: Hieke & Wrase (1988: 89)
- **Type status:** Other material. **Occurrence:** recordedBy: P. Beron; individualCount: 2; **Location:** countryCode: BG; locality: Kiten; **Event:** eventDate: 16-22.12.1984; **Record Level:** institutionCode: NMNHS
- **Type status:** Other material. **Occurrence:** recordedBy: B. Guéorguiev; individualCount: 2; **Location:** countryCode: BG; locality: Aydere River; verbatimElevation: 350-450; **Event:** eventDate: 26/05/1995; **Record Level:** institutionCode: NMNHS
- **Type status:** Other material. **Occurrence:** recordedBy: B. Guéorguiev; individualCount: 2; **Location:** countryCode: BG; locality: Rezovska River at Uzunbudzhak Reserve; **Event:** eventDate: 26/05/1995; **Record Level:** institutionCode: NMNHS
- **Type status:** Other material. **Occurrence:** recordedBy: B. Guéorguiev; individualCount: 2; **Location:** countryCode: BG; locality: Sinemorets Village, south bay; **Event:** eventDate: 27/05/1995; **Record Level:** institutionCode: NMNHS
- **Type status:** Other material. **Occurrence:** recordedBy: B. Guéorguiev; individualCount: 8; **Location:** countryCode: BG; locality: Tisovitsa Reserve near Bulgari Vill.; **Event:** eventDate: 27/05/1995; **Record Level:** institutionCode: NMNHS
- **Type status:** Other material. **Occurrence:** recordedBy: B. Guéorguiev; individualCount: 1; **Location:** countryCode: BG; locality: mouth of Veleka River near Sinemorets Vill.; **Event:** eventDate: 28/05/1995; **Record Level:** institutionCode: NMNHS
- **Type status:** Other material. **Occurrence:** recordedBy: B. Guéorguiev; individualCount: 2; **Location:** countryCode: BG; locality: Ahtopol; **Event:** eventDate: 28/05/1995; **Record Level:** institutionCode: NMNHS
- **Type status:** Other material. **Occurrence:** recordedBy: B. Guéorguiev; individualCount: 1; **Location:** countryCode: BG; locality: Arapya, near Tsarevo; **Event:** eventDate: 28/05/1995; **Record Level:** institutionCode: NMNHS
- **Type status:** Other material. **Occurrence:** recordedBy: B. Guéorguiev; individualCount: 1; **Location:** countryCode: BG; locality: Nature Park Ropotamo; **Event:** eventDate: 29/05/1995; **Record Level:** institutionCode: NMNHS

#### Calathus (Calathus) longicollis

Motschulsky, 1865

##### Materials

- **Type status:** Other material. **Occurrence:** recordedBy: R. Kostova; individualCount: 1; **Location:** countryCode: BG; locality: Sinemorets Vill., PA"Silistar", "Butamyata" Place; verbatimElevation: 17; verbatimCoordinates: N42°03'10.3", E27°59'13.4"; geodeticDatum: WGS84; **Event:** eventDate: 09.06-02.07.2009; habitat: oak forest
- **Type status:** Other material. **Occurrence:** recordedBy: R. Kostova; individualCount: 1; **Location:** countryCode: BG; locality: Sinemorets Vill., PA"Silistar", "Butamyata" Place; verbatimElevation: 17; verbatimCoordinates: N42°03'10.3", E27°59'13.4"; geodeticDatum: WGS84; **Event:** eventDate: 03.08-07.09.2009; habitat: oak forest
- **Type status:** Other material. **Occurrence:** recordedBy: R. Kostova; individualCount: 1; **Location:** countryCode: TR; locality: Kiyiköy surroundings; verbatimElevation: 38; verbatimCoordinates: N41°39'25.7", E28°05'11.2"; geodeticDatum: WGS84; **Event:** eventDate: 06.07 - 29.09.2009; habitat: oak with Arbutus unedo
- **Type status:** Other material. **Location:** countryCode: TR; locality: Poyralı Köyü, near Kirklareli; **Record Level:** bibliographicCitation: Battoni & Vereschagina (1984: 148)
- **Type status:** Other material. **Location:** countryCode: BG; locality: Ahtopol; **Record Level:** bibliographicCitation: Wrase (1991: 10)
- **Type status:** Other material. **Location:** countryCode: BG; locality: Ahtopol; **Record Level:** bibliographicCitation: Guéorguiev & Guéorguiev (1995: 150)

#### Calathus (Neocalathus) ambiguus ambiguus

(Paykull, 1790)

##### Materials

- **Type status:** Other material. **Occurrence:** recordedBy: R. Kostova; individualCount: 1; **Location:** countryCode: BG; locality: Stoilovo Vill., "Petrova niva" Place; verbatimElevation: 132; verbatimCoordinates: N42°03'41.9", E27°31'58.9"; geodeticDatum: WGS84; **Event:** eventDate: 06/05/2009; habitat: meadow, single shrubs
- **Type status:** Other material. **Occurrence:** recordedBy: W. Beier; individualCount: 1; **Location:** countryCode: BG; locality: Mouth of Veleka River near Sinemorets Vill.; verbatimElevation: 20; verbatimCoordinates: N42°03'33.0", E27°58'04.0"; geodeticDatum: WGS84; **Event:** eventDate: 14/07/2006; **Record Level:** collectionCode: cWB
- **Type status:** Other material. **Occurrence:** recordedBy: J. Mařan & K. Taborsky; individualCount: 3; **Location:** countryCode: BG; locality: Maslen nos Cape; **Event:** eventDate: VI.1933; **Record Level:** institutionCode: NMP
- **Type status:** Other material. **Occurrence:** recordedBy: K. Tuleshkov; individualCount: 1; **Location:** countryCode: BG; locality: Ahtopol; **Event:** eventDate: 15/05/1930; **Record Level:** institutionCode: NMNHS
- **Type status:** Other material. **Location:** countryCode: BG; locality: Ahtopol; **Record Level:** bibliographicCitation: Hieke & Wrase (1988: 90)

#### Calathus (Neocalathus) cinctus

Motschulsky, 1850

##### Materials

- **Type status:** Other material. **Occurrence:** recordedBy: R. Kostova; **Location:** countryCode: BG; locality: Sinemorets Vill., PA"Silistar", "Butamyata" Place; verbatimElevation: 17; verbatimCoordinates: N42°03'10.3", E27°59'13.4"; geodeticDatum: WGS84; **Event:** eventDate: 08.05-09.06.2009; habitat: oak forest
- **Type status:** Other material. **Occurrence:** recordedBy: R. Kostova; **Location:** countryCode: BG; locality: Brodilovo Vill., under Papiya peak; verbatimElevation: 336; verbatimCoordinates: N42°06'23.7", E27°50'36.1"; geodeticDatum: WGS84; **Event:** eventDate: 08.05-09.06.2009; habitat: oak forest

#### Calathus (Neocalathus) melanocephalus melanocephalus

(Linnaeus, 1758)

##### Materials

- **Type status:** Other material. **Occurrence:** recordedBy: R. Kostova; individualCount: 1; **Location:** countryCode: BG; locality: Stoilovo Vill., "Petrova niva" Place; verbatimElevation: 132; verbatimCoordinates: N42°03'41.9", E27°31'58.9"; geodeticDatum: WGS84; **Event:** eventDate: 06/05/2009; habitat: meadow, single shrubs
- **Type status:** Other material. **Occurrence:** recordedBy: R. Kostova; individualCount: 49; **Location:** countryCode: BG; locality: Kosti Vill., "St. Ilia" Place; verbatimElevation: 35; verbatimCoordinates: N42°03'23.2", E27°45'51.6"; geodeticDatum: WGS84; **Event:** eventDate: 15.04-07.09.2009; habitat: meadow with single trees
- **Type status:** Other material. **Occurrence:** recordedBy: R. Kostova; individualCount: 1; **Location:** countryCode: BG; locality: Stoilovo Vill., "Petrova niva" Place; verbatimElevation: 234; verbatimCoordinates: N42°03'40.3", E27°31'42.3"; geodeticDatum: WGS84; **Event:** eventDate: 09.06-02.07.2009; habitat: meadow, single shrubs
- **Type status:** Other material. **Occurrence:** recordedBy: J. Mařan & K. Taborsky; individualCount: 1; **Location:** countryCode: BG; locality: Maslen nos Cape; **Event:** eventDate: VI.1933; **Record Level:** institutionCode: NMP
- **Type status:** Other material. **Occurrence:** recordedBy: P. Beron; individualCount: 4; **Location:** countryCode: BG; locality: Kiten; **Event:** eventDate: 16-22.12.1984; **Record Level:** institutionCode: NMNHS
- **Type status:** Other material. **Occurrence:** recordedBy: B. Guéorguiev; individualCount: 1; **Location:** countryCode: BG; locality: Aydere River; verbatimElevation: 350-450; **Event:** eventDate: 26/05/1995; **Record Level:** institutionCode: NMNHS

#### Calathus (Neocalathus) metallicus

Dejean, 1828

##### Materials

- **Type status:** Other material. **Location:** countryCode: BG; locality: Maslen nos Cape; **Record Level:** bibliographicCitation: Guéorguiev & Guéorguiev (1995: 151)

##### Notes

This record is probably based on mislabelled specimen.

#### Calathus (Neocalathus) mollis mollis

(Marsham, 1802)

##### Materials

- **Type status:** Other material. **Location:** countryCode: BG; locality: Kiten; **Record Level:** bibliographicCitation: Hieke & Wrase (1988: 91)

#### Dolichus halensis

(Schaller, 1783)

##### Materials

- **Type status:** Other material. **Occurrence:** recordedBy: S. Petrov; individualCount: 1; **Location:** countryCode: BG; locality: Vitanovo Forest Reserve; **Event:** eventDate: 1-4.08.1999; habitat: malaise trap; **Record Level:** institutionCode: NMNHS

#### Laemostenus (Laemostenus) venustus

(Dejean, 1828)

##### Materials

- **Type status:** Other material. **Occurrence:** recordedBy: R. Kostova; individualCount: 2; **Location:** countryCode: BG; locality: Bliznak Vill., PA"Bataka"; verbatimElevation: 324; verbatimCoordinates: N42°11'37.3", E27°19'35.8"; geodeticDatum: WGS84; **Event:** eventDate: 15.04-08.06.2009; habitat: oak forest
- **Type status:** Other material. **Occurrence:** recordedBy: R. Kostova; individualCount: 5; **Location:** countryCode: BG; locality: Malko Tarnovo, "Propada" Place; verbatimElevation: 401; verbatimCoordinates: N41°58'49.6", E27°29'25.7"; geodeticDatum: WGS84; **Event:** eventDate: 09.05-02.07.2009; habitat: oriental beech forest
- **Type status:** Other material. **Occurrence:** recordedBy: R. Kostova; individualCount: 1; **Location:** countryCode: BG; locality: Kosti Vill., "St. Ilia" Place; verbatimElevation: 35; verbatimCoordinates: N42°03'23.2", E27°45'51.6"; geodeticDatum: WGS84; **Event:** eventDate: 09.05-08.06.2009; habitat: meadow with single trees
- **Type status:** Other material. **Occurrence:** recordedBy: V. Sakalian; individualCount: 1; **Location:** countryCode: BG; locality: Sinemorets; **Event:** eventDate: 20/06/1991; **Record Level:** institutionCode: NMNHS
- **Type status:** Other material. **Occurrence:** recordedBy: N. Vihodcevsky; individualCount: 2; **Location:** countryCode: BG; locality: Kachul Place; **Event:** eventDate: 01/07/1955; **Record Level:** institutionCode: NMNHS

#### Laemostenus (Pristonychus) cimmerius

(Fischer-Waldheim, 1823) s.l.

##### Materials

- **Type status:** Other material. **Occurrence:** recordedBy: R. Bekchiev; individualCount: 1; **Location:** countryCode: TR; locality: Sarpdere Vill., Dupnisa Cave surroundings; verbatimElevation: 356; verbatimCoordinates: N41°50'26.0", E27°33'22.4"; geodeticDatum: WGS84; **Event:** eventDate: 05/07/2009
- **Type status:** Other material. **Occurrence:** recordedBy: R. Bekchiev; individualCount: 1; **Location:** countryCode: TR; locality: Sarpdere Vill., Dupnisa Cave surroundings; verbatimElevation: 356; verbatimCoordinates: N41°50'26.0", E27°33'22.4"; geodeticDatum: WGS84; **Event:** eventDate: 23/05/2010
- **Type status:** Other material. **Occurrence:** recordedBy: R. Kostova; individualCount: 93; **Location:** countryCode: BG; locality: Bliznak Vill., PA"Bataka"; verbatimElevation: 324; verbatimCoordinates: N42°11'37.3", E27°19'35.8"; geodeticDatum: WGS84; **Event:** eventDate: 15.04-07.09.2009; habitat: oak forest
- **Type status:** Other material. **Occurrence:** recordedBy: R. Kostova; individualCount: 43; **Location:** countryCode: BG; locality: Malko Tarnovo, "Propada" Place; verbatimElevation: 401; verbatimCoordinates: N41°58'49.6", E27°29'25.7"; geodeticDatum: WGS84; **Event:** eventDate: 15.04-07.09.2009; habitat: oriental beech forest
- **Type status:** Other material. **Occurrence:** recordedBy: R. Kostova; individualCount: 1; **Location:** countryCode: BG; locality: Slivarovo Vll. surroundings; verbatimElevation: 331; verbatimCoordinates: N41°58'11.9", E27°39'31.1"; geodeticDatum: WGS84; **Event:** eventDate: 03.08-07.09.2009; habitat: shrubs, mainly Calluna vulgaris
- **Type status:** Other material. **Occurrence:** recordedBy: R. Kostova; individualCount: 1; **Location:** countryCode: BG; locality: Kondolovo Vill., "Byalata prast" Place; verbatimElevation: 271; verbatimCoordinates: N42°05'47.7", E27°39'55.0"; geodeticDatum: WGS84; **Event:** eventDate: 03.07-02.08.2009; habitat: oak-beech mixed forest
- **Type status:** Other material. **Occurrence:** recordedBy: R. Kostova; individualCount: 1; **Location:** countryCode: BG; locality: Brodilovo Vill., under Papiya peak; verbatimElevation: 336; verbatimCoordinates: N42°06'23.7", E27°50'36.1"; geodeticDatum: WGS84; **Event:** eventDate: 03.07-02.08.2009; habitat: oak forest
- **Type status:** Other material. **Occurrence:** recordedBy: R. Kostova; individualCount: 12; **Location:** countryCode: BG; locality: Sinemorets Vill., PA"Silistar", "Butamyata" Place; verbatimElevation: 17; verbatimCoordinates: N42°03'10.3", E27°59'13.4"; geodeticDatum: WGS84; **Event:** eventDate: 15.04-02.07.2009; habitat: oak forest
- **Type status:** Other material. **Occurrence:** recordedBy: R. Kostova; individualCount: 1; **Location:** countryCode: BG; locality: Sinemorets Vill., PA"Silistar", "Butamyata" Place; verbatimElevation: 17; verbatimCoordinates: N42°03'10.3", E27°59'13.4"; geodeticDatum: WGS84; **Event:** eventDate: 03.08-07.09.2009; habitat: oak forest
- **Type status:** Other material. **Occurrence:** recordedBy: R. Kostova; individualCount: 2; **Location:** countryCode: BG; locality: Sinemorets Vill., PA"Silistar", "Butamyata" Place; verbatimElevation: 10; verbatimCoordinates: N42°03'11.2", E27°59'19.7"; geodeticDatum: WGS84; **Event:** eventDate: 15.04-08.06.2009; habitat: meadow, single shrubs
- **Type status:** Other material. **Occurrence:** recordedBy: R. Kostova; individualCount: 26; **Location:** countryCode: BG; locality: Mladezhko Vill., the springs of Mladezhka River; verbatimElevation: 231; verbatimCoordinates: N42°09'04.5", E27°21'26.1"; geodeticDatum: WGS84; **Event:** eventDate: 09.05-07.09.2009; habitat: black alder and hornbeam forest
- **Type status:** Other material. **Occurrence:** recordedBy: R. Kostova; individualCount: 1; **Location:** countryCode: TR; locality: Igneada surroundings; verbatimElevation: 15; verbatimCoordinates: N41°51'51.0", E27°56'54.1"; geodeticDatum: WGS84; **Event:** eventDate: 06.07-29.09.2009; habitat: swamp forest
- **Type status:** Other material. **Location:** countryCode: BG; locality: Strandzha; **Record Level:** bibliographicCitation: Guéorguiev & Guéorguiev (1995: 187, as L. terricola punctatus)
- **Type status:** Other material. **Location:** countryCode: BG; locality: Isperets Peak; **Record Level:** bibliographicCitation: Guéorguiev & Guéorguiev (1995: 187, as L. terricola punctatus)
- **Type status:** Other material. **Location:** countryCode: BG; locality: Kosti Vill.; **Record Level:** bibliographicCitation: Guéorguiev & Guéorguiev (1995: 187, as L. terricola punctatus)
- **Type status:** Other material. **Occurrence:** recordedBy: R. Radev; **Location:** countryCode: BG; locality: Sinemorets Vill.; **Event:** eventDate: 07/08/1989; **Record Level:** collectionID: R. Radev coll.
- **Type status:** Other material. **Occurrence:** recordedBy: P. Petkov; individualCount: 1; **Location:** countryCode: BG; locality: Kosti Vill.; **Event:** eventDate: 29-30.04.1921; **Record Level:** institutionCode: NMNHS
- **Type status:** Other material. **Location:** countryCode: BG; locality: Ahtopol; **Record Level:** bibliographicCitation: Casale (1988: 809, as L. cimmerius weiratheri)
- **Type status:** Other material. **Location:** countryCode: BG; locality: Ahtopol; **Record Level:** bibliographicCitation: Hieke & Wrase (1988: 93)
- **Type status:** Other material. **Occurrence:** recordedBy: T. Ivanova; individualCount: 1; **Location:** countryCode: BG; locality: Bratanova Peshtera Cave at Vitanovo Reserve; **Event:** eventDate: 05/01/2000; **Record Level:** institutionCode: NMNHS
- **Type status:** Other material. **Occurrence:** recordedBy: B. Petrov; individualCount: 1; **Location:** countryCode: BG; locality: Cave Leyarnitsata near Mladezko Vill.; verbatimElevation: 250; verbatimCoordinates: N42°09'05.7", E27°21'15.1"; **Event:** eventDate: 17/07/2002; **Record Level:** institutionCode: NMNHS
- **Type status:** Other material. **Occurrence:** recordedBy: P. Stoev & S. Lazarov; individualCount: 4; **Location:** countryCode: TR; locality: Dupnitsa Cave, 4 km SW Sardere Vill.; verbatimElevation: 348; verbatimCoordinates: N41°50'26.8", E27°33'21.5"; **Event:** eventDate: 25/07/2006; habitat: underground river, clay, guano; fieldNumber: un; **Record Level:** institutionCode: NMNHS
- **Type status:** Other material. **Occurrence:** recordedBy: B. Petrov; individualCount: 2; **Location:** countryCode: BG; locality: Cave Leyarnitsata near Mladezko Vill.; verbatimElevation: 250; verbatimCoordinates: N42°09'05.7", E27°21'15.1"; **Event:** eventDate: 05/08/2006; **Record Level:** institutionCode: NMNHS
- **Type status:** Other material. **Occurrence:** recordedBy: B. Petrov; individualCount: 2; **Location:** countryCode: BG; locality: Cellar-cave in "Sveta Troitsa" Chapel, 4km NW Gramatikovo Vill.; verbatimElevation: 270; verbatimCoordinates: N42°05'31.2", E27°37'21.3"; **Event:** eventDate: 08/08/2006; **Record Level:** institutionCode: NMNHS
- **Type status:** Other material. **Occurrence:** recordedBy: B. Petrov; individualCount: 1; **Location:** countryCode: BG; locality: Cave Elenina doupka, 2km W of Byala voda Vill.; verbatimElevation: 245; verbatimCoordinates: N42°09'50.4", E27°25'56.4"; **Event:** eventDate: 18/09/2006; **Record Level:** institutionCode: NMNHS

#### Synuchus (Synuchus) vivalis vivalis

(Illiger, 1798)

##### Materials

- **Type status:** Other material. **Occurrence:** recordedBy: R. Bekchiev; individualCount: 1; **Location:** countryCode: TR; locality: Sislioba Vill. surroundings; verbatimElevation: 49; verbatimCoordinates: N41°57'44.2", E27°54'36.1"; geodeticDatum: WGS84; **Event:** eventDate: 08/07/2009
- **Type status:** Other material. **Occurrence:** recordedBy: R. Kostova; individualCount: 1; **Location:** countryCode: BG; locality: Slivarovo Vll. surroundings; verbatimElevation: 331; verbatimCoordinates: N41°58'11.9", E27°39'31.1"; geodeticDatum: WGS84; **Event:** eventDate: 09.06-02.07.2009; habitat: shrubs, mainly Calluna vulgaris
- **Type status:** Other material. **Occurrence:** recordedBy: R. Kostova; individualCount: 1; **Location:** countryCode: BG; locality: Bulgari Vill., PA "Marina reka"; verbatimElevation: 183; verbatimCoordinates: N42°06'41.7", E27°45'53.0"; geodeticDatum: WGS84; **Event:** eventDate: 03.07-02.08.2009; habitat: oriental beech-oak forest with Rododendron ponticum
- **Type status:** Other material. **Occurrence:** recordedBy: E. Migliaccio & M. Langourov; individualCount: 1; **Location:** countryCode: BG; locality: Kachulski dol Place near Veleka River; verbatimElevation: 80; **Event:** eventDate: 25-29.05.2003; **Record Level:** institutionCode: NMNHS

#### Amara (Amara) aenea

(DeGeer, 1774)

##### Materials

- **Type status:** Other material. **Occurrence:** recordedBy: R. Kostova; individualCount: 1; **Location:** countryCode: BG; locality: Slivarovo Vill., "Shafariitsa" Place; verbatimElevation: 224; verbatimCoordinates: N41°57'37.8", E27°39'34.8"; geodeticDatum: WGS84; **Event:** eventDate: 19/04/2009
- **Type status:** Other material. **Occurrence:** recordedBy: R. Kostova; individualCount: 1; **Location:** countryCode: BG; locality: Stoilovo Vill., "Sredoka" Reserve, along Mechi dol River; verbatimElevation: 206; verbatimCoordinates: N42°01'51.1", E27°30'50.1"; geodeticDatum: WGS84; **Event:** eventDate: 19/04/2009
- **Type status:** Other material. **Occurrence:** recordedBy: R. Bekchiev; individualCount: 2; **Location:** countryCode: BG; locality: Stoilovo Vill., "Petrova niva" Place; verbatimElevation: 132; verbatimCoordinates: N42°03'41.9", E27°31'58.9"; geodeticDatum: WGS84; **Event:** eventDate: 06/05/2009; habitat: meadow, single shrubs
- **Type status:** Other material. **Occurrence:** recordedBy: R. Kostova; individualCount: 1; **Location:** countryCode: BG; locality: Sinemorets Vill., PA "Estuary of Veleka river"; verbatimElevation: 5; verbatimCoordinates: N42°03'39.6", E27°57'55.2"; geodeticDatum: WGS84; **Event:** eventDate: 30/05/2010; habitat: meadow
- **Type status:** Other material. **Occurrence:** recordedBy: R. Kostova; individualCount: 1; **Location:** countryCode: TR; locality: Kiyiköy surroundings; verbatimElevation: 32; verbatimCoordinates: N41°38'12.8", E28°04'08.7"; geodeticDatum: WGS84; **Event:** eventDate: 22/05/2011
- **Type status:** Other material. **Occurrence:** recordedBy: R. Bekchiev; individualCount: 1; **Location:** countryCode: TR; locality: Kiyiköy surroundings; verbatimElevation: 38; verbatimCoordinates: N41°39'25.7", E28°05'11.2"; geodeticDatum: WGS84; **Event:** eventDate: 22/05/2011
- **Type status:** Other material. **Occurrence:** recordedBy: R. Kostova; individualCount: 1; **Location:** countryCode: BG; locality: Kosti Vill., "St. Ilia" Place; verbatimElevation: 35; verbatimCoordinates: N42°03'23.2", E27°45'51.6"; geodeticDatum: WGS84; **Event:** eventDate: 09.05-08.06.2009; habitat: meadow with single trees
- **Type status:** Other material. **Location:** countryCode: BG; locality: Ahtopol; **Record Level:** bibliographicCitation: Hieke & Wrase (1988: 100)
- **Type status:** Other material. **Location:** countryCode: BG; locality: Brodilovo Vill.; **Record Level:** bibliographicCitation: Hieke & Wrase (1988: 100)
- **Type status:** Other material. **Location:** countryCode: BG; locality: Kiten; **Record Level:** bibliographicCitation: Hieke & Wrase (1988: 100)
- **Type status:** Other material. **Location:** countryCode: BG; locality: Kosti Vill.; **Record Level:** bibliographicCitation: Hieke & Wrase (1988: 100)
- **Type status:** Other material. **Location:** countryCode: BG; locality: Maslen nos Cape; **Record Level:** bibliographicCitation: Hieke & Wrase (1988: 100)
- **Type status:** Other material. **Location:** countryCode: BG; locality: Tsarevo (= Micurin); **Record Level:** bibliographicCitation: Hieke & Wrase (1988: 100)
- **Type status:** Other material. **Location:** countryCode: BG; locality: Primorsko; **Record Level:** bibliographicCitation: Hieke & Wrase (1988: 100)
- **Type status:** Other material. **Location:** countryCode: BG; locality: Ropotamo; **Record Level:** bibliographicCitation: Hieke & Wrase (1988: 100)
- **Type status:** Other material. **Location:** countryCode: BG; locality: Malko Tarnovo env.; **Record Level:** bibliographicCitation: Hieke & Wrase (1988: 100)
- **Type status:** Other material. **Location:** countryCode: BG; locality: Strandzha; **Record Level:** bibliographicCitation: Hieke & Wrase (1988: 100)

#### Amara (Amara) anthobia

A. Villa & G.B. Villa, 1833

##### Materials

- **Type status:** Other material. **Occurrence:** recordedBy: I. Gijonov; individualCount: 4; **Location:** countryCode: BG; locality: Mladezhko Vill., the springs of Mladezhka River; verbatimElevation: 231; verbatimCoordinates: N42°09'04.5", E27°21'26.1"; geodeticDatum: WGS84; **Event:** eventDate: 17/04/2009; habitat: black alder and hornbeam forest
- **Type status:** Other material. **Occurrence:** recordedBy: R. Kostova; individualCount: 2; **Location:** countryCode: TR; locality: Along Pinarhisar - Demirköy road; verbatimElevation: 618; verbatimCoordinates: N41°47'57.7", E27°44'02.4"; geodeticDatum: WGS84; **Event:** eventDate: 06/07/2009
- **Type status:** Other material. **Occurrence:** recordedBy: R. Kostova; individualCount: 1; **Location:** countryCode: TR; locality: Kiyiköy surroundings; verbatimElevation: 38; verbatimCoordinates: N41°39'25.7", E28°05'11.2"; geodeticDatum: WGS84; **Event:** eventDate: 22/05/2011
- **Type status:** Other material. **Occurrence:** recordedBy: R. Kostova; individualCount: 1; **Location:** countryCode: TR; locality: Vize surroundings; verbatimElevation: 366; verbatimCoordinates: N41°35'06.1", E27°47'41.5"; geodeticDatum: WGS84; **Event:** eventDate: 25/05/2011
- **Type status:** Other material. **Occurrence:** recordedBy: R. Kostova; individualCount: 2; **Location:** countryCode: BG; locality: Slivarovo Vill., "Shafariitsa" Place; verbatimElevation: 224; verbatimCoordinates: N41°57'37.8", E27°39'34.8"; geodeticDatum: WGS84; **Event:** eventDate: 15.04-08.06.2009; habitat: black alder-oak forest
- **Type status:** Other material. **Occurrence:** recordedBy: R. Kostova; individualCount: 2; **Location:** countryCode: BG; locality: Kondolovo Vill., "Byalata prast" Place; verbatimElevation: 271; verbatimCoordinates: N42°05'47.7", E27°39'55.0"; geodeticDatum: WGS84; **Event:** eventDate: 15.04-08.06.2009; habitat: oak-beech mixed forest
- **Type status:** Other material. **Location:** countryCode: BG; locality: Ahtopol; **Record Level:** bibliographicCitation: Hieke & Wrase (1988: 102)
- **Type status:** Other material. **Location:** countryCode: BG; locality: Tsarevo (= Micurin); **Record Level:** bibliographicCitation: Hieke & Wrase (1988: 102)
- **Type status:** Other material. **Location:** countryCode: BG; locality: Primorsko; **Record Level:** bibliographicCitation: Hieke & Wrase (1988: 102)

#### Amara (Amara) convexior

Stephens, 1828

##### Materials

- **Type status:** Other material. **Occurrence:** recordedBy: R. Kostova; individualCount: 1; **Location:** countryCode: BG; locality: Malko Tarnovo, "Propada" Place; verbatimElevation: 388; verbatimCoordinates: N41°58'52.5", E27°29'28.6"; geodeticDatum: WGS84; **Event:** eventDate: 17/04/2009; habitat: meadow
- **Type status:** Other material. **Occurrence:** recordedBy: R. Kostova; individualCount: 1; **Location:** countryCode: BG; locality: Bulgari Vill., PA "Marina reka"; verbatimElevation: 183; verbatimCoordinates: N42°06'41.7", E27°45'53.0"; geodeticDatum: WGS84; **Event:** eventDate: 15.04-08.05.2009; habitat: oriental beech-oak forest with Rododendron ponticum
- **Type status:** Other material. **Occurrence:** recordedBy: R. Kostova; individualCount: 2; **Location:** countryCode: BG; locality: Malko Tarnovo, "Propada" Place; verbatimElevation: 401; verbatimCoordinates: N41°58'49.6", E27°29'25.7"; geodeticDatum: WGS84; **Event:** eventDate: 09.05-08.06.2009; habitat: oriental beech forest
- **Type status:** Other material. **Occurrence:** recordedBy: R. Kostova; individualCount: 68; **Location:** countryCode: BG; locality: Slivarovo Vill., "Shafariitsa" Place; verbatimElevation: 224; verbatimCoordinates: N41°57'37.8", E27°39'34.8"; geodeticDatum: WGS84; **Event:** eventDate: 15.04-07.09.2009; habitat: black alder-oak forest

#### Amara (Amara) eurynota

(Panzer, 1796)

##### Materials

- **Type status:** Other material. **Location:** countryCode: BG; locality: Strandzha; **Record Level:** bibliographicCitation: Hieke & Wrase (1988: 101)

#### Amara (Amara) lucida

(Duftschmid, 1812)

##### Materials

- **Type status:** Other material. **Occurrence:** recordedBy: I. Gijonov; individualCount: 2; **Location:** countryCode: BG; locality: Slivarovo Village; verbatimElevation: 338; verbatimCoordinates: N41°58'11.9", E27°39'31.1"; geodeticDatum: WGS84; **Event:** eventDate: 19/04/2009
- **Type status:** Other material. **Occurrence:** recordedBy: R. Kostova; individualCount: 1; **Location:** countryCode: BG; locality: Stoilovo Vill., "Petrova niva" Place; verbatimElevation: 234; verbatimCoordinates: N42°03'40.3", E27°31'42.3"; geodeticDatum: WGS84; **Event:** eventDate: 09.05-08.06.2009; habitat: meadow, single shrubs
- **Type status:** Other material. **Occurrence:** recordedBy: W. Beier; individualCount: 2; **Location:** countryCode: BG; locality: Brashlyan Vill.; verbatimElevation: 374; verbatimCoordinates: N42°02'30.0", E27°25'40.0"; geodeticDatum: WGS84; **Event:** eventDate: 08/05/2002; **Record Level:** collectionCode: cWB
- **Type status:** Other material. **Location:** countryCode: BG; locality: Maslen nos Cape; **Record Level:** bibliographicCitation: Hieke & Wrase (1988: 102)

#### Amara (Amara) lunicollis

Schiødte, 1837

##### Materials

- **Type status:** Other material. **Location:** countryCode: BG; locality: Strandzha; **Record Level:** bibliographicCitation: Hieke & Wrase (1988: 99)

#### Amara (Amara) ovata

(Fabricius, 1792)

##### Materials

- **Type status:** Other material. **Occurrence:** recordedBy: R. Kostova; individualCount: 1; **Location:** countryCode: TR; locality: Sergen Vill. surroundings; verbatimElevation: 818; verbatimCoordinates: N41°44'37.0", E27°42'24.8"; geodeticDatum: WGS84; **Event:** eventDate: 25/05/2011
- **Type status:** Other material. **Occurrence:** recordedBy: R. Kostova; individualCount: 2; **Location:** countryCode: BG; locality: Slivarovo Vill., "Shafariitsa" Place; verbatimElevation: 224; verbatimCoordinates: N41°57'37.8", E27°39'34.8"; geodeticDatum: WGS84; **Event:** eventDate: 15.04-08.06.2009; habitat: black alder-oak forest
- **Type status:** Other material. **Occurrence:** recordedBy: R. Kostova; individualCount: 3; **Location:** countryCode: BG; locality: Kosti Vill., "St. Ilia" Place; verbatimElevation: 35; verbatimCoordinates: N42°03'23.2", E27°45'51.6"; geodeticDatum: WGS84; **Event:** eventDate: 15.04-08.06.2009; habitat: meadow with single trees
- **Type status:** Other material. **Location:** countryCode: BG; locality: Strandzha; **Record Level:** bibliographicCitation: Hieke & Wrase (1988: 96)

#### Amara (Bradytus) apricaria

(Paykull, 1790)

##### Materials

- **Type status:** Other material. **Location:** countryCode: BG; locality: Varvara Vill.; **Record Level:** bibliographicCitation: Hieke & Wrase (1988: 107)

#### Amara (Bradytus) consularis

(Duftschmid, 1812)

##### Materials

- **Type status:** Other material. **Occurrence:** recordedBy: R. Bekchiev; individualCount: 1; **Location:** countryCode: TR; locality: Sarpdere Vill., Dupnisa Cave surroundings; verbatimElevation: 440; verbatimCoordinates: N41°49'56.3", E27°33'24.0"; geodeticDatum: WGS84; **Event:** eventDate: 08/07/2009
- **Type status:** Other material. **Location:** countryCode: BG; locality: Varvara Vill.; **Record Level:** bibliographicCitation: Hieke & Wrase (1988: 108)

#### Amara (Bradytus) crenata

Dejean, 1828

##### Materials

- **Type status:** Other material. **Occurrence:** recordedBy: R. Bekchiev; individualCount: 2; **Location:** countryCode: TR; locality: Sarpdere Vill., Dupnisa Cave surroundings; verbatimElevation: 440; verbatimCoordinates: N41°49'56.3", E27°33'24.0"; geodeticDatum: WGS84; **Event:** eventDate: 08/07/2009

#### Amara (Bradytus) majuscula

(Chaudoir, 1850)

##### Materials

- **Type status:** Other material. **Location:** countryCode: BG; locality: Varvara Vill., near Ahtopol; **Record Level:** bibliographicCitation: Hieke & Wrase (1988: 107)

#### Amara (Paracelia) serdicana

Apfelbeck, 1904

##### Materials

- **Type status:** Other material. **Location:** countryCode: BG; locality: Strandzha; **Record Level:** bibliographicCitation: Hieke & Wrase (1988: 106)

#### Amara (Xenocelia) fusca

Dejean, 1828

##### Materials

- **Type status:** Other material. **Location:** countryCode: BG; locality: Ropotamo; **Record Level:** bibliographicCitation: Hieke & Wrase (1988: 104)
- **Type status:** Other material. **Occurrence:** recordedBy: W. Beier; individualCount: 1; **Location:** countryCode: BG; locality: Mouth of Ropotamo River; **Event:** eventDate: 17/05/2003; **Record Level:** collectionCode: cWB

#### Amara (Xenocelia) ingenua

(Duftcshmid, 1812)

##### Materials

- **Type status:** Other material. **Occurrence:** recordedBy: R. Kostova; individualCount: 1; **Location:** countryCode: BG; locality: Primorsko, Dunes; verbatimElevation: 6; verbatimCoordinates: N41°47'44.0", E27°59'50.4"; geodeticDatum: WGS84; **Event:** eventDate: 10/05/2009

#### Amara (Zezea) concinna

C. Zimmermann, 1832

##### Materials

- **Type status:** Other material. **Location:** countryCode: BG; locality: Primorsko; **Record Level:** bibliographicCitation: Hieke (1970: 142)

#### Amara (Zezea) tricuspidata

Dejean, 1831

##### Materials

- **Type status:** Other material. **Occurrence:** recordedBy: R. Kostova; individualCount: 1; **Location:** countryCode: BG; locality: Bliznak Vill., PA"Bataka"; verbatimElevation: 324; verbatimCoordinates: N42°11'37.3", E27°19'35.8"; geodeticDatum: WGS84; **Event:** eventDate: 09.06-02.07.2009; habitat: oak forest
- **Type status:** Other material. **Occurrence:** recordedBy: R. Kostova; individualCount: 2; **Location:** countryCode: BG; locality: Bliznak Vill., PA"Bataka"; verbatimElevation: 324; verbatimCoordinates: N42°11'37.3", E27°19'35.8"; geodeticDatum: WGS84; **Event:** eventDate: 03.08-07.09.2009; habitat: oak forest

#### Zabrus (Pelor) spinipes spinipes

(Fabricius, 1798)

##### Materials

- **Type status:** Other material. **Location:** countryCode: BG; locality: Malko Tarnovo env.; **Record Level:** bibliographicCitation: Drensky et all. (1951: 283)

#### Zabrus (Zabrus) tenebrioides tenebrioides

(Goeze, 1777)

##### Materials

- **Type status:** Other material. **Occurrence:** recordedBy: W. Beier; individualCount: 1; **Location:** countryCode: BG; locality: Vizitsa Vill.; verbatimElevation: 290; verbatimCoordinates: N42°07'15.0", E27°36'40.0"; geodeticDatum: WGS84; **Event:** eventDate: 06/10/2005; **Record Level:** collectionCode: cWB

#### Polistichus connexus

(Geoffroy, 1785)

##### Materials

- **Type status:** Other material. **Occurrence:** recordedBy: R. Bekchiev; individualCount: 1; **Location:** countryCode: BG; locality: Primorsko, "Arcutino" Place; verbatimElevation: 60; verbatimCoordinates: N42°20'16.0", E27°43'18.1"; geodeticDatum: WGS84; **Event:** eventDate: 30/06/2009
- **Type status:** Other material. **Occurrence:** recordedBy: R. Bekchiev; individualCount: 2; **Location:** countryCode: TR; locality: Sarpdere Vill., Dupnisa Cave surroundings; verbatimElevation: 440; verbatimCoordinates: N41°49'56.3", E27°33'24.0"; geodeticDatum: WGS84; **Event:** eventDate: 08/07/2009
- **Type status:** Other material. **Occurrence:** recordedBy: J. Mařan & K. Taborsky; individualCount: 1; **Location:** countryCode: BG; locality: Maslen nos Cape; **Event:** eventDate: VI.1933; **Record Level:** institutionCode: NMNHS
- **Type status:** Other material. **Location:** countryCode: BG; locality: Ahtopol; **Record Level:** bibliographicCitation: Hieke & Wrase (1988: 163)
- **Type status:** Other material. **Location:** countryCode: BG; locality: Ropotamo; **Record Level:** bibliographicCitation: Hieke & Wrase (1988: 163)

#### Polistichus fasciolatus

(P. Rossi, 1790)

##### Materials

- **Type status:** Other material. **Occurrence:** recordedBy: R. Kostova; individualCount: 1; **Location:** countryCode: BG; locality: Primorsko, Perla Beach; verbatimElevation: 1; verbatimCoordinates: N42°17'10.2", E27°45'13.6"; geodeticDatum: WGS84; **Event:** eventDate: 10/05/2009
- **Type status:** Other material. **Occurrence:** recordedBy: R. Kostova; individualCount: 1; **Location:** countryCode: BG; locality: Primorsko, "Arcutino" Place; verbatimElevation: 60; verbatimCoordinates: N42°20'16.0", E27°43'18.1"; geodeticDatum: WGS84; **Event:** eventDate: 30/06/2009

## Discussion

The present study lists 329 species and subspecies of the Carabidae, belonging to 328 either monotypic or polytypic species. Of these, 328 species and subspecies occur in the investigated region. One species is represented with two subspecies: *Bembidion decorum decorum* and *B. decorum bodemeyeri*.

One species, *Apotomus testaceus* is here excluded from the list of the Bulgarian fauna. The only specimen available of it, mentioned by Guéorguiev (1992) and later catalogued by Guéorguiev and Guéorguiev (1995), was subsequently revised and determined as *Apotomus clypeonitens adanensis*. It is worth noting that, although published as *A. testaceus*, the specimen bears an identification label “*Apotomus rufus* (Rossi) Kryzanovskji det.”, and a subsequently added label: “*Apotomus clypeonitens adanensis* Jedl. B. Guéorguiev det. 2015”.

BULGARIAN PART OF AREA:

Altogether, 315 species occur in the Bulgarian zone. Of these, 84 species are known only by literature data, 77 species represent new species records, and another 155 species are listed both in the literature and recorded in the field. Specimens relating to most of the published taxa have been revised.

Two taxa, one subgenus (*Haptotapinus* Reitter, 1886) and one species (*Pterostichus crassiusculus*) are new to the fauna of Bulgaria. Formerly, Apfelbeck (1904) reported this species from “Balkangebirge”, based on its type locality: “aus der Türkei und dem Balkangebirge” (Reitter 1886). Reitter’s record was discussed by Guéorguiev and Guéorguiev (1995) and the species was not included for the Bulgarian fauna, the precise location of the type locality remains unknown. “Balkangebirge” is a geographic term generally used more than one century ago for various mountains situated between the Danube River and the Aegean Sea, though in recent times it is restricted only to the Balkan Range (= Stara Planina Mts.). In fact, we consider it highly likely that the mountain which is referred to as Reitter’s type locality is Strandzha Mt. However, as we noted in the introduction, the largest area of this massif lies outside Bulgaria and closer to the known area of species distribution, i.e. the surroundings of the Bosphorus. Therefore, the finding near Slivarovo Villlage is certainly the first precise record of *P. crassiusculus* from Bulgaria.

Six species (*Carabus violaceus azurescens*, *Platynus proximus*, *Molops alpestris kalofericus*, *M. dilatatus angulicollis*, *Pterostichus merklii*, and *Calathus metallicus*) are considered to be doubtful for the region, though we have revised material of them. The material on which the above species are based, published by Guéorguiev (1992) and/or Guéorguiev and Guéorguiev (1995), is labelled in the same manner, was recorded on the same date (16.7.1933), and was collected by the same collector (K. Tuleshkov). The question arises because all those species being primarily mountain dwellers, have been reported from a region of low altitude. Another doubtful record for the regional fauna, as well as for the fauna of Bulgaria, is *Apotomus rufus*; we have never seen material of this species from the country. This species has been omitted for Bulgaria in the Palaearctic Catalogue of Carabidae (Wrase 2003).

TURKISH PART OF AREA:

In this area 114 species and subspecies have been confirmed for the Turkish part of the region. Of these, 110 species represent new records for the area, while four species are known from both previous and new records. A total of 43 carabid species and subspecies found in the Turkish part of the study region represent first records for the European part of Turkey. Carabid taxa new to the fauna of Turkey comprise two genera (*Myas*, *Oxypselaphus*), one subgenus (*Feronidius*), nine species and subspecies (*Carabus granulatus granulatus*, *Dyschirius tristis*, *Bembidion normannum apfelbecki*, *B. subcostatum vau*, *Acupalpus exiguus*, *Myas chalybaeus*, *Oxypselaphus obscurus*, *Pterostichus leonisi*, *Pt. melas*). It seems that the ranges of most of these taxa (exept for *B. normannum apfelbecki*) are confined to the European part of the country and their populations do not extend east of the Bosphorus. It should be noted that one of the species (*O. obscurus*) was included in the checklist of the caraboid beetles of Anatolia (Casale and Vigna Taglianti 1999), as "? : occurrence in Anatolia doubtful or presumable, but not confirmed". As we have not found any source citing that species for the country, we treat it as new to the fauna of Turkey. Also, there are seven species which were included for Anatolia by Casale and Vigna Taglianti (1999) ("taxa presumably valid or confirmed") but were subsequently not mentioned for Asian Turkey in the Palaearctic Catalogue of Carabidae (Lӧbl and Smetana 2003). These species are: *Asaphidion flavipes*, *Bembidion assimile*, *B. guttula*, *B. articulatum*, *Tachyura parvula*, *Parophonus maculicornis* and *Polistichus connexus*. We consider that if these species occur in the European territory of the country, they should therefore be considered as taxa confirmed for Turkey.
